# Proteomic profile and predictive markers of outcome in patients with subarachnoid hemorrhage

**DOI:** 10.1186/s12014-024-09493-6

**Published:** 2024-07-23

**Authors:** Sara Diana Lolansen, Nina Rostgaard, Markus Harboe Olsen, Maud Eline Ottenheijm, Lylia Drici, Tenna Capion, Nicolas Hernandez Nørager, Nanna MacAulay, Marianne Juhler

**Affiliations:** 1 Department of Neurosurgery, the Neuroscience Centre, Copenhagen University Hospital – Rigshospitalet, Copenhagen, Denmark; 2 Department of Neuroscience, University of Copenhagen, Copenhagen, Denmark; 3 Department of Neuroanaesthesiology, the Neuroscience Centre, Copenhagen University Hospital – Rigshospitalet, Copenhagen, Denmark; 4 Department of Anaesthesiology, Zealand University Hospital, Køge, Denmark; 5 NNF Center for Protein Research, University of Copenhagen, Copenhagen, Denmark; 6 Department of Clinical Biochemistry, Copenhagen University Hospital – Bispebjerg and Frederiksberg Hospital, Copenhagen, Denmark; 7 Department of Clinical Medicine, Faculty of Health and Medical Sciences, University of Copenhagen, Copenhagen, Denmark

**Keywords:** Subarachnoid hemorrhage, Posthemorrhagic hydrocephalus, Cerebrospinal fluid, Biomarkers, Mass spectrometry, Proteomics

## Abstract

**Background:**

The molecular mechanisms underlying development of posthemorrhagic hydrocephalus (PHH) following subarachnoid hemorrhage (SAH) remain incompletely understood. Consequently, treatment strategies tailored towards the individual patient remain limited. This study aimed to identify proteomic cerebrospinal fluid (CSF) biomarkers capable of predicting shunt dependency and functional outcome in patients with SAH in order to improve informed clinical decision making.

**Methods:**

Ventricular CSF samples were collected twice from 23 patients with SAH who required external ventricular drain (EVD) insertion (12 patients with successful EVD weaning, 11 patients in need of permanent CSF shunting due to development of PHH). The paired CSF samples were collected acutely after ictus and later upon EVD removal. Cisternal CSF samples were collected from 10 healthy control subjects undergoing vascular clipping of an unruptured aneurysm. All CSF samples were subjected to mass spectrometry-based proteomics analysis. Proteomic biomarkers were quantified using area under the curve (AUC) estimates from a receiver operating curve (ROC).

**Results:**

CSF from patients with SAH displayed a distinct proteomic profile in comparison to that of healthy control subjects. The CSF collected acutely after ictus from patients with SAH was moreover distinct from that collected weeks later but appeared similar in the weaned and shunted patient groups. Sixteen unique proteins were identified as potential predictors of shunt dependency, while three proteins were identified as potential predictors of functional outcome assessed six months after ictus with the modified Rankin Scale.

**Conclusions:**

We here identified several potential proteomic biomarkers in CSF from patients with SAH capable of predicting (i) shunt dependency and thus development of PHH and (ii) the functional outcome assessed six months after ictus. These proteomic biomarkers may have the potential to aid clinical decision making by predicting shunt dependency and functional outcome following SAH.

**Supplementary Information:**

The online version contains supplementary material available at 10.1186/s12014-024-09493-6.

## Background

Aneurysmal subarachnoid hemorrhage (SAH) is associated with high mortality and morbidity [1]. Despite neurosurgical intervention and specialized neuro-intensive care, more than one third of patients with SAH develop an unfavorable long-term functional outcome. An unfavorable outcome is characterized by an affected cognitive performance including impaired memory and executive functions as well as sleep disturbances and fatigue [2, 3] and it remains incompletely understood why some patients experience worse outcomes [4]. Immediately after the hemorrhage, extravasated blood enters the cerebrospinal fluid (CSF) spaces and the intracranial pressure (ICP) rises. Several pathological alterations commonly occur within minutes to days, i.e., cerebral vasospasms, acute and delayed brain ischemia, inflammation, seizures, and cortical spreading depression that all contribute to the risk of an unfavorable outcome [5–8]. The molecular mechanisms underlying the initial injury expansion following SAH and the factors contributing to an unfavorable outcome remain to be fully elucidated, which limits effective (pharmaceutical) treatment options [9]. Posthemorrhagic hydrocephalus (PHH) is a common and serious complication of SAH. Extravasated blood can obstruct the CSF drainage pathways either directly via formation of blood clots or indirectly by promoting meningeal inflammation and subsequent fibrosis [8, 10, 11]. Hypersecretion by the CSF-producing tissue, the choroid plexus, in response to the sudden presence of hemorrhage-derived molecules or inflammatory markers in the CSF may, moreover, contribute to the pathological CSF accumulation observed in PHH [11–14]. Acute PHH is often treated with external ventricular drainage (EVD) to alleviate the ICP and mitigate neurological damage [15, 16]. Although lifesaving, EVD placement comes with a significant risk of infection, which increases with the duration of the EVD [17–20]. ICP alleviation weighed against removal of the EVD as early as possible is often a difficult clinical decision in management of PHH in the acute and subacute phase. In some patients with SAH, CSF dynamics normalize after the initial hemorrhage, and they can successfully be weaned from the EVD. However, a considerable proportion of the patients fails EVD weaning and undergoes subsequent shunt insertion due to development of chronic PHH. A recent review reported the published risk of developing shunt-dependent PHH after SAH to be between 8 and 63% [21]. Such wide variation implies uncertainty regarding both the acute and subacute management of PHH and prediction of shunt-dependent chronic PHH development [15, 16, 22, 23]. Although several publications have investigated clinical, radiological, and treatment features as predictive indicators for PHH development following SAH, none have proven valid as predictors of shunt dependency [15, 16, 22–29]. Our group recently identified a subset of inflammatory proteins in ventricular CSF from patients with SAH as potential biomarkers of shunt dependency and functional outcome [30]. This suggests that neuroinflammation plays a pivotal role in the development of PHH following SAH. However, to delineate the precise pathological alterations following SAH and elucidate why some patients develop PHH while others do not, the molecular and cellular alterations occurring within the brain of patients with SAH require further investigation. Here, we assessed the proteomic profile in CSF from patients with SAH by comparison to CSF from healthy individuals with unruptured aneurysms. We quantified whether certain proteomic patterns could be used to distinguish patients with SAH who went on to develop PHH from those who did not, and we identified possible biomarkers of shunt dependency and functional outcome. Such biomarkers could potentially serve as clinical tools in management of SAH.

## Methods

### Patients and CSF collection

This study included paired CSF samples collected from 23 patients with SAH (median age: 61 years; range: 34–77 years; 18 F/5 M) who were diagnosed and treated at the Department of Neurosurgery, Rigshospitalet, Copenhagen, Denmark, between June 2019 and September 2021. The CSF samples were collected as part of a prospective trial on EVD weaning (clinical trial identifier: NCT03948256 [31]). The first CSF samples (“start samples”) were collected from the SAH patients through their EVD either acutely within 24 h of ictus (*n* = 19) or as soon as possible thereafter (*n* = 4). The time interval from ictus to first CSF collection was on average 23 h, range: 2 h – 7 days. Of the 23 patients with SAH, 12 patients could be successfully weaned off the EVD (“weaned”) and did not require further CSF diversion, while 11 patients underwent ventriculoperitoneal shunt surgery upon EVD weaning due to chronic PHH development (“shunted”). The last CSF samples (“end samples”) were collected prior to shunt insertion or EVD withdrawal (*n* = 23 in total). The time interval between the paired CSF samples (start and end) was on average 19 days, range: 5–30 days. The CSF samples from the patients with SAH were collected directly for analytical purposes and the CSF therefore did not reside in the EVDs prior to collection. The control group consisted of 10 patients with unruptured aneurysms undergoing preventive surgery (vascular clipping) (median age: 60 years, range: 39–71 years, 6 F/4 M) from whom the CSF was collected from the basal cisterns during surgery prior to clipping of the aneurysm. All CSF samples were centrifuged at 2000 × *g* for 10 min at 4 °C within 2 h from collection, and the supernatant aliquoted in polypropylene microtubes (Sarstedt, Nümbrecht, Germany) and subsequently stored at -80 °C [32].

Functional outcome was assessed for each SAH patient six months after ictus by the modified Rankin Scale (mRS) [33]. Thirteen patients had a favorable functional outcome (mRS 0–2), while 10 patients had an unfavorable functional outcome (mRS 3–6), including two deceased patients (mRS 6). Written informed consent was obtained from all patients or their next of kin, depending on the capability of the patients, and the study was approved by the Danish National Committee on Health Research Ethics (approval no. H-19001474 and H-17011472/69197) and the Danish Data Protection Agency (VD-2019-210). Aliquots of the CSF samples have been analyzed for other components in unrelated studies [11, 30, 34–36].

### Protein digestion and evotips loading

Human CSF sample preparation was performed on an Agilent Bravo Liquid Handling Platform (Agilent) according to an optimized version of previously published protocols [37, 38]. Briefly, CSF samples were aliquoted into a 96-well format plate and introduced to the Bravo Robot (Agilent). 20 µl CSF sample was mixed with 30 µl PreOmics Lysis buffer (P.O. 00001, PreOmics GmbH) and incubated at 95 °C for 10 min in order to denature proteins, reduce disulfide bridges, and alkylate cysteines [39]. After cooling the sample for 15 min at room temperature, trypsin and LysC (0.5 µg/µl, Promega) were added in a ratio of 1 µg enzyme to 100 µg proteins and the mixture incubated at 37 °C for 4 h. Similar protein quantity was observed in tested random samples (on average five samples per plate) using Nanodrop. The peptide mixtures were diluted in 100 µl 99% isopropanol, 1% Trifluoro-acetic acid (TFA) and desalted using two-gauge reversed-phase styrenedivinylbenzene (SDB-RPS) stage-tips. Afterwards, the stage-tips were washed using 200 µl 99% isopropanol, 1% TFA, followed by 200 µl 0.2% TFA. The purified peptides were eluted using 80% acetonitrile (VWR chemicals) containing 1% ammonia (Merck) and subsequently dried down. Peptides were resuspended in solvent A (0.1% formic acid (FA) in water) and loaded onto Evotips (Evosep Biosystem, Denmark) according to the manufacturer’s recommendations. The Evotips were wetted with isopropanol for 5 min, activated with 20 µl solvent B (99% CAN, 0.1% FA) and centrifuged at 700 x *g* for 1 min. 20 µl of solution A was then added to equilibrate the tips followed by sample loading. Finally, 20 µl buffer A was used to wash the Evotip and 100 µl was added to avoid drying.

### Liquid chromatography and mass spectrometry (MS) analysis

The samples were injected in single shots into an Exploris 480 Thermo Fischer Scientific system using Evosep One (Evosep Biosystem). A preset chromatographic method was used corresponding to 60 samples per day. The peptides were separated on an 8 cm Pepsep column (150 μm, ID 1.5 μm bead size Reprosil-Pur C18 beads, Marslev, Denmark) at 1 µl/min flow rate with a 21 min gradient. The heated capillary temperature was set to 275 °C, the spray voltage to 2300 V and the funnel radiofrequency to 40 Hz. The mass spectrometer was operated in a data-independent mode (DIA) with a full MS range from 350 to 1650 m/z at a resolution of 60,000 at 200 m/z. The AGC target was set to 300% with an injection time of 50 ms. The AGC value of the targeted MS2 experiment was set to 1000%. Twenty-two windows of variable sizes were defined for target MS2 (tMS2) acquisition and subjected to high-energy collisional dissociation (HCD) fragmentation with a normalized collision energy at 30%. Each tMS2 scan was acquired at a resolution of 30,000 with a maximum ion injection time (IT) of 28 ms for a scan range of m/z 349.5 to 1650.5. Window sizes are provided in Supplementary file 3, Supplementary Table 7.

### Data handling

The MS raw files were processed with Spectronaut version 15 (Biognosys, Switzerland). A CSF spectral library, generated in connection with an unpublished CSF study, was imported from MaxQuant software analyses. The library contained 2733 protein groups and 17,301 peptides. DIA files were searched against the library using default parameters except for the normalization, which was set to local, as it offered more stringent criteria for data analysis and thus helps avoid false hits. Dynamic mass and retention time tolerances (for both MS1 and MS2) were applied. Q-value cutoff was set to 1% both at precursor and protein level using a mutated decoy method [40]. The calibration was performed based on a local regression model [41]. The MS2-based quantitation was used for all further analysis. Protein data was exported from Spectronaut and further processed using the clinical knowledge graph (CKG) [42] together with their matching experimental and clinical data. Intensities were log-transformed before further statistical analysis.

### Statistical analysis

Statistical analyses were carried out using R v. 4.1.0 (R Core Team, Vienna, Austria). Unpaired analyses were conducted for comparisons between healthy control subjects and SAH patients, while paired analyses were conducted for comparisons using CSF samples collected from the same SAH patients (start versus end). Continuous data were presented as mean and standard deviation or median and interquartile range depending on normality. Proteomics data were analyzed to assess which proteins were measured above the detection level. As cutoff, only proteins detectable in at least five CSF samples from healthy control subjects (corresponding to 50% of the control subjects) and 10 CSF samples from patients with SAH (corresponding to 43% of the SAH patients) were included in subsequent analyses. A principal component analysis (PCA) plot with complete case analysis was conducted to reveal systematic group differences [43]. A Volcano plot was used to investigate if protein abundance was higher in one group compared to another, similar between groups, or unclear. Statistical analysis was performed with Student’s t-test as indicated in figure and table legends, and *P*-values were corrected for multiple testing using a Bonferroni correction. Since data initially were skewed, but logarithmic transformed before analyses, the normality assumption was met. Proteins with adjusted *P*-values < 0.05 and a fold change in protein abundance > 2 were assigned as significantly different between groups to ensure that minor variation would not induce too many false positives. Conversely, proteins with similar abundance in groups were defined by uncorrected *P*-values > 0.05 and a fold change in protein abundance < 2. Lack of blood contamination of the CSF samples was verified by quantification of blood proteins (Supplementary file 1, Supplementary Fig. S1-S2). The sensitivity and specificity of CSF proteins for prediction of shunt dependency and/or functional outcome in SAH patients were assessed using receiver operating characteristic (ROC) curve analysis, and the corresponding area under the curve (AUC) values were computed as a measure of accuracy. CSF proteins with a lower confidence limit ≥ 0.7 using the AUC with a 95% confidence interval (CI) were considered potential predictors. The lower confidence limit of ≥ 0.7 was selected to ensure predictions of moderate to high accuracy [44].

## Results

### CSF from patients with SAH displays a distinct proteomic profile

**Fig. 1:**
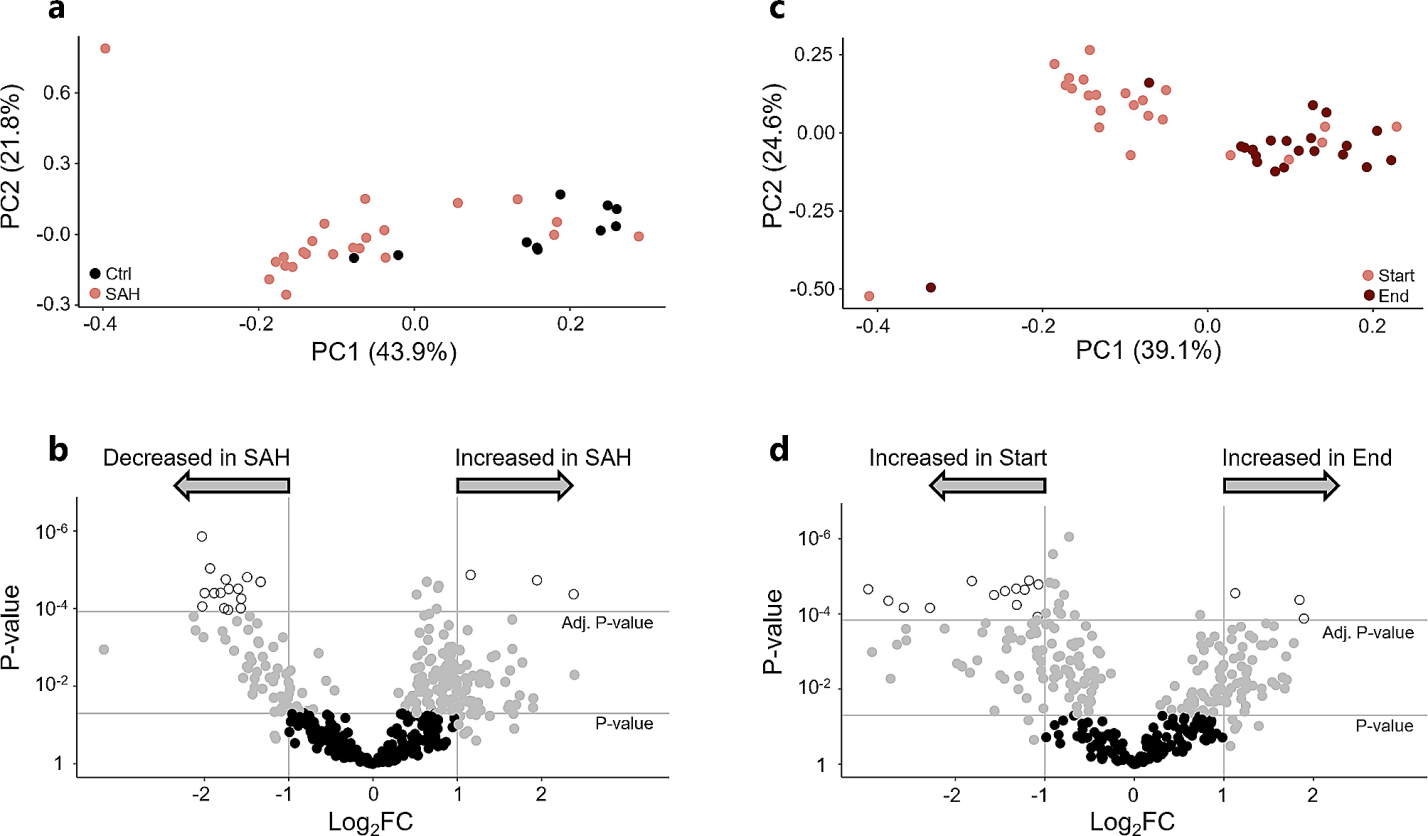
Proteomic profile of CSF from healthy control subjects and patients with SAH over time. **a** PCA plot of the proteomic profiles of patients with SAH (*n* = 23) and healthy control subjects (*n* = 10). **b** Volcano plot of proteomic data from patients and control subjects from panel a. Ctrl: controls; SAH: subarachnoid hemorrhage. **c** PCA plot of the CSF samples collected upon EVD placement (start samples, light red) and just before EVD removal (end samples, dark red) from patients with SAH (*n* = 23). **d** Volcano plot of proteomic data from patients with SAH (*n* = 23) employing both the start and end samples. Significance threshold was defined as a two-fold change in protein abundance combined with statistical significance with a t-test followed by Bonferroni correction (*P* < 0.05). Grey dots represent proteins, which were categorized as *unclear*, either not reaching a significant Bonferroni-corrected *P*-value or a two-fold change in protein abundance. Black dots represent proteins found in *similar* abundance in the two groups

To determine whether the proteomic profile of CSF from patients with SAH differed from that of healthy control subjects, ventricular CSF collected upon EVD placement (start samples) from patients with SAH was compared to cisternal CSF collected from healthy control subjects undergoing vascular clipping of an unruptured aneurysm. Principal component analysis (PCA) of the CSF protein abundance revealed no clear overall clustering that could distinguish the SAH patient group from the group of healthy control subjects (Fig. 1a). Of the 1,205 unique proteins identified in the CSF samples using MS-based proteomics, 416 proteins met the requirements for comparison (detected in at least five control samples and 10 SAH start samples). Proteomic analysis employing a volcano plot for visualization (Fig. 1.b) revealed that three proteins were significantly increased in abundance in the CSF from patients with SAH when compared to that of healthy control subjects: immunoglobulin heavy variable 1–2 (IGHV1-2), protein S100-B (S100B), and serum amyloid A-1 protein (SAA1), Table 1. Conversely, 15 proteins were significantly decreased in abundance in the SAH patient CSF: amyloid beta precursor like protein 1 (APLP1), amyloid-beta precursor protein (APP), chromogranin-A (CHGA), **s**ecretogranin-1 (CHGB), neural cell adhesion molecule L1-like protein (CHL1), calsyntenin-1 (CLSTN1), beta-Ala-His dipeptidase (CNDP1), dickkopf WNT signaling pathway inhibitor 3 (DKK3), neural cell adhesion molecule 1 (NCAM1), neuronal cell adhesion molecule (NRCAM), proSAAS (PCSK1N), prostaglandin-H2 D-isomerase (PTGDS), **s**ecretogranin-3 (SCG3), SPARC-like protein 1 (SPARCL1), and thy-1 cell surface antigen (THY1), Table 2. Of the remaining 398 proteins, 213 proteins were detected in similar abundance in the SAH patient CSF and control CSF, while 185 proteins were categorized as unclear (Supplementary file 2, Supplementary Table 1).

**Table 1:** P**roteins significantly more abundant in CSF from patients with SAH versus healthy control subjects**

Protein Name	Uniprot ID	Ctrl Mean (SD) [*N*] in a.u.	SAH start Mean (SD) [*N*] in a.u.	log_2_FC	*P*-value	Adj. *P*-value
IGHV1-2	P23083	15.1 (0.5) [9]	16.3 (0.5) [19]	1.16	< 0.001	0.006
S100B	P04271	13.8 (0.8) [7]	16.2 (1.3) [20]	2.38	< 0.001	0.018
SAA1	P0DJI8	14.4 (0.6) [7]	16.3 (1.1) [19]	1.95	< 0.001	0.008

Ctrl: control subjects; SAH: subarachnoid hemorrhage; a.u.: arbitrary units; SD: standard deviation; N: number of subjects/patients; log_2_FC: log_2_(fold change); IGHV1-2: Immunoglobulin heavy variable 1–2; S100B: Protein S100-B; SAA1: Serum amyloid A-1 protein. Statistical significance was determined with Student’s t-test with Bonferroni correction

**Table 2:** **Proteins significantly less abundant in CSF from patients with SAH versus healthy control subjects**

Protein Name	Uniprot ID	Ctrl Mean (SD) [*N*] in a.u.	SAH start Mean (SD) [*N*] in a.u.	log_2_FC	*P*-value	Adj. *P*-value
APLP1	B7Z4G8	16.4 (0.7) [10]	14.5 (1.2) [20]	-1.93	< 0.001	0.004
APP	P05067	15.2 (0.8) [10]	13.4 (1.0) [20]	-1.75	< 0.001	0.007
CHGA	P10645	16.1 (0.7) [10]	14.5 (1.0) [18]	-1.60	< 0.001	0.013
CHGB	P05060	16.0 (0.6) [10]	14.0 (1.2) [21]	-2.03	< 0.001	0.001
CHL1	O00533	15.4 (0.8) [10]	13.6 (1.1) [19]	-1.81	< 0.001	0.016
CLSTN1	O94985	16.4 (0.7) [10]	14.9 (0.9) [15]	-1.56	< 0.001	0.023
CNDP1	Q96KN2	16.7 (0.6) [10]	15.2 (1.0) [22]	-1.49	< 0.001	0.006
DKK3	F6SYF8	16.2 (0.7) [10]	14.4 (1.4) [20]	-1.77	< 0.001	0.041
NCAM1	P13591	15.6 (0.7) [10]	14.1 (1.2) [20]	-1.57	< 0.001	0.041
NRCAM	C9JYY6	15.8 (0.8) [10]	14.1 (1.0) [16]	-1.72	< 0.001	0.045
PCSK1N	Q9UHG2	15.7 (0.7) [10]	14.0 (1.2) [21]	-1.71	< 0.001	0.013
PTGDS	P41222	20.1 (0.7) [10]	18.1 (1.9) [23]	-2.03	< 0.001	0.037
SCG3	Q8WXD2	15.9 (0.6) [10]	14.0 (1.2) [16]	-1.88	< 0.001	0.017
SPARCL1	Q14515	14.8 (0.6) [10]	13.5 (0.9) [21]	-1.33	< 0.001	0.009
THY1	E9PIM6	16.9 (0.9) [10]	14.9 (1.2) [19]	-2.00	< 0.001	0.017

Ctrl: control subjects; SAH: subarachnoid hemorrhage; a.u.: arbitrary units; SD: standard deviation; N: number of subjects/patients; log_2_FC: log_2_(fold change); APLP1: Amyloid beta precursor like protein 1; APP: Amyloid-beta precursor protein; CHGA: Chromogranin-A; CHGB: Secretogranin-1; CHL1: Neural cell adhesion molecule L1-like protein; CLSTN1: Calsyntenin-1; CNDP1: Beta-Ala-His dipeptidase; DKK3: Dickkopf WNT signaling pathway inhibitor 3; NCAM1: Neural cell adhesion molecule 1; NRCAM: Neuronal cell adhesion molecule; PCSK1N: ProSAAS; PTGDS: Prostaglandin-H2 D-isomerase; SCG3: Secretogranin-3; SPARCL1: SPARC-like protein 1; THY1: Thy-1 cell surface antigen. Statistical significance was determined with Student’s t-test with Bonferroni correction

### The proteomic profile in CSF from patients with SAH changes over time

To elucidate whether the progression of SAH from ictus to EVD removal was associated with alterations in CSF protein composition, the proteomic profiles of paired CSF samples collected upon EVD placement (start samples) and EVD removal (end samples) from patients with SAH were compared. PCA revealed a tendency towards clustering of the start and end samples into distinct proteomic distributions (Fig. 1c), indicating that the proteomic profile of SAH patient CSF may change over time after the initial hemorrhagic event. Of the 344 unique proteins detected in the CSF samples that met requirements for comparison (detected in at least 10 start samples and 10 end samples), 13 proteins were significantly increased in abundance in the start samples: serum amyloid P-component (APCS), apolipoprotein B-100 (APOB), apolipoprotein C-I (APOC1), complement component C6 (C6), carboxypeptidase N subunit 2 (CPN2), coagulation factor X (F10), insulin-like growth factor-binding protein complex acid labile subunit (IGFALS), IGHV1-2, immunoglobulin heavy variable 3–49 (IGHV3-49), inter-alpha-trypsin inhibitor heavy chain H2 (ITIH2), plasma kallikrein (KLKB1), **s**erum paraoxonase/arylesterase 1 (PON1), and S100B, Fig. 1d; Table 3. Conversely, three proteins were significantly increased in abundance in the end samples: apolipoprotein E (APOE), monocyte differentiation antigen CD14 (CD14), and lysozyme (LYZ), Fig. 1d; Table 4. Of the 328 remaining proteins, 140 proteins were detected in similar abundance in the start and end samples, while 188 proteins were categorized as unclear (Supplementary file 2, Supplementary Table 2).

**Table 3:** **Proteins significantly more abundant in the CSF samples collected acutely after ictus (start samples)**

Protein Name	Uniprot ID	SAH Start Mean (SD) [*N*] in a.u.	SAH End Mean (SD) [*N*] in a.u.	log_2_FC	*P*-value	Adj. *P*-value
APCS	P02743	17.7 (1.6) [19]	15.1 (1.3) [19]	-2.58	< 0.001	0.023
APOB	P04114	18.9 (2.0) [21]	16.0 (1.3) [21]	-2.97	< 0.001	0.008
APOC1	K7ERI9	18.1 (1.7) [20]	15.9 (1.0) [20]	-2.28	< 0.001	0.024
C6	P13671	16.4 (0.8) [20]	15.3 (0.5) [20]	-1.08	< 0.001	0.041
CPN2	P22792	16.8 (1.0) [20]	15.3 (0.8) [20]	-1.45	< 0.001	0.008
F10	P00742	15.5 (0.7) [13]	14.2 (0.5) [13]	-1.32	< 0.001	0.007
IGFALS	P35858	16.0 (0.8) [19]	14.8 (0.7) [19]	-1.23	< 0.001	0.008
IGHV1-2	P23083	16.3 (0.6) [15]	15.2 (0.8) [15]	-1.07	< 0.001	0.006
IGHV3-49	A0A0A0MS15	18.0 (0.8) [20]	16.8 (0.7) [20]	-1.18	< 0.001	0.004
ITIH2	P19823	17.1 (0.9) [22]	15.8 (0.7) [22]	-1.31	< 0.001	0.020
KLKB1	H0YAC1	15.7 (0.9) [19]	14.1 (0.7) [19]	-1.57	< 0.001	0.011
PON1	P27169	17.5 (1.1) [20]	15.7 (1.0) [20]	-1.82	< 0.001	0.005
S100B	P04271	16.2 (1.3) [12]	13.5 (1.1) [12]	-2.75	< 0.001	0.015

SAH: subarachnoid hemorrhage; a.u.: arbitrary units; SD: standard deviation; N: number of patients; log_2_FC: log_2_(fold change); APCS: Serum amyloid P-component; APOB: Apolipoprotein B-100; APOC1: Apolipoprotein C-I; C6: Complement component C6; CPN2: Carboxypeptidase N subunit 2; F10: Coagulation factor X; IGFALS: Insulin-like growth factor-binding protein complex acid labile subunit; IGHV1-2: Immunoglobulin heavy variable 1–2; IGHV3-49: Immunoglobulin heavy variable 3–49; ITIH2: Inter-alpha-trypsin inhibitor heavy chain H2; KLKB1: Plasma kallikrein; PON1: Serum paraoxonase/arylesterase 1; S100B: Protein S100-B. Statistical significance was determined with Student’s t-test with Bonferroni correction

**Table 4:** Proteins significantly more abundant in the CSF samples collect upon EVD removal (end samples)

Protein Name	Uniprot ID	SAH Start Mean (SD) [*N*] in a.u.	SAH End Mean (SD) [*N*] in a.u.	log_2_FC	*P*-value	Adj. *P*-value
APOE	P02649	16.6 (0.8) [22]	17.7 (0.6) [22]	1.13	< 0.001	0.010
CD14	P08571	15.1 (1.3) [20]	17.0 (1.0) [20]	1.89	< 0.001	0.046
LYZ	A0A0B4J259	15.7 (1.3) [20]	17.5 (0.9) [20]	1.84	< 0.001	0.015

SAH: subarachnoid hemorrhage; a.u.: arbitrary units; SD: standard deviation; N: number of patients; log_2_FC: log_2_(fold change); APOE: Apolipoprotein E; CD14: Monocyte differentiation antigen CD14; LYZ: Lysozyme. Statistical significance was determined with Student’s t-test with Bonferroni correction

### The proteomic profile is similar in weaned and shunted patients with SAH

**Fig. 2:**
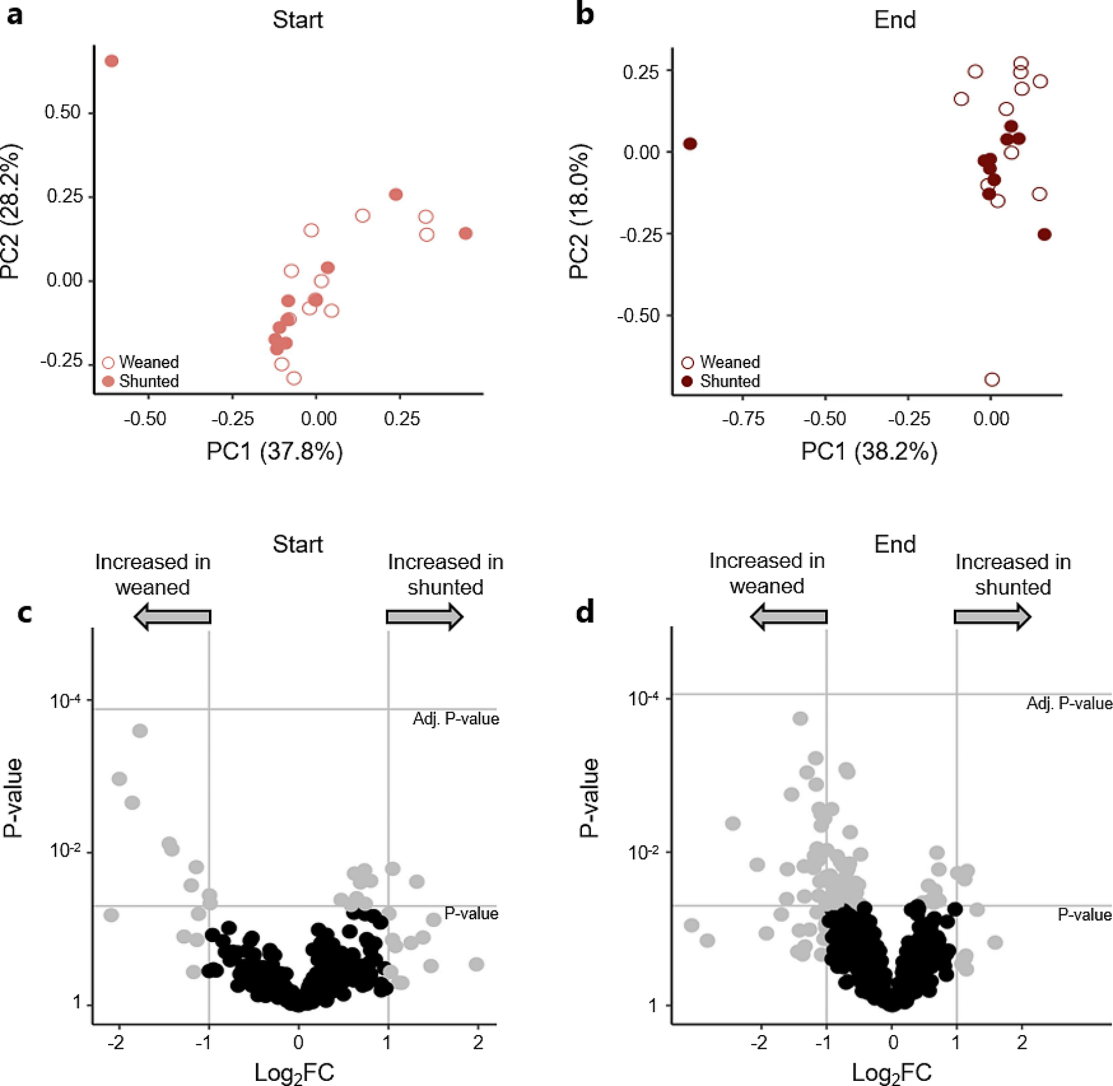
Weaned and shunted SAH patients display a similar proteomic profile. **a** PCA plot of the start samples (light red) collected from weaned (open circles, *n* = 12) and shunted (filled circles, *n* = 11) patients with SAH. **b** PCA plot of the end samples (dark red) collected from weaned (open circles, *n* = 12) and shunted (filled circles, *n* = 11) patients with SAH. **c** Volcano plot of proteomic data (*n* = 380 proteins) using the start samples collected from patients with SAH (*n* = 23). Significance threshold was defined as a two-fold change in protein abundance combined with statistical significance with a t-test followed by Bonferroni correction. Grey dots represent proteins, which were categorized as *unclear* (*n* = 35 proteins), either not reaching a significant Bonferroni-corrected *P*-value or a two-fold change in protein abundance. Black dots represent proteins found in *similar* abundance in the start samples from weaned and shunted SAH patients (*n* = 345 proteins). **d** Volcano plot of proteomic data (*n* = 577 proteins) using the end samples collected from patients with SAH (*n* = 23 patients). Significance threshold was defined as a two-fold change in protein abundance and a significant Bonferroni corrected *P*-value. Grey dots represent proteins which were categorized as *unclear* (*n* = 91 proteins), either not reaching a significant Bonferroni-corrected *P* value or a two-fold change in protein abundance. Black dots represent proteins found in *similar* abundance in the end samples from weaned and shunted SAH patients (*n* = 486 proteins)

To determine whether shunted SAH patients displayed a distinct proteomic CSF profile in comparison to patients who could be successfully weaned from their EVD, we compared the proteomic profiles of the two patient groups (weaned: *n* = 12; shunted: *n* = 11, see *Methods*) using both the CSF samples collected upon EVD placement (start samples) and EVD removal (end samples). Regardless of the time of CSF collection (start samples and end samples), PCA revealed no clear clustering of the weaned and shunted SAH patient groups, albeit a tendency towards clustering of the end sample was apparent (Fig. 2a-b). In agreement, none of the proteins identified using MS-based proteomics were detected at significantly different levels in either the start samples or the end samples (Fig. 2c-d). All proteins identified in the start and end samples are listed in Supplementary file 2, Supplementary Tables 3–4.

### Predictors of shunt dependency and functional outcome

**Fig. 3:**
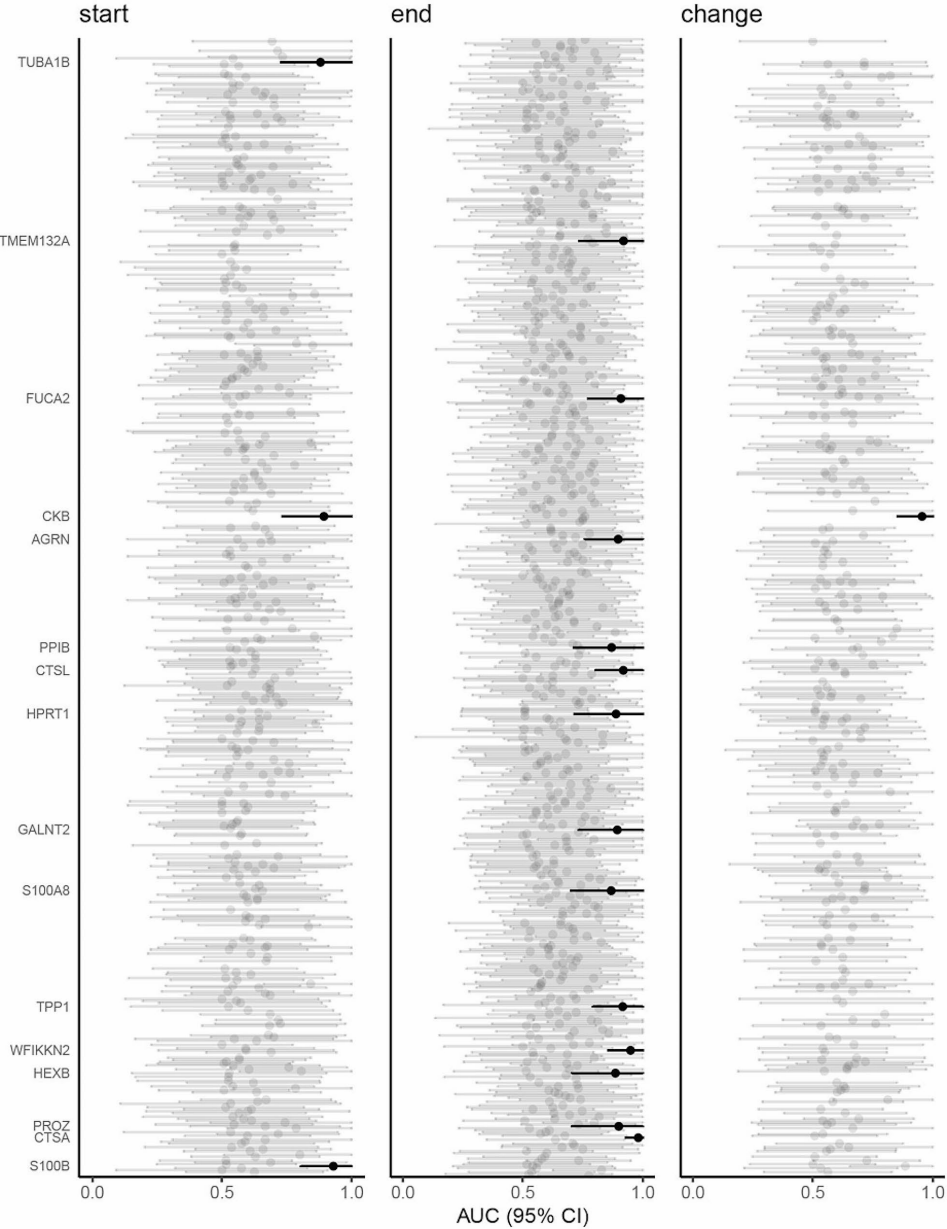
Potential proteomic predictors of shunt dependency. The figure illustrates the calculated AUC and 95% CI for prediction of shunt dependency in patients with SAH (*n* = 23), employing the proteins detected in the start samples, the end samples, and the calculated average change in protein abundance per day. Proteins that reached a lower confidence limit of ≥ 0.7 were accepted as possible predictors (black lines). AUC: area under the curve; CI: confidence interval

To determine whether certain proteins detected in the CSF from patients with SAH could serve as potential predictors of shunt dependency, the proteomic data were analyzed by a receiver operating curve (ROC). Proteins with a lower confidence limit of ≥ 0.7 using the area under the curve (AUC) with a 95% confidence interval were considered potential predictors of shunt dependency. A total of 16 unique proteins had a lower confidence limit of ≥ 0.7 and could thus be considered potential predictors of shunt dependency (Fig. 3; Table 5). For the start samples, three proteins were identified as possible predictors of shunt dependency: creatine kinase B-type (CKB), S100B, and tubulin alpha-1B chain (TUBA1B). For the end samples, 13 proteins were identified as possible predictors of shunt dependency: agrin (AGRN), lysosomal protective protein (CTSA), procathepsin L (CTSL), plasma alpha-L-fucosidase, (FUCA2), polypeptide N-acetylgalactosaminyltransferase 2 (GALNT2), hexosaminidase subunit beta (HEXB), hypoxanthine-guanine phosphoribosyltransferase (HPRT1), peptidyl-prolyl cis-trans isomerase B (PPIB), vitamin K-dependent protein Z (PROZ), protein S100-A8 (S100A8), transmembrane protein 132 A (TMEM132A), tripeptidyl-peptidase 1 (TPP1), and WAP, follistatin/kazal, immunoglobulin, kunitz and netrin domain containing 2 (WFIKKN2). Considering the average change in protein abundance per day, only one protein, CKB, was identified as a possible predictor of shunt dependency. The remaining proteins that did not reach a lower confidence limit of ≥ 0.7 are listed in Supplementary file 2, Supplementary Table 5.

**Table 5:** **Possible predictors of shunt dependency in SAH patients**

Protein Name	Uniprot ID	Time	SAH Weaned Mean (SD) [*N*] in a.u.	SAH Shunted Mean (SD) [*N*] in a.u.	AUC (95%CI)	Cut-off	Sens.	Spec.
AGRN	O004686	end	14.2 (0.4) [11]	13.2 (0.9) [8]	0.90 (0.76-1.00)	13.9	0.88	0.82
CKB	P12277	start	17.3 (1.4) [12]	15.3 (0.8) [7]	0.89 (0.73-1.00)	15.8	0.86	0.92
CKB	P12277	change	0.23 (0.18) [9]	-0.05 (0.15) [5]	0.96 (0.85-1.00)	0.13	1.00	0.89
CTSA	P10619	end	13.4 (0.6) [9]	12.2 (0.4) [6]	0.98 (0.93-1.00)	12.8	1.00	0.89
CTSL	P07711	end	15.2 (0.9) [11]	14.1 (0.4) [9]	0.92 (0.80-1.00)	14.6	1.00	0.73
FUCA2	Q9BTY2	end	14.4 (1.1) [11]	12.8 (0.8) [9]	0.91 (0.77-1.00)	13.8	1.00	0.82
GALNT2	Q10471	end	13.8 (0.4) [11]	13.1 (0.3) [6]	0.89 (0.73-1.00)	13.3	0.83	0.91
HEXB	P07686	end	14.4 (1.2) [11]	13.3 (0.5) [8]	0.89 (0.70-1.00)	14.0	1.00	0.82
HPRT1	P00492	end	14.5 (0.5) [9]	13.6 (0.7) [6]	0.89 (0.72-1.00)	14.2	1.00	0.78
PPIB	P23284	end	15.0 (0.9) [12]	13.9 (0.6) [9]	0.87 (0.71-1.00)	14.3	0.89	0.83
PROZ	P22891	end	13.2 (0.6) [10]	13.9 (0.3) [7]	0.90 (0.70-1.00)	13.4	1.00	0.90
S100A8	P05109	end	18.3 (1.7) [11]	15.9 (1.6) [9]	0.87 (0.70-1.00)	17.7	0.89	0.82
S100B	P04271	start	17.0 (1.0) [11]	15.2 (0.8) [9]	0.93 (0.80-1.00)	16.4	1.00	0.82
TMEM132A	Q24JP5	end	14.0 (0.8) [5]	12.8 (0.5) [5]	0.92 (0.74-1.00)	13.1	0.80	1.00
TPP1	O14773	end	14.6 (0.9) [12]	13.3 (0.5) [7]	0.92 (0.79-1.00)	13.8	0.86	0.83
TUBA1B	P68363	start	17.3 (1.5) [12]	15.5 (1.0) [9]	0.88 (0.73-1.00)	16.4	1.00	0.67
WFIKKN2	C9J6G4	end	15.3 (0.7) [11]	13.9 (0.6) [9]	0.95 (0.86-1.00)	14.5	0.89	0.91

SAH: subarachnoid hemorrhage; a.u.: arbitrary units; SD: standard deviation; N: number of patients; AUC: area under the curve; CI: confidence interval; sens: sensitivity; spec: specificity; AGRN: Agrin; CKB: Creatine kinase B-type; CTSA: Lysosomal protective protein; CTSL: Procathepsin L; FUCA2: Plasma alpha-L-fucosidase; GALNT2: Polypeptide N-acetylgalactosaminyltransferase 2; HEXB: hexosaminidase subunit beta; HPRT1: Hypoxanthine-guanine phosphoribosyltransferase; PPIB: Peptidyl-prolyl cis-trans isomerase B; PROZ: Vitamin K-dependent protein Z; S100A8: Protein S100-A8; S100B: Protein S100-B; TMEM132A: Transmembrane protein 132 A; TPP1: Tripeptidyl-peptidase 1; TUBA1B: Tubulin alpha-1B chain; WFIKKN2: WAP, follistatin/kazal, immunoglobulin, kunitz and netrin domain containing 2. Statistical significance was determined with Student’s t-test with Bonferroni correction

### Predictors of functional outcome

**Fig. 4:**
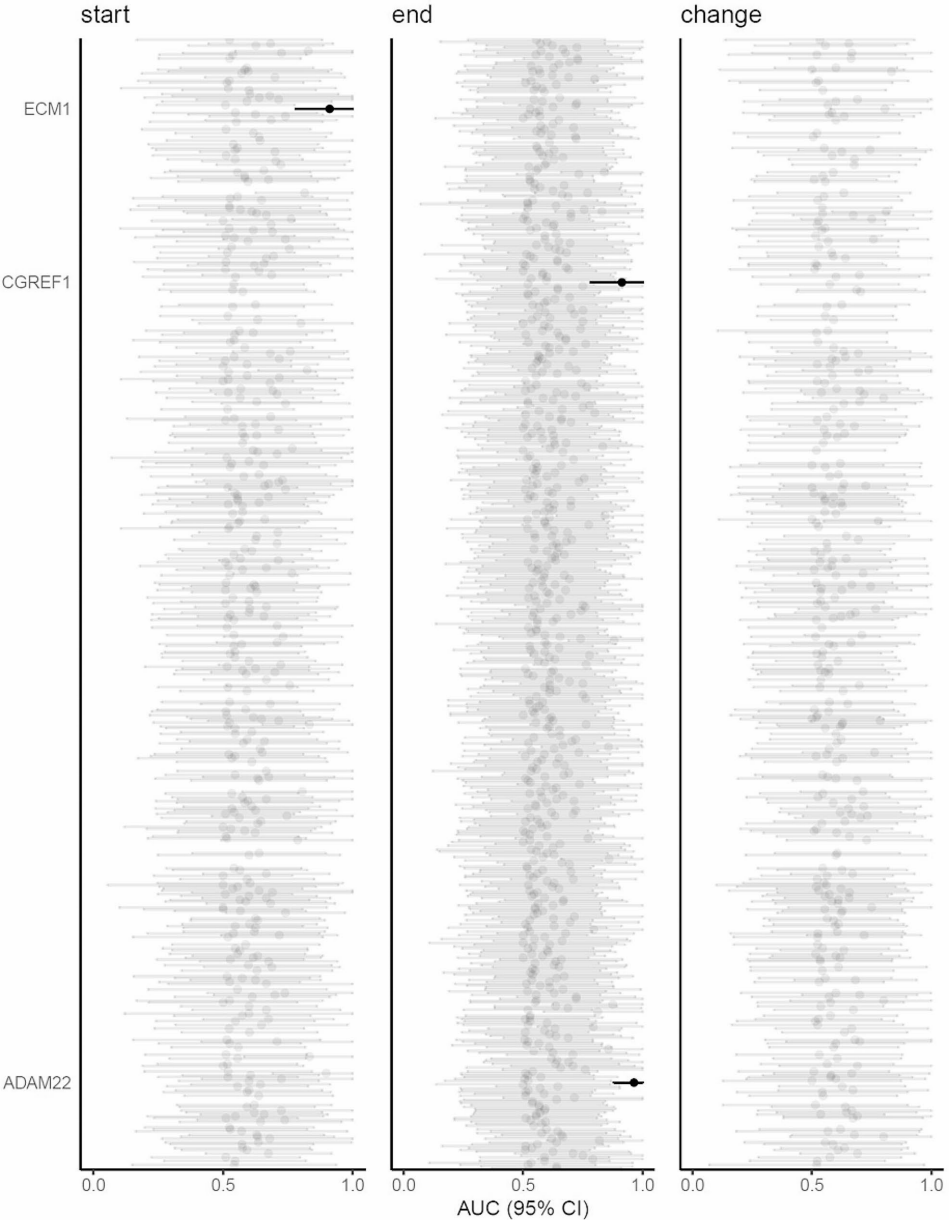
Potential proteomic predictors of functional outcome. The figure illustrates the calculated AUC and 95% CI for prediction of functional outcome assessed after six months in patients with SAH (*n* = 23), employing the proteins detected in the start samples, the end samples, and the calculated average change in protein abundance per day. Proteins that reached a lower confidence limit of ≥ 0.7 were accepted as possible predictors (black lines). AUC: area under the curve; CI: confidence interval

To determine whether certain proteins detected in the CSF from patients with SAH could serve as potential predictors of functional outcome assessed six months after ictus (favorable: mRS 0–2, *n* = 13; unfavorable: mRS 3–6, *n* = 10, see *Methods*), we employed ROC analysis and AUC values (Fig. 4; Table 6). For the start samples, one protein, extracellular matrix protein 1 (ECM1), was identified as a possible predictor of functional outcome. For the end samples, two proteins, ADAM metallopeptidase domain 22 (ADAM22) and cell growth regulator with EF hand domain protein 1 (CGREF1), were identified as possible predictors of functional outcome. No proteins were identified as possible predictors of functional outcome when considering the average change in protein abundance per day. The proteins that did not reach a lower confidence limit of ≥ 0.7 are listed in Supplementary file 2, Supplementary Table 6.

**Table 6:** **Possible predictors of functional outcome in SAH patients**

Protein Name	Uniprot ID	Time	mRS 0–2 Mean (SD) [*N*] in a.u.	mRS 3–6 Mean (SD) [*N*] in a.u.	AUC (95%CI)	Cut-off	Sens.	Spec.
ADAM22	F8WAD8	end	12.0 (1.0) [7]	10.2 (0.6) [8]	0.96 (0.88-1.00)	11.2	1.00	0.86
CGREF1	Q99674	end	13.5 (0.6) [9]	12.6 (0.5) [9]	0.91 (0.78-1.00)	13.4	1.00	0.67
ECM1	Q16610	start	14.7 (0.3) [10]	14.1 (0.4) [9]	0.91 (0.78-1.00)	14.6	1.00	0.70

mRS: Modified Rankin Scale; a.u.: arbitrary units; SD: standard deviation; N: number of patients; AUC: area under the curve; CI: confidence interval; sens: sensitivity; spec: specificity; ADAM22: ADAM metallopeptidase domain 22; CGREF1: Cell growth regulator with EF hand domain protein 1; ECM1: Extracellular matrix protein 1. Statistical significance was determined with Student’s t-test with Bonferroni correction

## Discussion

### Proteomic profiles

#### SAH vs. controls

We here revealed that the proteomic profile of CSF from patients with SAH differs from that of healthy control subjects as evident by an altered abundance of select CSF proteins. Substantiating the notion that SAH promotes neuroinflammation [45], CSF collected acutely after ictus from patients with SAH contained an increased abundance of SAA1 and S100B, proteins which are both linked to inflammation. While SAA1 serves as an acute-phase protein produced in large quantities in response to injury, infection, and inflammation [46], S100B, a Ca^2+^ binding protein predominantly derived from astrocytes, has been widely recognized as a putative biomarker of brain injury [47] and, only more recently, as a possible neuroinflammatory mediator [48]. Elevated CSF S100B levels have previously been reported in patients with SAH [49–52] suggesting that the protein may serve as a possible biomarker of SAH in general. However, whether some of the neuroinflammatory actions of S100B, such as its ability to promote microglial activation [48], contribute to the subsequent disease progression following SAH awaits determination. Among the proteins detected in lower abundance in the SAH patient CSF, when compared to that of control CSF, was APLP1 and APP, two proteins belonging to the family of amyloid precursor proteins [53]. Corroborating the present findings, a recent study revealed significantly decreased CSF levels of APLP1 and a similar trend for APP in CSF from patients with non-traumatic acute brain injury (the majority of which had SAH) when compared to that of healthy controls undergoing elective clipping of an unruptured aneurysm [54]. However, another proteomics study revealed significantly elevated APLP1 levels in SAH patient CSF when compared to control CSF [55]. The study employed lumbar CSF for their proteomics analysis, the composition of which differs from that of ventricular CSF [56], as employed here. Hence, methodological differences may explain some of these discrepancies.

### Time course

In the present study, we furthermore demonstrated that CSF collected acutely after ictus displays a distinct proteomic profile in comparison to CSF collected weeks later (on average 19 days, range: 5–30 days), thus substantiating that the CSF proteome in SAH patients is dynamic. Noticeably, the majority of proteins associated with an altered CSF abundance were elevated in the CSF samples collected acutely after ictus, while only a few proteins appeared elevated in the CSF samples collected weeks later. Among the proteins elevated acutely after ictus were different inflammatory mediators, components of the coagulation system, and different apolipoproteins. Proteins linked to the innate immune system were likewise elevated in the CSF samples collected just prior to EVD removal.

### Shunt dependency

Regardless of the time of CSF collection, we found no evidence of distinct proteomic CSF profiles when comparing patients with SAH who went on to develop shunt-dependent PHH and patients who could be successfully weaned off their EVD. This observation may possibly be explained by the statistical requirement of correction for multiple comparisons that accompanied our unbiased quantification of the CSF protein content, which may have led to the neglection of potentially relevant proteins capable of predicting shunt dependency and/or functional outcome. We therefore sought to identify potential biomarkers through use of an ROC analysis.

### Proteomic biomarkers

#### Shunt dependency

Evaluation of CSF biomarkers is gaining significance as potential clinical tools to guide treatment and predict the functional outcome of various neurological conditions. At present, it remains unresolved why some patients with SAH can be successfully weaned off their EVD, while other patients require permanent shunt insertion due to development of chronic PHH. Identification of predictive biomarkers for PHH is thus greatly needed. In the present study, we identified 16 unique proteins as possible predictors of shunt dependency, the majority of which were derived from the CSF samples collected weeks after ictus, just prior to EVD removal (end samples). Biomarkers of shunt dependency should ideally be detectable in the CSF as early as possible to minimize the risk of infection, which increases with the duration of the EVD [17–20]. However, the pathophysiological events culminating in development of PHH may manifest in the weeks following SAH and thus not be adequately reflected in the CSF samples collected acutely after ictus. Of all the proteins identified as possible biomarkers of shunt dependency, only one protein, S100B, was also found significantly more abundant in the SAH patient CSF when compared to the control CSF. Noticeably, employing the CSF samples collected acutely after ictus, S100B appeared less abundant in patients who required permanent CSF shunting compared to patients who could be successfully weaned from their EVD. The present observation thus suggests that lower CSF levels of S100B indicate a higher probability of permanent shunt requirement. However, this finding contradicts an earlier observation of higher CSF S100B levels in SAH patients requiring permanent shunt placement [29]. Hence, whether CSF S100B levels may assist clinical decision making with regards to permanent shunt placement after SAH requires further elucidation.

### Functional outcome

Employing the mRS for evaluation of functional outcome six months after ictus in patients with SAH, we identified three possible predictors, ADAM22, CGREF1, and ECM1, the levels of which were all elevated in patients with a favorable outcome (mRS 0–2) and lower in patients with an unfavorable outcome (mRS 3–6). To the best of our knowledge, none of these proteins have previously been identified as possible predictors of functional outcome after SAH and additional studies are thus required to elucidate their biomarker potential. Potential biomarkers of functional outcome previously identified in patients with SAH comprise, among others, inflammatory markers [30] and various proteins, including S100B [57–60], the protein here identified as a possible biomarker of shunt dependency. Hence, although the present study did not identify S100B as a possible biomarker of functional outcome, the protein appears intimately linked to the SAH pathophysiology.

### Strengths and limitations

To the best of our knowledge, previous proteomic studies of CSF from patients with SAH have generally relied on control CSF obtained from the lumbar compartment [55, 61], the protein composition of which differs from that of ventricular CSF [56]. The present study sought to limit this potentially confounding factor by comparing ventricular CSF collected from patients with SAH to cisternal CSF collected from healthy control subjects undergoing vascular clipping for an unruptured aneurysm. The different CSF sampling sites (ventricular versus cisternal) were dictated by ethical limitations but could potentially influence our results if the protein content and concentration of individual proteins differ between these CSF compartments. Although a recent study revealed no overall difference in cisternal and ventricular CSF protein content [62], further exploration is required to elucidate the extent to which the ventricular and cisternal CSF proteomes are comparable. Moreover, we cannot exclude that the surgical EVD insertion itself may have led to release of cellular debris or other proteins into the CSF, which could potentially have altered the protein content in the SAH patient samples and thus our present results, as others have previously demonstrated altered CSF protein levels upon EVD insertion [63]. Furthermore, we cannot exclude that alterations in the protein content may have occurred within the limited time frame from CSF collection to storage (maximum of 2 h), albeit the CSF samples were kept on ice to minimize such alterations. Although the total number of unique CSF proteins identified in the present study (1205 proteins) is similar to that identified in one of our recent studies (1251 proteins) [64], others have previously reported greater numbers (~ 2000–3000 CSF proteins) utilizing different separation methodologies and MS-based proteomics [65–68]. Hence, it is possible that some CSF proteins of potential relevance to the SAH pathophysiology were undetected. Lastly, the present study is limited by the relatively limited number of patient samples (*n* = 23) and control samples (*n* = 10), which may preclude detection of all potentially statistically relevant markers.

## Conclusions

Biomarkers capable of distinguishing SAH patients with only a temporary need for CSF diversion from those in need of permanent CSF shunting due to chronic PHH development are in great demand. Here we identified several possible biomarkers of shunt dependency and functional outcome in patients with SAH using an unbiased MS-based proteomics approach. The applicability of these biomarkers as potential clinical tools should be validated in larger patient cohorts, preferentially with additional targeted CSF analysis techniques to validate the promising biomarkers, such as benchtop immunoassays (e.g. ELISAs).

### Electronic supplementary material

Below is the link to the electronic supplementary material.

Supplementary Material 1: Figs. 1–2 can be found in Supplementary file 1

Supplementary Material 2: Tables 1–6 can be found in Supplementary file 2

Supplementary Material 3: Table 7 can be found in Supplementary file 3

## Data Availability

No datasets were generated or analysed during the current study.

## Abbreviations

AUC: Area under the curve
CSF: Cerebrospinal fluid
EVD: External ventricular drainage
ICP: Intracranial pressure
mRS: Modified Rankin Scale
MS: Mass spectrometry
PCA: Principal component analysis
PHH: Posthemorrhagic hydrocephalus
ROC: Receiver operating curve
SAH: Subarachnoid hemorrhage
