# Supplementary material for: Proteomic profile and predictive markers of outcome in patients with subarachnoid hemorrhage
Source: Clin Proteomics. 2024 Jul 23;21:51. doi: 10.1186/s12014-024-09493-6 (PMC11267790; doi:10.1186/s12014-024-09493-6)
Supplement: Supplementary file 1 — Supplementary Material 1: Figs. 1–2 can be found in Supplementary file 1 [file 12014_2024_9493_MOESM1_ESM.pptx]

## Slide 1
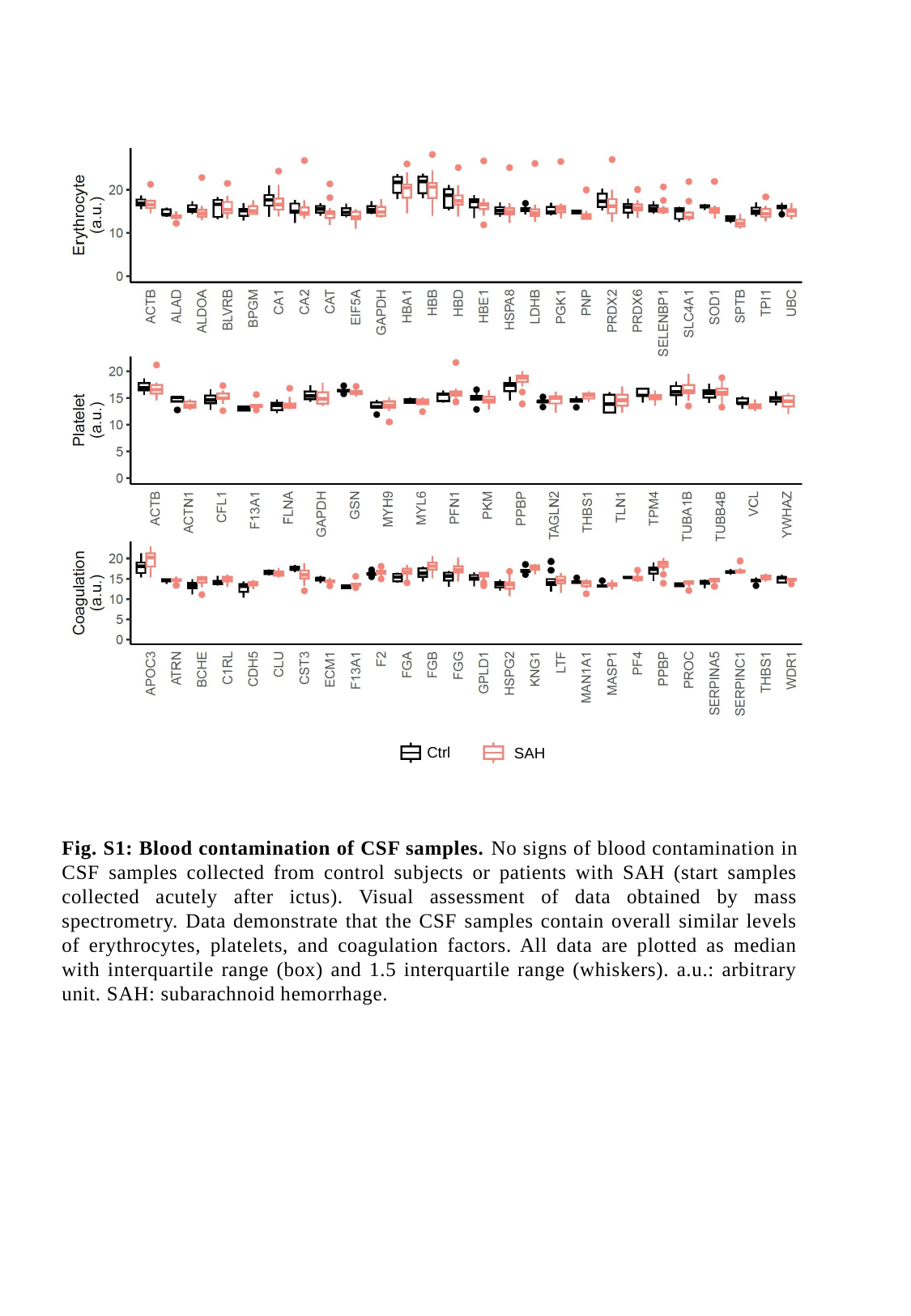

Ctrl
SAH
Fig. S1: Blood contamination of CSF samples. No signs of blood contamination in CSF samples collected from control subjects or patients with SAH (start samples collected acutely after ictus). Visual assessment of data obtained by mass spectrometry. Data demonstrate that the CSF samples contain overall similar levels of erythrocytes, platelets, and coagulation factors. All data are plotted as median with interquartile range (box) and 1.5 interquartile range (whiskers). a.u.: arbitrary unit. SAH: subarachnoid hemorrhage.

## Slide 2
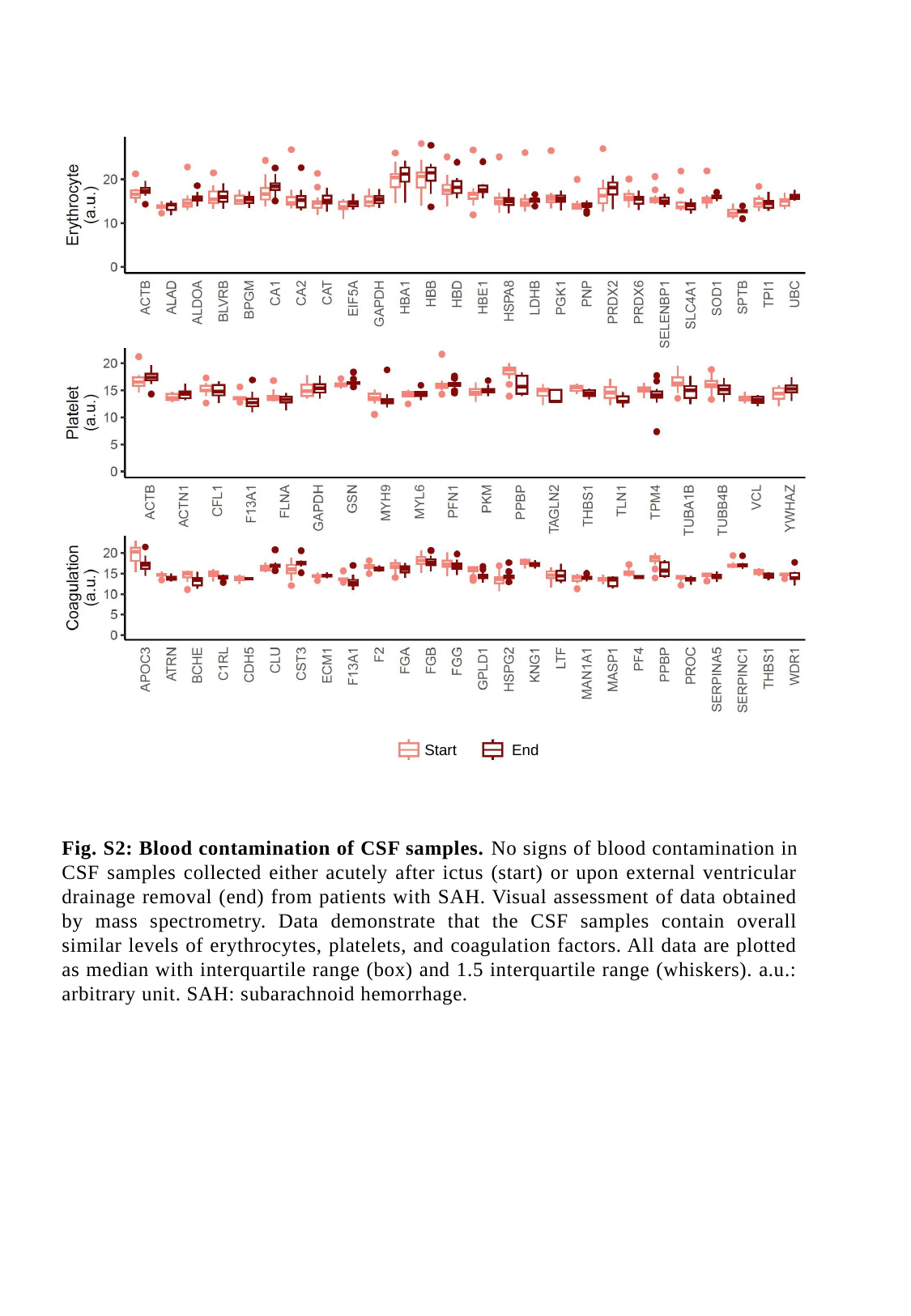

Start
End
Fig. S2: Blood contamination of CSF samples. No signs of blood contamination in CSF samples collected either acutely after ictus (start) or upon external ventricular drainage removal (end) from patients with SAH. Visual assessment of data obtained by mass spectrometry. Data demonstrate that the CSF samples contain overall similar levels of erythrocytes, platelets, and coagulation factors. All data are plotted as median with interquartile range (box) and 1.5 interquartile range (whiskers). a.u.: arbitrary unit. SAH: subarachnoid hemorrhage.
