# Supplementary material for: Proteomic profile and predictive markers of outcome in patients with subarachnoid hemorrhage
Source: Clin Proteomics. 2024 Jul 23;21:51. doi: 10.1186/s12014-024-09493-6 (PMC11267790; doi:10.1186/s12014-024-09493-6)
Supplement: Supplementary file 2 — Supplementary Material 2: Tables 1–6 can be found in Supplementary file 2 [file 12014_2024_9493_MOESM2_ESM.pdf]

Supplementary Tables 1-6

Supplementary Table 1: Other proteins in CSF from patients with SAH versus healthy control subjects.

**Protein Name**	**Uniprot ID**	**Ctrl** Mean (SD) [N]	**SAH start** Mean (SD) [N]	**log_2FC**	**P-value**	**Adj. P-value**
A1BG	P04217-2	17.1 (0.5) [10]	17.7 (0.7) [23]	0.60	0.012	1
A2M	P01023	17.2 (0.7) [10]	18.2 (0.9) [23]	0.96	0.003	1
ABHD14B	Q96IU4	14.1 (1.2) [8]	13.1 (1) [14]	-0.97	0.082	1
ACSBG2	Q5FVE4	20.3 (1.3) [10]	20.3 (0.7) [18]	-0.07	0.88	1
ACTA2	P62736	16.5 (1) [10]	16.4 (0.9) [19]	-0.13	0.723	1
ACTB	P60709	17.1 (1.1) [10]	16.7 (1.4) [23]	-0.44	0.334	1
ACTBL2	Q562R1	18.9 (1.1) [10]	18.3 (1.2) [22]	-0.59	0.191	1
ADIPOQ	Q15848	13 (1.1) [8]	14 (1.2) [14]	1.03	0.059	1
AFM	P43652	15.2 (0.3) [10]	15.8 (0.5) [22]	0.61	<0.001	0.062
AGRN	O00468-6	13.9 (0.9) [9]	13.4 (1.1) [10]	-0.52	0.272	1
AGT	P01019	16.9 (0.7) [10]	16.3 (0.6) [23]	-0.52	0.061	1
AHSG	P02765	17 (0.6) [10]	18.1 (1.7) [23]	1.06	0.015	1
ALB	P02768	19.1 (0.3) [10]	19.9 (0.6) [23]	0.78	<0.001	0.011
ALDOA	P04075	15.7 (1.1) [10]	14.9 (2) [22]	-0.72	0.196	1
ALDOC	P09972	14.4 (0.7) [10]	14.1 (1.1) [15]	-0.29	0.447	1
AMBP	P02760	16.1 (0.7) [10]	17 (0.8) [23]	0.86	0.005	1
ANXA5	P08758	15.7 (1.3) [9]	14.6 (1) [10]	-1.17	0.046	1
APCS	P02743	16 (1.9) [10]	17.6 (1.6) [22]	1.60	0.036	1
APLP2	Q06481	14.9 (0.8) [10]	14.2 (1.1) [14]	-0.70	0.085	1
APOA1	P02647	18.8 (1.1) [10]	20 (1.1) [23]	1.27	0.008	1
APOA2	P02652	18 (0.7) [10]	19 (0.9) [23]	0.95	0.003	1
APOA4	P06727	16.7 (0.8) [10]	17.4 (0.9) [22]	0.69	0.038	1
APOB	P04114	16.4 (1.9) [10]	18.8 (2.1) [23]	2.39	0.005	1
APOC1	K7ERI9	16.5 (1.3) [10]	18.1 (1.7) [22]	1.57	0.008	1
APOC3	B0YIW2	18.1 (2.1) [10]	19.7 (2.2) [22]	1.67	0.051	1
APOC4-APOC2	A0A024R0T9	15.9 (1.6) [10]	17.7 (2.1) [22]	1.74	0.017	1
APOD	C9JF17	16.5 (0.7) [10]	16.5 (0.8) [22]	-0.04	0.891	1
APOE	P02649	17.6 (0.7) [10]	16.5 (0.8) [23]	-1.07	0.001	0.229
APOH	P02749	15.8 (0.6) [10]	16.5 (0.7) [22]	0.63	0.016	1
APOL1	O14791	14.9 (1.9) [9]	16 (1.6) [22]	1.12	0.146	1
APOM	O95445	16.5 (1.3) [9]	17.7 (1.4) [22]	1.19	0.036	1
ARPC4-TTLL3	A0A0A6YYG9	16.3 (0.3) [10]	16.8 (0.5) [22]	0.50	0.003	1
ATP6AP1	Q15904	15.1 (0.8) [10]	13.7 (0.9) [17]	-1.36	0.001	0.294
ATRN	O75882	14.6 (0.4) [9]	14.7 (0.5) [20]	0.12	0.501	1

**Protein Name**	**Uniprot ID**	**Ctrl** Mean (SD) [N]	**SAH start** Mean (SD) [N]	**log$_2$FC**	**P-value**	**Adj. P-value**
AZGP1	P25311	16.4 (0.4) [10]	17 (0.6) [22]	0.56	0.003	1
B2M	P61769	16.4 (0.9) [10]	15.3 (2) [23]	-1.09	0.038	1
B4GAT1	O43505	16.5 (1) [10]	14.8 (1.3) [18]	-1.75	0.001	0.26
BCAN	Q96GW7	15.3 (0.9) [10]	14.7 (1.2) [17]	-0.62	0.144	1
BLVRB	P30043	15.8 (2.1) [9]	16.1 (2.2) [13]	0.28	0.768	1
BTD	P43251	15.8 (0.7) [10]	15.5 (0.4) [22]	-0.27	0.294	1
C1QA	P02745	14.8 (0.5) [9]	15.3 (0.5) [22]	0.55	0.014	1
C1QB	D6R934	15.4 (0.7) [10]	15.7 (0.6) [22]	0.33	0.196	1
C1QC	P02747	17.1 (0.6) [10]	17.6 (0.5) [22]	0.46	0.061	1
C1R	B4DPQ0	15.6 (0.5) [10]	16 (0.4) [22]	0.36	0.054	1
C1RL	Q9NZP8	14.3 (0.7) [9]	14.8 (0.9) [20]	0.59	0.073	1
C1S	P09871	16.3 (0.4) [10]	16.3 (0.4) [22]	0.06	0.702	1
C2	P06681	15 (0.5) [10]	15.5 (0.5) [22]	0.54	0.007	1
C2orf40	B8ZZE5	15.2 (2) [9]	14.8 (1.3) [16]	-0.44	0.563	1
C3	P01024	17.3 (0.4) [10]	17.9 (0.7) [23]	0.58	0.006	1
C4A	A0A0G2JPR0	15.6 (0.9) [10]	15.7 (0.8) [22]	0.09	0.783	1
C4B	P0C0L5	16.8 (0.4) [10]	16.9 (0.3) [23]	0.11	0.474	1
C4BPA	P04003	14.2 (1.8) [9]	16.1 (2) [22]	1.90	0.021	1
C5	P01031	14.7 (0.7) [10]	15.8 (0.7) [22]	1.03	0.001	0.482
C6	P13671	15.5 (0.6) [10]	16.4 (0.8) [22]	0.87	0.003	1
C7	P10643	15.8 (0.6) [10]	15.8 (0.6) [22]	0.08	0.733	1
C8A	P07357	15.3 (0.5) [10]	16 (0.7) [23]	0.71	0.005	1
C8B	F5GY80	14.1 (0.4) [10]	14.7 (0.6) [22]	0.65	0.001	0.265
C8G	P07360	14.5 (0.6) [6]	14.6 (0.5) [16]	0.15	0.583	1
C9	P02748	15.5 (0.4) [10]	16.1 (0.5) [22]	0.58	0.001	0.509
CA1	P00915	17.5 (2.1) [10]	17.1 (2.6) [19]	-0.44	0.623	1
CA2	P00918	15.4 (1.7) [9]	15.6 (2.9) [19]	0.23	0.79	1
CACNA2D1	P54289	14.6 (0.7) [10]	14.1 (1) [11]	-0.50	0.194	1
CADM4	Q8NFZ8	15.9 (0.8) [9]	15 (0.9) [10]	-0.90	0.033	1
CALR	P27797	13.5 (1) [10]	13.3 (0.9) [10]	-0.20	0.647	1
CARTPT	Q16568	13.7 (0.8) [9]	12.9 (0.9) [13]	-0.77	0.043	1
CASP14	P31944	14 (0.5) [7]	15 (1.1) [15]	1.00	0.009	1
CBR1	P16152	14.7 (0.9) [8]	15.1 (1.1) [17]	0.41	0.348	1
CD14	P08571	16 (1) [10]	15.1 (1.3) [22]	-0.86	0.053	1
CD44	H0YD13	16.5 (0.8) [10]	15.3 (1) [21]	-1.13	0.004	1
CDH13	P55290	15.3 (0.8) [10]	14.1 (0.8) [19]	-1.18	0.001	0.405
CDH2	P19022	15.7 (0.8) [10]	14.7 (1) [11]	-1.01	0.02	1
CFD	K7ERG9	15.7 (0.6) [10]	15.4 (0.8) [20]	-0.35	0.189	1
CFH	P08603	16 (0.5) [10]	16.9 (0.9) [23]	0.90	0.001	0.409
CFHR1	B1AKG0	15.4 (0.8) [10]	16 (1) [22]	0.62	0.079	1
CFI	E7ETH0	14.7 (0.2) [10]	15.4 (0.5) [22]	0.64	<0.001	0.008

**Protein Name**	**Uniprot ID**	**Ctrl** Mean (SD) [N]	**SAH start** Mean (SD) [N]	**log₂FC**	**P-value**	**Adj. P-value**
CFL1	E9PK25	14.8 (1.2) [10]	15.2 (1) [21]	0.42	0.342	1
CHI3L1	P36222	15.4 (1.4) [10]	15.4 (1.9) [21]	-0.02	0.978	1
CHI3L2	Q15782	12.6 (1.9) [5]	14.2 (1.6) [14]	1.67	0.124	1
CKB	P12277	15.4 (1.3) [9]	16.6 (1.5) [19]	1.16	0.052	1
CLCNKB	A0A087X136	16.2 (0.9) [10]	15.2 (1) [10]	-0.99	0.036	1
CLEC3B	E9PHK0	16.4 (0.4) [10]	15.8 (0.5) [22]	-0.65	0.001	0.587
CLU	P10909	16.6 (0.5) [10]	16.4 (0.6) [23]	-0.30	0.157	1
CNTN1	Q12860	15.5 (0.8) [10]	14 (1) [17]	-1.48	<0.001	0.146
CNTN2	A0A1W2PQ11	15.3 (1.2) [10]	14.2 (1.1) [17]	-1.18	0.02	1
COL18A1	P39060	14.8 (0.7) [9]	14.2 (1.2) [12]	-0.60	0.156	1
COL6A1	A0A087X0S5	14.8 (0.8) [10]	13.6 (1.2) [16]	-1.16	0.005	1
COL6A3	P12111	13.9 (0.5) [8]	13.9 (0.7) [12]	0.03	0.907	1
CP	P00450	16.3 (0.4) [10]	16.8 (0.6) [23]	0.53	0.009	1
CPB2	A0A087WSY5	15.4 (0.4) [10]	15.7 (0.7) [22]	0.28	0.17	1
CPE	P16870	16.5 (1.1) [10]	15 (1.2) [21]	-1.48	0.003	1
CPN1	P15169	14.6 (0.7) [5]	15.4 (0.7) [19]	0.77	0.075	1
CPN2	P22792	15.3 (1.2) [10]	16.7 (1.1) [22]	1.37	0.008	1
CPQ	Q9Y646	15.3 (1) [10]	14.2 (1) [13]	-1.06	0.023	1
CPVL	Q9H3G5	14.7 (1.1) [8]	14.2 (0.7) [14]	-0.55	0.247	1
CRP	P02741	14 (1) [5]	14.2 (1.2) [18]	0.18	0.742	1
CRTAC1	A0A0C4DFP6	15.3 (0.6) [10]	14.3 (1) [17]	-0.98	0.004	1
CSF1	P09603	14.5 (0.7) [10]	14.1 (1) [14]	-0.36	0.323	1
CST3	P01034	17.6 (0.6) [10]	15.9 (1.6) [23]	-1.61	<0.001	0.088
CTSA	P10619	13.6 (1) [6]	13.3 (0.5) [10]	-0.26	0.571	1
CTSB	P07858	13.6 (1) [9]	14.6 (1.3) [13]	0.96	0.067	1
CTSD	A0A1B0GV23	16.1 (1.5) [10]	15.5 (1.1) [21]	-0.64	0.251	1
CTSH	A0A087X0D5	14.6 (1.2) [9]	13.9 (0.9) [11]	-0.74	0.147	1
CTSL	P07711	15.3 (0.9) [8]	15 (0.9) [13]	-0.28	0.509	1
CTSZ	Q9UBR2	15.5 (1.1) [8]	15.1 (0.7) [10]	-0.40	0.384	1
DAG1	Q14118	15.3 (0.5) [10]	14.3 (1) [14]	-1.03	0.005	1
DBI	A0A0A0MTI5	14.7 (0.6) [10]	14.6 (0.5) [17]	-0.06	0.79	1
DPYSL2	A0A1C7CYX9	14.9 (0.8) [5]	15.5 (1.3) [15]	0.64	0.225	1
ECM1	Q16610	15 (0.5) [10]	14.4 (0.4) [20]	-0.56	0.007	1
EFEMP1	A0A0U1RQV3	16 (1.3) [10]	15.6 (1.3) [17]	-0.43	0.418	1
EIF5A	I3L397	15 (1.1) [8]	13.9 (1.1) [17]	-1.09	0.04	1
ENDOD1	O94919	15.1 (1) [10]	14.5 (0.6) [14]	-0.56	0.153	1
ENO2	P09104	14.7 (0.6) [9]	15.2 (2.5) [18]	0.51	0.416	1
ENPP2	E7EUF1	16.4 (1.6) [10]	15.7 (1.2) [22]	-0.71	0.229	1
EPHA4	E9PG71	15.2 (0.7) [9]	13.8 (1.2) [12]	-1.44	0.002	0.946
ERN1	O75460	16.8 (1.7) [8]	17.9 (2) [21]	1.08	0.168	1
F10	P00742	14.3 (0.6) [9]	15.5 (0.7) [17]	1.12	<0.001	0.15

**Protein Name**	**Uniprot ID**	**Ctrl** Mean (SD) [N]	**SAH start** Mean (SD) [N]	**log$_2$FC**	**P-value**	**Adj. P-value**
F12	P00748	16.3 (0.9) [10]	17.1 (0.9) [22]	0.81	0.026	1
F13B	P05160	13.5 (0.5) [5]	14 (1.9) [17]	0.44	0.404	1
F2	P00734	16.3 (0.5) [10]	16.6 (0.7) [22]	0.30	0.169	1
F5	A0A0A0MRJ7	14.9 (1.2) [10]	14.9 (0.4) [22]	-0.01	0.987	1
F9	P00740	13.5 (0.5) [10]	14.2 (0.6) [21]	0.67	0.002	0.937
FAM3C	Q92520	15.3 (0.9) [10]	13.9 (0.9) [16]	-1.31	0.002	0.729
FBLN1	P23142	16.2 (0.8) [10]	15.4 (1) [22]	-0.84	0.015	1
FCGR3A	A0A1W2PQB1	14.3 (1.1) [10]	14.2 (1.2) [17]	-0.09	0.84	1
FCN3	O75636	13.8 (1.4) [6]	15.4 (1.6) [18]	1.70	0.03	1
FETUB	Q9UGM5	14.6 (0.8) [9]	15.5 (1.2) [20]	0.94	0.022	1
FGA	P02671	15.3 (1) [10]	16.7 (1.1) [23]	1.41	0.002	0.74
FGB	P02675	16.5 (1.2) [10]	18.1 (1.5) [23]	1.63	0.004	1
FGG	P02679	15.5 (1.3) [10]	17.3 (1.4) [23]	1.77	0.002	1
FLNA	P21333	13.5 (0.9) [8]	13.9 (1) [15]	0.37	0.393	1
FN1	P02751	16.1 (0.4) [10]	16.6 (0.7) [23]	0.54	0.007	1
FSTL1	Q12841	14 (1) [8]	13.2 (1.4) [17]	-0.73	0.153	1
FUCA1	P04066	14.1 (0.8) [8]	13.6 (1.1) [10]	-0.51	0.269	1
FUCA2	Q9BTY2	14.1 (0.9) [10]	13.8 (1.1) [15]	-0.33	0.436	1
GAPDH	P04406	15.6 (1.1) [10]	15.1 (1.2) [21]	-0.50	0.261	1
GC	P02774	16.7 (0.2) [10]	17.2 (0.7) [23]	0.53	0.002	0.662
GDI2	P50395	14.2 (0.6) [9]	14.3 (1) [15]	0.13	0.695	1
GGH	Q92820	14.8 (0.8) [10]	14.3 (0.4) [17]	-0.44	0.12	1
GM2A	P17900	15.9 (0.9) [10]	14.7 (1.2) [19]	-1.23	0.006	1
GOT1	P17174	15.3 (0.8) [10]	14.6 (0.7) [11]	-0.71	0.04	1
GPI	A0A0A0MTS2	13.6 (0.6) [8]	13.7 (0.9) [10]	0.14	0.693	1
GPLD1	P80108	15.2 (1.2) [7]	15.8 (1) [20]	0.60	0.251	1
GPX3	A0A087X1J7	16.2 (0.9) [10]	16 (0.6) [22]	-0.16	0.615	1
GSN	P06396	16.4 (0.4) [10]	16.1 (0.4) [23]	-0.33	0.066	1
GSTP1	P09211	16.1 (0.9) [10]	15.2 (1.1) [18]	-0.82	0.05	1
HBA1	P69905	21.1 (2.1) [10]	20 (2.6) [23]	-1.17	0.189	1
HBB	P68871	21.2 (2.1) [10]	20.1 (3) [23]	-1.15	0.223	1
HBD	P02042	18.2 (2.3) [10]	17.9 (2.3) [21]	-0.34	0.702	1
HBG2	P69892	16.3 (1.5) [9]	16.3 (2.4) [17]	0.00	0.999	1
HEXB	P07686	14.4 (1.3) [8]	13.5 (0.8) [12]	-0.98	0.081	1
HGFAC	D6RAR4	14.4 (0.6) [10]	15.1 (0.6) [20]	0.75	0.004	1
HP	P00738	17.3 (1.7) [10]	18.9 (2.1) [23]	1.62	0.031	1
HPR	P00739	16.1 (1.4) [10]	16.8 (1.7) [22]	0.74	0.198	1
HPX	P02790	17.9 (0.7) [10]	18.4 (0.5) [23]	0.51	0.05	1
HRG	P04196	16 (0.7) [10]	16.9 (0.7) [23]	0.87	0.006	1
HSP90AA1	P07900	14.4 (0.7) [8]	15 (1) [18]	0.58	0.104	1
HSPA5	P11021	14.5 (0.4) [9]	13.9 (0.8) [12]	-0.51	0.087	1

**Protein Name**	**Uniprot ID**	**Ctrl** Mean (SD) [N]	**SAH start** Mean (SD) [N]	**log$_2$FC**	**P-value**	**Adj. P-value**
HSPA8	P11142	15.3 (1.1) [10]	15.4 (2.5) [20]	0.16	0.814	1
HSPG2	P98160	13.6 (0.9) [10]	13.5 (1.6) [18]	-0.07	0.885	1
HTRA1	Q92743	14.1 (1) [9]	13.4 (0.9) [15]	-0.71	0.109	1
ICOSLG	K4DIA0	15.4 (1.1) [10]	13.9 (1.2) [18]	-1.50	0.003	1
IGFALS	P35858	15.2 (0.7) [10]	16.1 (0.8) [22]	0.83	0.006	1
IGFBP6	P24592	17.1 (0.8) [10]	16 (1.6) [20]	-1.09	0.018	1
IGFBP7	Q16270	15.9 (2) [10]	15.8 (1.5) [23]	-0.06	0.936	1
IGHA1	A0A286YEY1	17.6 (1.1) [10]	19.2 (1.1) [23]	1.65	0.001	0.456
IGHA2	A0A286YEY5	17.7 (0.9) [10]	19.3 (1.1) [23]	1.65	<0.001	0.08
IGHD	A0A0A0MS09	15.8 (1.1) [8]	16.2 (1.4) [20]	0.43	0.395	1
IGHG1	P01857	19.5 (0.7) [10]	20.4 (0.6) [23]	0.93	0.002	0.644
IGHG2	P01859	19.4 (0.7) [10]	20.3 (0.6) [23]	0.85	0.004	1
IGHG3	P01860	16.6 (1.3) [10]	17.8 (1) [22]	1.15	0.024	1
IGHG4	A0A286YFJ8	15.9 (0.7) [10]	16.7 (0.9) [23]	0.82	0.013	1
IGHM	A0A1B0GUU9	17.4 (2.3) [10]	19.3 (1.8) [22]	1.90	0.036	1
IGHV1-18	A0A0C4DH31	14.2 (0.7) [6]	14.8 (1) [15]	0.64	0.108	1
IGHV1OR15-1	A0A075B7D0	17 (1.5) [9]	17.4 (1.5) [21]	0.36	0.558	1
IGHV2-26	A0A0B4J1V2	13.1 (0.6) [8]	14.1 (1.1) [19]	0.99	0.007	1
IGHV2-5	P01817	12.7 (0.7) [5]	13.2 (1.1) [13]	0.48	0.293	1
IGHV3-15	A0A0B4J1V0	16 (0.4) [10]	16.2 (0.6) [22]	0.18	0.336	1
IGHV3-30	P01768	16.9 (0.2) [10]	17.4 (0.6) [22]	0.55	<0.001	0.175
IGHV3-38	A0A0C4DH36	15.4 (0.7) [10]	15.6 (0.6) [22]	0.19	0.456	1
IGHV3-49	A0A0A0MS15	16.9 (0.8) [10]	17.9 (0.8) [22]	1.04	0.004	1
IGHV3-64D	A0A0J9YX35	15.9 (0.4) [10]	15.9 (0.7) [22]	0.07	0.729	1
IGHV3-7	P01780	17 (0.2) [10]	17.8 (0.6) [22]	0.77	<0.001	0.012
IGHV3-72	A0A0B4J1Y9	16.3 (0.6) [10]	17.2 (0.8) [22]	0.87	0.001	0.589
IGHV3OR16-9	A0A0B4J2B5	19.8 (0.8) [10]	20.4 (0.6) [23]	0.67	0.028	1
IGHV4-34	P06331	15.1 (0.6) [10]	15.9 (0.9) [22]	0.76	0.012	1
IGHV5-51	A0A0C4DH38	15.8 (0.4) [10]	16.4 (0.8) [22]	0.62	0.008	1
IGKC	P01834	20.1 (0.8) [10]	21 (1.3) [23]	0.99	0.014	1
IGKV1-12	A0A0C4DH73	16.9 (1) [10]	17.8 (1.1) [22]	0.92	0.028	1
IGKV1-16	P04430	14.4 (0.6) [7]	14.7 (0.8) [17]	0.34	0.254	1
IGKV1-17	P01599	15.6 (0.6) [10]	16.5 (0.8) [22]	0.89	0.002	0.732
IGKV1-5	P01602	16.4 (0.6) [10]	16.8 (0.7) [22]	0.41	0.083	1
IGKV1-8	A0A0C4DH67	16.3 (1.2) [10]	17.3 (1.1) [22]	1.02	0.034	1
IGKV1D-33	P01593	17.7 (1.1) [10]	18.3 (1) [22]	0.53	0.216	1
IGKV1D-37	A0A075B6S9	16.7 (1.1) [8]	17.9 (1.1) [18]	1.15	0.026	1
IGKV2-28	A0A075B6P5	16 (0.7) [10]	16.8 (0.9) [21]	0.83	0.009	1
IGKV2-29	A2NJV5	16.3 (0.7) [10]	17.2 (1) [22]	0.93	0.005	1
IGKV2-40	A0A087WW87	13.5 (0.6) [8]	14.2 (0.9) [11]	0.74	0.05	1

Protein Name	Uniprot ID	Ctrl Mean (SD) [N]	SAH start Mean (SD) [N]	log_2FC	P-value	Adj. P-value
IGKV2D-24	A0A075B6R9	16.9 (1.2) [10]	17.5 (1.1) [22]	0.53	0.257	1
IGKV2D-29	A0A075B6S2	13.9 (0.6) [6]	14.5 (0.7) [12]	0.60	0.096	1
IGKV3-15	P01624	17.4 (0.7) [10]	17.7 (0.6) [22]	0.34	0.176	1
IGKV3-20	P01619	18.3 (0.8) [10]	19.2 (0.9) [23]	0.92	0.008	1
IGKV3-7	A0A075B6H7	17.3 (1.4) [9]	18.6 (1.5) [20]	1.28	0.041	1
IGKV3D-11	A0A0A0MRZ8	17.6 (0.5) [10]	18.1 (0.5) [22]	0.48	0.025	1
IGKV3D-15	A0A087WSY6	17.5 (0.9) [9]	18.2 (0.9) [22]	0.73	0.054	1
IGKV3D-20	A0A0C4DH25	18.4 (0.5) [10]	19.4 (1.2) [22]	1.03	0.001	0.499
IGKV4-1	P06312	17.4 (0.5) [10]	17.9 (0.7) [22]	0.51	0.027	1
IGLC3	P0DOY3	19.9 (0.5) [10]	20.8 (0.7) [22]	0.90	<0.001	0.112
IGLL1	P15814	15.6 (0.9) [6]	16.7 (0.9) [17]	1.18	0.025	1
IGLL5	A0A0B4J231	18 (0.8) [10]	18.8 (0.8) [23]	0.80	0.014	1
IGLV1-36	A0A0B4J1U3	15.5 (1) [5]	16.5 (0.8) [10]	0.94	0.114	1
IGLV1-47	P01700	16.2 (0.4) [10]	17.1 (0.8) [22]	0.84	<0.001	0.194
IGLV1-51	P01701	16 (0.9) [8]	16.7 (0.8) [21]	0.75	0.057	1
IGLV3-10	A0A075B6K4	15.5 (0.7) [8]	16.3 (0.9) [16]	0.82	0.025	1
IGLV3-19	P01714	14.3 (0.4) [9]	15.1 (0.5) [21]	0.75	<0.001	0.042
IGLV3-21	P80748	15.2 (1) [9]	16.1 (1.1) [20]	0.93	0.042	1
IGLV3-25	P01717	14.3 (1) [10]	15.8 (1) [21]	1.45	0.002	0.67
IGLV3-9	A0A075B6K5	15.2 (1.4) [10]	16.5 (1.3) [22]	1.25	0.027	1
IGLV6-57	P01721	14.6 (0.8) [8]	15.4 (0.8) [22]	0.84	0.021	1
IGLV7-46	A0A075B6I9	15.5 (0.5) [7]	16.2 (0.7) [22]	0.71	0.007	1
IGLV8-61	A0A075B6I0	15.8 (0.8) [9]	16.5 (0.9) [16]	0.75	0.05	1
IGSF8	Q969P0	15.4 (1.2) [10]	14.5 (1.1) [14]	-0.89	0.078	1
ISLR	O14498	14.9 (0.7) [9]	13.9 (1.3) [16]	-1.01	0.02	1
ITIH1	P19827	16 (0.9) [10]	17.4 (1.6) [23]	1.34	0.005	1
ITIH2	P19823	16 (1) [10]	17.1 (0.9) [23]	1.18	0.005	1
ITIH3	Q06033	14.8 (0.9) [9]	15.6 (0.8) [22]	0.82	0.039	1
ITIH4	Q14624	16.6 (0.8) [10]	17.5 (0.7) [23]	0.94	0.004	1
ITPR2	Q14571	18.1 (1.1) [10]	18.8 (0.9) [22]	0.76	0.071	1
JCHAIN	D6RD17	16.6 (1.8) [9]	18.3 (1.5) [22]	1.62	0.031	1
KLK6	Q92876	16.4 (1.4) [10]	15 (1.2) [21]	-1.43	0.016	1
KLKB1	H0YAC1	14.6 (1.1) [10]	15.7 (1) [22]	1.10	0.012	1
KNG1	P01042	17.1 (0.9) [10]	17.8 (0.8) [22]	0.64	0.058	1
KRT1	P04264	14.9 (1.5) [8]	15.4 (3.5) [16]	0.53	0.603	1
LAMP2	P13473	15.5 (1.1) [10]	13.9 (1.2) [20]	-1.65	0.001	0.507
LBP	P18428	14 (0.5) [7]	14.6 (0.7) [17]	0.51	0.054	1
LCAT	P04180	14.9 (0.4) [8]	14.8 (0.6) [21]	-0.08	0.702	1
LCP1	P13796	14.6 (0.7) [7]	14.2 (0.7) [15]	-0.46	0.17	1
LDHA	P00338	15 (1.4) [10]	14.3 (1.2) [15]	-0.68	0.212	1
LDHB	P07195	15.4 (0.8) [10]	15.2 (2.7) [21]	-0.27	0.675	1

**Protein Name**	**Uniprot ID**	**Ctrl** Mean (SD) [N]	**SAH start** Mean (SD) [N]	**log$_2$FC**	**P-value**	**Adj. P-value**
LGALS1	P09382	14.8 (0.6) [10]	14.2 (0.6) [12]	-0.55	0.055	1
LGALS3BP	Q08380	16.3 (1.1) [10]	15.5 (0.7) [22]	-0.85	0.05	1
LIAS	A0A1W2PNQ5	14.5 (1.3) [7]	15.7 (1.2) [18]	1.22	0.055	1
LMAN2	D6RBV2	14.9 (0.6) [9]	14 (1.2) [12]	-0.88	0.042	1
LRG1	P02750	16.6 (0.3) [10]	16.9 (0.4) [22]	0.30	0.015	1
LSAMP	H3BLU2	15.1 (0.9) [10]	13.7 (1.1) [14]	-1.46	0.002	0.721
LTF	E7EQB2	14.6 (2.4) [8]	14.5 (1.4) [14]	-0.15	0.873	1
LUM	P51884	15.8 (0.6) [10]	16 (0.5) [22]	0.18	0.39	1
LYVE1	Q9Y5Y7	14.8 (0.8) [10]	14.1 (1.5) [20]	-0.69	0.109	1
LYZ	A0A0B4J259	16.3 (1.2) [10]	15.7 (1.3) [22]	-0.62	0.211	1
MAN1A1	P33908	14.3 (0.4) [10]	13.8 (1) [12]	-0.53	0.131	1
MASP1	P48740	13.4 (0.5) [10]	13.6 (0.6) [14]	0.23	0.319	1
MDH1	P40925	15.9 (0.7) [10]	15.5 (0.9) [13]	-0.45	0.183	1
MMP2	P08253	14.8 (0.8) [10]	14.1 (1.4) [13]	-0.66	0.18	1
MST1	G3XAK1	13.8 (0.7) [6]	14.1 (1) [11]	0.30	0.484	1
NBL1	A0A087WTY6	17.7 (0.7) [10]	17.3 (1.3) [11]	-0.38	0.408	1
NEGR1	Q7Z3B1	15.6 (1) [10]	13.6 (1.4) [14]	-2.01	0.001	0.228
NEO1	Q92859	14.6 (1) [10]	13.8 (1.1) [12]	-0.80	0.089	1
NPC2	E7EMS2	16 (1.3) [10]	15.3 (1.1) [16]	-0.69	0.175	1
NPTX1	Q15818	15.8 (0.9) [10]	13.6 (1.7) [16]	-2.10	<0.001	0.151
NPTXR	A0A1X7SBT7	15.4 (1.1) [10]	14.1 (1) [10]	-1.31	0.012	1
NRXN2	G5E9G7	14.7 (1) [10]	15.1 (1.1) [10]	0.39	0.421	1
NRXN3	A0A0U1RQC5	14.4 (1) [10]	13 (1) [11]	-1.38	0.004	1
NTM	Q9P121-4	14.9 (0.8) [10]	13.7 (1) [11]	-1.18	0.007	1
NUCB1	Q02818	13.5 (1.1) [10]	13.3 (1.1) [16]	-0.27	0.55	1
OGN	P20774	15.8 (1) [10]	14.3 (1.7) [19]	-1.54	0.006	1
OMG	P23515	15.4 (1.1) [10]	14.7 (1.1) [11]	-0.71	0.159	1
ORM1	P02763	18.8 (0.7) [10]	19.5 (0.5) [23]	0.64	0.025	1
ORM2	P19652	17.3 (0.5) [10]	17.8 (0.5) [23]	0.47	0.018	1
PAM	P19021	15.2 (0.6) [10]	13.8 (0.9) [13]	-1.37	<0.001	0.1
PCOLCE	Q15113	15.8 (0.9) [10]	15.1 (1.2) [16]	-0.70	0.102	1
PCSK9	Q8NBP7	12 (1) [5]	12.2 (0.6) [10]	0.15	0.771	1
PEBP1	P30086	16.2 (0.7) [10]	15.7 (1) [19]	-0.48	0.161	1
PEBP4	Q96S96	15.6 (0.9) [9]	14.7 (1.1) [12]	-0.91	0.048	1
PENK	P01210	14.9 (0.9) [10]	14.2 (0.9) [11]	-0.76	0.058	1
PEPD	P12955	12.8 (0.9) [9]	12.6 (0.6) [17]	-0.17	0.63	1
PFN1	P07737	15.3 (0.8) [9]	16.1 (1.4) [20]	0.76	0.084	1
PGK1	P00558	15.2 (1) [10]	15.9 (2.9) [17]	0.66	0.4	1
PGLYRP2	Q96PD5	14.8 (0.7) [10]	15.6 (0.8) [23]	0.81	0.006	1
PLG	P00747	16.4 (0.8) [10]	17.3 (0.9) [23]	0.89	0.009	1
PLTP	P55058	15.4 (0.9) [10]	15 (1.2) [22]	-0.41	0.276	1

**Protein Name**	**Uniprot ID**	**Ctrl** Mean (SD) [N]	**SAH start** Mean (SD) [N]	**log₂FC**	**P-value**	**Adj. P-value**
PLXDC2	Q6UX71	14.7 (0.9) [10]	13.5 (1.2) [18]	-1.28	0.003	1
PMFBP1	G3V1Q7	16.2 (0.5) [7]	15.8 (0.8) [11]	-0.36	0.242	1
PON1	P27169	16.2 (0.9) [9]	17.4 (1.2) [22]	1.28	0.005	1
PPBP	P02775	17 (1.5) [7]	18.4 (1.4) [20]	1.39	0.058	1
PPIA	P62937	16.3 (0.9) [10]	15.7 (1.2) [22]	-0.60	0.147	1
PPIB	P23284	15.3 (1.1) [10]	14.4 (0.9) [16]	-0.89	0.038	1
PRDX1	Q06830	17.1 (1.5) [10]	16.5 (2.3) [22]	-0.52	0.458	1
PRDX2	P32119	17.6 (1.9) [10]	16.7 (2.9) [23]	-0.93	0.293	1
PRDX6	P30041	15.8 (1.5) [10]	15.9 (1.5) [20]	0.12	0.839	1
PRG4	A0A0U1RR20	12.9 (1) [7]	13.6 (0.8) [20]	0.65	0.152	1
PROC	E7END6	13.6 (0.4) [9]	13.9 (0.6) [20]	0.38	0.052	1
PROCR	Q9UNN8	14.5 (0.7) [10]	14 (0.7) [19]	-0.49	0.099	1
PROS1	P07225	15.3 (0.5) [10]	15.9 (0.5) [22]	0.55	0.012	1
PROZ	P22891	14 (1) [8]	15.1 (0.9) [17]	1.04	0.025	1
PSAT1	Q9Y617	13.5 (1) [6]	14.3 (0.7) [11]	0.77	0.125	1
PTPRG	P23470	15 (0.8) [10]	14 (0.9) [13]	-0.98	0.011	1
PTPRZ1	P23471	14.7 (1) [10]	13.8 (1) [14]	-0.94	0.039	1
QSOX1	O00391	14.1 (0.6) [10]	14 (0.4) [19]	-0.08	0.723	1
RARRES2	Q99969	15 (1.1) [10]	14.3 (1) [14]	-0.63	0.165	1
RBP4	P02753	15.7 (0.6) [10]	16.4 (0.9) [23]	0.66	0.016	1
S100A8	P05109	15.9 (1.9) [8]	15.9 (2) [20]	-0.03	0.967	1
SAA4	P35542	15.6 (0.9) [10]	16.7 (1.1) [22]	1.10	0.006	1
SCG2	P13521	14.3 (0.8) [10]	12.8 (0.7) [18]	-1.46	<0.001	0.065
SCG5	P05408-2	16 (0.7) [10]	14.6 (1.1) [16]	-1.36	0.001	0.321
SCRG1	O75711	16.8 (0.9) [9]	15.9 (1.5) [12]	-0.89	0.103	1
SELENOP	A0A182DWH7	15.5 (0.4) [9]	15.8 (0.4) [17]	0.33	0.052	1
SELL	P14151	15.4 (0.7) [10]	16 (0.6) [19]	0.54	0.058	1
SERPINA1	P01009	18.4 (0.3) [10]	18.7 (0.6) [23]	0.38	0.032	1
SERPINA10	G3V2W1	12.9 (1.5) [5]	13.8 (1) [18]	0.87	0.273	1
SERPINA3	P01011	17 (0.6) [10]	17.5 (0.7) [23]	0.52	0.049	1
SERPINA4	P29622	15 (0.5) [10]	15.7 (0.5) [22]	0.69	0.002	0.815
SERPINA5	P05154	14.1 (0.6) [10]	14.6 (0.5) [21]	0.51	0.042	1
SERPINA6	P08185	15.3 (0.2) [10]	16 (0.7) [23]	0.66	<0.001	0.139
SERPINA7	P05543	14.7 (0.3) [10]	14.9 (0.5) [22]	0.22	0.15	1
SERPINC1	P01008	16.7 (0.4) [10]	17 (0.6) [23]	0.28	0.123	1
SERPIND1	P05546	15.5 (0.3) [10]	16.2 (0.6) [23]	0.67	<0.001	0.057
SERPINF1	P36955	17.4 (1.2) [10]	16.6 (1) [23]	-0.78	0.1	1
SERPINF2	P08697	16.3 (0.4) [10]	16.8 (0.6) [23]	0.43	0.023	1
SERPING1	P05155	16.5 (0.2) [10]	17 (0.3) [23]	0.52	<0.001	0.018
SERPINI1	Q99574	14.7 (1.2) [9]	13.3 (1) [11]	-1.41	0.011	1
SHBG	I3L145	14.7 (1) [7]	14.8 (1.2) [19]	0.12	0.801	1

Protein Name	Uniprot ID	Ctrl Mean (SD) [N]	SAH start Mean (SD) [N]	log_2FC	P-value	Adj. P-value
SIRPA	P78324	14.8 (1) [10]	13.8 (0.7) [11]	-1.00	0.015	1
SKP1	E5RJR5	14.1 (0.7) [9]	14.1 (0.7) [16]	0.04	0.897	1
SOD1	P00441	16.1 (0.4) [10]	15.5 (1.8) [18]	-0.66	0.151	1
SOD3	P08294	15.3 (1.4) [10]	14.6 (1.1) [22]	-0.68	0.191	1
SPARC	P09486	15.1 (1.8) [10]	14.7 (0.8) [22]	-0.39	0.529	1
SPP1	P10451	15.6 (0.6) [10]	14.5 (2.3) [23]	-1.09	0.045	1
SYNE3	G3V533	15 (0.7) [7]	15.7 (0.9) [15]	0.70	0.062	1
SYT2	Q8N9I0	17.3 (1.1) [9]	18.4 (0.9) [12]	1.11	0.025	1
TAGLN2	P37802	14.3 (0.6) [5]	14.7 (1) [16]	0.34	0.397	1
TALDO1	P37837	15.1 (1.5) [9]	15.4 (1.8) [12]	0.36	0.617	1
TF	P02787	17.7 (0.5) [10]	18 (0.5) [23]	0.28	0.129	1
TFRC	G3V0E5	11.5 (0.3) [7]	12.3 (0.7) [13]	0.83	0.001	0.589
TGFBI	Q15582	14.5 (0.8) [10]	14.7 (1.6) [23]	0.23	0.596	1
THBS1	P07996	14.5 (0.7) [6]	15.4 (0.6) [19]	0.87	0.022	1
TIMP1	P01033	16.3 (1.8) [10]	17.1 (2.4) [18]	0.77	0.349	1
TNXB	A0A140TA41	13.5 (0.6) [7]	13.2 (0.7) [11]	-0.22	0.472	1
TPI1	P60174	15.2 (1.1) [10]	14.5 (1.3) [21]	-0.69	0.133	1
TPM4	P67936	15.7 (1.1) [5]	15.1 (0.7) [15]	-0.63	0.28	1
TPP1	O14773	15 (1) [8]	14.4 (0.5) [13]	-0.54	0.185	1
TPP2	P29144	14 (0.9) [10]	15.1 (1.1) [20]	1.12	0.008	1
TREM2	Q9NZC2	13.5 (0.6) [8]	13.3 (0.8) [12]	-0.20	0.539	1
TTR	P02766	18.6 (1.4) [10]	18.5 (0.7) [23]	-0.09	0.848	1
TUBA1B	P68363	16 (1.5) [9]	16.5 (1.5) [21]	0.49	0.42	1
TUBB	P07437	16.6 (1) [7]	17.6 (1.4) [11]	1.01	0.096	1
TUBB4B	P68371	15.9 (1.1) [9]	16.1 (1.4) [22]	0.23	0.63	1
TXN	P10599	17 (0.9) [10]	16.3 (1) [14]	-0.72	0.079	1
UBC	F5H265	15.9 (0.8) [10]	14.9 (1.1) [19]	-1.00	0.008	1
VASN	Q6EMK4	14.7 (0.4) [10]	14.3 (0.5) [16]	-0.46	0.013	1
VCAM1	P19320	13.3 (0.7) [8]	13.4 (0.8) [12]	0.09	0.801	1
VCAN	P13611	14.3 (0.3) [8]	14.3 (1) [12]	0.03	0.911	1
VGF	O15240	15.4 (1.2) [10]	13.3 (0.9) [15]	-2.13	<0.001	0.067
VIM	P08670	18.2 (1.8) [8]	15 (1.4) [19]	-3.19	0.001	0.472
VIP	P01282	18 (0.3) [10]	18.4 (0.6) [21]	0.35	0.051	1
VTN	P04004	17.3 (0.8) [10]	18.2 (0.8) [22]	0.89	0.01	1
VWF	P04275	13.9 (0.7) [8]	14.4 (1) [17]	0.50	0.17	1
WFIKKN2	C9J6G4	15.2 (1.7) [9]	14.8 (1) [15]	-0.39	0.547	1
YWHAE	P62258	14.7 (1) [9]	15.4 (2.1) [14]	0.72	0.288	1
YWHAZ	P63104	14.9 (0.8) [10]	14.4 (1.2) [20]	-0.53	0.152	1
CAT	P04040	15.5 (1.1) [7]	14.8 (2.4) [14]	-0.67	0.399	1
CORO1A	P31146	13 (0.9) [8]	13.1 (0.8) [13]	0.09	0.812	1
CSTB	P04080	15.3 (1.1) [10]	14.9 (1.1) [11]	-0.39	0.416	1

**Protein Name**	**Uniprot ID**	**Ctrl** Mean (SD) [N]	**SAH start** Mean (SD) [N]	**log_2FC**	**P-value**	**Adj. P-value**
ENO1	P06733	15.8 (0.7) [10]	16.5 (1.8) [18]	0.71	0.152	1
FCGBP	Q9Y6R7	14.6 (1.3) [8]	14.8 (1.6) [12]	0.20	0.762	1
GPR37L1	O60883	14.9 (1.1) [10]	13.5 (1.2) [17]	-1.40	0.006	1
GSTO1	P78417	15.2 (0.8) [7]	14.5 (0.8) [11]	-0.77	0.062	1
HIST1H4A	P62805	14.8 (1.3) [9]	15.7 (2.6) [13]	0.84	0.335	1
HSPA1B	A0A0G2JIW1	15.5 (1) [9]	14.6 (1.2) [15]	-0.96	0.052	1
IGFBP2	P18065	14 (0.8) [7]	13.8 (1.2) [10]	-0.22	0.654	1
IGKV6D-21	A0A0A0MT36	13.7 (2) [5]	14.9 (1.1) [13]	1.22	0.252	1
MARCKS	P29966	12.3 (0.6) [8]	12.1 (0.9) [14]	-0.13	0.679	1
MIF	P14174	16.1 (0.7) [9]	16.2 (1.2) [12]	0.12	0.785	1
PEA15	Q15121	14.5 (0.7) [6]	14.7 (0.9) [11]	0.25	0.555	1
PGAM1	P18669	14.9 (0.6) [10]	15 (1.1) [15]	0.04	0.909	1
PI16	Q6UXB8	13.3 (0.6) [10]	13.1 (0.6) [18]	-0.20	0.434	1
PKM	P14618	15 (1) [10]	14.6 (0.9) [16]	-0.40	0.315	1
PRSS3	B1AN99	18.5 (1.5) [5]	18.7 (1.7) [14]	0.11	0.891	1
S100A1	P23297	13.7 (1) [5]	13.7 (1.2) [14]	0.06	0.914	1
S100A9	P06702	15.6 (1.7) [8]	16.4 (2) [17]	0.81	0.305	1
SELENBP1	Q13228	15.8 (1) [10]	15.7 (1.8) [12]	-0.03	0.958	1
SERPINB1	P30740	14 (0.6) [7]	14 (0.6) [11]	0.00	0.99	1
SH3BGRL	O75368	13.8 (0.7) [7]	13.6 (0.8) [10]	-0.25	0.507	1
SH3BGRL3	Q5T123	14 (0.6) [7]	14.5 (0.8) [11]	0.46	0.201	1
SLC4A1	P02730	14.6 (1.5) [6]	14.9 (2.8) [10]	0.25	0.819	1
TGOLN2	F8W8W7	12.2 (0.9) [10]	10.4 (1) [12]	-1.79	<0.001	0.16
TKT	P29401	14.7 (0.6) [7]	14.5 (1) [10]	-0.14	0.702	1
YWHAB	P31946	13.4 (0.7) [6]	13.6 (0.7) [12]	0.16	0.639	1
YWHAG	P61981	12.4 (0.6) [8]	13 (1) [10]	0.67	0.099	1
YWHAQ	P27348	13.4 (1.1) [8]	13.7 (0.9) [14]	0.31	0.519	1
AK1	P00568	15.2 (1.5) [6]	15.1 (1.1) [10]	-0.14	0.842	1
COL1A1	P02452	15.6 (1.5) [9]	14.6 (1.5) [13]	-0.99	0.152	1
CTSC	P53634	14.1 (0.8) [5]	13.6 (0.7) [10]	-0.45	0.33	1
HBE1	P02100	16.7 (1.9) [6]	16.9 (3.8) [10]	0.18	0.901	1
IGHV1-69	P01742	15 (0.4) [5]	15.3 (1.4) [13]	0.23	0.601	1
IGHV3OR16-12	A0A075B7B8	13.1 (0.4) [7]	14.1 (0.7) [16]	0.94	0.001	0.403
SULF2	Q8IWU5	14.3 (0.7) [10]	13.5 (1.2) [10]	-0.87	0.063	1
THBS2	P35442	14 (0.4) [6]	15.1 (1) [13]	1.11	0.003	1
KRT2	P35908	13.7 (1.9) [9]	14.2 (1.4) [11]	0.47	0.538	1
KRT9	P35527	14.4 (1.1) [7]	14.1 (1.4) [10]	-0.27	0.672	1

Ctrl: control subjects; SAH: subarachnoid hemorrhage; SD: standard deviation; N: number of subjects/patients; log_2FC: log_2(fold change).

Supplementary Table 2: Other CSF proteins in start samples versus end samples from patients with SAH.

Protein Name	Uniprot ID	SAH Start Mean (SD) [N]	SAH End Mean (SD) [N]	log_2FC	P-value	Adj. P-value
A1BG	P04217-2	17.6 (0.7) [22]	17 (0.4) [22]	-0.61	0.001	0.198
A2M	P01023	18.1 (0.9) [22]	17.3 (0.6) [22]	-0.89	0.003	1
ACSBG2	Q5FVE4	20.2 (0.6) [16]	21 (0.7) [16]	0.77	0.002	0.8
ACTA2	P62736	16.4 (0.9) [17]	17.3 (1.2) [17]	0.91	0.012	1
ACTB	P60709	16.7 (1.4) [22]	17.4 (1.1) [22]	0.72	0.042	1
ACTBL2	Q562R1	18.3 (1.2) [20]	19.3 (1.2) [20]	1.09	0.004	1
ADIPOQ	Q15848	13.8 (1.3) [11]	11.8 (0.7) [11]	-1.92	0.003	0.87
AFM	P43652	15.8 (0.5) [21]	15.5 (1) [21]	-0.31	0.175	1
AGT	P01019	16.3 (0.6) [22]	16.5 (0.3) [22]	0.14	0.349	1
AHSG	P02765	18.1 (1.8) [22]	17.2 (1.4) [22]	-0.89	0.091	1
ALB	P02768	19.9 (0.6) [22]	19.4 (0.5) [22]	-0.52	0.004	1
ALDOA	P04075	15 (2) [21]	15.7 (0.9) [21]	0.75	0.109	1
ALDOC	P09972	14.1 (1.1) [13]	14.6 (0.5) [13]	0.41	0.304	1
AMBP	P02760	17 (0.8) [22]	16 (0.5) [22]	-0.95	<0.001	0.005
APLP1	B7Z4G8	14.5 (1.2) [18]	15.6 (0.7) [18]	1.09	<0.001	0.161
APLP2	Q06481	14.1 (1.1) [13]	14.6 (0.8) [13]	0.50	0.228	1
APOA1	P02647	20 (1.1) [22]	18.6 (1) [22]	-1.43	0.001	0.289
APOA2	P02652	18.9 (0.9) [22]	17.6 (1.1) [22]	-1.36	<0.001	0.15
APOA4	P06727	17.3 (0.9) [21]	16.3 (1) [21]	-1.09	0.001	0.464
APOC3	B0YIW2	19.8 (2.2) [20]	17.1 (1.6) [20]	-2.65	0.001	0.226
APOC4-APOC2	A0A024R0T9	17.7 (2) [20]	15.2 (1.4) [20]	-2.55	0.001	0.174
APOD	C9JF17	16.5 (0.8) [21]	17.5 (1.3) [21]	0.96	0.005	1
APOH	P02749	16.4 (0.7) [20]	15.8 (0.7) [20]	-0.67	0.001	0.251
APOL1	O14791	16.1 (1.6) [19]	14.3 (1.4) [19]	-1.75	0.002	0.59
APOM	O95445	17.8 (1.2) [18]	15.7 (1.1) [18]	-2.12	<0.001	0.085
APP	P05067	13.5 (1) [18]	14 (0.9) [18]	0.53	0.034	1
ARPC4-TTLL3	A0A0A6YYG9	16.8 (0.5) [20]	16.4 (0.4) [20]	-0.38	0.005	1
ATP6AP1	Q15904	13.7 (1) [15]	14.4 (0.6) [15]	0.74	<0.001	0.136
ATRN	O75882	14.6 (0.4) [14]	13.9 (0.5) [14]	-0.77	0.003	1
AZGP1	P25311	17 (0.6) [21]	17 (0.7) [21]	0.08	0.622	1
B2M	P61769	15.4 (2) [22]	17 (1) [22]	1.66	0.005	1
B4GAT1	O43505	14.8 (1.3) [17]	16.3 (1.8) [17]	1.42	0.01	1
BCAN	Q96GW7	14.8 (1.3) [15]	14.9 (1.2) [15]	0.17	0.725	1
BLVRB	P30043	16.1 (2.2) [13]	16.1 (1.8) [13]	0.02	0.969	1
BTD	P43251	15.6 (0.4) [20]	15.4 (0.5) [20]	-0.13	0.278	1
C1QA	P02745	15.3 (0.5) [21]	15.6 (0.7) [21]	0.25	0.168	1
C1QB	D6R934	15.7 (0.6) [21]	16.3 (1.1) [21]	0.60	0.023	1
C1QC	P02747	17.5 (0.5) [20]	17.8 (0.7) [20]	0.24	0.257	1

**Protein Name**	**Uniprot ID**	**SAH Start** Mean (SD) [N]	**SAH End** Mean (SD) [N]	**log$_2$FC**	**P-value**	**Adj. P-value**
C1R	B4DPQ0	16 (0.4) [21]	16 (0.5) [21]	0.07	0.621	1
C1RL	Q9NZP8	14.9 (0.9) [14]	14 (0.6) [14]	-0.92	0.001	0.213
C1S	P09871	16.3 (0.4) [21]	16.5 (0.5) [21]	0.16	0.221	1
C2	P06681	15.5 (0.5) [20]	15.5 (0.5) [20]	-0.02	0.909	1
C2orf40	B8ZZE5	14.9 (1.4) [14]	15.6 (1.2) [14]	0.72	0.071	1
C3	P01024	17.9 (0.7) [22]	17.6 (0.6) [22]	-0.29	0.158	1
C4A	A0A0G2JPR0	15.7 (0.8) [21]	15.9 (0.7) [21]	0.20	0.389	1
C4B	P0C0L5	16.9 (0.3) [22]	17 (0.4) [22]	0.13	0.208	1
C4BPA	P04003	16.2 (1.9) [18]	13.7 (1.3) [18]	-2.55	<0.001	0.086
C5	P01031	15.8 (0.7) [20]	14.8 (0.4) [20]	-0.95	<0.001	0.005
C7	P10643	15.8 (0.6) [21]	16.5 (0.7) [21]	0.65	0.002	0.561
C8A	P07357	16 (0.7) [22]	15.3 (0.6) [22]	-0.71	0.004	1
C8B	F5GY80	14.7 (0.5) [19]	14 (0.4) [19]	-0.67	<0.001	0.037
C8G	P07360	14.7 (0.4) [12]	14 (0.6) [12]	-0.68	0.011	1
C9	P02748	16 (0.5) [21]	15.8 (0.8) [21]	-0.25	0.2	1
CA1	P00915	17.3 (2.5) [18]	18.4 (1.8) [18]	1.10	0.065	1
CA2	P00918	15.8 (3.1) [17]	15.3 (2.4) [17]	-0.48	0.632	1
CBR1	P16152	14.9 (1.1) [12]	14.1 (0.8) [12]	-0.80	0.069	1
CD44	H0YD13	15.3 (1) [17]	16.3 (0.9) [17]	1.04	0.013	1
CDH13	P55290	14.1 (0.9) [18]	14.8 (0.9) [18]	0.67	0.046	1
CFD	K7ERG9	15.4 (0.8) [17]	16.6 (0.6) [17]	1.19	<0.001	0.075
CFH	P08603	16.9 (0.9) [22]	16.2 (0.8) [22]	-0.64	0.029	1
CFHR1	B1AKG0	16 (1) [20]	15 (0.8) [20]	-0.99	<0.001	0.033
CFI	E7ETH0	15.4 (0.5) [20]	14.9 (0.4) [20]	-0.47	<0.001	0.131
CFL1	E9PK25	15.2 (1) [19]	15 (1.2) [19]	-0.25	0.526	1
CHGA	P10645	14.5 (1) [16]	15.8 (0.9) [16]	1.25	0.004	1
CHGB	P05060	14 (1.3) [19]	15.6 (0.8) [19]	1.56	<0.001	0.072
CHI3L1	P36222	15.5 (2) [19]	16.7 (1) [19]	1.19	0.018	1
CHI3L2	Q15782	14.4 (1.6) [12]	13.9 (1.5) [12]	-0.48	0.373	1
CHL1	O00533	13.6 (1.1) [17]	14.6 (0.8) [17]	0.98	0.005	1
CKB	P12277	16.5 (1.6) [14]	14.9 (1.8) [14]	-1.56	0.039	1
CLEC3B	E9PHK0	15.8 (0.5) [20]	16.1 (0.5) [20]	0.36	0.02	1
CLSTN1	O94985	15 (0.9) [13]	16.1 (0.5) [13]	1.11	0.002	0.53
CLU	P10909	16.4 (0.6) [22]	17 (1) [22]	0.65	0.012	1
CNDP1	Q96KN2	15.2 (1) [21]	16 (0.6) [21]	0.77	0.001	0.318
CNTN1	Q12860	14.1 (1.1) [15]	14.8 (0.5) [15]	0.75	0.013	1
CNTN2	A0A1W2PQ11	14.2 (1.2) [15]	14.8 (0.7) [15]	0.56	0.057	1
COL18A1	P39060	14.4 (1.1) [10]	14.7 (1.1) [10]	0.30	0.556	1
COL6A1	A0A087X0S5	13.6 (1.2) [14]	14.6 (0.3) [14]	0.93	0.01	1
COL6A3	P12111	13.8 (0.6) [11]	14.1 (0.3) [11]	0.26	0.288	1
CP	P00450	16.8 (0.6) [22]	16.6 (0.6) [22]	-0.25	0.188	1
CPB2	A0A087WSY5	15.7 (0.7) [21]	15.7 (1.5) [21]	0.01	0.971	1

**Protein Name**	**Uniprot ID**	**SAH Start** Mean (SD) [N]	**SAH End** Mean (SD) [N]	**log$_2$FC**	**P-value**	**Adj. P-value**
CPE	P16870	15 (1.3) [19]	15.4 (1) [19]	0.36	0.19	1
CPN1	P15169	15.4 (0.7) [12]	14.2 (1) [12]	-1.20	0.017	1
CPQ	Q9Y646	14.3 (1.1) [11]	14.4 (1.1) [11]	0.06	0.833	1
CPVL	Q9H3G5	14.2 (0.8) [13]	14.3 (0.9) [13]	0.11	0.715	1
CRP	P02741	14.2 (1) [12]	14.9 (1.4) [12]	0.77	0.062	1
CRTAC1	A0A0C4DFP6	14.3 (1) [16]	14.7 (0.5) [16]	0.41	0.035	1
CSF1	P09603	13.9 (0.9) [10]	14.5 (0.9) [10]	0.65	0.174	1
CST3	P01034	16 (1.6) [22]	17.5 (0.9) [22]	1.55	0.001	0.205
CTSB	P07858	14.7 (1.3) [11]	14.9 (1.6) [11]	0.26	0.676	1
CTSD	A0A1B0GV23	15.5 (1.2) [19]	16.2 (1.1) [19]	0.76	0.037	1
CTSL	P07711	15 (1) [12]	15 (0.9) [12]	0.06	0.866	1
DAG1	Q14118	14.3 (1.1) [12]	15.2 (0.2) [12]	0.94	0.01	1
DBI	A0A0A0MTI5	14.7 (0.5) [15]	15.1 (0.7) [15]	0.43	0.061	1
DKK3	F6SYF8	14.4 (1.5) [18]	15.6 (0.9) [18]	1.24	0.006	1
ECM1	Q16610	14.3 (0.4) [18]	14.5 (0.4) [18]	0.21	0.106	1
EFEMP1	A0A0U1RQV3	15.6 (1.4) [15]	16.2 (0.7) [15]	0.61	0.153	1
EIF5A	I3L397	13.9 (1.2) [14]	14.6 (1) [14]	0.75	0.083	1
ENDOD1	O94919	14.5 (0.7) [12]	15 (0.4) [12]	0.55	0.066	1
ENO2	P09104	15.3 (2.6) [15]	14.4 (1) [15]	-0.99	0.193	1
ENPP2	E7EUF1	15.6 (1.2) [20]	16.5 (1) [20]	0.84	0.022	1
EPHA4	E9PG71	14 (1.2) [10]	14.8 (0.8) [10]	0.85	0.071	1
ERN1	O75460	17.7 (2) [12]	14.9 (1.8) [12]	-2.72	0.005	1
F12	P00748	17.1 (0.9) [20]	16.2 (0.6) [20]	-0.85	0.001	0.479
F2	P00734	16.6 (0.7) [20]	16.1 (0.4) [20]	-0.43	0.005	1
F5	A0A0A0MRJ7	14.9 (0.5) [20]	14.7 (1) [20]	-0.19	0.408	1
F9	P00740	14.1 (0.6) [18]	13.2 (0.5) [18]	-0.89	<0.001	0.005
FAM3C	Q92520	14 (0.9) [14]	15 (0.4) [14]	0.99	0.001	0.324
FBLN1	P23142	15.4 (1) [21]	16.4 (1) [21]	1.00	0.002	0.776
FCGR3A	A0A1W2PQB1	14 (1.1) [15]	15.5 (0.8) [15]	1.45	0.001	0.237
FETUB	Q9UGM5	15.4 (1.3) [16]	14.4 (0.7) [16]	-1.04	0.002	0.633
FGA	P02671	16.7 (1.1) [22]	16 (0.9) [22]	-0.68	0.051	1
FGB	P02675	18.1 (1.5) [22]	17.7 (1.2) [22]	-0.37	0.388	1
FGG	P02679	17.3 (1.5) [22]	16.8 (1.1) [22]	-0.43	0.295	1
FN1	P02751	16.6 (0.7) [22]	16.6 (0.7) [22]	-0.04	0.846	1
FSTL1	Q12841	13.2 (1.3) [15]	14.1 (1.5) [15]	0.92	0.152	1
FUCA2	Q9BTY2	13.8 (1.2) [13]	14.1 (1.1) [13]	0.28	0.544	1
GAPDH	P04406	15.2 (1.2) [19]	15.5 (1.1) [19]	0.22	0.572	1
GC	P02774	17.2 (0.7) [22]	17 (0.7) [22]	-0.22	0.271	1
GDI2	P50395	14.3 (1) [14]	14.3 (1) [14]	0.02	0.951	1
GGH	Q92820	14.3 (0.5) [12]	14 (0.7) [12]	-0.35	0.186	1
GM2A	P17900	14.6 (1.3) [17]	15.7 (0.8) [17]	1.04	0.013	1
GOT1	P17174	14.6 (0.7) [10]	15.4 (0.6) [10]	0.84	0.001	0.198

Protein Name	Uniprot ID	SAH Start<br>Mean (SD) [N]	SAH End<br>Mean (SD) [N]	log_2FC	P-value	Adj. P-value
GPLD1	P80108	15.9 (1.1) [13]	14.4 (1) [13]	-1.50	0.004	1
GPX3	A0A087X1J7	15.9 (0.6) [20]	15.9 (0.6) [20]	0.00	0.985	1
GSN	P06396	16.1 (0.5) [22]	16.4 (0.5) [22]	0.31	0.052	1
GSTP1	P09211	15.3 (1.1) [16]	15.7 (0.9) [16]	0.38	0.352	1
HBA1	P69905	20.1 (2.6) [22]	20.9 (2.4) [22]	0.86	0.21	1
HBB	P68871	20.2 (3.1) [22]	21.2 (2.8) [22]	0.98	0.196	1
HBD	P02042	18.1 (2.4) [19]	18.5 (1.9) [19]	0.47	0.463	1
HBG2	P69892	16.6 (2.5) [14]	16.3 (2.7) [14]	-0.36	0.732	1
HGFAC	D6RAR4	15.1 (0.6) [13]	14 (0.5) [13]	-1.09	0.001	0.323
HP	P00738	18.9 (2.1) [22]	16 (2.3) [22]	-2.93	0.001	0.357
HPR	P00739	16.7 (1.7) [20]	15.1 (2) [20]	-1.56	0.037	1
HPX	P02790	18.4 (0.5) [22]	17.7 (0.7) [22]	-0.70	0.001	0.221
HRG	P04196	16.8 (0.7) [22]	16 (0.5) [22]	-0.85	<0.001	0.028
HSP90AA1	P07900	15 (1.1) [14]	14.6 (1.1) [14]	-0.45	0.333	1
HSPA5	P11021	13.9 (0.6) [10]	14.8 (0.6) [10]	0.90	<0.001	0.137
HSPA8	P11142	15.5 (2.6) [18]	15.2 (1.3) [18]	-0.37	0.598	1
HSPG2	P98160	13.6 (1.6) [16]	14.3 (1) [16]	0.65	0.127	1
HTRA1	Q92743	13.4 (0.8) [12]	14.4 (0.6) [12]	0.98	0.011	1
ICOSLG	K4DIA0	13.9 (1.3) [16]	15.3 (0.4) [16]	1.41	0.001	0.233
IGFBP6	P24592	15.9 (1.6) [18]	17.5 (1) [18]	1.58	0.008	1
IGFBP7	Q16270	15.8 (1.6) [22]	16.5 (1.1) [22]	0.66	0.129	1
IGHA1	A0A286YEY1	19.2 (1.1) [22]	18.3 (0.9) [22]	-0.90	0.005	1
IGHA2	A0A286YEY5	19.3 (1.2) [22]	18.3 (0.8) [22]	-1.02	0.005	1
IGHD	A0A0A0MS09	16.2 (1.2) [13]	15.4 (1.3) [13]	-0.85	0.165	1
IGHG1	P01857	20.4 (0.7) [22]	19.6 (0.4) [22]	-0.79	<0.001	0.011
IGHG2	P01859	20.3 (0.6) [22]	19.7 (0.6) [22]	-0.62	0.001	0.462
IGHG3	P01860	17.8 (1) [20]	16.4 (1) [20]	-1.40	<0.001	0.096
IGHG4	A0A286YFJ8	16.7 (1) [22]	15.9 (1.1) [22]	-0.83	0.001	0.45
IGHM	A0A1B0GUU9	19.2 (1.9) [20]	17.3 (1.3) [20]	-1.94	0.002	0.819
IGHV1-18	A0A0C4DH31	15 (1.1) [11]	14.5 (0.9) [11]	-0.53	0.199	1
IGHV1OR15-1	A0A075B7D0	17.5 (1.5) [19]	17 (1.5) [19]	-0.57	0.094	1
IGHV2-26	A0A0B4J1V2	14.2 (1.2) [15]	13.8 (0.7) [15]	-0.37	0.237	1
IGHV3-15	A0A0B4J1V0	16.1 (0.6) [20]	16.1 (0.6) [20]	-0.01	0.97	1
IGHV3-30	P01768	17.5 (0.6) [20]	16.9 (0.5) [20]	-0.54	<0.001	0.072
IGHV3-38	A0A0C4DH36	15.5 (0.6) [20]	15.4 (0.7) [20]	-0.11	0.572	1
IGHV3-64D	A0A0J9YX35	16 (0.7) [16]	15.9 (0.6) [16]	-0.10	0.678	1
IGHV3-7	P01780	17.8 (0.6) [20]	17.4 (0.5) [20]	-0.41	0.001	0.328
IGHV3-72	A0A0B4J1Y9	17.3 (0.8) [20]	16.6 (0.6) [20]	-0.66	0.001	0.202
IGHV3OR16-9	A0A0B4J2B5	20.5 (0.6) [22]	20 (0.6) [22]	-0.49	0.022	1
IGHV4-34	P06331	15.9 (1) [20]	15.3 (0.7) [20]	-0.58	0.018	1

Protein Name	Uniprot ID	SAH Start Mean (SD) [N]	SAH End Mean (SD) [N]	log_2FC	P-value	Adj. P-value
IGHV5-51	A0A0C4DH38	16.4 (0.8) [21]	16.3 (1.8) [21]	-0.13	0.777	1
IGKC	P01834	21 (1.4) [22]	20.4 (1.4) [22]	-0.64	0.18	1
IGKV1-12	A0A0C4DH73	17.8 (1.1) [21]	17.3 (0.8) [21]	-0.52	0.078	1
IGKV1-16	P04430	14.8 (0.8) [12]	14.4 (0.7) [12]	-0.40	0.18	1
IGKV1-17	P01599	16.5 (0.8) [20]	15.7 (0.8) [20]	-0.82	<0.001	0.035
IGKV1-27	A0A075B6S5	16.4 (0.7) [12]	15.8 (0.5) [12]	-0.64	0.045	1
IGKV1-5	P01602	16.8 (0.7) [20]	16.8 (0.7) [20]	0.01	0.963	1
IGKV1-8	A0A0C4DH67	17.4 (1.1) [21]	16.8 (0.9) [21]	-0.58	0.084	1
IGKV1D-33	P01593	18.3 (1) [21]	17.6 (0.8) [21]	-0.72	0.007	1
IGKV1D-37	A0A075B6S9	17.9 (1.3) [11]	16 (1) [11]	-1.98	0.002	0.719
IGKV2-28	A0A075B6P5	16.9 (0.9) [18]	16.4 (0.6) [18]	-0.49	0.019	1
IGKV2-29	A2NJV5	17.2 (1) [20]	16.3 (0.7) [20]	-0.91	<0.001	0.001
IGKV2D-24	A0A075B6R9	17.4 (1.1) [20]	16.6 (0.8) [20]	-0.74	0.003	1
IGKV3-15	P01624	17.7 (0.6) [20]	17.8 (0.9) [20]	0.04	0.846	1
IGKV3-20	P01619	19.2 (0.9) [22]	18.8 (1) [22]	-0.45	0.141	1
IGKV3-7	A0A075B6H7	18.6 (1.6) [17]	18.9 (1.3) [17]	0.33	0.477	1
IGKV3D-11	A0A0A0MRZ8	18.1 (0.5) [20]	17.6 (0.5) [20]	-0.45	0.003	1
IGKV3D-15	A0A087WSY6	18.2 (0.9) [19]	17.1 (0.9) [19]	-1.09	<0.001	0.112
IGKV3D-20	A0A0C4DH25	19.4 (1.2) [20]	18.3 (0.9) [20]	-1.11	<0.001	0.088
IGKV4-1	P06312	17.9 (0.7) [20]	17.4 (0.5) [20]	-0.56	0.006	1
IGLC3	P0DOY3	20.8 (0.7) [20]	20.3 (0.5) [20]	-0.51	0.026	1
IGLL5	A0A0B4J231	18.8 (0.8) [22]	18.1 (0.6) [22]	-0.72	0.002	0.596
IGLV1-47	P01700	17.1 (0.8) [21]	17.1 (1.6) [21]	0.01	0.985	1
IGLV1-51	P01701	16.7 (0.8) [17]	15.6 (0.7) [17]	-1.03	0.001	0.318
IGLV3-10	A0A075B6K4	16.3 (0.9) [15]	15.3 (0.7) [15]	-1.04	0.003	1
IGLV3-19	P01714	15.1 (0.5) [18]	14.6 (0.6) [18]	-0.45	0.008	1
IGLV3-21	P80748	16.3 (1.1) [15]	15.8 (1.1) [15]	-0.51	0.039	1
IGLV3-25	P01717	15.9 (0.9) [19]	14.8 (0.7) [19]	-1.15	<0.001	0.062
IGLV3-9	A0A075B6K5	16.5 (1.3) [20]	15.3 (0.7) [20]	-1.27	0.001	0.172
IGLV6-57	P01721	15.5 (0.8) [18]	14.8 (0.8) [18]	-0.65	0.009	1
IGLV7-46	A0A075B6I9	16.2 (0.7) [19]	15.4 (0.9) [19]	-0.85	0.004	1
IGLV8-61	A0A075B6I0	16.6 (0.8) [10]	15.6 (0.5) [10]	-0.99	<0.001	0.065
IGSF8	Q969P0	14.6 (1.1) [12]	15.3 (0.6) [12]	0.67	0.096	1
ISLR	O14498	13.9 (1.3) [14]	15.2 (0.7) [14]	1.32	0.008	1
ITIH1	P19827	17.3 (1.6) [22]	15.7 (0.6) [22]	-1.66	<0.001	0.061
ITIH3	Q06033	15.6 (0.8) [19]	14.5 (0.6) [19]	-1.08	<0.001	0.051
ITIH4	Q14624	17.5 (0.7) [22]	16.5 (0.8) [22]	-0.95	<0.001	0.063
ITPR2	Q14571	18.9 (0.9) [20]	18.3 (1) [20]	-0.58	0.119	1
JCHAIN	D6RD17	18.3 (1.5) [19]	17 (1.3) [19]	-1.30	0.01	1
KLK6	Q92876	15 (1.3) [19]	15.8 (1) [19]	0.89	0.022	1
KNG1	P01042	17.8 (0.8) [21]	17.1 (0.5) [21]	-0.63	0.002	0.637
KRT1	P04264	14.6 (1.3) [12]	15.8 (1.9) [12]	1.13	0.114	1

Protein Name	Uniprot ID	SAH Start Mean (SD) [N]	SAH End Mean (SD) [N]	log_2FC	P-value	Adj. P-value
LAMP2	P13473	13.9 (1.2) [18]	15.1 (0.8) [18]	1.21	0.001	0.262
LBP	P18428	14.6 (0.7) [13]	13.8 (0.7) [13]	-0.73	0.017	1
LCAT	P04180	14.9 (0.6) [17]	14.7 (0.5) [17]	-0.21	0.159	1
LCP1	P13796	14 (0.6) [14]	15.3 (1.3) [14]	1.26	0.003	1
LDHA	P00338	14.3 (1) [13]	15.5 (0.9) [13]	1.20	0.007	1
LDHB	P07195	15.2 (2.8) [19]	15.3 (0.6) [19]	0.10	0.874	1
LGALS1	P09382	14.2 (0.4) [10]	15.3 (1.1) [10]	1.03	0.023	1
LGALS3BP	Q08380	15.5 (0.8) [20]	16.2 (0.8) [20]	0.70	0.003	1
LIAS	A0A1W2PNQ5	15.9 (1.3) [13]	14.5 (1) [13]	-1.42	<0.001	0.169
LMAN2	D6RBV2	14 (1.3) [11]	14.8 (0.3) [11]	0.80	0.077	1
LRG1	P02750	16.9 (0.4) [20]	17 (0.5) [20]	0.04	0.764	1
LSAMP	H3BLU2	13.8 (1.2) [12]	14.6 (0.9) [12]	0.83	0.051	1
LTF	E7EQB2	14.4 (1.4) [13]	15.2 (1.4) [13]	0.83	0.12	1
LUM	P51884	16 (0.5) [21]	15.6 (0.3) [21]	-0.38	0.001	0.388
LYVE1	Q9Y5Y7	13.9 (1.4) [18]	15.6 (1.1) [18]	1.70	0.001	0.465
MDH1	P40925	15.4 (0.9) [12]	15.9 (0.6) [12]	0.55	0.108	1
MMP2	P08253	14.1 (1.5) [11]	15.3 (0.6) [11]	1.23	0.04	1
NCAM1	P13591	14.1 (1.2) [19]	15.4 (0.9) [19]	1.32	0.002	0.58
NEGR1	Q7Z3B1	13.6 (1.4) [13]	14.3 (1.2) [13]	0.72	0.055	1
NEO1	Q92859	13.9 (1.1) [10]	14.1 (0.4) [10]	0.20	0.471	1
NPC2	E7EMS2	15.3 (1.1) [14]	16.5 (1) [14]	1.20	0.012	1
NPTX1	Q15818	13.9 (1.8) [13]	14.6 (1) [13]	0.70	0.069	1
NRCAM	C9JYY6	14.1 (1.1) [14]	15 (0.7) [14]	0.95	0.001	0.248
NRXN3	A0A0U1RQC5	13.1 (1) [10]	13.8 (0.8) [10]	0.78	0.016	1
NUCB1	Q02818	13.2 (1.1) [13]	14 (0.8) [13]	0.78	0.043	1
OGN	P20774	14.2 (1.8) [17]	15.7 (0.8) [17]	1.54	0.007	1
OMG	P23515	14.9 (1) [10]	15 (0.6) [10]	0.10	0.647	1
ORM1	P02763	19.5 (0.5) [22]	19.6 (0.7) [22]	0.14	0.342	1
ORM2	P19652	17.8 (0.5) [22]	18.1 (0.7) [22]	0.29	0.027	1
PAM	P19021	13.9 (1) [11]	14.6 (0.5) [11]	0.67	0.02	1
PCOLCE	Q15113	15.1 (1.3) [14]	15.9 (0.9) [14]	0.86	0.07	1
PCSK1N	Q9UHG2	13.9 (1.3) [19]	15.2 (0.6) [19]	1.33	<0.001	0.151
PEBP1	P30086	15.8 (1) [17]	16 (0.6) [17]	0.22	0.384	1
PEBP4	Q96S96	14.7 (1.2) [10]	15.6 (0.6) [10]	0.88	0.041	1
PEPD	P12955	12.6 (0.5) [13]	13.3 (0.5) [13]	0.73	<0.001	0.037
PFN1	P07737	16.1 (1.5) [18]	16.1 (0.9) [18]	0.01	0.981	1
PGK1	P00558	16 (3.1) [15]	15.8 (0.9) [15]	-0.19	0.827	1
PGLYRP2	Q96PD5	15.6 (0.8) [21]	14.7 (1.2) [21]	-0.91	0.009	1
PLG	P00747	17.3 (1) [22]	16.2 (0.8) [22]	-1.08	0.001	0.372
PLTP	P55058	15 (1.2) [21]	16.2 (0.9) [21]	1.20	0.002	0.853
PLXDC2	Q6UX71	13.4 (1.2) [16]	14.9 (0.6) [16]	1.45	0.001	0.233
PPIA	P62937	15.8 (1.3) [21]	16.4 (1.1) [21]	0.59	0.033	1

Protein Name	Uniprot ID	SAH Start Mean (SD) [N]	SAH End Mean (SD) [N]	log_2FC	P-value	Adj. P-value
PPIB	P23284	14.3 (0.8) [14]	14.6 (0.9) [14]	0.32	0.341	1
PRDX1	Q06830	16.6 (2.3) [21]	17.2 (1.9) [21]	0.59	0.333	1
PRDX2	P32119	16.8 (3) [22]	17.9 (1.9) [22]	1.15	0.09	1
PRDX6	P30041	15.8 (1.5) [17]	15.3 (1.2) [17]	-0.58	0.208	1
PRG4	A0A0U1RR20	13.7 (0.8) [16]	13.7 (1.1) [16]	-0.01	0.985	1
PROC	E7END6	13.9 (0.7) [17]	13.6 (0.5) [17]	-0.36	0.072	1
PROCR	Q9UNN8	13.9 (0.7) [17]	14.4 (0.6) [17]	0.44	0.095	1
PROS1	P07225	15.8 (0.5) [21]	15.2 (0.6) [21]	-0.61	0.002	0.824
PROZ	P22891	14.9 (1) [11]	13.2 (0.6) [11]	-1.70	<0.001	0.107
PTGDS	P41222	18.1 (1.9) [22]	19.9 (0.8) [22]	1.78	0.001	0.207
PTPRZ1	P23471	13.9 (1) [11]	14.5 (0.7) [11]	0.59	0.139	1
QSOX1	O00391	14 (0.4) [16]	14.4 (0.4) [16]	0.45	0.008	1
RARRES2	Q99969	14.3 (1) [12]	14.9 (0.6) [12]	0.57	0.122	1
RBP4	P02753	16.4 (0.9) [22]	16.3 (0.8) [22]	-0.07	0.73	1
S100A8	P05109	16 (1.9) [17]	17.4 (2.1) [17]	1.43	0.015	1
SAA1	P0DJI8	16.3 (1.1) [11]	15.2 (1.4) [11]	-1.02	0.033	1
SAA4	P35542	16.7 (1.1) [20]	15.6 (0.6) [20]	-1.16	<0.001	0.121
SCG2	P13521	12.8 (0.8) [16]	13.5 (0.6) [16]	0.75	0.001	0.227
SCG3	Q8WXD2	14 (1.3) [14]	15.4 (0.6) [14]	1.47	<0.001	0.062
SCG5	P05408-2	14.7 (1.1) [15]	15.8 (1.1) [15]	1.15	0.007	1
SCRG1	O75711	16 (1.4) [10]	16.9 (0.6) [10]	0.85	0.081	1
SELL	P14151	15.9 (0.6) [16]	15.4 (0.4) [16]	-0.48	0.012	1
SERPINA1	P01009	18.7 (0.6) [22]	18.6 (0.7) [22]	-0.14	0.418	1
SERPINA10	G3V2W1	13.6 (1) [13]	12.1 (1) [13]	-1.45	0.005	1
SERPINA3	P01011	17.5 (0.7) [22]	17.9 (0.8) [22]	0.38	0.091	1
SERPINA4	P29622	15.6 (0.5) [20]	14.9 (0.4) [20]	-0.73	<0.001	0
SERPINA5	P05154	14.5 (0.5) [19]	14.3 (0.7) [19]	-0.23	0.149	1
SERPINA6	P08185	16 (0.7) [22]	15.4 (0.6) [22]	-0.56	0.007	1
SERPINA7	P05543	14.9 (0.5) [21]	14.9 (0.6) [21]	-0.01	0.943	1
SERPINC1	P01008	17 (0.6) [22]	17 (0.6) [22]	0.07	0.72	1
SERPIND1	P05546	16.1 (0.6) [22]	15.3 (0.5) [22]	-0.84	<0.001	0.018
SERPINF1	P36955	16.6 (1) [22]	17.3 (0.8) [22]	0.66	0.025	1
SERPINF2	P08697	16.8 (0.6) [22]	16.3 (0.5) [22]	-0.50	0.004	1
SERPING1	P05155	17 (0.3) [22]	16.7 (0.3) [22]	-0.26	0.004	1
SHBG	I3L145	15 (1.1) [13]	13.7 (0.5) [13]	-1.29	0.004	1
SKP1	E5RJR5	14.1 (0.6) [12]	14.2 (0.9) [12]	0.11	0.645	1
SOD1	P00441	15.5 (1.8) [16]	16.1 (0.4) [16]	0.53	0.292	1
SOD3	P08294	14.5 (1.1) [20]	15.4 (0.9) [20]	0.90	0.012	1
SPARC	P09486	14.7 (0.8) [21]	15.7 (1) [21]	0.99	0.003	0.895
SPARCL1	Q14515	13.5 (0.9) [19]	14.5 (0.6) [19]	0.95	0.003	1
SPP1	P10451	14.5 (2.4) [21]	16.2 (1.1) [21]	1.68	0.01	1
TALDO1	P37837	15.4 (1.9) [11]	15 (1.3) [11]	-0.35	0.657	1

Protein Name	Uniprot ID	SAH Start Mean (SD) [N]	SAH End Mean (SD) [N]	log_2FC	P-value	Adj. P-value
TF	P02787	17.9 (0.5) [22]	17.8 (0.7) [22]	-0.15	0.433	1
TGFBI	Q15582	14.8 (1.6) [22]	15.6 (1.1) [22]	0.75	0.064	1
THBS1	P07996	15.2 (0.6) [12]	14.4 (0.6) [12]	-0.86	0.004	1
THY1	E9PIM6	15 (1.2) [17]	16.1 (0.8) [17]	1.10	<0.001	0.091
TIMP1	P01033	17.2 (2.4) [17]	18.6 (1.9) [17]	1.39	0.096	1
TPI1	P60174	14.5 (1.3) [19]	14.7 (1.1) [19]	0.13	0.762	1
TPM4	P67936	15.2 (0.7) [10]	14.1 (2.8) [10]	-1.12	0.225	1
TPP1	O14773	14.5 (0.4) [11]	14.2 (1.2) [11]	-0.29	0.428	1
TPP2	P29144	15.1 (1.2) [16]	14.7 (0.9) [16]	-0.36	0.231	1
TREM2	Q9NZC2	13.4 (0.9) [10]	14.4 (0.5) [10]	0.95	0.02	1
TTR	P02766	18.6 (0.7) [22]	19.2 (0.8) [22]	0.63	0.014	1
TUBA1B	P68363	16.7 (1.5) [18]	14.9 (1.6) [18]	-1.84	0.004	1
TUBB4B	P68371	16.3 (1.4) [14]	15.1 (1.3) [14]	-1.18	0.068	1
TXN	P10599	16.1 (0.9) [13]	17.3 (0.8) [13]	1.18	0.001	0.458
UBC	F5H265	14.9 (1) [17]	16.1 (0.7) [17]	1.29	<0.001	0.128
VASN	Q6EMK4	14.3 (0.5) [14]	14.4 (0.3) [14]	0.13	0.253	1
VCAN	P13611	14.4 (0.9) [10]	14.5 (0.8) [10]	0.07	0.813	1
VGF	O15240	13.3 (0.9) [13]	14.3 (0.9) [13]	0.95	0.009	1
VIM	P08670	15 (1.3) [17]	16.7 (1.8) [17]	1.70	0.006	1
VIP	P01282	18.3 (0.6) [19]	18.3 (0.6) [19]	-0.04	0.76	1
VTN	P04004	18.1 (0.9) [20]	17.2 (0.5) [20]	-0.89	<0.001	0.083
WFIKKN2	C9J6G4	14.9 (1) [13]	14.9 (0.9) [13]	-0.02	0.95	1
YWHAE	P62258	15.5 (2.2) [13]	14.6 (1.3) [13]	-0.83	0.28	1
YWHAZ	P63104	14.4 (1.2) [18]	15.3 (1) [18]	0.91	0.013	1
CAT	P04040	14.8 (2.4) [14]	15.6 (1.3) [14]	0.80	0.235	1
CSTB	P04080	14.7 (0.9) [10]	15.8 (1) [10]	1.08	0.009	1
ENO1	P06733	16.5 (1.9) [17]	16 (1) [17]	-0.48	0.357	1
GPR37L1	O60883	13.6 (1.3) [15]	14.4 (1.2) [15]	0.82	0.156	1
GSTO1	P78417	14.3 (0.7) [10]	15.6 (0.7) [10]	1.25	0.01	1
HIST1H4A	P62805	15.7 (2.6) [13]	16.7 (2.1) [13]	1.07	0.329	1
HSPA1B	A0A0G2JIW1	14.6 (1.3) [10]	15.4 (1.2) [10]	0.78	0.217	1
MARCKS	P29966	12.2 (0.9) [11]	12.4 (0.6) [11]	0.19	0.572	1
MIF	P14174	16 (1) [11]	16.5 (0.8) [11]	0.49	0.195	1
PGAM1	P18669	15 (1.1) [14]	15.1 (0.8) [14]	0.13	0.748	1
PI16	Q6UXB8	13.1 (0.6) [16]	13.4 (1.3) [16]	0.27	0.479	1
PKM	P14618	14.7 (0.9) [14]	15.2 (0.7) [14]	0.51	0.12	1
S100A9	P06702	16.3 (2.1) [15]	16.9 (1.6) [15]	0.54	0.468	1
SELENBP1	Q13228	15.6 (1.8) [11]	15.4 (0.9) [11]	-0.20	0.753	1
YWHAQ	P27348	13.9 (0.9) [11]	13.9 (1.2) [11]	0.00	0.997	1
COL1A1	P02452	14.8 (1.4) [12]	16.1 (2) [12]	1.24	0.028	1

SAH: subarachnoid hemorrhage; SD: standard deviation; N: number of patients; log_2FC: log_2(fold change).

Supplementary Table 3: Other CSF proteins in start samples from weaned versus shunted SAH patients.

Protein Name	Uniprot ID	SAH Weaned<br>Mean (SD) [N]	SAH Shunted<br>Mean (SD) [N]	log_2FC	P-value	Adj. P-value
CDH13	P55290	14.2 (0.7) [11]	14.1 (1.1) [8]	-0.15	0.727	1
CDH2	P19022	14.7 (0.9) [6]	14.8 (1.2) [5]	0.05	0.94	1
CARTPT	Q16568	12.6 (1) [7]	13.2 (0.7) [6]	0.60	0.215	1
CASP14	P31944	14.6 (1) [8]	15.5 (1.1) [7]	0.85	0.154	1
CBR1	P16152	15.6 (1.1) [10]	14.4 (0.5) [7]	-1.14	0.015	1
CD14	P08571	15.2 (1.5) [12]	15.1 (1.2) [10]	-0.04	0.943	1
CD44	H0YD13	15.4 (0.9) [12]	15.3 (1.2) [9]	-0.04	0.937	1
C2orf40	B8ZZE5	15 (1.3) [9]	14.4 (1.4) [7]	-0.59	0.396	1
C3	P01024	17.8 (0.4) [12]	18.1 (0.9) [11]	0.29	0.355	1
C4A	A0A0G2JPR0	15.7 (0.6) [12]	15.7 (1) [10]	-0.03	0.936	1
C4B	P0C0L5	16.8 (0.3) [12]	17 (0.2) [11]	0.16	0.186	1
C4BPA	P04003	16.1 (1.2) [12]	16.1 (2.7) [10]	-0.02	0.979	1
C5	P01031	15.7 (0.6) [12]	15.8 (0.8) [10]	0.06	0.849	1
C6	P13671	16.2 (0.7) [12]	16.6 (0.8) [10]	0.35	0.297	1
C7	P10643	15.8 (0.7) [12]	15.9 (0.6) [10]	0.10	0.729	1
C8A	P07357	16 (0.6) [12]	16.1 (0.9) [11]	0.06	0.854	1
C8B	F5GY80	14.7 (0.6) [12]	14.8 (0.6) [10]	0.08	0.753	1
C8G	P07360	14.7 (0.4) [9]	14.6 (0.6) [7]	-0.04	0.872	1
C9	P02748	16 (0.5) [12]	16.1 (0.5) [10]	0.04	0.853	1
CA1	P00915	17 (1.2) [10]	17.2 (3.7) [9]	0.15	0.906	1
CA2	P00918	15.1 (1.2) [11]	16.3 (4.4) [8]	1.11	0.503	1
CACNA2D1	P54289	13.6 (0.7) [5]	14.4 (1.2) [6]	0.80	0.19	1
A1BG	P04217-2	17.5 (0.7) [12]	17.8 (0.6) [11]	0.32	0.256	1
A2M	P01023	18.1 (0.7) [12]	18.3 (1) [11]	0.24	0.514	1
ABHD14B	Q96IU4	13.3 (0.9) [8]	12.9 (1.2) [6]	-0.39	0.522	1
ACSBG2	Q5FVE4	20.4 (0.7) [10]	20.2 (0.7) [8]	-0.17	0.598	1
ACTA2	P62736	16.6 (0.6) [12]	16.1 (1.2) [7]	-0.54	0.29	1
ACTB	P60709	16.9 (0.7) [12]	16.3 (1.9) [11]	-0.61	0.334	1
ACTBL2	Q562R1	18.8 (0.7) [12]	17.6 (1.4) [10]	-1.20	0.027	1
ADIPOQ	Q15848	13.8 (0.9) [7]	14.2 (1.5) [7]	0.41	0.557	1
AFM	P43652	15.7 (0.5) [12]	15.8 (0.5) [10]	0.10	0.625	1
AGRN	O00468-6	13.3 (0.9) [5]	13.5 (1.4) [5]	0.15	0.842	1
AGT	P01019	16 (0.4) [12]	16.7 (0.7) [11]	0.62	0.019	1
AHSG	P02765	17.7 (0.7) [12]	18.5 (2.4) [11]	0.77	0.327	1
ALB	P02768	19.8 (0.5) [12]	20 (0.8) [11]	0.29	0.289	1
ALDOA	P04075	14.9 (0.8) [12]	15 (2.9) [10]	0.07	0.945	1
ALDOC	P09972	14.2 (1.2) [10]	14 (1) [5]	-0.21	0.736	1
AMBP	P02760	16.9 (0.7) [12]	17 (1) [11]	0.11	0.748	1
APCS	P02743	17.3 (1.4) [12]	17.9 (1.9) [10]	0.68	0.36	1

Protein Name	Uniprot ID	SAH Weaned<br>Mean (SD) [N]	SAH Shunted<br>Mean (SD) [N]	log_2FC	P-value	Adj. P-value
APLP1	B7Z4G8	14.7 (0.8) [10]	14.3 (1.5) [10]	-0.43	0.431	1
APLP2	Q06481	13.7 (1.1) [8]	14.7 (0.7) [6]	1.00	0.063	1
APOA1	P02647	19.8 (1) [12]	20.3 (1.1) [11]	0.50	0.279	1
APOA2	P02652	19 (0.8) [12]	19 (1) [11]	-0.06	0.873	1
APOA4	P06727	17.3 (0.9) [12]	17.5 (1) [10]	0.17	0.672	1
APOB	P04114	18.4 (1.5) [12]	19.2 (2.7) [11]	0.71	0.446	1
APOC1	K7ERI9	17.7 (1.5) [12]	18.6 (1.8) [10]	0.85	0.252	1
APOC3	B0YIW2	19.3 (2.1) [12]	20.2 (2.3) [10]	0.96	0.328	1
APOC4-APOC2	A0A024R0T9	17.3 (1.8) [12]	18.1 (2.4) [10]	0.80	0.391	1
APOD	C9JF17	16.5 (0.9) [12]	16.5 (0.7) [10]	-0.06	0.876	1
APOE	P02649	16.5 (0.7) [12]	16.6 (0.8) [11]	0.02	0.953	1
APOF	Q13790	16.2 (1) [7]	15.9 (0.6) [8]	-0.32	0.468	1
APOH	P02749	16.3 (0.6) [12]	16.7 (0.8) [10]	0.40	0.207	1
APOL1	O14791	16 (1.5) [12]	16.2 (1.9) [10]	0.20	0.785	1
APOM	O95445	17.5 (1.1) [12]	17.9 (1.7) [10]	0.39	0.535	1
APP	P05067	13.5 (0.8) [11]	13.3 (1.1) [9]	-0.15	0.743	1
ARPC4-TTLL3	A0A0A6YYG9	16.8 (0.5) [12]	16.8 (0.6) [10]	0.08	0.747	1
ATP6AP1	Q15904	13.8 (0.8) [9]	13.7 (1.1) [8]	-0.11	0.815	1
ATRN	O75882	14.6 (0.5) [11]	14.8 (0.5) [9]	0.18	0.46	1
AZGP1	P25311	16.8 (0.6) [12]	17.1 (0.5) [10]	0.32	0.18	1
B2M	P61769	15.3 (2.5) [12]	15.4 (1.4) [11]	0.17	0.845	1
B4GAT1	O43505	15.1 (1.1) [10]	14.4 (1.5) [8]	-0.63	0.334	1
BCAN	Q96GW7	14.7 (1) [10]	14.6 (1.6) [7]	-0.06	0.934	1
BCHE	P06276	14.9 (0.7) [6]	13.9 (1.9) [5]	-0.95	0.336	1
BTD	P43251	15.4 (0.2) [12]	15.7 (0.5) [10]	0.22	0.253	1
C1QA	P02745	15.4 (0.6) [12]	15.2 (0.4) [10]	-0.27	0.22	1
C1QB	D6R934	15.9 (0.6) [12]	15.6 (0.7) [10]	-0.30	0.296	1
C1QC	P02747	17.6 (0.5) [12]	17.5 (0.5) [10]	-0.05	0.827	1
C1R	B4DPQ0	16.1 (0.4) [12]	15.9 (0.4) [10]	-0.14	0.387	1
C1RL	Q9NZP8	14.8 (1.1) [10]	14.9 (0.7) [10]	0.06	0.882	1
C1S	P09871	16.4 (0.4) [12]	16.3 (0.4) [10]	-0.07	0.661	1
C2	P06681	15.5 (0.5) [12]	15.5 (0.5) [10]	0.00	0.99	1
GAPDH	P04406	15.2 (1.1) [12]	15 (1.4) [9]	-0.14	0.814	1
GC	P02774	17.1 (0.5) [12]	17.4 (0.8) [11]	0.35	0.23	1
GDI2	P50395	14.3 (0.7) [9]	14.3 (1.5) [6]	-0.07	0.914	1
GGH	Q92820	14.4 (0.5) [9]	14.3 (0.4) [8]	-0.09	0.668	1
GM2A	P17900	14.9 (1.1) [10]	14.3 (1.4) [9]	-0.61	0.303	1
GPLD1	P80108	15.7 (1) [11]	15.9 (1.1) [9]	0.23	0.635	1
GPX3	A0A087X1J7	16 (0.6) [12]	16 (0.6) [10]	0.05	0.852	1
GSN	P06396	16.1 (0.4) [12]	16.1 (0.5) [11]	0.06	0.747	1

Protein Name	Uniprot ID	SAH Weaned<br>Mean (SD) [N]	SAH Shunted<br>Mean (SD) [N]	log_2FC	P-value	Adj. P-value
GSTP1	P09211	15.7 (1.1) [10]	14.7 (0.8) [8]	-0.99	0.046	1
HABP2	Q14520	13.8 (0.5) [5]	14.4 (0.5) [7]	0.62	0.061	1
HBA1	P69905	20.2 (1.7) [12]	19.8 (3.4) [11]	-0.37	0.751	1
HBB	P68871	20.3 (1.9) [12]	19.9 (4) [11]	-0.45	0.737	1
HBD	P02042	17.5 (1.5) [12]	18.3 (3.2) [9]	0.75	0.529	1
HBG2	P69892	15.9 (1) [8]	16.6 (3.3) [9]	0.71	0.55	1
HEXB	P07686	13.4 (0.8) [6]	13.5 (0.9) [6]	0.06	0.903	1
HGFAC	D6RAR4	15.1 (0.5) [10]	15.2 (0.6) [10]	0.12	0.64	1
HP	P00738	18.3 (2) [12]	19.6 (2) [11]	1.25	0.152	1
HPR	P00739	16.5 (1.4) [12]	17.2 (2) [10]	0.69	0.362	1
HPX	P02790	18.3 (0.5) [12]	18.5 (0.6) [11]	0.20	0.38	1
HRG	P04196	16.8 (0.7) [12]	16.9 (0.8) [11]	0.09	0.784	1
HSP90AA1	P07900	14.9 (1) [10]	15.2 (1.1) [8]	0.33	0.518	1
HSPA5	P11021	14 (0.6) [6]	13.9 (1) [6]	-0.18	0.723	1
HSPA8	P11142	15.4 (0.8) [12]	15.5 (4) [8]	0.10	0.946	1
HSPG2	P98160	13.6 (1.5) [10]	13.4 (1.8) [8]	-0.13	0.873	1
HTRA1	Q92743	13.1 (0.7) [9]	13.8 (1) [6]	0.77	0.147	1
HYAL1	Q12794	17.7 (1.4) [8]	18.3 (1.2) [5]	0.58	0.437	1
ICOSLG	K4DIA0	13.9 (1.1) [10]	14 (1.3) [8]	0.14	0.822	1
CFD	K7ERG9	15.2 (0.7) [11]	15.5 (0.9) [9]	0.33	0.37	1
CFH	P08603	16.7 (0.5) [12]	17 (1.2) [11]	0.35	0.39	1
CFHR1	B1AKG0	15.7 (1) [12]	16.4 (0.8) [10]	0.74	0.064	1
CFI	E7ETH0	15.3 (0.4) [12]	15.5 (0.5) [10]	0.16	0.463	1
CFL1	E9PK25	15.3 (0.7) [12]	15 (1.3) [9]	-0.28	0.575	1
CHGA	P10645	14.6 (1) [10]	14.3 (1) [8]	-0.25	0.607	1
CHGB	P05060	14.1 (1) [11]	13.9 (1.4) [10]	-0.27	0.636	1
CHI3L1	P36222	15.3 (1.9) [10]	15.5 (2.1) [11]	0.17	0.851	1
CHI3L2	Q15782	14.3 (1.4) [7]	14.2 (1.8) [7]	-0.03	0.977	1
CHL1	O00533	13.6 (1.1) [11]	13.6 (1.2) [8]	-0.02	0.976	1
CKB	P12277	17.3 (1.4) [12]	15.3 (0.8) [7]	-2.00	0.001	0.41
CLEC3B	E9PHK0	15.7 (0.4) [12]	15.9 (0.6) [10]	0.16	0.509	1
CLSTN1	O94985	14.8 (0.7) [9]	14.9 (1.2) [6]	0.05	0.922	1
CLU	P10909	16.3 (0.7) [12]	16.4 (0.5) [11]	0.15	0.574	1
CNDP1	Q96KN2	15.2 (0.9) [12]	15.3 (1.1) [10]	0.07	0.875	1
CNTN1	Q12860	14.1 (0.9) [9]	13.9 (1.2) [8]	-0.22	0.681	1
CNTN2	A0A1W2PQ11	14.2 (1.2) [9]	14.1 (1.1) [8]	-0.05	0.93	1
COL18A1	P39060	14.5 (1) [7]	13.8 (1.3) [5]	-0.65	0.389	1
COL6A1	A0A087X0S5	13.6 (1.2) [9]	13.6 (1.3) [7]	-0.05	0.935	1
COL6A3	P12111	14 (0.6) [7]	13.9 (0.9) [5]	-0.09	0.854	1
CP	P00450	16.7 (0.4) [12]	17 (0.8) [11]	0.33	0.231	1

Protein Name	Uniprot ID	SAH Weaned Mean (SD) [N]	SAH Shunted Mean (SD) [N]	log_2FC	P-value	Adj. P-value
CPB2	A0A087WSY5	15.6 (0.7) [12]	15.8 (0.7) [10]	0.22	0.49	1
CPE	P16870	15 (1.4) [11]	15.1 (1.1) [10]	0.16	0.775	1
CPN1	P15169	15.1 (0.6) [11]	15.8 (0.6) [8]	0.65	0.039	1
CPN2	P22792	16.6 (0.9) [12]	16.8 (1.3) [10]	0.14	0.781	1
CPQ	Q9Y646	14.1 (0.9) [8]	14.4 (1.3) [5]	0.32	0.637	1
CPVL	Q9H3G5	14.2 (0.8) [8]	14.1 (0.8) [6]	-0.12	0.767	1
CRP	P02741	14.4 (1.2) [10]	14 (1.3) [8]	-0.44	0.462	1
CRTAC1	A0A0C4DFP6	14.2 (0.8) [10]	14.4 (1.3) [7]	0.16	0.773	1
CSF1	P09603	14.5 (1.1) [7]	13.8 (1) [7]	-0.74	0.198	1
CST3	P01034	15.8 (1.7) [12]	16 (1.5) [11]	0.20	0.77	1
CTSB	P07858	14.3 (1.2) [7]	15 (1.4) [6]	0.71	0.349	1
CTSD	A0A1B0GV23	15.7 (1.2) [11]	15.2 (1.1) [10]	-0.53	0.3	1
CTSH	A0A087X0D5	13.9 (0.8) [6]	14 (1.2) [5]	0.10	0.874	1
DAG1	Q14118	14.3 (1) [7]	14.3 (1.1) [7]	0.00	0.993	1
DBI	A0A0A0MTI5	14.7 (0.6) [10]	14.5 (0.2) [7]	-0.17	0.413	1
DKK3	F6SYF8	14.8 (1.2) [10]	14 (1.5) [10]	-0.82	0.2	1
ECM1	Q16610	14.4 (0.3) [10]	14.4 (0.5) [10]	-0.05	0.799	1
EFEMP1	A0A0U1RQV3	15.4 (1.1) [10]	15.7 (1.7) [7]	0.31	0.679	1
EIF5A	I3L397	14.3 (0.9) [10]	13.3 (1.3) [7]	-0.96	0.12	1
ENDOD1	O94919	14.4 (0.5) [8]	14.6 (0.8) [6]	0.18	0.649	1
ENO2	P09104	15.1 (0.8) [11]	15.4 (4) [7]	0.31	0.847	1
ENPP2	E7EUF1	15.7 (1.4) [12]	15.6 (0.9) [10]	-0.15	0.768	1
ERN1	O75460	17.2 (2.2) [12]	18.7 (1.4) [9]	1.50	0.076	1
F10	P00742	15.1 (0.7) [9]	15.8 (0.5) [8]	0.69	0.025	1
F12	P00748	16.9 (0.8) [12]	17.4 (0.9) [10]	0.46	0.216	1
F13B	P05160	13.3 (2.4) [9]	14.7 (0.4) [8]	1.39	0.129	1
F2	P00734	16.4 (0.6) [12]	16.9 (0.7) [10]	0.58	0.048	1
F5	A0A0A0MRJ7	14.9 (0.5) [12]	14.9 (0.4) [10]	-0.05	0.798	1
F9	P00740	14 (0.5) [11]	14.3 (0.6) [10]	0.35	0.182	1
FAM3C	Q92520	14 (0.8) [9]	13.9 (1.1) [7]	-0.06	0.903	1
FBLN1	P23142	15.6 (1.1) [12]	15.1 (0.9) [10]	-0.51	0.236	1
FCGR3A	A0A1W2PQB1	13.8 (1.3) [10]	14.6 (0.9) [7]	0.80	0.149	1
FCN3	O75636	15 (1.8) [10]	16 (0.9) [8]	1.05	0.139	1
FETUB	Q9UGM5	15.5 (1.4) [11]	15.6 (0.8) [9]	0.10	0.844	1
FGA	P02671	17.1 (1.1) [12]	16.3 (1.1) [11]	-0.78	0.097	1
FGB	P02675	18.4 (1.3) [12]	17.7 (1.6) [11]	-0.75	0.238	1

Protein Name	Uniprot ID	SAH Weaned<br>Mean (SD) [N]	SAH Shunted<br>Mean (SD) [N]	log_2FC	P-value	Adj. P-value
FGG	P02679	17.6 (1.2) [12]	16.9 (1.6) [11]	-0.73	0.242	1
FLNA	P21333	13.6 (0.7) [9]	14.3 (1.4) [6]	0.65	0.33	1
FN1	P02751	16.5 (0.5) [12]	16.8 (0.8) [11]	0.24	0.418	1
FSTL1	Q12841	13.5 (1.4) [10]	12.8 (1.5) [7]	-0.64	0.383	1
FUCA2	Q9BTY2	13.7 (1.3) [8]	13.9 (1) [7]	0.16	0.791	1
LAMP2	P13473	13.9 (1.2) [11]	13.8 (1.2) [9]	-0.10	0.859	1
LBP	P18428	14.5 (0.6) [8]	14.6 (0.7) [9]	0.17	0.614	1
LCAT	P04180	14.9 (0.7) [11]	14.7 (0.5) [10]	-0.15	0.576	1
LCP1	P13796	14.2 (0.6) [10]	14.1 (1) [5]	-0.02	0.969	1
LDHA	P00338	14.5 (0.9) [9]	14.2 (1.5) [6]	-0.28	0.696	1
LDHB	P07195	14.8 (1.1) [12]	15.7 (4) [9]	0.92	0.523	1
LGALS3BP	Q08380	15.5 (0.7) [12]	15.5 (0.8) [10]	-0.05	0.891	1
LIAS	A0A1W2PNQ5	15.6 (1.2) [8]	15.8 (1.2) [10]	0.27	0.645	1
LMAN2	D6RBV2	13.5 (0.9) [7]	14.6 (1.4) [5]	1.08	0.167	1
LRG1	P02750	16.8 (0.3) [12]	17.1 (0.4) [10]	0.25	0.153	1
LUM	P51884	16.1 (0.4) [12]	15.9 (0.6) [10]	-0.20	0.38	1
LYVE1	Q9Y5Y7	14.3 (1.6) [11]	13.8 (1.5) [9]	-0.41	0.55	1
LYZ	A0A0B4J259	15.9 (1.4) [12]	15.5 (1.3) [10]	-0.35	0.557	1
MAN1A1	P33908	14 (0.7) [6]	13.5 (1.3) [6]	-0.52	0.414	1
MASP1	P48740	13.3 (0.5) [7]	14 (0.4) [7]	0.73	0.017	1
MBL2	P11226	14.2 (1.4) [7]	14.1 (0.5) [7]	-0.04	0.942	1
MMP2	P08253	14.2 (1.9) [6]	14 (1) [7]	-0.23	0.794	1
MST1	G3XAK1	14 (0.9) [6]	14.1 (1.2) [5]	0.06	0.931	1
NBL1	A0A087WTY6	17.4 (1.1) [6]	17.1 (1.6) [5]	-0.33	0.71	1
NCAM1	P13591	14.3 (0.9) [11]	13.8 (1.5) [9]	-0.57	0.341	1
NEGR1	Q7Z3B1	13.5 (1.4) [9]	13.7 (1.6) [5]	0.24	0.786	1
NEO1	Q92859	13.9 (1.1) [7]	13.7 (1.1) [5]	-0.14	0.842	1
NPC2	E7EMS2	15.6 (0.9) [9]	15 (1.3) [7]	-0.61	0.306	1
NPTX1	Q15818	13.9 (1.7) [8]	13.4 (1.7) [8]	-0.52	0.551	1
NRCAM	C9JYY6	14 (0.9) [9]	14.1 (1.2) [7]	0.07	0.897	1
NRXN3	A0A0U1RQC5	13.1 (0.8) [5]	13 (1.2) [6]	-0.11	0.86	1
NTM	Q9P121-4	13.8 (0.7) [6]	13.6 (1.3) [5]	-0.20	0.773	1
NUCB1	Q02818	13.5 (0.7) [10]	12.9 (1.6) [6]	-0.58	0.431	1
OGN	P20774	14.4 (1.9) [11]	14.1 (1.6) [8]	-0.35	0.665	1
ORM1	P02763	19.2 (0.4) [12]	19.7 (0.6) [11]	0.47	0.042	1
ORM2	P19652	17.8 (0.5) [12]	17.9 (0.4) [11]	0.11	0.57	1
PAM	P19021	13.7 (0.8) [7]	14 (1.1) [6]	0.22	0.687	1
PCOLCE	Q15113	15.4 (1.1) [9]	14.7 (1.4) [7]	-0.76	0.256	1
PCSK1N	Q9UHG2	14.1 (1.2) [11]	13.9 (1.3) [10]	-0.22	0.695	1

**Protein Name**	**Uniprot ID**	**SAH Weaned** Mean (SD) [N]	**SAH Shunted** Mean (SD) [N]	**log$_2$FC**	**P-value**	**Adj. P-value**
PCSK9	Q8NBP7	12.1 (0.8) [5]	12.3 (0.4) [5]	0.20	0.645	1
PEBP1	P30086	16 (1) [12]	15 (0.9) [7]	-1.00	0.036	1
PEBP4	Q96S96	14.4 (0.8) [7]	15.2 (1.3) [5]	0.80	0.27	1
PENK	P01210	13.9 (0.8) [6]	14.5 (0.9) [5]	0.56	0.313	1
PEPD	P12955	12.6 (0.6) [8]	12.7 (0.6) [9]	0.09	0.758	1
PF4	P02776	15.3 (1.1) [5]	15.3 (0.7) [6]	-0.01	0.988	1
PFN1	P07737	15.8 (0.5) [12]	16.4 (2.2) [8]	0.61	0.471	1
PGK1	P00558	15.5 (0.7) [10]	16.5 (4.6) [7]	0.97	0.6	1
PGLYRP2	Q96PD5	15.4 (0.7) [12]	15.7 (0.9) [11]	0.27	0.41	1
PLG	P00747	17.1 (0.7) [12]	17.5 (1.1) [11]	0.47	0.257	1
PLTP	P55058	15 (1) [11]	14.9 (1.3) [11]	-0.10	0.85	1
PLXDC2	Q6UX71	13.5 (1.1) [10]	13.4 (1.3) [8]	-0.06	0.916	1
PMFBP1	G3V1Q7	15.5 (0.8) [5]	16 (0.8) [6]	0.50	0.328	1
PON1	P27169	17.4 (1) [12]	17.6 (1.4) [10]	0.20	0.711	1
PON3	C9JZ99	13.1 (1) [6]	14.4 (0.5) [6]	1.32	0.024	1
PPBP	P02775	18.5 (1) [11]	18.3 (1.8) [9]	-0.18	0.791	1
PPIA	P62937	16.4 (0.7) [12]	15 (1.3) [10]	-1.44	0.008	1
PPIB	P23284	14.3 (0.6) [8]	14.4 (1.1) [8]	0.12	0.79	1
PRDX1	Q06830	16.8 (1) [12]	16.2 (3.4) [10]	-0.68	0.551	1
PRDX2	P32119	16.7 (1.5) [12]	16.6 (4.1) [11]	-0.09	0.947	1
PRDX6	P30041	16.1 (0.7) [11]	15.6 (2.1) [9]	-0.52	0.491	1
PRG4	A0A0U1RR20	13.9 (0.9) [10]	13.3 (0.6) [10]	-0.54	0.142	1
PROC	E7END6	14 (0.5) [10]	13.9 (0.8) [10]	-0.01	0.977	1
PROCR	Q9UNN8	14 (0.9) [10]	14 (0.5) [9]	0.01	0.981	1
PROS1	P07225	15.8 (0.4) [12]	15.9 (0.6) [10]	0.14	0.517	1
PROZ	P22891	14.9 (1) [9]	15.3 (0.8) [8]	0.46	0.317	1
PTGDS	P41222	18.1 (2.2) [12]	18.1 (1.5) [11]	-0.04	0.957	1
PTPRG	P23470	13.8 (0.6) [7]	14.2 (1.2) [6]	0.37	0.53	1
PTPRZ1	P23471	13.8 (0.8) [9]	13.8 (1.4) [5]	0.06	0.936	1
PZP	P20742	14.7 (0.8) [9]	14.8 (0.8) [8]	0.08	0.844	1
QSOX1	O00391	14 (0.5) [10]	14 (0.3) [9]	0.04	0.855	1
RARRES2	Q99969	14.3 (0.8) [8]	14.4 (1.3) [6]	0.05	0.929	1
RBP4	P02753	16 (0.5) [12]	16.8 (1) [11]	0.74	0.047	1
S100A8	P05109	16.3 (1.3) [12]	15.3 (2.7) [8]	-1.00	0.355	1
S100B	P04271	17 (1) [11]	15.2 (0.8) [9]	-1.77	<0.001	0.096
SAA1	P0DJI8	16.1 (1.1) [10]	16.5 (1.1) [9]	0.43	0.397	1
SAA4	P35542	16.6 (0.9) [12]	16.8 (1.3) [10]	0.24	0.623	1
SCG2	P13521	12.8 (0.5) [9]	12.7 (0.9) [9]	-0.09	0.813	1
SCG3	Q8WXD2	14 (1.1) [9]	14.1 (1.6) [7]	0.10	0.886	1
SCG5	P05408-2	14.8 (0.8) [9]	14.4 (1.3) [7]	-0.49	0.417	1
SCRG1	O75711	16.1 (1.7) [6]	15.7 (1.4) [6]	-0.35	0.705	1

**Protein Name**	**Uniprot ID**	**SAH Weaned** Mean (SD) [N]	**SAH Shunted** Mean (SD) [N]	**log$_2$FC**	**P-value**	**Adj. P-value**
SELENOP	A0A182DWH7	15.7 (0.4) [8]	16 (0.3) [9]	0.31	0.119	1
SELL	P14151	15.9 (0.6) [10]	16.1 (0.6) [9]	0.18	0.516	1
SERPINA1	P01009	18.6 (0.6) [12]	18.9 (0.7) [11]	0.32	0.242	1
SERPINA10	G3V2W1	13.4 (1) [10]	14.2 (0.9) [8]	0.84	0.068	1
SERPINA3	P01011	17.4 (0.6) [12]	17.6 (0.8) [11]	0.20	0.518	1
SERPINA4	P29622	15.6 (0.5) [12]	15.8 (0.4) [10]	0.13	0.504	1
SERPINA5	P05154	14.5 (0.4) [11]	14.7 (0.6) [10]	0.18	0.461	1
SERPINA6	P08185	15.8 (0.6) [12]	16.2 (0.8) [11]	0.36	0.229	1
SERPINA7	P05543	14.9 (0.6) [12]	14.9 (0.4) [10]	-0.02	0.927	1
SERPINC1	P01008	16.8 (0.4) [12]	17.2 (0.8) [11]	0.35	0.205	1
SERPIND1	P05546	16.1 (0.5) [12]	16.3 (0.7) [11]	0.19	0.466	1
SERPINF1	P36955	16.7 (1.1) [12]	16.5 (1) [11]	-0.16	0.709	1
SERPINF2	P08697	16.7 (0.3) [12]	16.9 (0.8) [11]	0.22	0.385	1
SERPING1	P05155	16.9 (0.3) [12]	17.1 (0.3) [11]	0.22	0.102	1
SERPINI1	Q99574	13.2 (0.6) [6]	13.4 (1.4) [5]	0.13	0.85	1
SHBG	I3L145	14.6 (1.3) [10]	15 (1.2) [9]	0.33	0.575	1
SIRPA	P78324	13.7 (0.6) [6]	14 (0.8) [5]	0.29	0.52	1
SKP1	E5RJR5	14.3 (0.6) [9]	13.8 (0.7) [7]	-0.52	0.13	1
SOD1	P00441	15.1 (0.7) [11]	16.1 (2.7) [7]	1.02	0.363	1
SOD3	P08294	14.6 (1.3) [12]	14.6 (0.9) [10]	-0.06	0.894	1
SPARC	P09486	14.7 (0.6) [12]	14.7 (1) [10]	0.00	0.999	1
SPARCL1	Q14515	13.6 (0.6) [11]	13.4 (1.1) [10]	-0.23	0.559	1
SPP1	P10451	14.2 (1.9) [12]	14.9 (2.8) [11]	0.76	0.456	1
SYNE3	G3V533	15.5 (0.7) [7]	15.9 (1) [8]	0.47	0.305	1
SYT2	Q8N9I0	18.2 (1.1) [7]	18.7 (0.8) [5]	0.44	0.423	1
TAGLN2	P37802	14.5 (1.1) [9]	14.8 (1) [7]	0.31	0.558	1
TF	P02787	17.9 (0.4) [12]	18 (0.6) [11]	0.16	0.434	1
TFRC	G3V0E5	12 (0.7) [8]	12.8 (0.4) [5]	0.80	0.023	1
TGFBI	Q15582	14.5 (0.9) [12]	15 (2.1) [11]	0.49	0.499	1
THBS1	P07996	15.2 (0.5) [10]	15.6 (0.6) [9]	0.38	0.159	1
THY1	E9PIM6	15.1 (0.9) [10]	14.8 (1.5) [9]	-0.32	0.573	1
TIMP1	P01033	18.1 (2.2) [9]	16 (2.3) [9]	-2.09	0.066	1
TNXB	A0A140TA41	13.2 (0.2) [5]	13.3 (1) [6]	0.17	0.703	1
TPI1	P60174	14.5 (0.9) [12]	14.6 (1.9) [9]	0.11	0.867	1
TPM4	P67936	15.2 (0.7) [9]	14.9 (0.7) [6]	-0.37	0.331	1
TPP1	O14773	14.5 (0.4) [8]	14.4 (0.5) [5]	-0.04	0.904	1
TPP2	P29144	15 (1.2) [10]	15.2 (1.1) [10]	0.23	0.655	1
TREM2	Q9NZC2	13.6 (0.9) [5]	13.1 (0.8) [7]	-0.44	0.404	1
TTR	P02766	18.6 (0.7) [12]	18.4 (0.8) [11]	-0.17	0.574	1
TUBA1B	P68363	17.3 (1.4) [12]	15.5 (0.9) [9]	-1.86	0.002	0.842
TUBB4B	P68371	16.8 (1.3) [12]	15.3 (1) [10]	-1.41	0.009	1

Protein Name	Uniprot ID	SAH Weaned<br>Mean (SD) [N]	SAH Shunted<br>Mean (SD) [N]	log_2FC	P-value	Adj. P-value
TXN	P10599	16.2 (0.9) [9]	16.4 (1.3) [5]	0.29	0.68	1
UBC	F5H265	15.2 (0.7) [10]	14.5 (1.4) [9]	-0.68	0.202	1
VASN	Q6EMK4	14.1 (0.3) [9]	14.4 (0.6) [7]	0.33	0.199	1
VCAM1	P19320	13.5 (0.6) [6]	13.3 (1) [6]	-0.20	0.697	1
VCAN	P13611	14.4 (0.7) [7]	14.3 (1.4) [5]	-0.04	0.954	1
VGF	O15240	13.1 (0.8) [8]	13.5 (1) [7]	0.35	0.474	1
VIM	P08670	15.4 (0.8) [11]	14.3 (1.9) [8]	-1.14	0.139	1
VIP	P01282	18.2 (0.5) [11]	18.5 (0.7) [10]	0.32	0.25	1
VTN	P04004	17.9 (0.8) [12]	18.5 (0.8) [10]	0.57	0.108	1
VWF	P04275	14.1 (1) [8]	14.7 (1) [9]	0.61	0.225	1
WFIKKN2	C9J6G4	14.8 (0.9) [9]	14.7 (1.2) [6]	-0.14	0.815	1
YWHAE	P62258	15 (0.9) [9]	16.2 (3.5) [5]	1.15	0.507	1
YWHAZ	P63104	14.8 (0.8) [12]	13.7 (1.4) [8]	-1.12	0.063	1
IGKV1-16	P04430	14.7 (0.8) [9]	14.8 (0.8) [8]	0.17	0.68	1
IGKV1-17	P01599	16.6 (0.7) [12]	16.4 (0.9) [10]	-0.24	0.497	1
IGKV1-27	A0A075B6S5	16 (0.9) [9]	16.2 (0.8) [9]	0.18	0.67	1
IGKV1-5	P01602	16.7 (0.7) [12]	17 (0.6) [10]	0.23	0.419	1
IGKV1-8	A0A0C4DH67	17.3 (1) [12]	17.4 (1.2) [10]	0.10	0.839	1
IGKV1D-33	P01593	18.4 (1) [12]	18.2 (1.1) [10]	-0.16	0.724	1
IGKV1D-37	A0A075B6S9	17.9 (1.1) [11]	17.9 (1.3) [7]	0.05	0.938	1
IGKV2-28	A0A075B6P5	16.7 (0.9) [11]	16.9 (0.9) [10]	0.19	0.637	1
IGKV2-29	A2NJV5	17.2 (0.9) [12]	17.1 (1.1) [10]	-0.12	0.776	1
IGKV2-40	A0A087WW87	14.2 (0.8) [6]	14.2 (1.2) [5]	0.00	0.998	1
IGKV2D-24	A0A075B6R9	17.4 (0.9) [12]	17.6 (1.2) [10]	0.23	0.637	1
IGKV2D-29	A0A075B6S2	14.4 (0.8) [7]	14.7 (0.7) [5]	0.40	0.378	1
IGKV3-15	P01624	17.7 (0.5) [12]	17.6 (0.7) [10]	-0.12	0.645	1
IGKV3-20	P01619	19.2 (0.5) [12]	19.3 (1.2) [11]	0.17	0.668	1
IGKV3-7	A0A075B6H7	18.3 (1.2) [10]	18.8 (1.7) [10]	0.51	0.456	1
IGKV3D-11	A0A0A0MRZ8	18.1 (0.6) [12]	18 (0.4) [10]	-0.07	0.754	1
IGKV3D-15	A0A087WSY6	18.2 (0.8) [12]	18.2 (1) [10]	0.04	0.916	1
IGKV3D-20	A0A0C4DH25	19.4 (1.1) [12]	19.4 (1.2) [10]	-0.01	0.99	1
IGKV4-1	P06312	18 (0.6) [12]	17.7 (0.9) [10]	-0.25	0.467	1
IGLC3	P0DOY3	20.9 (0.4) [12]	20.7 (1) [10]	-0.27	0.443	1
IGLL1	P15814	16.6 (1.1) [10]	17 (0.7) [7]	0.37	0.39	1

**Protein Name**	**Uniprot ID**	**SAH Weaned**<br>Mean (SD) [N]	**SAH Shunted**<br>Mean (SD) [N]	**log$_2$FC**	**P-value**	**Adj. P-value**
IGLL5	A0A0B4J231	18.9 (0.6) [12]	18.8 (1.1) [11]	-0.16	0.658	1
IGLV1-47	P01700	17.2 (0.8) [12]	16.9 (0.7) [10]	-0.24	0.478	1
IGLV1-51	P01701	16.6 (0.8) [12]	16.8 (0.9) [9]	0.19	0.626	1
IGLV3-10	A0A075B6K4	16.3 (0.7) [9]	16.3 (1.2) [7]	-0.02	0.974	1
IGLV3-19	P01714	15.1 (0.4) [12]	15 (0.5) [9]	-0.04	0.859	1
IGLV3-21	P80748	15.8 (1.1) [11]	16.5 (1.1) [9]	0.76	0.135	1
IGLV3-25	P01717	15.9 (0.7) [11]	15.7 (1.2) [10]	-0.24	0.593	1
IGLV3-9	A0A075B6K5	16.3 (1.2) [12]	16.7 (1.5) [10]	0.45	0.449	1
IGLV6-57	P01721	15.4 (0.7) [12]	15.5 (1) [10]	0.17	0.655	1
IGLV7-46	A0A075B6I9	16.2 (0.5) [12]	16.2 (1) [10]	0.04	0.897	1
IGLV8-61	A0A075B6I0	16 (0.8) [8]	17.1 (0.8) [8]	1.05	0.016	1
IGSF8	Q969P0	14.5 (1) [9]	14.6 (1.3) [5]	0.10	0.892	1
ISLR	O14498	14.1 (1.4) [9]	13.7 (1.2) [7]	-0.38	0.569	1
ITIH1	P19827	17 (0.7) [12]	17.8 (2.1) [11]	0.82	0.243	1
ITIH2	P19823	17 (0.9) [12]	17.3 (1) [11]	0.23	0.572	1
ITIH3	Q06033	15.6 (0.8) [12]	15.6 (0.8) [10]	-0.03	0.929	1
ITIH4	Q14624	17.5 (0.8) [12]	17.5 (0.8) [11]	0.04	0.9	1
ITPR2	Q14571	19.1 (0.8) [12]	18.6 (1) [10]	-0.50	0.216	1
JCHAIN	D6RD17	18.2 (1.1) [12]	18.3 (1.9) [10]	0.09	0.902	1
KLK6	Q92876	15.2 (1) [11]	14.7 (1.4) [10]	-0.48	0.377	1
KLKB1	H0YAC1	15.6 (0.8) [12]	15.9 (1.1) [10]	0.28	0.527	1
KNG1	P01042	17.6 (0.7) [12]	18 (0.8) [10]	0.37	0.273	1
KRT1	P04264	15.3 (1) [8]	15.6 (5) [8]	0.30	0.869	1
IGHG2	P01859	20.4 (0.5) [12]	20.2 (0.7) [11]	-0.18	0.477	1
IGHG3	P01860	17.7 (1) [12]	17.9 (1.1) [10]	0.20	0.648	1
IGHG4	A0A286YFJ8	16.9 (1.1) [12]	16.5 (0.7) [11]	-0.40	0.315	1
IGHM	A0A1B0GUU9	19.1 (1.4) [12]	19.5 (2.3) [10]	0.36	0.676	1
IGHV1-18	A0A0C4DH31	14.4 (1) [8]	15.3 (0.9) [7]	0.91	0.082	1
IGHV1-2	P23083	16.2 (0.6) [11]	16.4 (0.5) [8]	0.18	0.47	1
IGHV1OR15-1	A0A075B7D0	17.5 (1.6) [11]	17.3 (1.5) [10]	-0.14	0.832	1
IGHV2-26	A0A0B4J1V2	14.2 (1.1) [10]	14 (1.1) [9]	-0.15	0.771	1
IGHV2-5	P01817	13.2 (1.2) [8]	13.1 (1.2) [5]	-0.14	0.839	1
IGHV3-15	A0A0B4J1V0	16.3 (0.7) [12]	16 (0.5) [10]	-0.32	0.213	1
IGHV3-30	P01768	17.6 (0.6) [12]	17.3 (0.5) [10]	-0.32	0.189	1

Protein Name	Uniprot ID	SAH Weaned<br>Mean (SD) [N]	SAH Shunted<br>Mean (SD) [N]	log_2FC	P-value	Adj. P-value
IGHV3-38	A0A0C4DH36	15.5 (0.6) [12]	15.6 (0.6) [10]	0.12	0.623	1
IGHV3-49	A0A0A0MS15	18 (0.7) [12]	17.9 (1) [10]	-0.11	0.776	1
IGHV3-64D	A0A0J9YX35	16 (0.7) [12]	15.9 (0.7) [10]	-0.09	0.76	1
IGHV3-7	P01780	17.9 (0.7) [12]	17.7 (0.6) [10]	-0.14	0.625	1
IGHV3-72	A0A0B4J1Y9	17.2 (0.8) [12]	17.3 (0.8) [10]	0.11	0.739	1
IGHV3OR16-9	A0A0B4J2B5	20.5 (0.5) [12]	20.3 (0.8) [11]	-0.20	0.469	1
IGHV4-34	P06331	16 (0.9) [12]	15.8 (1) [10]	-0.20	0.64	1
IGHV5-51	A0A0C4DH38	16.5 (0.6) [12]	16.4 (1) [10]	-0.05	0.888	1
IGKC	P01834	20.9 (0.8) [12]	21.2 (1.8) [11]	0.27	0.646	1
IGKV1-12	A0A0C4DH73	18 (1.1) [12]	17.7 (1) [10]	-0.30	0.515	1
IGHG1	P01857	20.5 (0.5) [12]	20.3 (0.8) [11]	-0.20	0.494	1
IGFBP7	Q16270	15.6 (1.4) [12]	16 (1.8) [11]	0.34	0.61	1
IGFALS	P35858	15.8 (0.7) [12]	16.3 (0.9) [10]	0.47	0.204	1
IGFBP3	A6XND0	13.9 (1.1) [5]	13.6 (0.7) [6]	-0.35	0.553	1
IGFBP6	P24592	16.1 (1.7) [11]	15.9 (1.5) [9]	-0.24	0.741	1
IGHA1	A0A286YEY1	19.2 (0.9) [12]	19.2 (1.3) [11]	-0.03	0.952	1
IGHA2	A0A286YEY5	19.2 (1) [12]	19.5 (1.3) [11]	0.26	0.604	1
IGHD	A0A0A0MS09	16.2 (1.4) [12]	16.2 (1.4) [8]	0.06	0.922	1
CAT	P04040	14.1 (1.2) [9]	16.1 (3.6) [5]	1.98	0.29	1
GFAP	A0A1W2PR46	18 (2.3) [11]	16.8 (2.5) [6]	-1.17	0.366	1
GPR37L1	O60883	13.9 (1.3) [10]	13 (1) [7]	-0.85	0.144	1
HIST1H4A	P62805	15.3 (1.8) [8]	16.2 (3.8) [5]	0.92	0.635	1
HSPA1B	A0A0G2JIW1	15 (0.9) [10]	13.7 (1.5) [5]	-1.28	0.126	1
IGKV6D-21	A0A0A0MT36	14.7 (1.4) [7]	15.1 (0.9) [6]	0.42	0.517	1
MARCKS	P29966	12.1 (0.9) [9]	12.2 (1) [5]	0.10	0.86	1
PGAM1	P18669	14.9 (0.8) [10]	15.1 (1.6) [5]	0.21	0.79	1
PI16	Q6UXB8	13.2 (0.7) [9]	13.1 (0.6) [9]	-0.10	0.744	1
PRSS3	B1AN99	18.7 (2) [8]	18.6 (1.5) [6]	-0.09	0.926	1
S100A9	P06702	15.9 (0.7) [11]	17.3 (3.1) [6]	1.47	0.305	1
YWHAQ	P27348	13.9 (1) [9]	13.4 (0.6) [5]	-0.58	0.194	1
DBH	P09172	13.5 (1.2) [9]	14.3 (1.3) [6]	0.73	0.298	1
ENO1	P06733	16.3 (0.7) [12]	16.8 (3.1) [6]	0.50	0.716	1

**Protein Name**	**Uniprot ID**	**SAH Weaned** Mean (SD) [N]	**SAH Shunted** Mean (SD) [N]	**log_2FC**	**P-value**	**Adj. P-value**
FCGBP	Q9Y6R7	15.2 (1.7) [7]	14.3 (1.5) [5]	-0.92	0.35	1
IGHV1-69	P01742	15.1 (1.7) [8]	15.5 (0.7) [5]	0.32	0.655	1
IGHV3OR16-12	A0A075B7B8	14.2 (0.7) [10]	13.9 (0.8) [6]	-0.21	0.6	1
THBS2	P35442	15.2 (1.1) [8]	15 (0.9) [5]	-0.18	0.748	1

SAH: subarachnoid hemorrhage; SD: standard deviation; N: number of patients; log_2FC: log_2(fold change).

Supplementary Table 4: Other CSF proteins in end samples from weaned versus shunted SAH patients.

Protein Name	Uniprot ID	SAH Weaned Mean (SD) [N]	SAH Shunted Mean (SD) [N]	log_2FC	P-value	Adj. P-value
CALR	P27797	13.8 (1) [12]	13.6 (0.3) [9]	-0.28	0.391	1
CDH13	P55290	14.6 (0.5) [12]	14.7 (1.2) [10]	0.08	0.841	1
CDH2	P19022	15.7 (0.4) [11]	15.4 (0.4) [9]	-0.34	0.081	1
CDH4	P55283	12.8 (0.6) [7]	12.3 (0.3) [6]	-0.49	0.097	1
CDH6	D6RF86	13.3 (0.8) [8]	12.8 (0.4) [8]	-0.50	0.122	1
CARTPT	Q16568	13.3 (0.3) [8]	12.9 (0.8) [7]	-0.39	0.255	1
CASP14	P31944	14.4 (0.9) [9]	14.1 (2.1) [7]	-0.37	0.681	1
CBR1	P16152	14.5 (1) [9]	14.1 (0.7) [9]	-0.30	0.474	1
CD14	P08571	16.9 (1.2) [12]	17.1 (0.8) [9]	0.23	0.592	1
CD163	C9JHR8	15.9 (1.5) [11]	14.8 (1.5) [9]	-1.04	0.136	1
CD44	H0YD13	16.2 (1.2) [10]	16.3 (0.5) [9]	0.08	0.848	1
CD59	E9PNW4	15.2 (0.8) [11]	14.6 (0.8) [7]	-0.59	0.158	1
CD99L2	Q8TCZ2	16 (0.5) [11]	16 (0.6) [9]	0.04	0.877	1
C2orf40	B8ZZE5	15.9 (1.3) [11]	15 (0.5) [9]	-0.86	0.06	1
C3	P01024	17.5 (0.3) [12]	17.8 (0.7) [10]	0.34	0.191	1
C4A	A0A0G2JPR0	16.2 (0.4) [12]	15.5 (0.8) [10]	-0.62	0.057	1
C4B	P0C0L5	17.1 (0.3) [12]	17 (0.5) [10]	-0.04	0.82	1
C4BPA	P04003	13.6 (1.6) [10]	13.8 (0.8) [9]	0.20	0.721	1
C5	P01031	14.7 (0.5) [12]	15 (0.3) [9]	0.23	0.198	1
C6	P13671	15.3 (0.6) [12]	15.2 (0.5) [9]	-0.08	0.729	1
C7	P10643	16.4 (0.8) [12]	16.6 (0.6) [10]	0.16	0.58	1
C8A	P07357	15.2 (0.4) [12]	15.4 (0.7) [10]	0.22	0.411	1
C8B	F5GY80	14 (0.4) [11]	14.1 (0.3) [9]	0.11	0.518	1
C8G	P07360	14.2 (0.5) [10]	13.6 (0.4) [6]	-0.55	0.027	1
C9	P02748	15.6 (0.5) [12]	16 (0.9) [10]	0.40	0.245	1
CA1	P00915	18.5 (1.4) [11]	18.2 (2.1) [10]	-0.35	0.655	1
CA2	P00918	15.3 (1.6) [10]	15.3 (3) [9]	0.03	0.981	1
CACNA2D1	P54289	13.9 (0.7) [9]	13.1 (1) [9]	-0.73	0.087	1
CADM3	Q8N126	15.5 (1.2) [11]	14.9 (0.4) [9]	-0.59	0.14	1
CADM4	Q8NFZ8	15.8 (0.5) [11]	15.1 (0.2) [9]	-0.68	0.001	0.526
A1BG	P04217-2	16.9 (0.4) [12]	17.2 (0.4) [10]	0.26	0.15	1
A2M	P01023	17.1 (0.4) [12]	17.4 (0.7) [10]	0.29	0.275	1
ABHD14B	Q96IU4	13.8 (0.8) [9]	13.3 (0.9) [9]	-0.46	0.269	1
ACSBG2	Q5FVE4	21 (0.8) [12]	20.9 (0.5) [9]	-0.12	0.689	1
ACTA2	P62736	17.3 (1.4) [12]	17 (0.5) [9]	-0.36	0.423	1
ACTB	P60709	17.6 (1.3) [12]	17.3 (0.9) [10]	-0.28	0.569	1
ACTBL2	Q562R1	19.5 (1.4) [12]	19.1 (0.7) [9]	-0.40	0.405	1
ADA2	B4E3Q4	13.8 (0.7) [9]	13.3 (0.6) [7]	-0.45	0.18	1
ADAM29	Q9UKF5	15 (0.8) [6]	15.3 (0.9) [9]	0.31	0.499	1

**Protein Name**	**Uniprot ID**	**SAH Weaned** Mean (SD) [N]	**SAH Shunted** Mean (SD) [N]	**log_2FC**	**P-value**	**Adj. P-value**
ADIPOQ	Q15848	11.6 (0.5) [8]	12.2 (0.7) [8]	0.65	0.046	1
AEBP1	Q8IUX7	13.5 (0.7) [8]	13.4 (0.6) [8]	-0.05	0.885	1
AFM	P43652	15.3 (0.6) [12]	15.7 (1.3) [10]	0.38	0.391	1
AGRN	O00468-6	14.2 (0.4) [11]	13.2 (0.9) [8]	-0.99	0.022	1
AGT	P01019	16.5 (0.4) [12]	16.4 (0.3) [10]	-0.05	0.726	1
AHSG	P02765	16.9 (0.6) [12]	17.6 (2) [10]	0.73	0.28	1
ALB	P02768	19.3 (0.5) [12]	19.5 (0.6) [10]	0.22	0.358	1
ALCAM	Q13740	14.7 (0.6) [11]	14.3 (0.4) [9]	-0.38	0.102	1
ALDOA	P04075	15.6 (0.7) [12]	15.8 (1.1) [10]	0.19	0.66	1
ALDOC	P09972	14.5 (0.5) [12]	14.7 (0.7) [9]	0.22	0.409	1
AMBP	P02760	16.1 (0.6) [12]	15.9 (0.4) [10]	-0.13	0.569	1
ANXA5	P08758	15.1 (1.3) [11]	14.1 (1) [7]	-0.93	0.108	1
APCS	P02743	15.3 (1.6) [11]	15 (0.7) [9]	-0.31	0.579	1
APLP1	B7Z4G8	15.7 (0.7) [12]	15.4 (0.7) [9]	-0.30	0.357	1
APLP2	Q06481	14.4 (1) [12]	14.3 (0.9) [8]	-0.11	0.799	1
APOA1	P02647	18.2 (0.9) [12]	18.9 (1.1) [10]	0.70	0.128	1
APOA2	P02652	17.6 (1.4) [12]	17.6 (0.5) [10]	0.04	0.934	1
APOA4	P06727	16.1 (0.8) [12]	16.4 (1.2) [10]	0.30	0.508	1
APOB	P04114	15.8 (1.6) [12]	16.2 (0.8) [9]	0.42	0.453	1
APOC1	K7ERI9	15.9 (1.2) [12]	15.8 (0.5) [9]	-0.10	0.796	1
APOC3	B0YIW2	17.1 (1.8) [12]	17.3 (1.3) [9]	0.21	0.758	1
APOC4-APOC2	A0A024R0T9	15.2 (1.7) [12]	15.2 (1) [9]	0.05	0.929	1
APOD	C9JF17	17.2 (0.7) [12]	17.8 (1.7) [10]	0.61	0.312	1
APOE	P02649	17.6 (0.6) [12]	17.7 (0.5) [10]	0.06	0.794	1
APOH	P02749	15.9 (0.7) [12]	15.6 (0.7) [9]	-0.28	0.363	1
APOL1	O14791	14.2 (1.8) [11]	14.4 (0.5) [9]	0.19	0.744	1
APOM	O95445	15.6 (1.4) [10]	15.8 (0.7) [9]	0.27	0.599	1
APP	P05067	14.1 (0.9) [12]	13.7 (0.8) [9]	-0.36	0.331	1
ARPC4-TTLL3	A0A0A6YYG9	16.4 (0.4) [12]	16.3 (0.3) [9]	-0.12	0.473	1
ART3	E7ESB3	14.1 (0.5) [8]	13.6 (0.4) [5]	-0.52	0.065	1
ATP6AP1	Q15904	14.3 (0.9) [12]	14.1 (0.6) [9]	-0.27	0.425	1
ATRN	O75882	14.1 (0.5) [9]	13.7 (0.7) [6]	-0.37	0.309	1
AZGP1	P25311	16.9 (0.7) [12]	17.2 (0.7) [10]	0.34	0.26	1
B2M	P61769	16.9 (1.2) [12]	17.1 (0.7) [10]	0.13	0.752	1
B4GALT1	P15291	14.2 (0.7) [6]	14.8 (0.3) [7]	0.59	0.097	1
B4GAT1	O43505	16 (0.8) [12]	16.2 (2.3) [10]	0.19	0.811	1
BCAN	Q96GW7	14.8 (1.3) [12]	14.6 (0.9) [9]	-0.27	0.588	1
BGN	P21810	13.9 (0.8) [9]	13.4 (1) [7]	-0.53	0.296	1
BLVRB	P30043	16.3 (1.5) [11]	15.5 (1.7) [10]	-0.83	0.253	1
BTD	P43251	15.5 (0.6) [12]	15.3 (0.2) [9]	-0.21	0.262	1

Protein Name	Uniprot ID	SAH Weaned Mean (SD) [N]	SAH Shunted Mean (SD) [N]	log_2FC	P-value	Adj. P-value
C16orf89	A0A0A0MT71	13.8 (0.5) [9]	13.9 (0.4) [9]	0.04	0.876	1
C1QA	P02745	15.4 (0.7) [12]	15.7 (0.6) [10]	0.31	0.28	1
C1QB	D6R934	16.3 (1.1) [12]	16.4 (1) [10]	0.15	0.742	1
C1QC	P02747	17.7 (0.8) [12]	17.8 (0.6) [9]	0.06	0.841	1
C1R	B4DPQ0	16 (0.5) [12]	16.1 (0.4) [10]	0.12	0.535	1
C1RL	Q9NZP8	13.9 (0.6) [9]	14.2 (0.5) [8]	0.22	0.443	1
C1S	P09871	16.4 (0.5) [12]	16.5 (0.4) [10]	0.08	0.694	1
C2	P06681	15.5 (0.4) [12]	15.5 (0.6) [9]	-0.01	0.961	1
GALNT2	Q10471	13.8 (0.4) [11]	13.1 (0.3) [6]	-0.70	0.001	0.478
GAPDH	P04406	15.4 (1.1) [12]	15.4 (1) [9]	0.06	0.896	1
GC	P02774	16.8 (0.4) [12]	17.2 (0.9) [10]	0.45	0.155	1
GDI2	P50395	14.5 (1.2) [12]	14 (0.5) [9]	-0.46	0.248	1
GGH	Q92820	14.1 (0.5) [11]	13.4 (0.9) [5]	-0.76	0.145	1
GM2A	P17900	15.8 (0.9) [12]	15.5 (0.4) [9]	-0.27	0.353	1
GNPTG	Q9UJJ9	15.6 (0.6) [11]	15.2 (0.4) [8]	-0.41	0.074	1
GOT1	P17174	15.2 (0.7) [12]	14.9 (0.7) [9]	-0.36	0.258	1
GPI	A0A0A0MTS2	14 (0.9) [10]	13.3 (0.6) [9]	-0.69	0.06	1
GPLD1	P80108	14.8 (1.1) [8]	14 (0.7) [7]	-0.82	0.108	1
GPR37	O15354	15.2 (0.4) [10]	14.9 (0.4) [9]	-0.31	0.107	1
GPX3	A0A087X1J7	16.1 (0.7) [12]	15.7 (0.2) [9]	-0.48	0.049	1
GSN	P06396	16.3 (0.4) [12]	16.5 (0.7) [10]	0.16	0.514	1
GSTP1	P09211	15.8 (0.9) [12]	15.4 (0.9) [9]	-0.36	0.357	1
HBA1	P69905	20.4 (2.8) [12]	21.5 (1.7) [10]	1.08	0.287	1
HBB	P68871	20.6 (3) [12]	21.8 (2.5) [10]	1.14	0.339	1
HBD	P02042	18.4 (1.5) [11]	18.6 (2.4) [10]	0.18	0.838	1
HBG2	P69892	17 (1.2) [8]	15.6 (3) [10]	-1.37	0.216	1
HEXB	P07686	14.4 (1.2) [11]	13.3 (0.5) [8]	-1.12	0.013	1
HGFAC	D6RAR4	14.1 (0.6) [9]	14.1 (0.3) [7]	0.02	0.93	1
HP	P00738	15.7 (2.1) [12]	16.3 (2.5) [10]	0.55	0.593	1
HPR	P00739	14.7 (1.7) [12]	15.8 (2.2) [9]	1.14	0.224	1
HPX	P02790	17.7 (0.7) [12]	17.7 (0.6) [10]	-0.07	0.809	1
HRG	P04196	16 (0.6) [12]	16 (0.4) [10]	0.07	0.744	1
HSP90AA1	P07900	14.8 (1.1) [10]	14.3 (0.6) [7]	-0.56	0.213	1
HSPA5	P11021	15 (0.9) [11]	14.6 (0.1) [9]	-0.41	0.178	1
HSPA8	P11142	15.3 (1.5) [12]	14.6 (0.9) [9]	-0.73	0.171	1
HSPG2	P98160	14.6 (1.1) [12]	14 (0.4) [9]	-0.63	0.097	1
HTRA1	Q92743	14.4 (0.6) [10]	14.2 (0.5) [9]	-0.19	0.457	1
ICOSLG	K4DIA0	15.2 (0.9) [12]	15 (0.5) [9]	-0.16	0.63	1
CFD	K7ERG9	16.3 (0.7) [11]	16.9 (0.6) [9]	0.66	0.037	1
CFH	P08603	16 (0.4) [12]	16.4 (1.1) [10]	0.44	0.248	1
CFHR1	B1AKG0	14.9 (0.7) [12]	15.1 (0.8) [9]	0.17	0.629	1
CFI	E7ETH0	14.8 (0.4) [12]	15 (0.3) [9]	0.18	0.27	1

Protein Name	Uniprot ID	SAH Weaned Mean (SD) [N]	SAH Shunted Mean (SD) [N]	log_2FC	P-value	Adj. P-value
CFL1	E9PK25	15.1 (1.3) [12]	14.7 (0.7) [9]	-0.43	0.356	1
CHGA	P10645	15.7 (1) [12]	15.5 (0.7) [9]	-0.14	0.71	1
CHGB	P05060	15.5 (1) [12]	15.4 (0.6) [9]	-0.05	0.894	1
CHI3L1	P36222	16.8 (0.6) [11]	16.5 (1.2) [10]	-0.25	0.574	1
CHI3L2	Q15782	14 (1.5) [10]	13.3 (0.9) [9]	-0.63	0.278	1
CHL1	O00533	14.7 (0.8) [12]	14.2 (0.7) [9]	-0.52	0.128	1
CKB	P12277	14.3 (1.2) [9]	15.9 (2.2) [6]	1.59	0.151	1
CLCNKB	A0A087X136	15.9 (0.7) [11]	15.1 (0.8) [9]	-0.78	0.045	1
CLEC11A	Q9Y240	12.9 (0.6) [7]	13.2 (0.6) [8]	0.23	0.459	1
CLEC3B	E9PHK0	16 (0.6) [12]	16.3 (0.3) [9]	0.24	0.203	1
CLSTN1	O94985	15.9 (0.8) [11]	15.4 (0.8) [10]	-0.46	0.198	1
CLSTN3	Q9BQT9	11.1 (1.5) [8]	12.2 (2.3) [9]	1.12	0.241	1
CLU	P10909	16.8 (0.6) [12]	17.3 (1.3) [10]	0.46	0.311	1
CNDP1	Q96KN2	16 (0.7) [12]	15.9 (0.5) [10]	-0.07	0.781	1
CNTN1	Q12860	14.9 (0.8) [12]	14.4 (0.6) [9]	-0.45	0.156	1
CNTN2	A0A1W2PQ11	14.8 (0.9) [12]	14.4 (0.7) [9]	-0.34	0.336	1
COL18A1	P39060	14.7 (1) [12]	14.9 (0.5) [9]	0.20	0.557	1
COL6A1	A0A087X0S5	14.6 (0.4) [11]	14.4 (0.3) [9]	-0.27	0.116	1
COL6A3	P12111	14 (0.4) [11]	14.3 (0.2) [9]	0.30	0.056	1
CP	P00450	16.5 (0.3) [12]	16.7 (0.8) [10]	0.28	0.303	1
CPB2	A0A087WSY5	15.4 (0.6) [12]	15.8 (2.1) [10]	0.39	0.572	1
CPE	P16870	15.6 (1.2) [12]	14.9 (0.6) [9]	-0.68	0.106	1
CPN1	P15169	14.2 (0.9) [7]	14.6 (1.1) [8]	0.40	0.44	1
CPN2	P22792	15.3 (1) [12]	15.3 (0.5) [9]	-0.05	0.875	1
CPQ	Q9Y646	14.5 (1.1) [12]	13.7 (0.8) [9]	-0.83	0.066	1
CPVL	Q9H3G5	14.7 (0.9) [11]	13.6 (0.5) [9]	-1.03	0.004	1
CRP	P02741	15.2 (1.4) [8]	15.1 (1.4) [8]	-0.04	0.96	1
CRTAC1	A0A0C4DFP6	14.9 (0.4) [11]	14.5 (0.5) [9]	-0.37	0.101	1
CSF1	P09603	14.5 (0.9) [11]	13.7 (0.9) [8]	-0.82	0.073	1
CST3	P01034	17.4 (0.8) [12]	17.6 (1.1) [10]	0.24	0.564	1
CTBS	Q01459	14.5 (0.7) [10]	13.8 (0.3) [9]	-0.67	0.016	1
CTSA	P10619	13.4 (0.6) [9]	12.2 (0.4) [6]	-1.17	0.001	0.344
CTSB	P07858	15.1 (1.7) [11]	14.7 (0.8) [8]	-0.47	0.443	1
CTSD	A0A1B0GV23	16.6 (1.2) [12]	15.7 (0.5) [9]	-0.89	0.04	1
CTSH	A0A087X0D5	14.6 (1) [10]	13.5 (0.7) [7]	-1.19	0.011	1
CTSL	P07711	15.2 (0.9) [11]	14.1 (0.4) [9]	-1.16	0.001	0.757
DAG1	Q14118	15.3 (0.5) [11]	15.1 (0.3) [9]	-0.18	0.29	1
DBI	A0A0A0MTI5	15.2 (0.7) [12]	15 (0.3) [8]	-0.22	0.37	1
DKK3	F6SYF8	15.8 (1.1) [12]	15.5 (0.3) [9]	-0.28	0.404	1
DPP7	Q9UHL4	13.5 (1) [9]	12.4 (1.1) [6]	-1.01	0.097	1
DSC2	Q02487	13.9 (0.8) [10]	13.5 (0.6) [9]	-0.43	0.183	1
ECM1	Q16610	14.6 (0.5) [12]	14.4 (0.2) [9]	-0.23	0.169	1

Protein Name	Uniprot ID	SAH Weaned Mean (SD) [N]	SAH Shunted Mean (SD) [N]	log_2FC	P-value	Adj. P-value
EFEMP1	A0A0U1RQV3	16.3 (0.8) [11]	16.1 (0.4) [10]	-0.19	0.504	1
EIF5A	I3L397	15 (1) [11]	14 (0.7) [9]	-1.00	0.021	1
ENDOD1	O94919	15.1 (0.4) [11]	14.6 (0.3) [9]	-0.48	0.011	1
ENO2	P09104	14.5 (0.8) [11]	13.9 (1) [8]	-0.67	0.141	1
ENPP2	E7EUF1	16.7 (1.2) [12]	16 (0.4) [9]	-0.70	0.073	1
EPHA4	E9PG71	14.9 (0.8) [11]	14.3 (0.6) [9]	-0.56	0.078	1
ERN1	O75460	15.2 (1.9) [7]	14.7 (1.6) [6]	-0.45	0.652	1
EXTL2	Q9UBQ6	14.3 (0.6) [9]	13.7 (0.4) [9]	-0.65	0.014	1
F10	P00742	14.1 (0.6) [9]	14.2 (0.5) [8]	0.14	0.576	1
F12	P00748	16.1 (0.7) [12]	16.4 (0.6) [9]	0.29	0.313	1
F13A1	P00488	13.5 (2) [7]	12.4 (0.7) [8]	-1.08	0.217	1
F13B	P05160	12.9 (0.9) [5]	10 (3.5) [5]	-2.83	0.143	1
F2	P00734	16.1 (0.4) [12]	16.1 (0.5) [9]	0.01	0.968	1
F5	A0A0A0MRJ7	15.1 (0.9) [12]	14.1 (0.6) [9]	-1.08	0.004	1
F9	P00740	13.3 (0.6) [11]	13.2 (0.4) [9]	-0.08	0.733	1
FAM3C	Q92520	14.9 (0.7) [12]	14.5 (0.4) [9]	-0.44	0.091	1
FBLN1	P23142	16.4 (0.8) [12]	16.4 (1.1) [10]	0.02	0.96	1
FCGR3A	A0A1W2PQB1	15.5 (1.2) [12]	15.3 (0.6) [9]	-0.12	0.756	1
FETUB	Q9UGM5	14.5 (0.9) [10]	14.2 (0.6) [9]	-0.33	0.341	1
FGA	P02671	15.8 (1) [12]	16.4 (0.6) [10]	0.63	0.08	1
FGB	P02675	17.3 (1.2) [12]	18.2 (1) [10]	0.97	0.056	1
FGFR2	A0A0A0MR25	14.8 (0.5) [8]	14.3 (0.5) [5]	-0.58	0.08	1
FGG	P02679	16.5 (1.1) [12]	17.3 (1) [10]	0.85	0.081	1
FLNA	P21333	13.2 (0.7) [8]	13.4 (0.9) [9]	0.22	0.586	1
FN1	P02751	16.3 (0.3) [12]	17 (0.8) [10]	0.63	0.042	1
FSTL1	Q12841	14.1 (1.4) [12]	14.1 (1.1) [9]	0.01	0.991	1
FUCA1	P04066	14.3 (1) [12]	13.3 (0.5) [8]	-1.00	0.009	1
FUCA2	Q9BTY2	14.4 (1.1) [11]	12.8 (0.7) [9]	-1.54	0.002	1
FXYD6	Q9H0Q3	15 (0.5) [8]	14.5 (0.7) [8]	-0.48	0.148	1
LAMP2	P13473	15.3 (0.9) [12]	14.6 (0.5) [9]	-0.69	0.041	1
LBP	P18428	14 (0.8) [8]	14 (0.6) [8]	-0.04	0.912	1
LCAT	P04180	14.6 (0.5) [10]	14.8 (0.4) [9]	0.13	0.565	1
LCP1	P13796	15.7 (1.2) [12]	14.9 (0.9) [9]	-0.81	0.092	1
LDHA	P00338	15.4 (1.2) [12]	15 (0.5) [9]	-0.43	0.272	1
LDHB	P07195	15.4 (0.6) [12]	15 (0.5) [9]	-0.34	0.184	1
LGALS1	P09382	15.2 (1.1) [12]	15 (0.5) [9]	-0.15	0.68	1
LGALS3BP	Q08380	16.6 (0.7) [12]	15.7 (0.5) [9]	-0.92	0.003	1
LIAS	A0A1W2PNQ5	14.6 (1.2) [9]	14.9 (0.9) [8]	0.27	0.603	1
LMAN2	D6RBV2	14.9 (0.3) [11]	14.7 (0.4) [9]	-0.11	0.448	1
LRG1	P02750	16.8 (0.4) [12]	17.2 (0.6) [9]	0.32	0.164	1
LRP1	Q07954	13.3 (1) [8]	12.5 (0.5) [8]	-0.83	0.067	1
LRRC4B	Q9NT99	13.6 (0.9) [9]	13.1 (0.7) [7]	-0.56	0.199	1

Protein Name	Uniprot ID	SAH Weaned<br>Mean (SD) [N]	SAH Shunted<br>Mean (SD) [N]	log_2FC	P-value	Adj. P-value
LSAMP	H3BLU2	14.6 (0.9) [12]	14.1 (0.5) [9]	-0.51	0.132	1
LTF	E7EQB2	15 (1.6) [12]	14 (1) [9]	-1.01	0.086	1
LUM	P51884	15.7 (0.4) [12]	15.5 (0.3) [10]	-0.19	0.195	1
LYVE1	Q9Y5Y7	15.9 (1.3) [12]	15.6 (0.8) [9]	-0.36	0.444	1
LYZ	A0A0B4J259	17.4 (1) [12]	17.5 (0.8) [9]	0.10	0.789	1
MAN1A1	P33908	14 (0.6) [11]	13.9 (0.3) [8]	-0.15	0.485	1
MAN1C1	Q9NR34	13.4 (0.3) [7]	12.6 (1.1) [6]	-0.78	0.16	1
MAN2A2	P49641	13.9 (0.9) [9]	13.3 (0.4) [7]	-0.52	0.162	1
MBL2	P11226	11.4 (1.5) [6]	10.6 (1) [5]	-0.83	0.312	1
MCAM	P43121	14.4 (0.7) [11]	14.2 (0.8) [9]	-0.13	0.715	1
MDH1	P40925	15.8 (0.7) [12]	15.3 (0.8) [9]	-0.50	0.162	1
MEGF8	Q7Z7M0	13.7 (0.9) [7]	12.9 (0.6) [8]	-0.81	0.064	1
MMP2	P08253	15.2 (0.6) [11]	15.2 (0.5) [9]	-0.06	0.826	1
MST1	G3XAK1	13.1 (0.6) [8]	13.3 (0.5) [7]	0.12	0.669	1
NBL1	A0A087WTY6	18.2 (0.6) [11]	18 (0.4) [9]	-0.20	0.356	1
NCAM1	P13591	15.4 (1) [12]	15.3 (0.7) [10]	-0.04	0.904	1
NCAM2	H9KV31	14.7 (0.6) [11]	14.1 (0.3) [9]	-0.64	0.005	1
NCAN	O14594	14.5 (0.6) [9]	13.9 (0.9) [9]	-0.59	0.132	1
NEGR1	Q7Z3B1	14.4 (1.3) [12]	13.5 (1.2) [8]	-0.91	0.129	1
NELL2	F8VVB6	14.7 (0.6) [12]	13.8 (0.9) [9]	-0.90	0.024	1
NEO1	Q92859	14.4 (0.5) [11]	13.7 (0.6) [8]	-0.69	0.018	1
NFASC	O94856	14 (0.5) [8]	13.5 (0.7) [7]	-0.58	0.099	1
NPC2	E7EMS2	16.5 (0.9) [11]	16 (0.9) [10]	-0.49	0.224	1
NPDC1	Q5SPY9	13.2 (0.9) [11]	12.6 (0.7) [9]	-0.61	0.117	1
NPTX1	Q15818	14.6 (1.2) [11]	14 (1) [9]	-0.57	0.25	1
NPTXR	A0A1X7SBT7	14.5 (1.1) [12]	14.2 (1) [7]	-0.28	0.586	1
NRCAM	C9JYY6	15 (0.8) [12]	14.6 (0.8) [9]	-0.39	0.3	1
NRXN2	G5E9G7	14.5 (0.9) [10]	14.1 (0.9) [7]	-0.38	0.401	1
NRXN3	A0A0U1RQC5	13.4 (1.1) [10]	13.5 (1) [8]	0.10	0.844	1
NTM	Q9P121-4	14.6 (0.5) [11]	14 (1) [9]	-0.65	0.099	1
NUCB1	Q02818	14.1 (0.8) [11]	13.4 (0.2) [9]	-0.75	0.015	1
OAF	Q86UD1	14.6 (0.5) [8]	14.6 (0.2) [6]	0.03	0.888	1
OGN	P20774	15.6 (0.9) [12]	15.9 (0.4) [9]	0.27	0.342	1
OMD	Q99983	13.5 (1.1) [6]	13.1 (0.6) [6]	-0.42	0.427	1
OMG	P23515	15 (0.8) [10]	13.9 (1.3) [7]	-1.15	0.061	1
ORM1	P02763	19.5 (0.5) [12]	19.8 (0.9) [10]	0.26	0.426	1
ORM2	P19652	17.9 (0.7) [12]	18.4 (0.6) [10]	0.45	0.107	1
PAM	P19021	14.6 (0.6) [10]	14.2 (0.6) [9]	-0.44	0.129	1
PARK7	Q99497	14.1 (1) [8]	13.6 (0.7) [7]	-0.46	0.32	1
PCOLCE	Q15113	15.8 (1) [12]	16.3 (0.3) [9]	0.42	0.179	1
PCSK1N	Q9UHG2	15.3 (0.7) [12]	15 (0.4) [9]	-0.32	0.227	1
PDIA6	Q15084	14 (1.5) [8]	13.2 (0.9) [6]	-0.84	0.207	1

**Protein Name**	**Uniprot ID**	**SAH Weaned** Mean (SD) [N]	**SAH Shunted** Mean (SD) [N]	**log_2FC**	**P-value**	**Adj. P-value**
PEBP1	P30086	16.1 (0.7) [12]	15.6 (0.7) [9]	-0.45	0.141	1
PEBP4	Q96S96	15.5 (0.5) [11]	15.3 (0.5) [9]	-0.19	0.427	1
PENK	P01210	14.4 (1) [12]	13.5 (1.1) [9]	-0.88	0.082	1
PEPD	P12955	13.3 (0.7) [10]	13.4 (0.6) [9]	0.02	0.938	1
PFN1	P07737	16.3 (0.9) [12]	15.7 (0.6) [9]	-0.56	0.097	1
PGK1	P00558	15.7 (1.2) [12]	15.3 (0.7) [9]	-0.44	0.298	1
PGLYRP2	Q96PD5	14.9 (1.4) [11]	14.4 (0.9) [10]	-0.53	0.308	1
PIK3IP1	Q96FE7-4	15 (0.6) [10]	14.3 (0.9) [7]	-0.68	0.1	1
PLG	P00747	16.1 (0.7) [12]	16.3 (1) [10]	0.28	0.457	1
PLTP	P55058	16.1 (1.2) [12]	16.3 (0.6) [10]	0.18	0.652	1
PLXDC2	Q6UX71	14.7 (1.1) [12]	14.3 (0.7) [9]	-0.38	0.342	1
PMFBP1	G3V1Q7	16.2 (0.4) [8]	16.3 (0.3) [9]	0.04	0.848	1
PON1	P27169	15.5 (1.1) [12]	15.8 (0.9) [9]	0.30	0.475	1
PPIA	P62937	16.6 (1.1) [12]	16.1 (0.9) [10]	-0.49	0.271	1
PPIB	P23284	15 (0.9) [12]	13.9 (0.6) [9]	-1.11	0.003	1
PRCP	P42785	15 (0.6) [10]	14.6 (0.5) [5]	-0.36	0.222	1
PRDX1	Q06830	17 (1.7) [12]	17.4 (2.1) [10]	0.37	0.663	1
PRDX2	P32119	17.8 (2.2) [12]	18 (1.6) [10]	0.22	0.79	1
PRDX6	P30041	15.4 (1.4) [11]	15.1 (1.1) [9]	-0.33	0.554	1
PRG4	A0A0U1RR20	13.4 (0.7) [9]	14.1 (1.2) [9]	0.72	0.14	1
PRNP	A2A2V1	13.4 (0.4) [10]	13.3 (0.9) [9]	-0.10	0.749	1
PROC	E7END6	13.6 (0.6) [11]	13.6 (0.4) [9]	-0.01	0.979	1
PROCR	Q9UNN8	14.5 (0.6) [12]	14.2 (0.5) [9]	-0.30	0.223	1
PROS1	P07225	15.3 (0.5) [12]	15.1 (0.7) [10]	-0.28	0.31	1
PROZ	P22891	13.2 (0.6) [10]	13.9 (0.3) [7]	0.69	0.01	1
PTGDS	P41222	19.8 (1.1) [12]	20 (0.4) [10]	0.11	0.738	1
PTPRG	P23470	14.1 (1) [9]	13.8 (0.6) [8]	-0.29	0.461	1
PTPRZ1	P23471	14.5 (0.8) [11]	13.9 (0.6) [9]	-0.67	0.036	1
PVALB	B8ZZ19	13.8 (0.6) [9]	13.8 (0.5) [7]	0.01	0.981	1
PZP	P20742	13.6 (0.3) [5]	13.5 (1.9) [8]	-0.16	0.816	1
QDPR	P09417	14.2 (0.4) [8]	13.4 (0.9) [6]	-0.74	0.118	1
QPCT	Q16769	13.3 (0.5) [8]	12.8 (0.7) [8]	-0.48	0.141	1
QSOX1	O00391	14.4 (0.4) [11]	14.4 (0.4) [9]	0.02	0.902	1
RARRES2	Q99969	14.9 (0.8) [11]	14.5 (0.6) [9]	-0.42	0.222	1
RBP4	P02753	16.2 (0.6) [12]	16.5 (1) [10]	0.37	0.326	1
RNASET2	A0A087WZM2	14.7 (0.8) [11]	13.7 (0.6) [9]	-1.09	0.003	1
ROBO1	Q9Y6N7	13.4 (0.9) [8]	13.2 (0.3) [5]	-0.24	0.534	1
S100A8	P05109	18.3 (1.7) [11]	15.9 (1.6) [9]	-2.44	0.004	1
S100B	P04271	13.8 (1.4) [8]	13.7 (1) [6]	-0.08	0.899	1
SAA1	P0DJI8	15.4 (1.2) [9]	15.5 (1.7) [6]	0.13	0.878	1
SAA4	P35542	15.5 (0.7) [12]	15.8 (0.4) [9]	0.36	0.153	1
SCG2	P13521	13.4 (0.8) [12]	13.1 (0.8) [9]	-0.32	0.379	1

Protein Name	Uniprot ID	SAH Weaned Mean (SD) [N]	SAH Shunted Mean (SD) [N]	log_2FC	P-value	Adj. P-value
SCG3	Q8WXD2	15.2 (1) [12]	15.1 (0.7) [9]	-0.17	0.659	1
SCG5	P05408-2	15.7 (0.9) [12]	15.5 (1.1) [10]	-0.19	0.653	1
SCRG1	O75711	17.2 (0.7) [11]	16.9 (0.4) [9]	-0.28	0.266	1
SELL	P14151	15.6 (0.5) [11]	15.1 (0.4) [9]	-0.51	0.027	1
SERPINA1	P01009	18.4 (0.3) [12]	18.8 (0.9) [10]	0.35	0.267	1
SERPINA10	G3V2W1	12.6 (1) [9]	12.1 (1.1) [8]	-0.50	0.329	1
SERPINA3	P01011	17.7 (0.6) [12]	18.1 (1) [10]	0.40	0.263	1
SERPINA4	P29622	15 (0.5) [12]	14.8 (0.4) [9]	-0.23	0.217	1
SERPINA5	P05154	14.4 (0.8) [11]	14.1 (0.4) [9]	-0.32	0.27	1
SERPINA6	P08185	15.4 (0.5) [12]	15.4 (0.8) [10]	-0.01	0.976	1
SERPINA7	P05543	14.8 (0.4) [12]	14.9 (0.7) [10]	0.04	0.895	1
SERPINC1	P01008	16.9 (0.4) [12]	17.2 (0.8) [10]	0.37	0.216	1
SERPIND1	P05546	15.3 (0.4) [12]	15.3 (0.6) [10]	-0.01	0.955	1
SERPINF1	P36955	17.6 (0.9) [12]	16.9 (0.4) [10]	-0.74	0.026	1
SERPINF2	P08697	16.1 (0.3) [12]	16.4 (0.6) [10]	0.24	0.261	1
SERPING1	P05155	16.7 (0.3) [12]	16.8 (0.3) [10]	0.04	0.765	1
SEZ6	Q53EL9	13.5 (0.7) [8]	13.1 (0.4) [8]	-0.39	0.19	1
SEZ6L2	A0A087WYL5	14.5 (0.6) [11]	14.7 (0.6) [8]	0.20	0.494	1
SHBG	I3L145	13.5 (0.5) [8]	13.9 (0.5) [9]	0.42	0.095	1
SIRPA	P78324	14.4 (0.8) [10]	13.6 (0.8) [8]	-0.74	0.058	1
SKP1	E5RJR5	14.4 (0.7) [9]	13.9 (0.9) [9]	-0.43	0.279	1
SOD1	P00441	16.1 (0.5) [12]	15.8 (0.3) [9]	-0.29	0.126	1
SOD3	P08294	15.7 (1.1) [12]	15 (0.4) [9]	-0.67	0.071	1
SPARC	P09486	15.5 (1.2) [12]	15.8 (0.7) [10]	0.30	0.475	1
SPARCL1	Q14515	14.4 (0.8) [12]	14.4 (0.5) [9]	-0.03	0.913	1
SPOCK1	Q08629	13.2 (0.7) [8]	12.6 (0.9) [7]	-0.55	0.23	1
SPP1	P10451	16.1 (1.4) [12]	16.4 (0.7) [9]	0.27	0.557	1
SYNE3	G3V533	14.8 (0.9) [7]	15 (0.3) [7]	0.13	0.726	1
SYT2	Q8N9I0	17.5 (1) [8]	18.7 (0.6) [7]	1.16	0.018	1
TAGLN	Q01995	14.4 (0.5) [11]	15 (0.6) [9]	0.57	0.044	1
TALDO1	P37837	15.4 (1.1) [11]	14.3 (0.9) [9]	-1.10	0.024	1
TF	P02787	17.7 (0.5) [12]	17.9 (1) [10]	0.18	0.609	1
TFRC	G3V0E5	11.2 (0.6) [5]	10.8 (0.8) [6]	-0.43	0.344	1
TGFBI	Q15582	15.4 (1) [12]	15.7 (1.4) [10]	0.36	0.49	1
THBS1	P07996	14.4 (0.6) [8]	14.5 (0.7) [7]	0.17	0.624	1
THY1	E9PIM6	16.3 (0.9) [12]	15.8 (0.6) [9]	-0.58	0.096	1
TIMP1	P01033	18.5 (2.1) [12]	18.5 (1.4) [10]	-0.03	0.972	1
TIMP2	P16035	15.7 (0.7) [10]	15.4 (0.8) [7]	-0.27	0.466	1
TNXB	A0A140TA41	13.3 (0.5) [11]	13.5 (0.5) [9]	0.22	0.362	1
TPI1	P60174	14.9 (1.1) [12]	14 (1) [9]	-0.87	0.078	1
TPP1	O14773	14.6 (0.9) [12]	13.3 (0.5) [7]	-1.30	0.001	0.527
TPP2	P29144	14.4 (1.2) [10]	15.1 (0.8) [9]	0.62	0.208	1

Protein Name	Uniprot ID	SAH Weaned<br>Mean (SD) [N]	SAH Shunted<br>Mean (SD) [N]	log_2FC	P-value	Adj. P-value
TREM2	Q9NZC2	14.1 (0.7) [9]	14.4 (0.5) [9]	0.37	0.232	1
TTR	P02766	19.3 (0.9) [12]	19 (0.7) [10]	-0.28	0.445	1
TUBA1B	P68363	15.2 (1.5) [11]	14.5 (1.6) [9]	-0.78	0.29	1
TUBB4B	P68371	15.2 (1.4) [9]	15 (1.1) [6]	-0.18	0.789	1
TXN	P10599	17.1 (0.9) [12]	17.1 (0.5) [9]	-0.03	0.921	1
UBC	F5H265	16.3 (0.8) [12]	15.8 (0.3) [9]	-0.54	0.045	1
VASN	Q6EMK4	14.4 (0.2) [11]	14.3 (0.3) [9]	-0.11	0.392	1
VCAM1	P19320	14 (0.8) [10]	13.9 (0.3) [8]	-0.10	0.714	1
VCAN	P13611	14.5 (0.7) [11]	14.2 (0.7) [9]	-0.29	0.372	1
VGF	O15240	14.1 (1) [12]	13.7 (1.1) [9]	-0.39	0.423	1
VIM	P08670	16.8 (1.8) [12]	16.1 (1.5) [9]	-0.75	0.317	1
VIP	P01282	18.2 (0.4) [12]	18.4 (0.7) [9]	0.24	0.378	1
VTN	P04004	17.2 (0.6) [12]	17.3 (0.4) [9]	0.08	0.721	1
WFIKKN2	C9J6G4	15.3 (0.7) [11]	13.9 (0.6) [9]	-1.40	<0.001	0.104
YWHAE	P62258	14.7 (1.4) [12]	14.5 (0.6) [8]	-0.24	0.601	1
YWHAZ	P63104	15.4 (1.2) [12]	15 (0.5) [9]	-0.33	0.4	1
IGKV1-16	P04430	14.3 (0.4) [7]	14.4 (0.9) [8]	0.11	0.753	1
IGKV1-17	P01599	15.9 (0.8) [12]	15.4 (0.6) [9]	-0.43	0.176	1
IGKV1-27	A0A075B6S5	15.6 (0.6) [9]	15.9 (0.3) [8]	0.27	0.28	1
IGKV1-5	P01602	16.8 (0.7) [12]	16.8 (0.6) [9]	0.06	0.835	1
IGKV1-8	A0A0C4DH67	16.5 (0.6) [12]	17.1 (1.1) [10]	0.60	0.142	1
IGKV1D-33	P01593	17.4 (0.9) [12]	17.8 (0.5) [10]	0.37	0.247	1
IGKV1D-37	A0A075B6S9	16.2 (0.9) [6]	16.1 (1.6) [6]	-0.04	0.957	1
IGKV2-28	A0A075B6P5	16.2 (0.5) [11]	16.5 (0.7) [9]	0.31	0.282	1
IGKV2-29	A2NJV5	16.5 (0.6) [12]	16.1 (0.7) [9]	-0.32	0.293	1
IGKV2-40	A0A087WW87	13.5 (0.6) [7]	14.3 (1.9) [7]	0.81	0.306	1
IGKV2D-24	A0A075B6R9	16.8 (0.7) [12]	16.4 (1) [9]	-0.40	0.305	1
IGKV3-15	P01624	17.5 (0.9) [12]	18.2 (0.7) [9]	0.73	0.043	1
IGKV3-20	P01619	18.5 (0.6) [12]	19.1 (1.3) [10]	0.56	0.239	1
IGKV3-7	A0A075B6H7	18.8 (1.6) [11]	19.3 (1.1) [9]	0.50	0.416	1
IGKV3D-11	A0A0A0MRZ8	17.6 (0.6) [12]	17.6 (0.5) [9]	-0.04	0.859	1
IGKV3D-15	A0A087WSY6	17.2 (1.1) [11]	16.8 (0.8) [9]	-0.39	0.362	1
IGKV3D-20	A0A0C4DH25	18.2 (1.1) [12]	18.2 (0.7) [9]	-0.07	0.858	1
IGKV4-1	P06312	17.2 (0.5) [12]	17.6 (0.5) [9]	0.47	0.045	1
IGLC3	P0DOY3	20.1 (0.6) [12]	20.5 (0.3) [9]	0.41	0.055	1
IGLL5	A0A0B4J231	18 (0.6) [12]	18.2 (0.7) [10]	0.19	0.509	1
IGLV1-47	P01700	16.9 (0.6) [12]	17.3 (2.4) [10]	0.38	0.627	1
IGLV1-51	P01701	15.6 (0.5) [10]	15.8 (0.9) [8]	0.18	0.62	1
IGLV3-10	A0A075B6K4	15.3 (0.9) [10]	15.3 (0.5) [9]	0.05	0.884	1
IGLV3-19	P01714	14.7 (0.6) [11]	14.5 (0.6) [9]	-0.13	0.624	1
IGLV3-21	P80748	15.3 (1) [10]	16.5 (0.8) [7]	1.12	0.023	1
IGLV3-25	P01717	14.5 (0.6) [12]	15.1 (0.8) [9]	0.57	0.085	1

**Protein Name**	**Uniprot ID**	**SAH Weaned** Mean (SD) [N]	**SAH Shunted** Mean (SD) [N]	**log$_2$FC**	**P-value**	**Adj. P-value**
IGLV3-9	A0A075B6K5	14.9 (0.7) [12]	15.6 (0.6) [9]	0.72	0.017	1
IGLV6-57	P01721	14.6 (0.8) [10]	14.9 (0.8) [9]	0.36	0.355	1
IGLV7-46	A0A075B6I9	15.4 (0.7) [11]	15.2 (1.1) [9]	-0.18	0.679	1
IGLV8-61	A0A075B6I0	15.7 (0.9) [7]	15.4 (0.6) [8]	-0.27	0.513	1
IGSF8	Q969P0	15.3 (0.6) [11]	14.8 (0.4) [9]	-0.56	0.025	1
IL6ST	P40189	13.7 (0.5) [8]	13.3 (0.5) [6]	-0.47	0.094	1
ISLR	O14498	15.1 (0.7) [12]	15.7 (0.4) [9]	0.56	0.027	1
ITIH1	P19827	15.6 (0.7) [12]	15.8 (0.5) [10]	0.22	0.405	1
ITIH2	P19823	15.6 (0.7) [12]	16 (0.6) [10]	0.49	0.104	1
ITIH3	Q06033	14.5 (0.7) [11]	14.7 (0.5) [9]	0.17	0.545	1
ITIH4	Q14624	16.3 (0.7) [12]	16.7 (0.8) [10]	0.40	0.237	1
ITPR2	Q14571	18.2 (1.1) [12]	18.4 (0.8) [9]	0.20	0.642	1
JCHAIN	D6RD17	16.8 (1.4) [11]	17.2 (1) [9]	0.38	0.493	1
KLK6	Q92876	16 (1.2) [12]	15.6 (0.5) [9]	-0.36	0.378	1
KLKB1	H0YAC1	14.1 (0.9) [11]	14.1 (0.5) [9]	-0.03	0.92	1
KNG1	P01042	16.9 (0.6) [12]	17.3 (0.3) [10]	0.39	0.064	1
KRT1	P04264	15.5 (2.2) [11]	15.2 (0.9) [5]	-0.32	0.682	1
IGHG2	P01859	19.5 (0.6) [12]	19.9 (0.7) [10]	0.43	0.133	1
IGHG3	P01860	16.1 (1.1) [12]	16.7 (0.8) [9]	0.58	0.174	1
IGHG4	A0A286YFJ8	15.8 (1) [12]	15.9 (1.2) [10]	0.12	0.802	1
IGHM	A0A1B0GUU9	17 (1.4) [12]	17.8 (0.9) [9]	0.73	0.17	1
IGHV1-18	A0A0C4DH31	14.5 (0.9) [9]	14.5 (0.8) [8]	0.02	0.953	1
IGHV1-2	P23083	15.2 (0.9) [10]	15.2 (0.6) [8]	-0.05	0.885	1
IGHV1OR15-1	A0A075B7D0	17 (1.3) [12]	16.9 (1.6) [9]	-0.09	0.891	1
IGHV2-26	A0A0B4J1V2	13.6 (0.9) [10]	13.9 (0.5) [9]	0.24	0.485	1
IGHV2-5	P01817	12.8 (1.3) [7]	12.7 (1.4) [7]	-0.06	0.936	1
IGHV3-15	A0A0B4J1V0	16.1 (0.5) [12]	16.2 (0.8) [9]	0.06	0.828	1
IGHV3-30	P01768	17 (0.5) [12]	16.7 (0.4) [9]	-0.25	0.219	1
IGHV3-38	A0A0C4DH36	15.5 (0.7) [12]	15.2 (0.6) [9]	-0.23	0.446	1
IGHV3-49	A0A0A0MS15	16.9 (0.5) [12]	16.6 (0.9) [9]	-0.29	0.401	1
IGHV3-64D	A0A0J9YX35	15.9 (0.5) [10]	15.9 (0.7) [7]	-0.04	0.917	1
IGHV3-7	P01780	17.3 (0.6) [12]	17.5 (0.5) [9]	0.17	0.46	1
IGHV3-72	A0A0B4J1Y9	16.5 (0.6) [12]	16.8 (0.5) [9]	0.31	0.216	1
IGHV3OR16-9	A0A0B4J2B5	19.8 (0.5) [12]	20.1 (0.8) [10]	0.33	0.262	1
IGHV4-34	P06331	15.3 (0.8) [12]	15.3 (0.6) [9]	-0.01	0.977	1
IGHV5-51	A0A0C4DH38	16 (0.6) [12]	16.6 (2.6) [10]	0.60	0.482	1
IGKC	P01834	20 (0.5) [12]	20.9 (1.9) [10]	0.84	0.211	1
IGKV1-12	A0A0C4DH73	17.1 (0.6) [12]	17.6 (0.9) [10]	0.46	0.2	1
IGHG1	P01857	19.6 (0.5) [12]	19.7 (0.4) [10]	0.17	0.37	1
IGFBP7	Q16270	16.7 (1.3) [12]	16.2 (0.9) [10]	-0.55	0.258	1

Protein Name	Uniprot ID	SAH Weaned Mean (SD) [N]	SAH Shunted Mean (SD) [N]	log_2FC	P-value	Adj. P-value
IGFALS	P35858	14.8 (0.9) [11]	14.7 (0.4) [9]	-0.15	0.625	1
IGFBP6	P24592	17.4 (1.1) [12]	17.7 (0.8) [9]	0.38	0.375	1
IGHA1	A0A286YEY1	18 (0.8) [12]	18.7 (0.8) [10]	0.65	0.073	1
IGHA2	A0A286YEY5	18.1 (0.7) [12]	18.6 (1) [10]	0.46	0.216	1
IGHD	A0A0A0MS09	15.1 (1.4) [7]	15.5 (1.1) [7]	0.43	0.545	1
CD99	P14209	15.9 (0.8) [8]	15.8 (0.5) [9]	-0.05	0.876	1
CAT	P04040	15.4 (1.2) [12]	15.1 (1.6) [10]	-0.31	0.62	1
ACAT2	Q9BWD1	12.2 (1.1) [7]	11.9 (0.3) [5]	-0.27	0.563	1
BASP1	P80723	12 (1.2) [9]	12.3 (1.2) [9]	0.25	0.655	1
BPGM	P07738	15.6 (1) [8]	15 (1) [6]	-0.60	0.28	1
FTH1	P02794	13.9 (2.1) [10]	12.2 (1.6) [9]	-1.70	0.065	1
FTL	P02792	16.2 (2.5) [12]	16.3 (1.5) [9]	0.06	0.949	1
GAP43	P17677	12.2 (1.6) [5]	11.9 (1.1) [8]	-0.34	0.695	1
GPR37L1	O60883	14.5 (1.3) [12]	13.9 (0.6) [9]	-0.54	0.223	1
GSS	P48637	15.5 (1.7) [8]	14 (1.5) [7]	-1.42	0.105	1
GSTO1	P78417	15.4 (1) [12]	15.2 (0.5) [9]	-0.13	0.708	1
HEXA	H3BP20	14.4 (0.7) [11]	13.8 (0.6) [7]	-0.60	0.082	1
HIST1H2BK	O60814	15.9 (1.4) [10]	14.5 (1) [7]	-1.34	0.038	1
HIST1H4A	P62805	16.9 (2) [12]	15.6 (2.3) [10]	-1.33	0.17	1
HLA-C	A0A140T921	14.3 (1.1) [10]	13.5 (1.1) [6]	-0.79	0.185	1
HPRT1	P00492	14.5 (0.5) [9]	13.6 (0.7) [6]	-0.86	0.029	1
HSPA1B	A0A0G2JIW1	15.6 (1.1) [10]	14.9 (0.8) [7]	-0.69	0.16	1
IGFBP2	P18065	14.2 (0.6) [11]	14.2 (0.7) [9]	0.06	0.851	1
IGFBP5	P24593	12.2 (1) [8]	11.8 (0.5) [6]	-0.39	0.384	1
IL31RA	Q8NI17	23.6 (0.6) [8]	23.3 (0.5) [8]	-0.25	0.385	1
ITIH5	C9J2H1	13.2 (1) [6]	13.2 (0.7) [5]	0.03	0.96	1
LCN2	P80188	15.2 (1.3) [10]	15 (1.1) [9]	-0.21	0.722	1
MAG	P20916	13 (0.8) [7]	12.4 (0.5) [5]	-0.66	0.12	1
MARCKS	P29966	12.4 (0.7) [10]	12.3 (0.7) [9]	-0.13	0.697	1
MIF	P14174	16.4 (0.9) [12]	15.8 (0.7) [8]	-0.57	0.136	1
MOG	A0A0G2JHA9	13.7 (1) [10]	13 (0.7) [9]	-0.72	0.089	1
MSN	P26038	13.8 (1.5) [11]	13.3 (0.8) [9]	-0.54	0.327	1
MYH11	P35749	15.6 (0.7) [7]	16.1 (0.7) [9]	0.47	0.226	1
MYH9	P35579	13.3 (0.6) [6]	13.5 (2.2) [8]	0.25	0.768	1
NEFM	E7EMV2	13.9 (3.2) [7]	14.1 (2.6) [5]	0.20	0.908	1
P4HB	P07237	13.7 (1.2) [9]	12.8 (0.6) [9]	-0.92	0.056	1
PDIA3	P30101	14.4 (1.1) [11]	13.5 (0.5) [7]	-0.90	0.035	1
PEA15	Q15121	14.5 (0.8) [9]	14.1 (0.5) [7]	-0.45	0.217	1
PGAM1	P18669	15.1 (0.8) [12]	14.9 (0.4) [9]	-0.28	0.343	1
PI16	Q6UXB8	13.2 (0.9) [10]	13.2 (1.5) [10]	0.01	0.979	1
PKM	P14618	15.2 (0.7) [12]	14.8 (0.5) [9]	-0.45	0.124	1
PNP	P00491	14.1 (0.3) [7]	13.7 (1.4) [6]	-0.36	0.565	1

Protein Name	Uniprot ID	SAH Weaned Mean (SD) [N]	SAH Shunted Mean (SD) [N]	log_2FC	P-value	Adj. P-value
PRSS3	B1AN99	19.4 (2.2) [11]	19.8 (1.1) [6]	0.34	0.68	1
PSMA1	P25786	12.9 (0.7) [6]	12.7 (0.5) [5]	-0.25	0.507	1
PSMA6	G3V5Z7	13.7 (1.1) [8]	12.4 (0.8) [8]	-1.34	0.015	1
PTPRN2	Q92932	15 (0.7) [8]	15.5 (0.5) [5]	0.54	0.146	1
RNASE1	P07998	12 (1.1) [11]	12.3 (1) [9]	0.30	0.524	1
S100A9	P06702	17.4 (1.5) [11]	15.4 (1.8) [9]	-2.07	0.015	1
SELENBP1	Q13228	15.3 (0.9) [12]	14.8 (0.9) [9]	-0.52	0.2	1
SERPINB1	P30740	14.4 (1.1) [10]	13.5 (0.3) [8]	-0.85	0.048	1
SH3BGRL	O75368	14.3 (1.2) [9]	13.1 (0.4) [9]	-1.20	0.016	1
SH3BGRL3	Q5T123	15.1 (1.3) [10]	14.1 (0.6) [7]	-1.01	0.048	1
SLC4A1	P02730	14 (1) [7]	13.9 (1) [7]	-0.10	0.859	1
SNCA	E7EPV7	13 (1.6) [8]	13.1 (1.1) [5]	0.07	0.933	1
SOD2	P04179	14 (0.9) [12]	13.7 (0.4) [9]	-0.37	0.24	1
TCN2	B5MBX2	15.2 (0.6) [7]	15.1 (2) [7]	-0.12	0.883	1
TGOLN2	F8W8W7	11.3 (1.1) [10]	12.2 (1.6) [9]	0.87	0.193	1
TKT	P29401	15.2 (1.2) [11]	14.2 (1) [9]	-0.92	0.076	1
TNC	P24821	13.3 (0.9) [6]	12.6 (0.4) [5]	-0.71	0.109	1
TRBV7-2	A0A1B0GXF2	14.7 (0.5) [7]	14.9 (0.5) [9]	0.15	0.567	1
VSIG4	Q9Y279	16 (1.3) [10]	15.6 (0.6) [8]	-0.38	0.418	1
WDR1	O75083	14.7 (1.3) [9]	13.5 (1.2) [5]	-1.26	0.103	1
YWHAB	P31946	13.5 (1.4) [10]	12.7 (0.7) [7]	-0.75	0.159	1
YWHAG	P61981	13.1 (1.2) [10]	13.6 (1) [8]	0.46	0.404	1
YWHAQ	P27348	14.3 (1.4) [11]	13.6 (0.8) [8]	-0.71	0.181	1
ZNF511-PRAP1	H7BY64	14.4 (0.8) [10]	13.6 (0.3) [7]	-0.79	0.013	1
CYCS	C9JFR7	13.8 (1.2) [9]	12.9 (0.5) [8]	-0.87	0.071	1
EEF1A1	P68104	14.9 (1.1) [8]	15.1 (4) [9]	0.22	0.875	1
ENO1	P06733	16.2 (1) [12]	15.2 (0.8) [9]	-0.98	0.024	1
FAM49B	Q9NUQ9	13.5 (1) [9]	12.8 (0.5) [9]	-0.73	0.064	1
FCGBP	Q9Y6R7	16.3 (1.1) [10]	15.4 (1.2) [9]	-0.87	0.128	1
FKBP1A	P62942	15.6 (1.2) [10]	15 (0.5) [8]	-0.58	0.178	1
COL1A2	A0A087WTA8	15.4 (1.3) [12]	15.8 (0.3) [9]	0.47	0.266	1
CORO1A	P31146	13.9 (1.6) [10]	12.3 (0.8) [7]	-1.60	0.017	1
COTL1	Q14019	13.8 (1.2) [8]	13.1 (0.4) [9]	-0.66	0.176	1
CSF1R	E9PEK4	15.9 (0.5) [11]	15.4 (0.5) [9]	-0.54	0.034	1
CTSS	P25774	14.7 (1) [10]	13.8 (0.4) [8]	-0.88	0.024	1
CSTB	P04080	15.9 (1.2) [12]	15.6 (0.5) [9]	-0.33	0.42	1
COLEC12	Q5KU26	14.4 (0.5) [10]	14.4 (0.7) [8]	-0.05	0.861	1
CTSC	P53634	14.1 (1) [9]	13.6 (0.6) [6]	-0.48	0.252	1
DCN	P07585	13.4 (1.2) [8]	13 (1.2) [9]	-0.42	0.475	1
ESD	H7BZT7	12.7 (1.2) [8]	12.7 (1) [7]	-0.06	0.919	1
CCP110	O43303	20.7 (0.8) [8]	21 (0.8) [6]	0.33	0.466	1

Protein Name	Uniprot ID	SAH Weaned Mean (SD) [N]	SAH Shunted Mean (SD) [N]	log_2FC	P-value	Adj. P-value
COL1A1	P02452	15.6 (1.3) [12]	17 (1.7) [10]	1.31	0.056	1
COL2A1	P02458	16.3 (1.2) [7]	15.6 (2.4) [9]	-0.67	0.484	1
COL3A1	P02461	14.6 (1) [10]	15.6 (0.6) [8]	1.01	0.019	1
COL6A2	P12110	13.4 (0.8) [5]	13 (0.9) [5]	-0.47	0.389	1
IGHV3OR16-12	A0A075B7B8	13.2 (0.5) [8]	13.1 (0.6) [8]	-0.16	0.587	1
FRZB	Q92765	13.8 (1.1) [8]	12.9 (0.8) [8]	-0.96	0.079	1
IGF2	P01344	15 (0.7) [5]	14.5 (0.2) [8]	-0.48	0.2	1
ADAM22	F8WAD8	11 (1.3) [8]	11 (1.3) [7]	-0.03	0.961	1
PTPRS	Q13332	14.1 (0.5) [11]	13.4 (0.6) [6]	-0.73	0.028	1
SEMA7A	O75326	13.4 (1.1) [8]	12.7 (1) [7]	-0.67	0.24	1
SULF2	Q8IWU5	14.4 (0.6) [11]	14.1 (0.4) [9]	-0.29	0.226	1
THBS2	P35442	13.7 (0.4) [7]	13.9 (0.1) [7]	0.18	0.333	1
LAMA2	A0A087WX80	9.9 (1) [7]	10.5 (1.5) [8]	0.52	0.43	1
MGP	P08493	15.7 (0.8) [11]	16.3 (0.4) [8]	0.66	0.032	1
NID1	P14543	13.7 (0.6) [10]	13.6 (0.5) [8]	-0.06	0.824	1
NID2	Q14112	14.3 (0.6) [8]	13.8 (0.8) [8]	-0.49	0.202	1
PAPLN	O95428	13.3 (0.6) [7]	13 (0.5) [6]	-0.26	0.404	1
ADGRB2	A2A3C1	14.7 (0.8) [9]	14 (0.6) [6]	-0.71	0.07	1
ADGRL1	O94910	13.8 (0.5) [5]	13.8 (0.8) [7]	-0.04	0.916	1
CGREF1	Q99674	13 (0.6) [9]	13 (0.9) [9]	0.04	0.908	1
AGA	P20933	13.1 (1.5) [9]	13.9 (1) [5]	0.79	0.252	1
ATP1A1	P05023	13.7 (0.4) [5]	13.9 (0.4) [6]	0.22	0.334	1
CADM1	A0A087X0T8	15.3 (0.7) [10]	14.6 (0.5) [9]	-0.73	0.022	1
CADM2	Q8N3J6	14.1 (0.6) [7]	13.4 (0.6) [6]	-0.78	0.032	1
GOLM1	Q8NBJ4	13.5 (0.4) [10]	13.2 (0.5) [7]	-0.36	0.146	1
HSP90B1	P14625	14.6 (0.9) [10]	14.2 (0.3) [9]	-0.39	0.225	1
HYOU1	A0A087X054	14.2 (0.3) [6]	13.8 (0.4) [7]	-0.42	0.054	1
IMPAD1	Q9NX62	13.7 (0.7) [8]	12.6 (0.7) [7]	-1.15	0.009	1
KIAA1549L	H0YDE5	13.6 (0.9) [10]	13.3 (0.9) [7]	-0.26	0.577	1
KRT10	P13645	15.3 (2.3) [6]	13.4 (1.5) [6]	-1.92	0.115	1
KRT2	P35908	14.8 (2.6) [10]	14.1 (1.4) [5]	-0.70	0.504	1
LTBP2	G3V3X5	12.4 (1.2) [9]	13.1 (0.6) [8]	0.71	0.142	1
LY6H	O94772	11.4 (0.7) [9]	11 (0.6) [8]	-0.42	0.183	1
MFAP4	K7ES70	14.2 (1.2) [5]	13.4 (0.7) [5]	-0.88	0.19	1
MGAT1	P26572	14.2 (0.3) [8]	14.1 (0.2) [8]	-0.16	0.263	1
MRC1	P22897	15 (0.8) [11]	14.7 (0.5) [8]	-0.26	0.405	1
NPPC	P23582	13.7 (0.7) [7]	13.3 (0.2) [7]	-0.34	0.281	1
NRN1	A0A087WWT2	14.8 (0.5) [7]	14.5 (0.4) [8]	-0.25	0.346	1
NRP1	E7EX60	13.4 (0.5) [10]	13.4 (0.5) [9]	0.03	0.884	1
NSG1	A0A0A6YYJ2	10.8 (1.1) [7]	10.6 (0.9) [7]	-0.17	0.756	1
PCDHAC2	Q9Y5I4	14.5 (1.2) [5]	14.2 (2.2) [6]	-0.26	0.81	1

**Protein Name**	**Uniprot ID**	**SAH Weaned** Mean (SD) [N]	**SAH Shunted** Mean (SD) [N]	**log_2FC**	**P-value**	**Adj. P-value**
POMGNT1	Q8WZA1	13.8 (0.5) [9]	13.8 (1.1) [8]	0.01	0.986	1
PRELP	P51888	13.4 (1.1) [8]	13.2 (0.8) [8]	-0.26	0.603	1
PTPRD	P23468	14.1 (0.5) [9]	13.2 (0.7) [9]	-0.84	0.011	1
RGMB	J3KNF6	14 (0.9) [8]	13.1 (1.4) [5]	-0.91	0.249	1
RNASE4	P34096	13.4 (0.8) [6]	12.9 (0.6) [5]	-0.46	0.299	1
RNASE6	Q93091	14.1 (1.1) [9]	13.5 (0.6) [8]	-0.52	0.245	1
RP1	P56715	14.3 (0.4) [6]	14.9 (0.6) [5]	0.57	0.103	1
SEMA4B	J3KNP4	13.3 (0.3) [6]	13.1 (0.8) [8]	-0.20	0.538	1
SEZ6L	B0QYH4	13.5 (0.8) [9]	13.3 (0.9) [9]	-0.20	0.623	1
SLC3A2	F5GZS6	12.7 (0.4) [9]	13.1 (0.4) [7]	0.39	0.051	1
SORCS3	Q9UPU3	12.8 (0.8) [7]	12.3 (0.8) [8]	-0.53	0.205	1
SPOCK3	Q9BQ16	14.4 (1) [9]	13.5 (0.6) [7]	-0.86	0.043	1
STAB1	Q9NY15	12.7 (0.7) [6]	12.4 (0.5) [6]	-0.32	0.363	1
SUSD5	O60279	12.4 (0.5) [8]	12.6 (0.6) [9]	0.20	0.478	1
TMEM132A	Q24JP5	14 (0.8) [5]	12.8 (0.5) [5]	-1.12	0.037	1
VSTM2A	B5MCX6	14 (0.9) [9]	13.5 (1.7) [7]	-0.44	0.554	1
VSTM2B	A6NLU5	12.6 (0.7) [7]	13 (0.8) [7]	0.38	0.375	1
ECM2	O94769	14.1 (0.7) [9]	13.9 (0.6) [9]	-0.14	0.641	1
EFCAB14	O75071	14 (0.4) [8]	13.5 (0.8) [9]	-0.54	0.095	1
FMOD	Q06828	13.5 (1.1) [8]	13.4 (0.4) [9]	-0.12	0.782	1
ENOPH1	A0A0C4DGY8	13.5 (1) [5]	12.6 (0.3) [7]	-0.87	0.117	1
FBLN5	G3V4U0	14.1 (0.8) [9]	14.6 (0.8) [9]	0.57	0.15	1
FBN1	P35555	14 (1.3) [5]	14.8 (2) [7]	0.84	0.397	1
GANAB	Q14697	12.9 (0.5) [8]	12.3 (0.7) [9]	-0.53	0.094	1
CAPG	P40121	14.2 (1) [7]	14.5 (0.3) [5]	0.31	0.464	1
ANXA2	P07355	13.7 (2) [10]	12.2 (2.3) [7]	-1.44	0.199	1
FABP3	P05413	13.8 (0.8) [5]	13.6 (1.2) [5]	-0.20	0.775	1
ITM2B	Q9Y287	13.8 (1.2) [8]	12.2 (1.6) [8]	-1.61	0.041	1
KHSRP	A0A087WTP3	14 (0.7) [6]	14 (1.5) [5]	0.01	0.988	1
PPIC	P45877	14.5 (0.5) [8]	14.3 (0.3) [6]	-0.28	0.252	1
RNH1	P13489	14.7 (1.1) [9]	14.2 (0.9) [6]	-0.51	0.358	1
SDF4	Q9BRK5	13.3 (1) [9]	13.1 (0.5) [7]	-0.18	0.656	1
FRRS1L	Q9P0K9	10.2 (0.8) [6]	10.8 (2.5) [5]	0.57	0.64	1
SPON1	Q9HCB6	14 (0.6) [8]	13.1 (0.7) [6]	-0.94	0.02	1
SHISA6	Q6ZSJ9	13.8 (0.5) [6]	13.5 (0.5) [7]	-0.31	0.258	1
ADAMTS1	Q9UHI8	14.5 (0.4) [7]	14.8 (0.4) [5]	0.23	0.334	1
ALDH1A1	P00352	14 (1.2) [5]	13.5 (0.8) [5]	-0.48	0.484	1
GSR	P00390	14 (0.8) [6]	13.8 (0.4) [5]	-0.17	0.642	1
PODXL2	Q9NZ53	12 (0.6) [6]	11.9 (1.2) [5]	-0.10	0.87	1
S100A4	P26447	14.3 (2.3) [8]	11.3 (3) [5]	-3.07	0.09	1

SAH: subarachnoid hemorrhage; SD: standard deviation; N: number of patients; log_2FC: log_2(fold change).

Supplementary Table 5: Proteins not identified as possible predictors of shunt dependency in SAH patients.

Protein Name	Uniprot ID	Time	SAH Weaned<br><br>Mean (SD) [N]	SAH Shunted<br><br>Mean (SD) [N]	AUC (95%CI)	Cut-off	Sens.	Spec.
A1BG	P04217-2	change	0.05 (0.07) [12]	0.03 (0.04) [10]	0.56 (0.3-0.81)	0.07	0.90	0.42
A1BG	P04217-2	end	16.92 (0.43) [12]	17.18 (0.39) [10]	0.66 (0.42-0.89)	16.75	1.00	0.33
A1BG	P04217-2	start	17.51 (0.68) [12]	17.83 (0.65) [11]	0.64 (0.41-0.88)	17.69	0.73	0.58
A2M	P01023	change	0.08 (0.09) [12]	0.03 (0.09) [10]	0.52 (0.25-0.78)	0.12	1.00	0.25
A2M	P01023	start	18.06 (0.69) [12]	18.31 (1.02) [11]	0.66 (0.41-0.91)	18.46	0.73	0.75
A2M	P01023	end	17.13 (0.4) [12]	17.42 (0.72) [10]	0.57 (0.31-0.82)	16.80	1.00	0.25
ABHD14B	Q96IU4	end	13.76 (0.82) [9]	13.3 (0.88) [9]	0.69 (0.42-0.96)	13.65	0.89	0.56
ABHD14B	Q96IU4	start	13.26 (0.93) [8]	12.87 (1.2) [6]	0.56 (0.21-0.91)	12.11	0.33	1.00
ACAT2	Q9BWD1	end	12.15 (1.12) [7]	11.88 (0.34) [5]	0.51 (0.14-0.89)	12.12	0.80	0.57
ACSBG2	Q5FVE4	end	21.02 (0.79) [12]	20.91 (0.53) [9]	0.61 (0.35-0.87)	21.13	0.78	0.58
ACSBG2	Q5FVE4	change	-0.08 (0.15) [10]	-0.04 (0.03) [6]	0.58 (0.29-0.88)	-0.09	1.00	0.30
ACSBG2	Q5FVE4	start	20.36 (0.71) [10]	20.18 (0.65) [8]	0.64 (0.34-0.94)	20.09	0.75	0.80
ACTA2	P62736	end	17.32 (1.36) [12]	16.96 (0.55) [9]	0.67 (0.41-0.92)	17.53	0.89	0.67
ACTA2	P62736	change	-0.06 (0.1) [12]	-0.07 (0.08) [5]	0.58 (0.28-0.89)	-0.08	0.60	0.67
ACTA2	P62736	start	16.61 (0.59) [12]	16.07 (1.17) [7]	0.69 (0.37-1)	16.21	0.71	0.83

Protein Name	Uniprot ID	Time	SAH Weaned<br><br>Mean (SD) [N]	SAH Shunted<br><br>Mean (SD) [N]	AUC (95%CI)	Cut-off	Sens.	Spec.
ACTB	P60709	change	-0.06 (0.11) [12]	-0.04 (0.11) [10]	0.57 (0.31-0.83)	-0.03	0.80	0.58
ACTB	P60709	end	17.55 (1.3) [12]	17.27 (0.94) [10]	0.67 (0.42-0.91)	17.03	0.60	0.83
ACTB	P60709	start	16.94 (0.68) [12]	16.34 (1.89) [11]	0.76 (0.53-0.98)	16.37	0.73	0.83
ACTBL2	Q562R1	change	-0.06 (0.13) [12]	-0.08 (0.09) [8]	0.64 (0.37-0.9)	-0.02	0.88	0.50
ACTBL2	Q562R1	end	19.51 (1.41) [12]	19.11 (0.7) [9]	0.73 (0.49-0.97)	19.22	0.78	0.83
ACTBL2	Q562R1	start	18.85 (0.75) [12]	17.65 (1.36) [10]	0.78 (0.54-1)	18.34	0.80	0.83
ADA2	B4E3Q4	end	13.78 (0.69) [9]	13.34 (0.57) [7]	0.68 (0.4-0.96)	13.66	0.86	0.56
ADAM22	F8WAD8	end	11.05 (1.29) [8]	11.01 (1.34) [7]	0.52 (0.19-0.85)	9.99	0.43	0.88
ADAM29	Q9UKF5	end	15.02 (0.78) [6]	15.33 (0.92) [9]	0.61 (0.3-0.92)	15.47	0.56	0.83
ADAMTS1	Q9UHI8	end	14.52 (0.35) [7]	14.75 (0.41) [5]	0.69 (0.32-1)	14.79	0.60	0.86
ADGRB2	A2A3C1	end	14.73 (0.81) [9]	14.02 (0.59) [6]	0.76 (0.49-1)	13.99	0.67	0.89
ADGRL1	O94910	end	13.8 (0.49) [5]	13.76 (0.8) [7]	0.57 (0.21-0.93)	13.65	0.57	0.80
ADIPOQ	Q15848	change	0.12 (0.04) [5]	0.08 (0.11) [6]	0.63 (0.27-1)	0.06	0.33	1.00
ADIPOQ	Q15848	end	11.58 (0.47) [8]	12.23 (0.68) [8]	0.8 (0.55-1)	12.23	0.62	1.00
ADIPOQ	Q15848	start	13.82 (0.92) [7]	14.23 (1.53) [7]	0.65 (0.31-1)	14.39	0.71	0.86
AEBP1	Q8IUX7	end	13.47 (0.69) [8]	13.42 (0.59) [8]	0.53 (0.22-0.84)	13.62	0.75	0.50
AFM	P43652	start	15.72 (0.46) [12]	15.83 (0.5) [10]	0.58 (0.33-0.84)	15.78	0.70	0.58
AFM	P43652	end	15.28 (0.55) [12]	15.66 (1.26) [10]	0.52 (0.27-0.78)	14.77	1.00	0.25

Protein Name	Uniprot ID	Time	SAH Weaned<br><br>Mean (SD) [N]	SAH Shunted<br><br>Mean (SD) [N]	AUC (95%CI)	Cut-off	Sens.	Spec.
AFM	P43652	change	0.04 (0.06) [12]	0 (0.08) [9]	0.56 (0.29-0.82)	0.05	1.00	0.33
AGA	P20933	end	13.06 (1.49) [9]	13.85 (0.96) [5]	0.64 (0.21-1)	14.30	0.60	1.00
AGRN	O00468-6	start	13.33 (0.87) [5]	13.48 (1.39) [5]	0.56 (0.13-0.99)	14.44	0.40	1.00
AGT	P01019	end	16.49 (0.38) [12]	16.44 (0.27) [10]	0.54 (0.29-0.8)	16.52	0.80	0.42
AGT	P01019	start	16.04 (0.44) [12]	16.66 (0.67) [11]	0.86 (0.69-1)	16.26	0.82	0.83
AGT	P01019	change	-0.03 (0.03) [12]	0.01 (0.04) [10]	0.83 (0.65-1)	-0.02	0.90	0.75
AHSG	P02765	change	0.07 (0.09) [12]	0.04 (0.2) [10]	0.61 (0.35-0.87)	0.05	0.70	0.67
AHSG	P02765	end	16.88 (0.55) [12]	17.61 (1.96) [10]	0.65 (0.41-0.89)	16.88	0.80	0.58
AHSG	P02765	start	17.73 (0.69) [12]	18.5 (2.41) [11]	0.57 (0.32-0.82)	18.57	0.27	1.00
ALB	P02768	change	0.05 (0.09) [12]	0.03 (0.06) [10]	0.52 (0.26-0.77)	0.04	0.70	0.50
ALB	P02768	end	19.27 (0.5) [12]	19.49 (0.58) [10]	0.59 (0.34-0.84)	19.33	0.60	0.67
ALB	P02768	start	19.76 (0.46) [12]	20.04 (0.76) [11]	0.61 (0.35-0.87)	20.19	0.45	0.92
ALCAM	Q13740	end	14.7 (0.59) [11]	14.33 (0.38) [9]	0.73 (0.49-0.97)	14.64	0.78	0.73
ALDH1A1	P00352	end	14 (1.19) [5]	13.52 (0.83) [5]	0.72 (0.32-1)	14.03	0.80	0.80
ALDOA	P04075	change	-0.07 (0.11) [12]	-0.04 (0.17) [9]	0.6 (0.34-0.87)	-0.04	0.78	0.58
ALDOA	P04075	start	14.91 (0.85) [12]	14.98 (2.92) [10]	0.67 (0.4-0.93)	14.09	0.60	0.83
ALDOA	P04075	end	15.64 (0.72) [12]	15.82 (1.14) [10]	0.56 (0.29-0.83)	15.62	0.60	0.75

**Protein Name**	**Uniprot ID**	**Time**	**SAH Weaned** Mean (SD) [N]	**SAH Shunted** Mean (SD) [N]	**AUC (95%CI)**	**Cut-off**	**Sens.**	**Spec.**
ALDOC	P09972	start	14.16 (1.24) [10]	13.95 (1.03) [5]	0.6 (0.27-0.93)	14.96	1.00	0.40
ALDOC	P09972	end	14.47 (0.49) [12]	14.69 (0.66) [9]	0.65 (0.38-0.92)	14.48	0.78	0.67
AMBP	P02760	start	16.92 (0.67) [12]	17.04 (0.96) [11]	0.61 (0.35-0.88)	17.32	0.64	0.83
AMBP	P02760	change	0.07 (0.09) [12]	0.05 (0.04) [10]	0.53 (0.27-0.79)	0.10	0.90	0.33
AMBP	P02760	end	16.08 (0.64) [12]	15.94 (0.43) [10]	0.54 (0.29-0.8)	16.56	1.00	0.25
ANXA2	P07355	end	13.67 (1.99) [10]	12.23 (2.26) [7]	0.66 (0.33-0.98)	12.20	0.71	0.80
ANXA5	P08758	end	15.07 (1.35) [11]	14.14 (0.97) [7]	0.77 (0.46-1)	14.03	0.71	1.00
APCS	P02743	end	15.27 (1.63) [11]	14.95 (0.75) [9]	0.52 (0.23-0.81)	14.17	1.00	0.45
APCS	P02743	change	0.18 (0.21) [11]	0.15 (0.11) [8]	0.53 (0.25-0.82)	0.12	0.88	0.45
APCS	P02743	start	17.25 (1.38) [12]	17.93 (1.9) [10]	0.68 (0.42-0.94)	18.90	0.50	0.92
APLP1	B7Z4G8	change	-0.08 (0.07) [10]	-0.05 (0.06) [8]	0.62 (0.33-0.92)	-0.06	0.75	0.70
APLP1	B7Z4G8	start	14.69 (0.82) [10]	14.26 (1.49) [10]	0.59 (0.32-0.86)	15.26	0.90	0.40
APLP1	B7Z4G8	end	15.72 (0.72) [12]	15.42 (0.71) [9]	0.65 (0.39-0.9)	15.67	0.78	0.67
APLP2	Q06481	start	13.74 (1.11) [8]	14.75 (0.71) [6]	0.77 (0.5-1)	13.91	1.00	0.50
APLP2	Q06481	end	14.36 (0.97) [12]	14.26 (0.85) [8]	0.55 (0.28-0.82)	15.50	1.00	0.17
APLP2	Q06481	change	-0.07 (0.07) [8]	0.02 (0.06) [5]	0.85 (0.62-1)	-0.01	0.80	0.88
APOA1	P02647	start	19.79 (0.99) [12]	20.29 (1.15) [11]	0.69 (0.45-0.93)	20.40	0.73	0.67

**Protein Name**	**Uniprot ID**	**Time**	**SAH Weaned**<br><br>Mean (SD) [N]	**SAH Shunted**<br><br>Mean (SD) [N]	**AUC (95%CI)**	**Cut-off**	**Sens.**	**Spec.**
APOA1	P02647	end	18.24 (0.86) [12]	18.94 (1.13) [10]	0.77 (0.56-0.98)	18.03	1.00	0.58
APOA1	P02647	change	0.13 (0.13) [12]	0.05 (0.12) [10]	0.67 (0.42-0.91)	0.12	0.80	0.67
APOA2	P02652	end	17.56 (1.39) [12]	17.6 (0.55) [10]	0.53 (0.28-0.79)	16.70	1.00	0.33
APOA2	P02652	change	0.13 (0.16) [12]	0.05 (0.07) [10]	0.67 (0.43-0.9)	0.11	0.90	0.50
APOA2	P02652	start	19.02 (0.8) [12]	18.95 (1.04) [11]	0.52 (0.27-0.78)	19.68	0.36	0.83
APOA4	P06727	change	0.1 (0.11) [12]	0.03 (0.09) [9]	0.69 (0.46-0.93)	0.11	1.00	0.42
APOA4	P06727	start	17.32 (0.85) [12]	17.48 (0.95) [10]	0.55 (0.29-0.81)	17.10	0.80	0.42
APOA4	P06727	end	16.1 (0.77) [12]	16.39 (1.2) [10]	0.57 (0.31-0.82)	16.44	0.40	0.83
APOB	P04114	end	15.78 (1.6) [12]	16.19 (0.85) [9]	0.61 (0.36-0.86)	15.71	0.78	0.58
APOB	P04114	start	18.45 (1.47) [12]	19.16 (2.65) [11]	0.64 (0.4-0.89)	18.96	0.82	0.58
APOB	P04114	change	0.22 (0.25) [12]	0.16 (0.16) [9]	0.55 (0.28-0.81)	0.23	0.89	0.42
APOC1	K7ERI9	change	0.16 (0.21) [12]	0.13 (0.1) [8]	0.53 (0.26-0.81)	0.18	0.62	0.67
APOC1	K7ERI9	end	15.88 (1.22) [12]	15.78 (0.46) [9]	0.55 (0.28-0.81)	15.08	1.00	0.33
APOC1	K7ERI9	start	17.72 (1.5) [12]	18.57 (1.82) [10]	0.7 (0.45-0.95)	18.89	0.70	0.83
APOC3	B0YIW2	start	19.29 (2.06) [12]	20.24 (2.35) [10]	0.66 (0.4-0.91)	20.48	0.70	0.75
APOC3	B0YIW2	change	0.2 (0.28) [12]	0.14 (0.14) [8]	0.55 (0.29-0.82)	0.27	1.00	0.33

**Protein Name**	**Uniprot ID**	**Time**	**SAH Weaned** Mean (SD) [N]	**SAH Shunted** Mean (SD) [N]	**AUC (95%CI)**	**Cut-off**	**Sens.**	**Spec.**
APOC3	B0YIW2	end	17.06 (1.83) [12]	17.27 (1.25) [9]	0.58 (0.33-0.84)	17.55	0.56	0.67
APOC4-APOC2	A0A024R0T9	end	15.16 (1.7) [12]	15.21 (0.98) [9]	0.56 (0.31-0.82)	13.90	1.00	0.25
APOC4-APOC2	A0A024R0T9	change	0.18 (0.24) [12]	0.13 (0.13) [8]	0.53 (0.26-0.8)	0.27	1.00	0.33
APOC4-APOC2	A0A024R0T9	start	17.29 (1.79) [12]	18.09 (2.36) [10]	0.64 (0.38-0.9)	18.90	0.50	0.92
APOD	C9JF17	change	-0.05 (0.08) [12]	-0.07 (0.09) [9]	0.59 (0.33-0.85)	-0.03	0.78	0.58
APOD	C9JF17	start	16.52 (0.95) [12]	16.46 (0.73) [10]	0.53 (0.28-0.79)	17.03	0.90	0.42
APOD	C9JF17	end	17.19 (0.65) [12]	17.8 (1.72) [10]	0.63 (0.38-0.88)	17.21	0.80	0.58
APOE	P02649	start	16.54 (0.7) [12]	16.56 (0.85) [11]	0.52 (0.27-0.77)	17.34	0.91	0.25
APOE	P02649	end	17.65 (0.6) [12]	17.71 (0.55) [10]	0.52 (0.26-0.78)	17.19	0.30	0.92
APOE	P02649	change	-0.08 (0.05) [12]	-0.06 (0.06) [10]	0.59 (0.34-0.85)	-0.04	0.40	0.92
APOF	Q13790	start	16.2 (0.97) [7]	15.88 (0.63) [8]	0.59 (0.26-0.91)	16.11	0.62	0.71
APOH	P02749	end	15.87 (0.7) [12]	15.59 (0.66) [9]	0.63 (0.37-0.89)	15.99	0.89	0.58
APOH	P02749	start	16.29 (0.6) [12]	16.69 (0.79) [10]	0.68 (0.44-0.93)	16.71	0.60	0.83
APOH	P02749	change	0.04 (0.06) [12]	0.05 (0.03) [8]	0.69 (0.42-0.95)	0.03	0.88	0.67
APOL1	O14791	start	15.95 (1.46) [12]	16.15 (1.87) [10]	0.59 (0.33-0.86)	16.51	0.70	0.58
APOL1	O14791	change	0.15 (0.2) [11]	0.08 (0.1) [8]	0.57 (0.3-0.84)	0.13	0.75	0.55

Protein Name	Uniprot ID	Time	**SAH Weaned** Mean (SD) [N]	**SAH Shunted** Mean (SD) [N]	AUC (95%CI)	Cut-off	Sens.	Spec.
APOL1	O14791	end	14.22 (1.8) [11]	14.41 (0.5) [9]	0.67 (0.41-0.93)	13.77	1.00	0.55
APOM	O95445	end	15.57 (1.39) [10]	15.83 (0.72) [9]	0.66 (0.38-0.93)	15.04	1.00	0.50
APOM	O95445	start	17.53 (1.08) [12]	17.92 (1.67) [10]	0.67 (0.41-0.94)	18.26	0.70	0.75
APOM	O95445	change	0.17 (0.18) [10]	0.1 (0.11) [8]	0.6 (0.32-0.88)	0.16	0.88	0.50
APP	P05067	end	14.07 (0.86) [12]	13.71 (0.78) [9]	0.69 (0.44-0.95)	13.91	0.78	0.75
APP	P05067	start	13.48 (0.83) [11]	13.33 (1.14) [9]	0.61 (0.32-0.89)	13.56	0.78	0.64
APP	P05067	change	-0.05 (0.07) [11]	-0.02 (0.03) [7]	0.66 (0.4-0.92)	-0.06	1.00	0.45
ARPC4-TTLL3	A0A0A6YYG9	end	16.43 (0.41) [12]	16.31 (0.32) [9]	0.6 (0.34-0.86)	16.28	0.67	0.67
ARPC4-TTLL3	A0A0A6YYG9	start	16.76 (0.52) [12]	16.84 (0.59) [10]	0.58 (0.32-0.84)	16.95	0.50	0.83
ARPC4-TTLL3	A0A0A6YYG9	change	0.02 (0.04) [12]	0.02 (0.02) [8]	0.51 (0.25-0.77)	0.05	1.00	0.25
ART3	E7ESB3	end	14.11 (0.52) [8]	13.59 (0.39) [5]	0.8 (0.55-1)	13.95	1.00	0.62
ATP1A1	P05023	end	13.67 (0.35) [5]	13.89 (0.37) [6]	0.67 (0.3-1)	13.98	0.50	1.00
ATP6AP1	Q15904	change	-0.07 (0.08) [9]	-0.04 (0.04) [6]	0.63 (0.31-0.95)	-0.08	1.00	0.33
ATP6AP1	Q15904	start	13.77 (0.82) [9]	13.66 (1.11) [8]	0.54 (0.23-0.86)	13.21	0.50	0.78
ATP6AP1	Q15904	end	14.33 (0.94) [12]	14.06 (0.56) [9]	0.65 (0.39-0.9)	14.33	0.89	0.58
ATRN	O75882	change	0.04 (0.04) [8]	0.04 (0.06) [6]	0.5 (0.16-0.84)	0.02	0.83	0.38
ATRN	O75882	start	14.6 (0.53) [11]	14.77 (0.51) [9]	0.61 (0.34-0.87)	14.63	0.78	0.55

Protein Name	Uniprot ID	Time	SAH Weaned<br><br>Mean (SD) [N]	SAH Shunted<br><br>Mean (SD) [N]	AUC (95%CI)	Cut-off	Sens.	Spec.
ATRN	O75882	end	14.1 (0.46) [9]	13.73 (0.74) [6]	0.72 (0.39-1)	13.39	0.50	1.00
AZGP1	P25311	start	16.82 (0.57) [12]	17.14 (0.53) [10]	0.68 (0.44-0.91)	17.01	0.60	0.75
AZGP1	P25311	change	0 (0.06) [12]	0 (0.03) [9]	0.54 (0.26-0.81)	-0.04	1.00	0.42
AZGP1	P25311	end	16.89 (0.65) [12]	17.23 (0.72) [10]	0.62 (0.37-0.86)	17.32	0.50	0.75
B2M	P61769	start	15.26 (2.48) [12]	15.43 (1.42) [11]	0.54 (0.29-0.79)	15.64	0.64	0.58
B2M	P61769	change	-0.12 (0.17) [12]	-0.08 (0.09) [10]	0.61 (0.36-0.85)	-0.11	0.70	0.58
B2M	P61769	end	16.95 (1.21) [12]	17.08 (0.73) [10]	0.51 (0.25-0.77)	17.07	0.70	0.50
B4GALT1	P15291	end	14.17 (0.7) [6]	14.76 (0.28) [7]	0.79 (0.46-1)	14.19	1.00	0.67
B4GAT1	O43505	change	-0.06 (0.07) [10]	-0.11 (0.17) [7]	0.59 (0.27-0.9)	-0.10	0.43	0.90
B4GAT1	O43505	start	15.07 (1.11) [10]	14.44 (1.46) [8]	0.68 (0.4-0.95)	15.19	0.88	0.50
B4GAT1	O43505	end	15.97 (0.84) [12]	16.16 (2.31) [10]	0.63 (0.38-0.89)	15.64	0.70	0.75
BASP1	P80723	end	12.02 (1.16) [9]	12.27 (1.17) [9]	0.54 (0.26-0.83)	12.32	0.56	0.67
BCAN	Q96GW7	end	14.84 (1.32) [12]	14.57 (0.92) [9]	0.56 (0.29-0.82)	13.73	0.33	0.92
BCAN	Q96GW7	start	14.68 (1.02) [10]	14.62 (1.6) [7]	0.56 (0.23-0.88)	14.33	0.57	0.70
BCAN	Q96GW7	change	-0.02 (0.11) [10]	-0.01 (0.08) [5]	0.6 (0.26-0.94)	0.02	0.60	0.80
BCHE	P06276	start	14.89 (0.71) [6]	13.94 (1.89) [5]	0.63 (0.25-1)	13.49	0.40	1.00
BGN	P21810	end	13.95 (0.83) [9]	13.42 (1.04) [7]	0.71 (0.41-1)	13.74	0.86	0.78

**Protein Name**	**Uniprot ID**	**Time**	**SAH Weaned**<br><br>Mean (SD) [N]	**SAH Shunted**<br><br>Mean (SD) [N]	**AUC (95%CI)**	**Cut-off**	**Sens.**	**Spec.**
BLVRB	P30043	end	16.35 (1.54) [11]	15.52 (1.67) [10]	0.67 (0.42-0.93)	16.00	0.80	0.64
BPGM	P07738	end	15.6 (0.97) [8]	15 (0.98) [6]	0.69 (0.39-0.99)	15.39	0.67	0.75
BTD	P43251	end	15.49 (0.56) [12]	15.28 (0.23) [9]	0.62 (0.37-0.87)	15.74	1.00	0.42
BTD	P43251	start	15.44 (0.2) [12]	15.65 (0.54) [10]	0.64 (0.37-0.91)	15.76	0.50	1.00
BTD	P43251	change	0 (0.03) [12]	0.02 (0.02) [8]	0.7 (0.46-0.94)	-0.01	1.00	0.42
C16orf89	A0A0A0MT71	end	13.82 (0.53) [9]	13.86 (0.4) [9]	0.53 (0.24-0.82)	13.60	0.78	0.44
C1QA	P02745	start	15.44 (0.59) [12]	15.17 (0.4) [10]	0.63 (0.39-0.88)	15.61	0.90	0.50
C1QA	P02745	change	0 (0.06) [12]	-0.03 (0.04) [9]	0.6 (0.34-0.86)	-0.03	0.44	0.83
C1QA	P02745	end	15.42 (0.66) [12]	15.73 (0.65) [10]	0.63 (0.39-0.88)	16.04	0.40	0.92
C1QB	D6R934	change	-0.04 (0.08) [12]	-0.04 (0.06) [9]	0.51 (0.25-0.77)	0.02	0.89	0.33
C1QB	D6R934	end	16.25 (1.09) [12]	16.4 (1.03) [10]	0.51 (0.24-0.77)	15.67	0.90	0.42
C1QB	D6R934	start	15.87 (0.57) [12]	15.58 (0.69) [10]	0.64 (0.39-0.89)	15.62	0.60	0.75
C1QC	P02747	end	17.74 (0.8) [12]	17.8 (0.61) [9]	0.52 (0.26-0.78)	17.46	0.89	0.42
C1QC	P02747	start	17.59 (0.55) [12]	17.53 (0.55) [10]	0.52 (0.26-0.78)	17.80	0.80	0.33
C1QC	P02747	change	-0.03 (0.11) [12]	-0.02 (0.04) [8]	0.53 (0.27-0.8)	-0.05	0.88	0.33
C1R	B4DPQ0	end	15.99 (0.49) [12]	16.12 (0.42) [10]	0.58 (0.33-0.84)	15.91	0.70	0.58

Protein Name	Uniprot ID	Time	SAH Weaned<br><br>Mean (SD) [N]	SAH Shunted<br><br>Mean (SD) [N]	AUC (95%CI)	Cut-off	Sens.	Spec.
C1R	B4DPQ0	start	16.05 (0.39) [12]	15.91 (0.37) [10]	0.57 (0.31-0.82)	16.23	0.90	0.33
C1R	B4DPQ0	change	-0.01 (0.06) [12]	-0.01 (0.03) [9]	0.54 (0.28-0.8)	0.01	0.89	0.42
C1RL	Q9NZP8	start	14.81 (1.1) [10]	14.87 (0.74) [10]	0.52 (0.24-0.8)	15.60	1.00	0.30
C1RL	Q9NZP8	change	0.06 (0.06) [7]	0.04 (0.03) [7]	0.61 (0.28-0.95)	0.09	1.00	0.43
C1RL	Q9NZP8	end	13.95 (0.62) [9]	14.17 (0.52) [8]	0.64 (0.35-0.93)	14.08	0.88	0.56
C1S	P09871	change	-0.01 (0.06) [12]	-0.01 (0.03) [9]	0.56 (0.3-0.83)	-0.01	0.56	0.75
C1S	P09871	start	16.36 (0.43) [12]	16.29 (0.36) [10]	0.58 (0.32-0.85)	16.68	1.00	0.42
C1S	P09871	end	16.44 (0.54) [12]	16.52 (0.4) [10]	0.52 (0.26-0.79)	16.20	0.90	0.42
C2	P06681	start	15.5 (0.46) [12]	15.5 (0.54) [10]	0.52 (0.25-0.78)	15.58	0.60	0.58
C2	P06681	change	0 (0.05) [12]	0 (0.02) [8]	0.55 (0.28-0.82)	0.00	0.75	0.58
C2	P06681	end	15.49 (0.43) [12]	15.48 (0.55) [9]	0.5 (0.23-0.77)	15.50	0.78	0.42
C2orf40	B8ZZE5	change	-0.06 (0.09) [9]	-0.03 (0.1) [5]	0.67 (0.31-1)	0.03	0.40	1.00
C2orf40	B8ZZE5	end	15.88 (1.27) [11]	15.02 (0.53) [9]	0.75 (0.51-0.98)	15.44	0.89	0.73
C2orf40	B8ZZE5	start	15.03 (1.26) [9]	14.44 (1.38) [7]	0.62 (0.32-0.92)	15.88	1.00	0.33
C3	P01024	change	0.03 (0.05) [12]	0.01 (0.08) [10]	0.54 (0.29-0.8)	0.04	0.90	0.33
C3	P01024	start	17.78 (0.44) [12]	18.07 (0.9) [11]	0.59 (0.35-0.84)	17.78	0.73	0.50

**Protein Name**	**Uniprot ID**	**Time**	**SAH Weaned**<br><br>Mean (SD) [N]	**SAH Shunted**<br><br>Mean (SD) [N]	**AUC (95%CI)**	**Cut-off**	**Sens.**	**Spec.**
C3	P01024	end	17.46 (0.29) [12]	17.8 (0.73) [10]	0.66 (0.42-0.9)	17.64	0.60	0.75
C4A	A0A0G2JPR0	change	-0.03 (0.04) [12]	0.01 (0.07) [9]	0.66 (0.41-0.9)	-0.05	1.00	0.33
C4A	A0A0G2JPR0	end	16.15 (0.45) [12]	15.53 (0.85) [10]	0.74 (0.51-0.97)	15.68	0.60	0.92
C4A	A0A0G2JPR0	start	15.7 (0.65) [12]	15.67 (1.01) [10]	0.53 (0.27-0.79)	16.20	0.40	0.83
C4B	P0C0L5	start	16.84 (0.32) [12]	17 (0.24) [11]	0.64 (0.4-0.87)	16.63	1.00	0.33
C4B	P0C0L5	change	-0.02 (0.03) [12]	0 (0.03) [10]	0.66 (0.4-0.92)	-0.02	0.90	0.67
C4B	P0C0L5	end	17.06 (0.3) [12]	17.02 (0.48) [10]	0.52 (0.24-0.79)	16.95	0.60	0.67
C4BPA	P04003	change	0.21 (0.22) [10]	0.1 (0.14) [8]	0.56 (0.27-0.86)	0.24	1.00	0.40
C4BPA	P04003	end	13.59 (1.58) [10]	13.79 (0.76) [9]	0.52 (0.24-0.81)	13.20	0.89	0.40
C4BPA	P04003	start	16.11 (1.24) [12]	16.09 (2.68) [10]	0.52 (0.26-0.79)	18.33	0.20	1.00
C5	P01031	end	14.74 (0.49) [12]	14.97 (0.29) [9]	0.68 (0.42-0.93)	14.77	0.89	0.67
C5	P01031	start	15.74 (0.63) [12]	15.8 (0.83) [10]	0.58 (0.32-0.84)	16.16	0.50	0.75
C5	P01031	change	0.08 (0.09) [12]	0.04 (0.04) [8]	0.64 (0.36-0.91)	0.07	1.00	0.58
C6	P13671	end	15.26 (0.61) [12]	15.18 (0.49) [9]	0.51 (0.24-0.78)	15.47	0.44	0.75
C6	P13671	start	16.21 (0.71) [12]	16.55 (0.79) [10]	0.67 (0.43-0.92)	16.63	0.70	0.75
C6	P13671	change	0.07 (0.08) [12]	0.06 (0.04) [8]	0.55 (0.28-0.82)	0.10	1.00	0.42

**Protein Name**	**Uniprot ID**	**Time**	**SAH Weaned** Mean (SD) [N]	**SAH Shunted** Mean (SD) [N]	**AUC (95%CI)**	**Cut-off**	**Sens.**	**Spec.**
C7	P10643	start	15.8 (0.66) [12]	15.9 (0.63) [10]	0.54 (0.28-0.8)	15.34	0.90	0.33
C7	P10643	end	16.4 (0.76) [12]	16.56 (0.6) [10]	0.58 (0.32-0.83)	16.55	0.70	0.58
C7	P10643	change	-0.06 (0.11) [12]	-0.03 (0.03) [9]	0.59 (0.34-0.85)	-0.06	0.89	0.42
C8A	P07357	start	16.01 (0.61) [12]	16.06 (0.87) [11]	0.58 (0.32-0.84)	16.09	0.73	0.67
C8A	P07357	end	15.19 (0.44) [12]	15.41 (0.71) [10]	0.61 (0.35-0.86)	15.10	0.70	0.58
C8A	P07357	change	0.07 (0.09) [12]	0.03 (0.08) [10]	0.52 (0.25-0.79)	0.02	0.40	0.83
C8B	F5GY80	end	13.97 (0.38) [11]	14.08 (0.34) [9]	0.59 (0.32-0.85)	13.69	1.00	0.27
C8B	F5GY80	start	14.71 (0.57) [12]	14.79 (0.63) [10]	0.54 (0.28-0.8)	15.14	0.40	0.83
C8B	F5GY80	change	0.06 (0.08) [11]	0.03 (0.03) [8]	0.53 (0.26-0.81)	0.07	1.00	0.18
C8G	P07360	end	14.2 (0.49) [10]	13.65 (0.39) [6]	0.83 (0.63-1)	14.10	1.00	0.70
C8G	P07360	start	14.66 (0.4) [9]	14.61 (0.62) [7]	0.51 (0.18-0.84)	14.80	0.57	0.67
C9	P02748	end	15.62 (0.5) [12]	16.03 (0.95) [10]	0.67 (0.43-0.91)	15.84	0.70	0.67
C9	P02748	start	16.04 (0.51) [12]	16.08 (0.49) [10]	0.51 (0.25-0.77)	15.95	0.70	0.50
C9	P02748	change	0.03 (0.06) [12]	0 (0.05) [9]	0.63 (0.38-0.88)	0.06	1.00	0.42
CA1	P00915	start	17.02 (1.16) [10]	17.17 (3.66) [9]	0.6 (0.29-0.91)	15.83	0.56	0.90
CA1	P00915	change	-0.13 (0.18) [10]	-0.03 (0.16) [8]	0.6 (0.32-0.88)	-0.21	1.00	0.30

Protein Name	Uniprot ID	Time	SAH Weaned<br><br>Mean (SD) [N]	SAH Shunted<br><br>Mean (SD) [N]	AUC (95%CI)	Cut-off	Sens.	Spec.
CA1	P00915	end	18.54 (1.42) [11]	18.18 (2.06) [10]	0.6 (0.34-0.86)	19.04	0.80	0.45
CA2	P00918	start	15.14 (1.22) [11]	16.26 (4.36) [8]	0.52 (0.22-0.82)	14.33	0.50	0.73
CA2	P00918	change	-0.02 (0.17) [10]	0.07 (0.32) [7]	0.61 (0.31-0.92)	-0.06	0.86	0.60
CA2	P00918	end	15.25 (1.63) [10]	15.28 (3.03) [9]	0.59 (0.31-0.87)	15.66	0.78	0.50
CACNA2D1	P54289	end	13.86 (0.66) [9]	13.13 (1) [9]	0.72 (0.45-0.98)	12.88	0.56	1.00
CACNA2D1	P54289	start	13.64 (0.68) [5]	14.45 (1.16) [6]	0.7 (0.35-1)	14.60	0.50	1.00
CADM1	A0A087X0T8	end	15.33 (0.72) [10]	14.6 (0.54) [9]	0.87 (0.68-1)	15.24	1.00	0.80
CADM2	Q8N3J6	end	14.15 (0.56) [7]	13.37 (0.58) [6]	0.81 (0.56-1)	13.91	0.83	0.71
CADM3	Q8N126	end	15.45 (1.16) [11]	14.86 (0.42) [9]	0.71 (0.47-0.95)	15.20	0.89	0.55
CADM4	Q8NFZ8	end	15.8 (0.48) [11]	15.12 (0.23) [9]	0.85 (0.64-1)	15.54	1.00	0.82
CALR	P27797	end	13.83 (1.04) [12]	13.55 (0.3) [9]	0.66 (0.41-0.9)	13.99	1.00	0.33
CAPG	P40121	end	14.22 (1) [7]	14.53 (0.34) [5]	0.66 (0.3-1)	14.34	0.80	0.71
CARTPT	Q16568	end	13.32 (0.34) [8]	12.93 (0.79) [7]	0.7 (0.37-1)	12.82	0.57	1.00
CARTPT	Q16568	start	12.65 (0.95) [7]	13.24 (0.67) [6]	0.71 (0.41-1)	11.95	1.00	0.43
CASP14	P31944	end	14.44 (0.94) [9]	14.07 (2.14) [7]	0.52 (0.19-0.86)	15.83	0.29	1.00
CASP14	P31944	start	14.63 (1.05) [8]	15.48 (1.11) [7]	0.71 (0.42-1)	15.14	0.71	0.75
CAT	P04040	end	15.43 (1.22) [12]	15.12 (1.58) [10]	0.58 (0.32-0.83)	16.27	0.90	0.33
CAT	P04040	start	14.12 (1.24) [9]	16.1 (3.58) [5]	0.6 (0.22-0.98)	16.98	0.40	1.00

**Protein Name**	**Uniprot ID**	**Time**	**SAH Weaned**<br><br>Mean (SD) [N]	**SAH Shunted**<br><br>Mean (SD) [N]	**AUC (95%CI)**	**Cut-off**	**Sens.**	**Spec.**
CAT	P04040	change	-0.07 (0.1) [9]	0 (0.2) [5]	0.56 (0.2-0.91)	-0.18	1.00	0.22
CBR1	P16152	start	15.56 (1.14) [10]	14.42 (0.52) [7]	0.84 (0.64-1)	14.83	0.86	0.80
CBR1	P16152	change	0.06 (0.12) [7]	0 (0.06) [5]	0.77 (0.47-1)	0.06	1.00	0.57
CBR1	P16152	end	14.45 (1.02) [9]	14.15 (0.7) [9]	0.6 (0.32-0.89)	15.06	1.00	0.33
CCP110	O43303	end	20.67 (0.8) [8]	21 (0.81) [6]	0.67 (0.36-0.97)	20.21	1.00	0.38
CD14	P08571	start	15.16 (1.48) [12]	15.12 (1.18) [10]	0.5 (0.24-0.76)	14.41	0.80	0.33
CD14	P08571	end	16.9 (1.17) [12]	17.13 (0.79) [9]	0.56 (0.29-0.82)	16.63	0.89	0.42
CD14	P08571	change	-0.16 (0.22) [12]	-0.1 (0.08) [8]	0.57 (0.31-0.83)	-0.16	0.88	0.42
CD163	C9JHR8	end	15.87 (1.48) [11]	14.83 (1.48) [9]	0.72 (0.48-0.96)	16.17	0.89	0.64
CD44	H0YD13	change	-0.08 (0.11) [10]	-0.05 (0.07) [7]	0.61 (0.33-0.9)	-0.11	1.00	0.40
CD44	H0YD13	end	16.24 (1.17) [10]	16.32 (0.46) [9]	0.54 (0.26-0.83)	17.12	1.00	0.30
CD44	H0YD13	start	15.36 (0.91) [12]	15.32 (1.23) [9]	0.51 (0.24-0.78)	15.77	0.78	0.42
CD59	E9PNW4	end	15.21 (0.79) [11]	14.63 (0.82) [7]	0.73 (0.48-0.97)	15.23	0.86	0.64
CD99	P14209	end	15.86 (0.82) [8]	15.81 (0.49) [9]	0.56 (0.24-0.87)	16.04	0.67	0.62
CD99L2	Q8TCZ2	end	16 (0.54) [11]	16.04 (0.57) [9]	0.51 (0.23-0.78)	15.24	1.00	0.18
CDH13	P55290	change	-0.04 (0.07) [11]	-0.05 (0.12) [7]	0.57 (0.27-0.87)	-0.03	0.71	0.64

**Protein Name**	**Uniprot ID**	**Time**	**SAH Weaned**<br>Mean (SD) [N]	**SAH Shunted**<br>Mean (SD) [N]	**AUC (95%CI)**	**Cut-off**	**Sens.**	**Spec.**
CDH13	P55290	end	14.6 (0.48) [12]	14.69 (1.21) [10]	0.57 (0.27-0.86)	14.08	0.50	1.00
CDH13	P55290	start	14.21 (0.65) [11]	14.06 (1.08) [8]	0.58 (0.28-0.88)	13.73	0.50	0.82
CDH2	P19022	end	15.73 (0.44) [11]	15.39 (0.38) [9]	0.73 (0.5-0.96)	15.80	0.89	0.55
CDH2	P19022	start	14.7 (0.88) [6]	14.75 (1.21) [5]	0.53 (0.14-0.93)	15.07	0.80	0.50
CDH4	P55283	end	12.82 (0.63) [7]	12.32 (0.28) [6]	0.83 (0.55-1)	12.78	1.00	0.71
CDH6	D6RF86	end	13.27 (0.75) [8]	12.77 (0.38) [8]	0.78 (0.52-1)	13.35	1.00	0.50
CFD	K7ERG9	start	15.21 (0.66) [11]	15.54 (0.87) [9]	0.64 (0.37-0.9)	15.37	0.67	0.73
CFD	K7ERG9	end	16.26 (0.66) [11]	16.92 (0.65) [9]	0.77 (0.52-1)	17.00	0.67	0.91
CFD	K7ERG9	change	-0.08 (0.03) [10]	-0.06 (0.08) [7]	0.67 (0.36-0.98)	-0.05	0.57	0.90
CFH	P08603	start	16.69 (0.45) [12]	17.05 (1.24) [11]	0.59 (0.34-0.84)	17.04	0.55	0.75
CFH	P08603	end	16.01 (0.37) [12]	16.45 (1.08) [10]	0.67 (0.43-0.9)	15.86	0.90	0.42
CFH	P08603	change	0.06 (0.06) [12]	0.03 (0.11) [10]	0.61 (0.36-0.86)	0.05	0.80	0.50
CFHR1	B1AKG0	end	14.92 (0.73) [12]	15.09 (0.81) [9]	0.57 (0.3-0.85)	15.50	0.44	0.92
CFHR1	B1AKG0	change	0.06 (0.08) [12]	0.06 (0.04) [8]	0.62 (0.34-0.91)	0.04	0.88	0.67
CFHR1	B1AKG0	start	15.7 (0.97) [12]	16.44 (0.8) [10]	0.76 (0.54-0.97)	15.57	0.90	0.58
CFI	E7ETH0	change	0.04 (0.04) [12]	0.02 (0.01) [8]	0.51 (0.24-0.78)	0.05	1.00	0.33
CFI	E7ETH0	start	15.31 (0.44) [12]	15.47 (0.53) [10]	0.65 (0.4-0.9)	15.08	0.90	0.42

**Protein Name**	**Uniprot ID**	**Time**	**SAH Weaned** Mean (SD) [N]	**SAH Shunted** Mean (SD) [N]	**AUC (95%CI)**	**Cut-off**	**Sens.**	**Spec.**
CFI	E7ETH0	end	14.85 (0.37) [12]	15.02 (0.34) [9]	0.63 (0.37-0.89)	14.73	0.89	0.42
CFL1	E9PK25	start	15.3 (0.72) [12]	15.02 (1.32) [9]	0.58 (0.31-0.86)	14.82	0.44	0.83
CFL1	E9PK25	change	0 (0.11) [12]	0.02 (0.12) [7]	0.58 (0.29-0.87)	0.01	0.71	0.58
CFL1	E9PK25	end	15.11 (1.35) [12]	14.68 (0.71) [9]	0.68 (0.42-0.94)	14.75	0.78	0.75
CGREF1	Q99674	end	12.99 (0.56) [9]	13.03 (0.87) [9]	0.54 (0.25-0.84)	12.53	0.44	0.89
CHGA	P10645	start	14.58 (0.99) [10]	14.33 (1.01) [8]	0.66 (0.38-0.95)	14.31	0.75	0.70
CHGA	P10645	end	15.68 (0.97) [12]	15.55 (0.7) [9]	0.65 (0.38-0.91)	15.47	0.56	0.83
CHGA	P10645	change	-0.1 (0.12) [10]	-0.06 (0.04) [6]	0.65 (0.37-0.93)	-0.13	1.00	0.40
CHGB	P05060	change	-0.11 (0.09) [11]	-0.08 (0.07) [8]	0.59 (0.32-0.86)	-0.14	0.88	0.45
CHGB	P05060	start	14.14 (1.05) [11]	13.87 (1.44) [10]	0.59 (0.32-0.86)	14.35	0.80	0.55
CHGB	P05060	end	15.48 (0.95) [12]	15.43 (0.62) [9]	0.56 (0.29-0.82)	16.02	0.89	0.50
CHI3L1	P36222	change	-0.13 (0.16) [9]	-0.03 (0.1) [10]	0.72 (0.46-0.99)	-0.12	0.90	0.67
CHI3L1	P36222	start	15.31 (1.86) [10]	15.48 (2.11) [11]	0.5 (0.24-0.76)	16.54	0.27	0.90
CHI3L1	P36222	end	16.76 (0.64) [11]	16.51 (1.24) [10]	0.6 (0.34-0.86)	16.45	0.60	0.73
CHI3L2	Q15782	end	13.97 (1.51) [10]	13.35 (0.85) [9]	0.61 (0.34-0.88)	13.29	0.56	0.80
CHI3L2	Q15782	start	14.26 (1.42) [7]	14.24 (1.84) [7]	0.55 (0.22-0.88)	12.32	0.29	1.00

Protein Name	Uniprot ID	Time	**SAH Weaned** Mean (SD) [N]	**SAH Shunted** Mean (SD) [N]	**AUC (95%CI)**	**Cut-off**	**Sens.**	**Spec.**
CHI3L2	Q15782	change	-0.03 (0.2) [7]	0.07 (0.08) [5]	0.69 (0.35-1)	-0.03	1.00	0.57
CHL1	O00533	change	-0.08 (0.09) [11]	-0.03 (0.04) [6]	0.7 (0.44-0.96)	-0.08	1.00	0.45
CHL1	O00533	start	13.58 (1.06) [11]	13.56 (1.17) [8]	0.52 (0.24-0.81)	13.81	0.75	0.45
CHL1	O00533	end	14.68 (0.78) [12]	14.16 (0.7) [9]	0.72 (0.49-0.96)	14.34	0.78	0.75
CKB	P12277	end	14.27 (1.16) [9]	15.86 (2.21) [6]	0.76 (0.5-1)	13.72	1.00	0.44
CLCNKB	A0A087X136	end	15.91 (0.74) [11]	15.14 (0.85) [9]	0.78 (0.55-1)	15.03	0.67	0.91
CLEC11A	Q9Y240	end	12.94 (0.59) [7]	13.17 (0.59) [8]	0.55 (0.23-0.87)	13.66	0.25	1.00
CLEC3B	E9PHK0	change	-0.03 (0.05) [12]	-0.02 (0.04) [8]	0.56 (0.3-0.83)	-0.06	1.00	0.25
CLEC3B	E9PHK0	start	15.71 (0.41) [12]	15.86 (0.64) [10]	0.52 (0.26-0.78)	16.46	0.20	1.00
CLEC3B	E9PHK0	end	16.01 (0.56) [12]	16.26 (0.26) [9]	0.69 (0.43-0.96)	16.07	0.89	0.75
CLSTN1	O94985	change	-0.08 (0.06) [8]	-0.05 (0.06) [5]	0.65 (0.3-1)	-0.01	0.40	1.00
CLSTN1	O94985	start	14.84 (0.73) [9]	14.89 (1.15) [6]	0.54 (0.2-0.87)	14.37	0.50	0.78
CLSTN1	O94985	end	15.87 (0.78) [11]	15.41 (0.8) [10]	0.66 (0.41-0.92)	15.47	0.60	0.82
CLSTN3	Q9BQT9	end	11.06 (1.48) [8]	12.18 (2.26) [9]	0.64 (0.35-0.93)	12.56	0.56	0.88
CLU	P10909	end	16.81 (0.59) [12]	17.26 (1.26) [10]	0.56 (0.3-0.82)	16.56	0.90	0.42
CLU	P10909	start	16.28 (0.69) [12]	16.43 (0.53) [11]	0.61 (0.35-0.86)	15.77	1.00	0.42
CLU	P10909	change	-0.03 (0.07) [12]	-0.04 (0.08) [10]	0.53 (0.28-0.79)	-0.05	0.70	0.50

Protein Name	Uniprot ID	Time	SAH Weaned Mean (SD) [N]	SAH Shunted Mean (SD) [N]	AUC (95%CI)	Cut-off	Sens.	Spec.
CNDP1	Q96KN2	end	16.01 (0.68) [12]	15.94 (0.52) [10]	0.56 (0.3-0.81)	16.48	0.90	0.42
CNDP1	Q96KN2	change	-0.06 (0.06) [12]	-0.03 (0.06) [9]	0.59 (0.34-0.85)	-0.06	0.78	0.50
CNDP1	Q96KN2	start	15.18 (0.91) [12]	15.25 (1.15) [10]	0.55 (0.29-0.81)	15.29	0.70	0.50
CNTN1	Q12860	end	14.86 (0.83) [12]	14.41 (0.57) [9]	0.69 (0.46-0.93)	14.99	1.00	0.50
CNTN1	Q12860	change	-0.06 (0.05) [9]	-0.03 (0.07) [6]	0.56 (0.22-0.89)	-0.03	0.50	0.78
CNTN1	Q12860	start	14.13 (0.91) [9]	13.91 (1.21) [8]	0.64 (0.35-0.93)	13.48	0.62	0.78
CNTN2	A0A1W2PQ11	end	14.78 (0.9) [12]	14.43 (0.69) [9]	0.65 (0.4-0.9)	14.54	0.67	0.67
CNTN2	A0A1W2PQ11	start	14.19 (1.16) [9]	14.14 (1.09) [8]	0.56 (0.26-0.85)	14.26	0.75	0.56
CNTN2	A0A1W2PQ11	change	-0.05 (0.07) [9]	-0.01 (0.06) [6]	0.63 (0.33-0.93)	-0.05	1.00	0.44
COL18A1	P39060	end	14.7 (0.95) [12]	14.89 (0.53) [9]	0.5 (0.24-0.76)	14.24	1.00	0.25
COL18A1	P39060	start	14.49 (1.03) [7]	13.83 (1.34) [5]	0.63 (0.25-1)	14.73	0.80	0.57
COL1A1	P02452	end	15.64 (1.28) [12]	16.95 (1.65) [10]	0.78 (0.56-0.99)	16.51	0.80	0.83
COL1A2	A0A087WTA8	end	15.38 (1.34) [12]	15.84 (0.33) [9]	0.75 (0.52-0.98)	15.63	0.78	0.75
COL2A1	P02458	end	16.32 (1.22) [7]	15.64 (2.43) [9]	0.51 (0.19-0.83)	13.74	0.33	1.00
COL3A1	P02461	end	14.6 (1.03) [10]	15.61 (0.56) [8]	0.81 (0.6-1)	14.82	1.00	0.60
COL6A1	A0A087X0S5	end	14.63 (0.45) [11]	14.36 (0.28) [9]	0.74 (0.51-0.97)	14.77	1.00	0.45
COL6A1	A0A087X0S5	start	13.64 (1.17) [9]	13.59 (1.25) [7]	0.52 (0.21-0.84)	14.55	0.86	0.33

Protein Name	Uniprot ID	Time	SAH Weaned Mean (SD) [N]	SAH Shunted Mean (SD) [N]	AUC (95%CI)	Cut-off	Sens.	Spec.
COL6A1	A0A087X0S5	change	-0.08 (0.1) [9]	-0.04 (0.08) [5]	0.58 (0.24-0.91)	-0.12	1.00	0.33
COL6A2	P12110	end	13.44 (0.75) [5]	12.97 (0.86) [5]	0.68 (0.27-1)	13.33	0.80	0.80
COL6A3	P12111	start	13.97 (0.6) [7]	13.89 (0.88) [5]	0.51 (0.13-0.9)	14.06	0.80	0.43
COL6A3	P12111	end	14.02 (0.42) [11]	14.32 (0.24) [9]	0.72 (0.48-0.95)	13.90	1.00	0.45
COLEC12	Q5KU26	end	14.42 (0.51) [10]	14.37 (0.65) [8]	0.51 (0.21-0.82)	14.77	0.38	0.90
CORO1A	P31146	end	13.88 (1.62) [10]	12.27 (0.77) [7]	0.81 (0.59-1)	13.31	1.00	0.70
COTL1	Q14019	end	13.77 (1.2) [8]	13.11 (0.42) [9]	0.71 (0.41-1)	13.61	1.00	0.62
CP	P00450	end	16.46 (0.26) [12]	16.75 (0.79) [10]	0.62 (0.36-0.88)	16.70	0.50	0.83
CP	P00450	change	0.02 (0.05) [12]	0.01 (0.07) [10]	0.54 (0.28-0.8)	0.00	0.80	0.42
CP	P00450	start	16.69 (0.36) [12]	17.02 (0.81) [11]	0.67 (0.42-0.91)	16.95	0.64	0.83
CPB2	A0A087WSY5	change	0.02 (0.08) [12]	-0.02 (0.11) [9]	0.52 (0.24-0.79)	0.00	0.78	0.58
CPB2	A0A087WSY5	start	15.59 (0.75) [12]	15.82 (0.73) [10]	0.6 (0.34-0.86)	15.29	0.80	0.50
CPB2	A0A087WSY5	end	15.45 (0.61) [12]	15.84 (2.07) [10]	0.54 (0.27-0.81)	15.19	0.50	0.75
CPE	P16870	change	-0.05 (0.08) [11]	0.01 (0.03) [8]	0.72 (0.47-0.96)	-0.02	0.88	0.55
CPE	P16870	start	14.97 (1.41) [11]	15.13 (1.09) [10]	0.5 (0.24-0.76)	15.28	0.50	0.64
CPE	P16870	end	15.59 (1.18) [12]	14.91 (0.64) [9]	0.69 (0.46-0.93)	16.27	1.00	0.42
CPN1	P15169	start	15.14 (0.61) [11]	15.79 (0.62) [8]	0.77 (0.53-1)	15.64	0.75	0.82

**Protein Name**	**Uniprot ID**	**Time**	**SAH Weaned** Mean (SD) [N]	**SAH Shunted** Mean (SD) [N]	**AUC (95%CI)**	**Cut-off**	**Sens.**	**Spec.**
CPN1	P15169	end	14.18 (0.89) [7]	14.58 (1.05) [8]	0.62 (0.32-0.93)	14.80	0.50	0.86
CPN1	P15169	change	0.13 (0.18) [6]	0.05 (0.09) [6]	0.58 (0.23-0.94)	0.31	1.00	0.17
CPN2	P22792	end	15.31 (1.04) [12]	15.25 (0.5) [9]	0.54 (0.27-0.8)	14.57	1.00	0.33
CPN2	P22792	change	0.12 (0.14) [12]	0.07 (0.08) [8]	0.56 (0.29-0.83)	0.14	1.00	0.25
CPN2	P22792	start	16.64 (0.86) [12]	16.78 (1.33) [10]	0.62 (0.36-0.89)	17.08	0.70	0.75
CPQ	Q9Y646	start	14.07 (0.91) [8]	14.39 (1.26) [5]	0.5 (0.14-0.86)	13.15	1.00	0.25
CPQ	Q9Y646	end	14.5 (1.1) [12]	13.67 (0.85) [9]	0.74 (0.52-0.96)	13.99	0.78	0.67
CPVL	Q9H3G5	end	14.65 (0.87) [11]	13.62 (0.45) [9]	0.87 (0.69-1)	13.82	0.89	0.82
CPVL	Q9H3G5	start	14.22 (0.77) [8]	14.09 (0.75) [6]	0.5 (0.16-0.84)	13.86	0.83	0.38
CPVL	Q9H3G5	change	-0.02 (0.06) [8]	0.02 (0.06) [5]	0.75 (0.44-1)	0.01	0.80	0.75
CRP	P02741	change	-0.06 (0.09) [6]	-0.04 (0.07) [6]	0.56 (0.18-0.93)	-0.03	0.67	0.67
CRP	P02741	start	14.41 (1.2) [10]	13.96 (1.26) [8]	0.62 (0.35-0.9)	14.45	0.75	0.60
CRP	P02741	end	15.16 (1.39) [8]	15.12 (1.4) [8]	0.5 (0.19-0.81)	16.33	0.38	0.88
CRTAC1	A0A0C4DFP6	end	14.88 (0.44) [11]	14.51 (0.5) [9]	0.73 (0.49-0.96)	14.46	0.56	0.91
CRTAC1	A0A0C4DFP6	change	-0.03 (0.04) [10]	-0.01 (0.05) [6]	0.65 (0.3-1)	-0.01	0.67	0.90
CRTAC1	A0A0C4DFP6	start	14.24 (0.76) [10]	14.4 (1.28) [7]	0.54 (0.21-0.88)	14.92	0.43	0.90
CSF1	P09603	start	14.52 (1.05) [7]	13.78 (0.96) [7]	0.73 (0.46-1)	14.43	0.71	0.71

**Protein Name**	**Uniprot ID**	**Time**	**SAH Weaned** Mean (SD) [N]	**SAH Shunted** Mean (SD) [N]	**AUC (95%CI)**	**Cut-off**	**Sens.**	**Spec.**
CSF1	P09603	end	14.55 (0.95) [11]	13.73 (0.89) [8]	0.74 (0.5-0.98)	14.54	1.00	0.64
CSF1R	E9PEK4	end	15.91 (0.51) [11]	15.37 (0.53) [9]	0.77 (0.55-0.98)	15.56	0.67	0.82
CST3	P01034	start	15.84 (1.73) [12]	16.04 (1.48) [11]	0.55 (0.29-0.8)	16.89	0.82	0.42
CST3	P01034	change	-0.1 (0.12) [12]	-0.07 (0.09) [10]	0.52 (0.27-0.78)	-0.15	0.90	0.33
CST3	P01034	end	17.39 (0.79) [12]	17.63 (1.06) [10]	0.6 (0.35-0.85)	17.80	0.90	0.42
CSTB	P04080	end	15.94 (1.24) [12]	15.61 (0.52) [9]	0.63 (0.37-0.89)	16.33	1.00	0.50
CTBS	Q01459	end	14.46 (0.69) [10]	13.79 (0.34) [9]	0.82 (0.62-1)	14.08	0.89	0.70
CTSB	P07858	end	15.13 (1.73) [11]	14.66 (0.79) [8]	0.57 (0.29-0.84)	15.71	1.00	0.36
CTSB	P07858	start	14.29 (1.21) [7]	14.99 (1.37) [6]	0.67 (0.35-0.99)	15.83	0.33	1.00
CTSC	P53634	end	14.07 (0.97) [9]	13.59 (0.56) [6]	0.59 (0.28-0.9)	14.12	0.83	0.44
CTSD	A0A1B0GV23	end	16.58 (1.23) [12]	15.69 (0.53) [9]	0.71 (0.47-0.95)	15.98	0.78	0.75
CTSD	A0A1B0GV23	start	15.75 (1.21) [11]	15.22 (1.06) [10]	0.66 (0.41-0.92)	15.19	0.60	0.82
CTSD	A0A1B0GV23	change	-0.07 (0.11) [11]	-0.03 (0.07) [8]	0.6 (0.33-0.87)	-0.06	0.75	0.55
CTSH	A0A087X0D5	end	14.64 (1.02) [10]	13.45 (0.67) [7]	0.83 (0.62-1)	14.56	1.00	0.60
CTSH	A0A087X0D5	start	13.86 (0.77) [6]	13.96 (1.18) [5]	0.5 (0.09-0.91)	13.78	0.60	0.67
CTSS	P25774	end	14.7 (0.98) [10]	13.82 (0.4) [8]	0.81 (0.59-1)	14.09	0.88	0.80

Protein Name	Uniprot ID	Time	**SAH Weaned** Mean (SD) [N]	**SAH Shunted** Mean (SD) [N]	**AUC (95%CI)**	**Cut-off**	**Sens.**	**Spec.**
CYCS	C9JFR7	end	13.79 (1.2) [9]	12.92 (0.5) [8]	0.76 (0.52-1)	13.46	1.00	0.56
DAG1	Q14118	end	15.28 (0.46) [11]	15.1 (0.28) [9]	0.67 (0.41-0.93)	15.27	0.78	0.64
DAG1	Q14118	change	-0.06 (0.06) [7]	-0.04 (0.07) [5]	0.54 (0.15-0.93)	-0.11	1.00	0.29
DAG1	Q14118	start	14.28 (1.03) [7]	14.27 (1.13) [7]	0.51 (0.18-0.84)	12.85	1.00	0.14
DBH	P09172	start	13.54 (1.19) [9]	14.26 (1.3) [6]	0.69 (0.36-1)	14.38	0.67	0.89
DBI	A0A0A0MTI5	end	15.19 (0.72) [12]	14.97 (0.33) [8]	0.62 (0.37-0.88)	15.37	1.00	0.42
DBI	A0A0A0MTI5	start	14.71 (0.59) [10]	14.53 (0.22) [7]	0.69 (0.41-0.96)	14.74	0.86	0.60
DBI	A0A0A0MTI5	change	-0.03 (0.07) [10]	-0.02 (0.02) [5]	0.52 (0.21-0.83)	-0.07	1.00	0.30
DCN	P07585	end	13.43 (1.18) [8]	13.02 (1.17) [9]	0.61 (0.33-0.9)	13.00	0.56	0.75
DKK3	F6SYF8	end	15.76 (1.07) [12]	15.48 (0.34) [9]	0.72 (0.49-0.95)	16.02	1.00	0.50
DKK3	F6SYF8	change	-0.09 (0.14) [10]	-0.08 (0.07) [8]	0.51 (0.22-0.81)	-0.07	0.75	0.60
DKK3	F6SYF8	start	14.83 (1.2) [10]	14.01 (1.52) [10]	0.67 (0.42-0.92)	14.36	0.70	0.70
DPP7	Q9UHL4	end	13.46 (0.98) [9]	12.44 (1.09) [6]	0.78 (0.52-1)	12.91	0.83	0.67
DSC2	Q02487	end	13.89 (0.78) [10]	13.45 (0.58) [9]	0.64 (0.37-0.92)	13.93	1.00	0.50
ECM1	Q16610	change	-0.01 (0.04) [10]	-0.01 (0.03) [8]	0.52 (0.24-0.81)	-0.04	0.88	0.40
ECM1	Q16610	start	14.42 (0.31) [10]	14.37 (0.52) [10]	0.53 (0.26-0.8)	14.57	0.40	0.80
ECM1	Q16610	end	14.62 (0.48) [12]	14.39 (0.25) [9]	0.66 (0.41-0.9)	14.83	1.00	0.42

Protein Name	Uniprot ID	Time	SAH Weaned<br><br>Mean (SD) [N]	SAH Shunted<br><br>Mean (SD) [N]	AUC (95%CI)	Cut-off	Sens.	Spec.
ECM2	O94769	end	14.06 (0.7) [9]	13.92 (0.55) [9]	0.51 (0.21-0.81)	14.20	0.56	0.78
EEF1A1	P68104	end	14.86 (1.06) [8]	15.09 (4.01) [9]	0.65 (0.37-0.94)	13.59	0.44	1.00
EFCAB14	O75071	end	13.99 (0.43) [8]	13.45 (0.78) [9]	0.67 (0.39-0.94)	13.58	0.44	0.88
EFEMP1	A0A0U1RQV3	start	15.44 (1.08) [10]	15.75 (1.7) [7]	0.53 (0.21-0.84)	16.84	0.29	1.00
EFEMP1	A0A0U1RQV3	change	-0.07 (0.09) [9]	0.01 (0.09) [6]	0.76 (0.49-1)	-0.07	1.00	0.67
EFEMP1	A0A0U1RQV3	end	16.28 (0.79) [11]	16.09 (0.44) [10]	0.62 (0.35-0.88)	16.46	0.90	0.55
EIF5A	I3L397	change	-0.03 (0.09) [9]	-0.07 (0.11) [5]	0.58 (0.24-0.91)	0.01	1.00	0.33
EIF5A	I3L397	start	14.28 (0.88) [10]	13.32 (1.3) [7]	0.74 (0.46-1)	13.47	0.71	0.80
EIF5A	I3L397	end	14.98 (1.03) [11]	13.98 (0.74) [9]	0.8 (0.58-1)	14.61	0.89	0.73
ENDOD1	O94919	end	15.09 (0.44) [11]	14.61 (0.31) [9]	0.82 (0.62-1)	14.67	0.67	0.91
ENDOD1	O94919	start	14.42 (0.5) [8]	14.6 (0.84) [6]	0.54 (0.2-0.89)	14.92	0.33	0.88
ENO1	P06733	end	16.21 (1.05) [12]	15.24 (0.78) [9]	0.78 (0.56-0.99)	15.70	0.78	0.83
ENO1	P06733	change	0 (0.08) [12]	0.07 (0.2) [5]	0.55 (0.19-0.91)	0.06	0.40	0.83
ENO1	P06733	start	16.35 (0.71) [12]	16.85 (3.14) [6]	0.62 (0.26-0.99)	15.30	0.50	1.00
ENO2	P09104	change	0.04 (0.11) [10]	0.11 (0.26) [5]	0.52 (0.18-0.86)	-0.09	1.00	0.20
ENO2	P09104	end	14.54 (0.85) [11]	13.87 (0.99) [8]	0.67 (0.41-0.93)	14.63	0.88	0.45

Protein Name	Uniprot ID	Time	SAH Weaned<br>Mean (SD) [N]	SAH Shunted<br>Mean (SD) [N]	AUC (95%CI)	Cut-off	Sens.	Spec.
ENO2	P09104	start	15.09 (0.8) [11]	15.4 (4.03) [7]	0.7 (0.41-0.99)	13.85	0.43	1.00
ENOPH1	A0A0C4DGY8	end	13.48 (0.97) [5]	12.62 (0.31) [7]	0.89 (0.69-1)	13.02	0.86	0.80
ENPP2	E7EUF1	change	-0.08 (0.11) [12]	-0.02 (0.06) [8]	0.66 (0.41-0.91)	-0.11	1.00	0.42
ENPP2	E7EUF1	end	16.74 (1.16) [12]	16.04 (0.41) [9]	0.71 (0.46-0.97)	16.56	0.89	0.67
ENPP2	E7EUF1	start	15.74 (1.39) [12]	15.59 (0.92) [10]	0.61 (0.36-0.86)	15.77	0.60	0.75
EPHA4	E9PG71	end	14.85 (0.78) [11]	14.29 (0.56) [9]	0.74 (0.51-0.97)	14.08	0.56	0.91
ERN1	O75460	end	15.19 (1.88) [7]	14.74 (1.61) [6]	0.55 (0.2-0.9)	17.00	1.00	0.29
ERN1	O75460	change	0.13 (0.18) [7]	0.16 (0.12) [5]	0.54 (0.18-0.91)	0.21	0.80	0.57
ERN1	O75460	start	17.24 (2.23) [12]	18.75 (1.43) [9]	0.71 (0.47-0.95)	19.05	0.56	0.92
ESD	H7BZT7	end	12.72 (1.18) [8]	12.67 (1.02) [7]	0.52 (0.19-0.84)	13.29	0.86	0.38
EXTL2	Q9UBQ6	end	14.33 (0.57) [9]	13.68 (0.39) [9]	0.83 (0.61-1)	13.95	0.78	0.89
F10	P00742	change	0.07 (0.04) [7]	0.07 (0.03) [6]	0.55 (0.19-0.91)	0.07	0.67	0.71
F10	P00742	end	14.07 (0.55) [9]	14.21 (0.45) [8]	0.51 (0.21-0.82)	14.14	0.62	0.67
F10	P00742	start	15.13 (0.66) [9]	15.82 (0.46) [8]	0.81 (0.57-1)	15.72	0.75	0.89
F12	P00748	change	0.07 (0.09) [12]	0.04 (0.03) [8]	0.55 (0.29-0.82)	0.07	0.88	0.42
F12	P00748	end	16.09 (0.69) [12]	16.38 (0.59) [9]	0.62 (0.36-0.88)	16.00	0.78	0.50
F12	P00748	start	16.89 (0.82) [12]	17.35 (0.87) [10]	0.67 (0.4-0.93)	17.37	0.70	0.83
F13A1	P00488	end	13.5 (2) [7]	12.42 (0.72) [8]	0.68 (0.34-1)	13.45	1.00	0.57

**Protein Name**	**Uniprot ID**	**Time**	**SAH Weaned** Mean (SD) [N]	**SAH Shunted** Mean (SD) [N]	**AUC (95%CI)**	**Cut-off**	**Sens.**	**Spec.**
F13B	P05160	start	13.32 (2.44) [9]	14.71 (0.36) [8]	0.85 (0.66-1)	14.00	1.00	0.56
F13B	P05160	end	12.87 (0.86) [5]	10.04 (3.46) [5]	0.76 (0.41-1)	12.12	0.80	0.80
F2	P00734	start	16.36 (0.61) [12]	16.94 (0.67) [10]	0.75 (0.53-0.97)	16.70	0.70	0.75
F2	P00734	change	0.02 (0.05) [12]	0.03 (0.01) [8]	0.54 (0.27-0.81)	0.02	1.00	0.42
F2	P00734	end	16.11 (0.42) [12]	16.12 (0.48) [9]	0.51 (0.24-0.78)	15.84	0.44	0.75
F5	A0A0A0MRJ7	change	-0.02 (0.06) [12]	0.04 (0.03) [8]	0.78 (0.57-1)	0.00	0.88	0.75
F5	A0A0A0MRJ7	start	14.92 (0.51) [12]	14.88 (0.36) [10]	0.54 (0.29-0.8)	15.33	1.00	0.25
F5	A0A0A0MRJ7	end	15.14 (0.9) [12]	14.06 (0.63) [9]	0.84 (0.67-1)	15.16	1.00	0.58
F9	P00740	end	13.26 (0.56) [11]	13.18 (0.42) [9]	0.59 (0.32-0.85)	13.60	1.00	0.36
F9	P00740	start	14 (0.5) [11]	14.35 (0.65) [10]	0.71 (0.47-0.95)	13.95	0.90	0.55
F9	P00740	change	0.05 (0.04) [10]	0.05 (0.03) [8]	0.58 (0.28-0.87)	0.03	0.88	0.50
FABP3	P05413	end	13.76 (0.82) [5]	13.57 (1.23) [5]	0.64 (0.23-1)	13.84	0.80	0.60
FAM3C	Q92520	start	13.97 (0.77) [9]	13.91 (1.06) [7]	0.56 (0.24-0.87)	13.48	0.57	0.67
FAM3C	Q92520	change	-0.08 (0.05) [9]	-0.03 (0.05) [5]	0.67 (0.33-1)	-0.07	1.00	0.44
FAM3C	Q92520	end	14.93 (0.72) [12]	14.49 (0.39) [9]	0.74 (0.52-0.97)	14.97	0.89	0.58
FAM49B	Q9NUQ9	end	13.54 (0.96) [9]	12.81 (0.5) [9]	0.74 (0.47-1)	13.07	0.78	0.78
FBLN1	P23142	start	15.61 (1.06) [12]	15.1 (0.89) [10]	0.71 (0.48-0.94)	14.91	0.50	0.92
FBLN1	P23142	change	-0.06 (0.08) [12]	-0.06 (0.07) [9]	0.53 (0.26-0.79)	-0.11	0.89	0.42

Protein Name	Uniprot ID	Time	SAH Weaned<br><br>Mean (SD) [N]	SAH Shunted<br><br>Mean (SD) [N]	AUC (95%CI)	Cut-off	Sens.	Spec.
FBLN1	P23142	end	16.36 (0.84) [12]	16.38 (1.12) [10]	0.65 (0.4-0.9)	16.57	0.90	0.58
FBLN5	G3V4U0	end	14.07 (0.82) [9]	14.65 (0.79) [9]	0.69 (0.44-0.95)	14.06	0.78	0.56
FBN1	P35555	end	13.97 (1.3) [5]	14.81 (1.98) [7]	0.63 (0.28-0.98)	14.08	0.71	0.60
FCGBP	Q9Y6R7	start	15.2 (1.68) [7]	14.27 (1.54) [5]	0.69 (0.36-1)	15.25	0.80	0.57
FCGBP	Q9Y6R7	end	16.3 (1.14) [10]	15.44 (1.22) [9]	0.71 (0.47-0.95)	16.44	0.89	0.50
FCGR3A	A0A1W2PQB1	start	13.83 (1.25) [10]	14.63 (0.92) [7]	0.69 (0.42-0.96)	13.85	0.86	0.60
FCGR3A	A0A1W2PQB1	change	-0.11 (0.08) [10]	-0.03 (0.05) [5]	0.8 (0.56-1)	-0.12	1.00	0.70
FCGR3A	A0A1W2PQB1	end	15.46 (1.15) [12]	15.33 (0.64) [9]	0.61 (0.35-0.87)	15.62	0.67	0.67
FCN3	O75636	start	14.98 (1.82) [10]	16.03 (0.95) [8]	0.72 (0.47-0.98)	16.11	0.62	0.80
FETUB	Q9UGM5	end	14.52 (0.86) [10]	14.2 (0.57) [9]	0.57 (0.28-0.85)	14.07	0.44	0.80
FETUB	Q9UGM5	start	15.49 (1.42) [11]	15.59 (0.8) [9]	0.59 (0.31-0.86)	16.34	1.00	0.45
FETUB	Q9UGM5	change	0.11 (0.2) [9]	0.05 (0.03) [7]	0.56 (0.21-0.9)	0.09	1.00	0.56
FGA	P02671	end	15.76 (1) [12]	16.39 (0.56) [10]	0.72 (0.48-0.95)	15.39	1.00	0.50
FGA	P02671	start	17.09 (1.08) [12]	16.31 (1.06) [11]	0.7 (0.47-0.92)	17.60	1.00	0.42
FGA	P02671	change	0.09 (0.11) [12]	-0.01 (0.05) [10]	0.75 (0.52-0.98)	0.06	1.00	0.67
FGB	P02675	start	18.43 (1.25) [12]	17.68 (1.65) [11]	0.64 (0.4-0.88)	18.52	0.73	0.58

**Protein Name**	**Uniprot ID**	**Time**	**SAH Weaned**<br><br>Mean (SD) [N]	**SAH Shunted**<br><br>Mean (SD) [N]	**AUC (95%CI)**	**Cut-off**	**Sens.**	**Spec.**
FGB	P02675	change	0.07 (0.13) [12]	-0.03 (0.08) [10]	0.72 (0.49-0.94)	0.07	0.90	0.58
FGB	P02675	end	17.26 (1.24) [12]	18.23 (1) [10]	0.74 (0.51-0.97)	17.69	0.80	0.75
FGFR2	A0A0A0MR25	end	14.85 (0.54) [8]	14.27 (0.5) [5]	0.8 (0.54-1)	15.00	1.00	0.62
FGG	P02679	change	0.07 (0.13) [12]	-0.02 (0.09) [10]	0.72 (0.48-0.95)	0.06	0.90	0.58
FGG	P02679	end	16.46 (1.13) [12]	17.31 (1.03) [10]	0.72 (0.49-0.95)	16.11	1.00	0.50
FGG	P02679	start	17.62 (1.17) [12]	16.89 (1.64) [11]	0.64 (0.41-0.88)	17.17	0.64	0.67
FKBP1A	P62942	end	15.62 (1.17) [10]	15.04 (0.49) [8]	0.74 (0.49-0.98)	16.14	1.00	0.50
FLNA	P21333	end	13.15 (0.66) [8]	13.37 (0.94) [9]	0.61 (0.32-0.9)	13.12	0.78	0.50
FLNA	P21333	start	13.64 (0.68) [9]	14.29 (1.41) [6]	0.61 (0.28-0.94)	13.13	1.00	0.33
FMOD	Q06828	end	13.51 (1.13) [8]	13.39 (0.37) [9]	0.56 (0.23-0.88)	13.47	0.78	0.62
FN1	P02751	change	0.02 (0.04) [12]	-0.01 (0.07) [10]	0.68 (0.44-0.92)	0.03	0.90	0.50
FN1	P02751	end	16.32 (0.34) [12]	16.95 (0.82) [10]	0.8 (0.59-1)	16.69	0.70	0.92
FN1	P02751	start	16.53 (0.48) [12]	16.77 (0.84) [11]	0.56 (0.31-0.81)	16.04	1.00	0.25
FRRS1L	Q9P0K9	end	10.22 (0.82) [6]	10.79 (2.47) [5]	0.5 (0.05-0.95)	12.37	0.40	1.00
FRZB	Q92765	end	13.83 (1.15) [8]	12.87 (0.82) [8]	0.75 (0.49-1)	14.04	1.00	0.50
FSTL1	Q12841	change	-0.08 (0.19) [10]	-0.06 (0.13) [5]	0.56 (0.25-0.87)	-0.20	1.00	0.30
FSTL1	Q12841	end	14.08 (1.44) [12]	14.09 (1.11) [9]	0.53 (0.26-0.8)	15.07	1.00	0.33

Protein Name	Uniprot ID	Time	SAH Weaned<br><br>Mean (SD) [N]	SAH Shunted<br><br>Mean (SD) [N]	AUC (95%CI)	Cut-off	Sens.	Spec.
FSTL1	Q12841	start	13.49 (1.38) [10]	12.85 (1.49) [7]	0.67 (0.39-0.96)	13.61	0.86	0.50
FTH1	P02794	end	13.92 (2.09) [10]	12.22 (1.64) [9]	0.74 (0.52-0.97)	14.00	0.89	0.50
FTL	P02792	end	16.23 (2.45) [12]	16.29 (1.53) [9]	0.51 (0.24-0.78)	16.04	0.56	0.67
FUCA1	P04066	end	14.34 (1.02) [12]	13.34 (0.49) [8]	0.82 (0.62-1)	14.23	1.00	0.67
FUCA2	Q9BTY2	start	13.72 (1.27) [8]	13.88 (1.04) [7]	0.52 (0.19-0.84)	12.67	1.00	0.25
FUCA2	Q9BTY2	change	-0.06 (0.1) [8]	0.04 (0.07) [5]	0.78 (0.5-1)	-0.04	1.00	0.50
FXYD6	Q9H0Q3	end	15.03 (0.54) [8]	14.55 (0.7) [8]	0.7 (0.41-1)	14.54	0.62	0.88
GANAB	Q14697	end	12.85 (0.49) [8]	12.32 (0.72) [9]	0.71 (0.44-0.98)	12.25	0.44	1.00
GAP43	P17677	end	12.23 (1.63) [5]	11.89 (1.13) [8]	0.62 (0.24-1)	13.50	1.00	0.40
GAPDH	P04406	end	15.38 (1.14) [12]	15.45 (1.04) [9]	0.51 (0.24-0.78)	15.67	0.78	0.50
GAPDH	P04406	start	15.17 (1.08) [12]	15.04 (1.41) [9]	0.56 (0.29-0.84)	14.65	0.56	0.75
GAPDH	P04406	change	-0.01 (0.11) [12]	-0.02 (0.1) [7]	0.54 (0.25-0.82)	0.00	0.71	0.58
GC	P02774	end	16.78 (0.43) [12]	17.23 (0.86) [10]	0.68 (0.45-0.92)	16.83	0.80	0.67
GC	P02774	change	0.03 (0.06) [12]	0.01 (0.07) [10]	0.55 (0.29-0.81)	0.02	0.90	0.42
GC	P02774	start	17.06 (0.49) [12]	17.4 (0.79) [11]	0.63 (0.39-0.87)	17.43	0.45	0.83
GDI2	P50395	change	0 (0.1) [9]	-0.01 (0.08) [5]	0.62 (0.28-0.97)	-0.03	0.60	0.78
GDI2	P50395	end	14.48 (1.18) [12]	14.02 (0.53) [9]	0.7 (0.46-0.94)	13.97	0.67	0.83

Protein Name	Uniprot ID	Time	**SAH Weaned** Mean (SD) [N]	**SAH Shunted** Mean (SD) [N]	**AUC (95%CI)**	**Cut-off**	**Sens.**	**Spec.**
GDI2	P50395	start	14.34 (0.7) [9]	14.27 (1.47) [6]	0.61 (0.24-0.98)	13.39	0.50	1.00
GFAP	A0A1W2PR46	start	17.97 (2.29) [11]	16.8 (2.51) [6]	0.65 (0.34-0.96)	14.43	0.33	1.00
GGH	Q92820	start	14.36 (0.51) [9]	14.27 (0.37) [8]	0.56 (0.26-0.85)	14.49	0.75	0.56
GGH	Q92820	end	14.13 (0.49) [11]	13.37 (0.93) [5]	0.75 (0.44-1)	13.53	0.60	0.91
GM2A	P17900	start	14.95 (1.07) [10]	14.34 (1.37) [9]	0.64 (0.38-0.91)	13.26	0.33	1.00
GM2A	P17900	end	15.8 (0.85) [12]	15.53 (0.4) [9]	0.74 (0.52-0.96)	16.05	1.00	0.50
GM2A	P17900	change	-0.07 (0.11) [10]	-0.06 (0.07) [7]	0.53 (0.23-0.82)	-0.12	0.86	0.40
GNPTG	Q9UJJ9	end	15.57 (0.58) [11]	15.16 (0.36) [8]	0.75 (0.5-1)	15.67	1.00	0.64
GOLM1	Q8NBJ4	end	13.51 (0.44) [10]	13.15 (0.49) [7]	0.79 (0.52-1)	12.98	0.71	0.90
GOT1	P17174	end	15.21 (0.73) [12]	14.86 (0.67) [9]	0.67 (0.42-0.92)	15.44	0.89	0.50
GPI	A0A0A0MTS2	end	13.98 (0.87) [10]	13.29 (0.6) [9]	0.72 (0.48-0.97)	13.34	0.67	0.80
GPLD1	P80108	end	14.82 (1.09) [8]	14 (0.71) [7]	0.7 (0.41-0.98)	15.09	1.00	0.38
GPLD1	P80108	change	0.11 (0.14) [7]	0.07 (0.09) [6]	0.62 (0.27-0.97)	0.13	1.00	0.43
GPLD1	P80108	start	15.69 (1.05) [11]	15.92 (1.05) [9]	0.62 (0.34-0.89)	16.56	0.33	1.00
GPR37	O15354	end	15.22 (0.45) [10]	14.91 (0.36) [9]	0.69 (0.44-0.94)	14.97	0.67	0.70
GPR37L1	O60883	start	13.89 (1.27) [10]	13.04 (0.99) [7]	0.69 (0.41-0.96)	12.95	0.71	0.70

Protein Name	Uniprot ID	Time	**SAH Weaned** Mean (SD) [N]	**SAH Shunted** Mean (SD) [N]	**AUC (95%CI)**	**Cut-off**	**Sens.**	**Spec.**
GPR37L1	O60883	end	14.46 (1.31) [12]	13.92 (0.6) [9]	0.66 (0.42-0.9)	15.20	1.00	0.33
GPR37L1	O60883	change	-0.07 (0.17) [10]	-0.05 (0.09) [5]	0.58 (0.27-0.89)	-0.17	1.00	0.40
GPX3	A0A087X1J7	end	16.14 (0.72) [12]	15.66 (0.24) [9]	0.7 (0.46-0.94)	16.07	1.00	0.58
GPX3	A0A087X1J7	start	15.98 (0.61) [12]	16.03 (0.6) [10]	0.53 (0.27-0.79)	16.29	0.50	0.75
GPX3	A0A087X1J7	change	-0.02 (0.05) [12]	0.01 (0.02) [8]	0.64 (0.38-0.89)	-0.03	1.00	0.42
GSN	P06396	change	-0.02 (0.04) [12]	-0.02 (0.05) [10]	0.5 (0.24-0.76)	-0.04	0.90	0.33
GSN	P06396	end	16.35 (0.41) [12]	16.51 (0.67) [10]	0.55 (0.3-0.8)	16.31	0.70	0.50
GSN	P06396	start	16.08 (0.44) [12]	16.14 (0.47) [11]	0.52 (0.26-0.77)	15.95	0.82	0.50
GSR	P00390	end	13.95 (0.77) [6]	13.78 (0.39) [5]	0.57 (0.19-0.95)	14.49	1.00	0.33
GSS	P48637	end	15.45 (1.68) [8]	14.04 (1.47) [7]	0.71 (0.41-1)	13.54	0.57	1.00
GSTO1	P78417	end	15.35 (1.04) [12]	15.22 (0.46) [9]	0.56 (0.31-0.82)	16.02	1.00	0.25
GSTP1	P09211	start	15.69 (1.12) [10]	14.7 (0.82) [8]	0.79 (0.55-1)	14.88	0.75	0.90
GSTP1	P09211	change	-0.01 (0.11) [10]	-0.05 (0.07) [6]	0.67 (0.38-0.95)	-0.01	0.83	0.60
GSTP1	P09211	end	15.79 (0.89) [12]	15.42 (0.86) [9]	0.66 (0.4-0.91)	15.49	0.78	0.67
HABP2	Q14520	start	13.77 (0.48) [5]	14.38 (0.51) [7]	0.8 (0.51-1)	14.17	0.86	0.80
HBA1	P69905	start	20.16 (1.71) [12]	19.79 (3.41) [11]	0.59 (0.33-0.86)	18.93	0.55	0.83
HBA1	P69905	end	20.44 (2.82) [12]	21.51 (1.74) [10]	0.62 (0.36-0.87)	19.21	1.00	0.42

Protein Name	Uniprot ID	Time	SAH Weaned<br><br>Mean (SD) [N]	SAH Shunted<br><br>Mean (SD) [N]	AUC (95%CI)	Cut-off	Sens.	Spec.
HBA1	P69905	change	-0.04 (0.22) [12]	-0.08 (0.19) [10]	0.54 (0.27-0.81)	0.00	0.90	0.50
HBB	P68871	end	20.64 (2.98) [12]	21.78 (2.49) [10]	0.55 (0.29-0.81)	19.34	1.00	0.25
HBB	P68871	start	20.31 (1.87) [12]	19.86 (4.01) [11]	0.6 (0.33-0.86)	19.98	0.64	0.75
HBB	P68871	change	-0.03 (0.2) [12]	-0.09 (0.23) [10]	0.56 (0.3-0.82)	0.01	0.90	0.42
HBD	P02042	change	-0.07 (0.18) [11]	0 (0.18) [8]	0.59 (0.31-0.87)	-0.09	0.88	0.55
HBD	P02042	start	17.54 (1.51) [12]	18.29 (3.21) [9]	0.52 (0.22-0.81)	17.50	0.67	0.58
HBD	P02042	end	18.45 (1.53) [11]	18.63 (2.36) [10]	0.52 (0.25-0.79)	19.64	0.40	0.82
HBG2	P69892	start	15.88 (0.97) [8]	16.59 (3.29) [9]	0.51 (0.21-0.82)	13.98	0.22	1.00
HBG2	P69892	end	17 (1.18) [8]	15.63 (3.04) [10]	0.68 (0.4-0.95)	15.12	0.50	1.00
HBG2	P69892	change	-0.06 (0.1) [6]	0.05 (0.23) [8]	0.56 (0.24-0.89)	-0.04	0.62	0.67
HEXA	H3BP20	end	14.41 (0.7) [11]	13.8 (0.65) [7]	0.75 (0.51-1)	14.17	0.86	0.73
HEXB	P07686	start	13.43 (0.84) [6]	13.5 (0.91) [6]	0.53 (0.15-0.9)	13.50	0.67	0.67
HGFAC	D6RAR4	end	14.05 (0.64) [9]	14.07 (0.29) [7]	0.54 (0.23-0.85)	14.13	0.71	0.56
HGFAC	D6RAR4	change	0.1 (0.13) [7]	0.05 (0.02) [6]	0.55 (0.18-0.91)	0.06	0.83	0.57
HGFAC	D6RAR4	start	15.07 (0.54) [10]	15.19 (0.62) [10]	0.53 (0.26-0.8)	15.57	0.30	1.00
HIST1H2BK	O60814	end	15.89 (1.42) [10]	14.55 (1) [7]	0.77 (0.54-1)	15.29	0.86	0.70

**Protein Name**	**Uniprot ID**	**Time**	**SAH Weaned**<br><br>Mean (SD) [N]	**SAH Shunted**<br><br>Mean (SD) [N]	**AUC (95%CI)**	**Cut-off**	**Sens.**	**Spec.**
HIST1H4A	P62805	end	16.91 (2.03) [12]	15.58 (2.29) [10]	0.69 (0.45-0.93)	16.95	0.80	0.58
HIST1H4A	P62805	start	15.32 (1.76) [8]	16.24 (3.84) [5]	0.55 (0.16-0.94)	17.51	0.40	0.88
HIST1H4A	P62805	change	-0.11 (0.16) [8]	-0.02 (0.31) [5]	0.55 (0.17-0.93)	-0.14	0.60	0.62
HLA-C	A0A140T921	end	14.32 (1.12) [10]	13.54 (1.05) [6]	0.68 (0.4-0.96)	13.57	0.67	0.70
HP	P00738	end	15.71 (2.09) [12]	16.26 (2.54) [10]	0.55 (0.29-0.81)	17.04	0.40	0.83
HP	P00738	change	0.25 (0.37) [12]	0.15 (0.23) [10]	0.52 (0.27-0.78)	0.24	0.60	0.58
HP	P00738	start	18.34 (1.99) [12]	19.59 (2.04) [11]	0.69 (0.46-0.92)	19.53	0.82	0.67
HPR	P00739	change	0.17 (0.26) [12]	0.03 (0.23) [8]	0.56 (0.29-0.83)	0.30	1.00	0.17
HPR	P00739	end	14.69 (1.69) [12]	15.82 (2.25) [9]	0.7 (0.46-0.95)	14.30	1.00	0.58
HPR	P00739	start	16.48 (1.35) [12]	17.18 (1.98) [10]	0.66 (0.4-0.92)	17.94	0.50	0.92
HPX	P02790	change	0.06 (0.09) [12]	0.04 (0.05) [10]	0.55 (0.29-0.81)	0.01	0.90	0.42
HPX	P02790	end	17.74 (0.71) [12]	17.66 (0.65) [10]	0.54 (0.29-0.8)	18.62	1.00	0.17
HPX	P02790	start	18.34 (0.46) [12]	18.54 (0.59) [11]	0.69 (0.44-0.94)	18.75	0.64	0.92
HRG	P04196	end	15.95 (0.58) [12]	16.02 (0.38) [10]	0.6 (0.35-0.85)	16.03	0.60	0.75
HRG	P04196	change	0.07 (0.07) [12]	0.04 (0.04) [10]	0.64 (0.39-0.89)	0.09	1.00	0.42
HRG	P04196	start	16.82 (0.72) [12]	16.91 (0.75) [11]	0.53 (0.28-0.78)	15.95	0.91	0.25

**Protein Name**	**Uniprot ID**	**Time**	**SAH Weaned** Mean (SD) [N]	**SAH Shunted** Mean (SD) [N]	**AUC (95%CI)**	**Cut-off**	**Sens.**	**Spec.**
HSP90B1	P14625	end	14.55 (0.89) [10]	14.17 (0.34) [9]	0.62 (0.34-0.9)	14.70	1.00	0.40
HSP90AA1	P07900	end	14.83 (1.14) [10]	14.27 (0.61) [7]	0.66 (0.38-0.94)	15.08	1.00	0.40
HSP90AA1	P07900	change	-0.01 (0.12) [9]	0.06 (0.03) [5]	0.62 (0.31-0.93)	0.00	1.00	0.44
HSP90AA1	P07900	start	14.86 (1.01) [10]	15.19 (1.05) [8]	0.58 (0.29-0.86)	15.84	0.38	0.90
HSPA1B	A0A0G2JIW1	end	15.63 (1.07) [10]	14.94 (0.85) [7]	0.7 (0.43-0.97)	15.38	0.86	0.60
HSPA1B	A0A0G2JIW1	start	15.02 (0.88) [10]	13.74 (1.46) [5]	0.78 (0.43-1)	14.09	0.80	0.80
HSPA5	P11021	start	14.04 (0.64) [6]	13.85 (1.05) [6]	0.67 (0.31-1)	13.61	0.67	0.83
HSPA5	P11021	end	15.02 (0.93) [11]	14.61 (0.13) [9]	0.64 (0.35-0.92)	14.89	1.00	0.55
HSPA8	P11142	end	15.33 (1.46) [12]	14.6 (0.86) [9]	0.73 (0.49-0.97)	15.06	0.89	0.75
HSPA8	P11142	start	15.39 (0.84) [12]	15.49 (4.02) [8]	0.72 (0.44-1)	14.49	0.62	0.92
HSPA8	P11142	change	-0.01 (0.12) [12]	0.06 (0.27) [6]	0.51 (0.2-0.83)	0.08	0.83	0.42
HSPG2	P98160	end	14.58 (1.12) [12]	13.95 (0.45) [9]	0.7 (0.47-0.94)	14.19	0.78	0.67
HSPG2	P98160	start	13.57 (1.48) [10]	13.44 (1.83) [8]	0.55 (0.25-0.85)	12.57	0.38	0.90
HSPG2	P98160	change	-0.06 (0.09) [10]	-0.01 (0.09) [6]	0.67 (0.35-0.98)	-0.08	0.83	0.50
HTRA1	Q92743	start	13.06 (0.71) [9]	13.83 (1.03) [6]	0.7 (0.39-1)	14.23	0.50	1.00
HTRA1	Q92743	end	14.43 (0.56) [10]	14.25 (0.5) [9]	0.5 (0.21-0.79)	14.18	0.78	0.50
HYAL1	Q12794	start	17.7 (1.38) [8]	18.29 (1.18) [5]	0.65 (0.32-0.98)	16.82	1.00	0.38

Protein Name	Uniprot ID	Time	SAH Weaned<br><br>Mean (SD) [N]	SAH Shunted<br><br>Mean (SD) [N]	AUC (95%CI)	Cut-off	Sens.	Spec.
HYOU1	A0A087X054	end	14.17 (0.32) [6]	13.75 (0.38) [7]	0.83 (0.58-1)	13.78	0.71	1.00
ICOSLG	K4DIA0	start	13.86 (1.15) [10]	14 (1.35) [8]	0.5 (0.21-0.79)	13.45	0.75	0.40
ICOSLG	K4DIA0	end	15.17 (0.95) [12]	15.01 (0.54) [9]	0.68 (0.42-0.93)	14.93	0.56	0.83
ICOSLG	K4DIA0	change	-0.13 (0.12) [10]	-0.05 (0.08) [6]	0.7 (0.43-0.97)	-0.14	1.00	0.40
IGF2	P01344	end	14.97 (0.7) [5]	14.48 (0.18) [8]	0.65 (0.22-1)	14.98	1.00	0.60
IGFALS	P35858	start	15.84 (0.69) [12]	16.31 (0.93) [10]	0.71 (0.46-0.96)	16.54	0.60	0.92
IGFALS	P35858	end	14.85 (0.89) [11]	14.7 (0.39) [9]	0.51 (0.23-0.78)	14.73	0.67	0.64
IGFALS	P35858	change	0.07 (0.08) [11]	0.08 (0.05) [8]	0.57 (0.29-0.84)	0.05	0.88	0.45
IGFBP2	P18065	end	14.17 (0.63) [11]	14.23 (0.73) [9]	0.55 (0.26-0.83)	14.52	0.44	0.91
IGFBP3	A6XND0	start	13.92 (1.09) [5]	13.56 (0.72) [6]	0.67 (0.27-1)	13.62	0.67	0.80
IGFBP5	P24593	end	12.18 (1.05) [8]	11.78 (0.54) [6]	0.65 (0.32-0.97)	12.03	0.83	0.62
IGFBP6	P24592	change	-0.11 (0.15) [11]	-0.09 (0.13) [7]	0.51 (0.21-0.8)	-0.12	0.71	0.55
IGFBP6	P24592	end	17.35 (1.07) [12]	17.73 (0.83) [9]	0.65 (0.39-0.9)	17.70	0.67	0.75
IGFBP6	P24592	start	16.11 (1.72) [11]	15.87 (1.52) [9]	0.53 (0.25-0.8)	15.78	0.67	0.64
IGFBP7	Q16270	change	-0.08 (0.11) [12]	0 (0.11) [10]	0.69 (0.46-0.92)	-0.11	1.00	0.42
IGFBP7	Q16270	end	16.75 (1.27) [12]	16.2 (0.95) [10]	0.69 (0.45-0.93)	16.32	0.70	0.75
IGFBP7	Q16270	start	15.65 (1.35) [12]	15.99 (1.77) [11]	0.57 (0.31-0.83)	15.95	0.73	0.58

**Protein Name**	**Uniprot ID**	**Time**	**SAH Weaned** Mean (SD) [N]	**SAH Shunted** Mean (SD) [N]	**AUC (95%CI)**	**Cut-off**	**Sens.**	**Spec.**
IGHA1	A0A286YEY1	change	0.1 (0.12) [12]	0.02 (0.08) [10]	0.72 (0.5-0.95)	0.05	0.70	0.75
IGHA1	A0A286YEY1	end	18.03 (0.81) [12]	18.69 (0.8) [10]	0.73 (0.52-0.95)	18.54	0.60	0.83
IGHA1	A0A286YEY1	start	19.25 (0.93) [12]	19.22 (1.3) [11]	0.52 (0.26-0.77)	17.63	0.27	0.92
IGHA2	A0A286YEY5	end	18.1 (0.68) [12]	18.57 (0.96) [10]	0.61 (0.36-0.85)	17.56	1.00	0.25
IGHA2	A0A286YEY5	start	19.21 (1) [12]	19.47 (1.32) [11]	0.51 (0.26-0.76)	19.41	0.64	0.58
IGHA2	A0A286YEY5	change	0.1 (0.15) [12]	0.04 (0.11) [10]	0.62 (0.37-0.87)	0.08	0.90	0.50
IGHD	A0A0A0MS09	start	16.16 (1.4) [12]	16.23 (1.37) [8]	0.6 (0.32-0.89)	16.16	0.62	0.67
IGHD	A0A0A0MS09	change	0.11 (0.16) [7]	0.02 (0.15) [6]	0.64 (0.31-0.97)	0.07	0.83	0.57
IGHD	A0A0A0MS09	end	15.11 (1.44) [7]	15.54 (1.14) [7]	0.69 (0.38-1)	14.49	1.00	0.43
IGHG1	P01857	change	0.08 (0.07) [12]	0.02 (0.04) [10]	0.75 (0.54-0.96)	0.06	0.90	0.58
IGHG1	P01857	end	19.56 (0.48) [12]	19.73 (0.37) [10]	0.57 (0.31-0.82)	19.76	0.50	0.75
IGHG1	P01857	start	20.53 (0.48) [12]	20.33 (0.8) [11]	0.6 (0.35-0.85)	19.69	0.27	1.00
IGHG2	P01859	change	0.07 (0.07) [12]	0.01 (0.05) [10]	0.79 (0.6-0.99)	0.03	0.70	0.83
IGHG2	P01859	end	19.47 (0.6) [12]	19.89 (0.66) [10]	0.69 (0.45-0.94)	20.06	0.50	1.00
IGHG2	P01859	start	20.37 (0.52) [12]	20.19 (0.68) [11]	0.58 (0.32-0.83)	20.46	0.82	0.50
IGHG3	P01860	end	16.14 (1.09) [12]	16.71 (0.78) [9]	0.68 (0.43-0.92)	15.85	0.89	0.58

Protein Name	Uniprot ID	Time	SAH Weaned Mean (SD) [N]	SAH Shunted Mean (SD) [N]	AUC (95%CI)	Cut-off	Sens.	Spec.
IGHG3	P01860	start	17.7 (0.96) [12]	17.9 (1.07) [10]	0.58 (0.32-0.84)	18.22	0.50	0.83
IGHG3	P01860	change	0.12 (0.12) [12]	0.05 (0.06) [8]	0.72 (0.48-0.96)	0.05	0.62	0.83
IGHG4	A0A286YFJ8	start	16.87 (1.1) [12]	16.47 (0.73) [11]	0.63 (0.39-0.87)	16.88	0.73	0.67
IGHG4	A0A286YFJ8	end	15.82 (1.04) [12]	15.94 (1.18) [10]	0.56 (0.3-0.82)	16.83	0.40	0.83
IGHG4	A0A286YFJ8	change	0.09 (0.14) [12]	0.03 (0.04) [10]	0.72 (0.49-0.94)	0.07	0.90	0.50
IGHM	A0A1B0GUU9	change	0.17 (0.2) [12]	0.07 (0.14) [8]	0.62 (0.36-0.89)	0.19	1.00	0.50
IGHM	A0A1B0GUU9	end	17.05 (1.4) [12]	17.77 (0.93) [9]	0.73 (0.5-0.96)	16.66	1.00	0.58
IGHM	A0A1B0GUU9	start	19.1 (1.43) [12]	19.45 (2.31) [10]	0.68 (0.43-0.92)	19.10	0.80	0.67
IGHV1-18	A0A0C4DH31	start	14.41 (0.95) [8]	15.32 (0.92) [7]	0.79 (0.52-1)	14.82	0.86	0.75
IGHV1-18	A0A0C4DH31	change	0.04 (0.18) [6]	0.05 (0.04) [5]	0.67 (0.29-1)	0.01	1.00	0.50
IGHV1-18	A0A0C4DH31	end	14.46 (0.9) [9]	14.48 (0.82) [8]	0.53 (0.23-0.82)	13.47	1.00	0.22
IGHV1-2	P23083	change	0.08 (0.07) [9]	0.05 (0.03) [6]	0.63 (0.33-0.93)	0.10	1.00	0.44
IGHV1-2	P23083	end	15.25 (0.94) [10]	15.19 (0.59) [8]	0.54 (0.24-0.83)	15.83	1.00	0.40
IGHV1-2	P23083	start	16.22 (0.56) [11]	16.41 (0.51) [8]	0.58 (0.3-0.86)	15.57	1.00	0.27
IGHV1-69	P01742	start	15.15 (1.73) [8]	15.46 (0.7) [5]	0.52 (0.17-0.88)	14.97	0.80	0.38
IGHV1OR15-1	A0A075B7D0	start	17.47 (1.61) [11]	17.32 (1.46) [10]	0.55 (0.28-0.81)	17.91	0.70	0.55
IGHV1OR15-1	A0A075B7D0	end	17.04 (1.33) [12]	16.95 (1.58) [9]	0.5 (0.21-0.79)	16.93	0.56	0.75

Protein Name	Uniprot ID	Time	SAH Weaned<br><br>Mean (SD) [N]	SAH Shunted<br><br>Mean (SD) [N]	AUC (95%CI)	Cut-off	Sens.	Spec.
IGHV1OR15-1	A0A075B7D0	change	0.04 (0.06) [11]	0.03 (0.09) [8]	0.53 (0.25-0.82)	0.06	0.75	0.45
IGHV2-26	A0A0B4J1V2	end	13.63 (0.92) [10]	13.87 (0.51) [9]	0.54 (0.27-0.82)	13.33	0.89	0.40
IGHV2-26	A0A0B4J1V2	change	0.03 (0.09) [8]	0 (0.05) [7]	0.62 (0.32-0.93)	0.07	1.00	0.38
IGHV2-26	A0A0B4J1V2	start	14.19 (1.11) [10]	14.04 (1.11) [9]	0.51 (0.23-0.79)	14.91	0.89	0.30
IGHV2-5	P01817	start	13.21 (1.17) [8]	13.06 (1.23) [5]	0.58 (0.22-0.93)	12.62	0.40	0.88
IGHV2-5	P01817	end	12.77 (1.25) [7]	12.72 (1.38) [7]	0.57 (0.24-0.91)	13.05	0.71	0.57
IGHV3-15	A0A0B4J1V0	change	0.03 (0.08) [12]	-0.01 (0.04) [8]	0.7 (0.43-0.97)	-0.01	0.62	0.83
IGHV3-15	A0A0B4J1V0	start	16.3 (0.68) [12]	15.98 (0.47) [10]	0.64 (0.4-0.89)	16.44	0.90	0.50
IGHV3-15	A0A0B4J1V0	end	16.09 (0.51) [12]	16.16 (0.75) [9]	0.56 (0.27-0.86)	16.63	0.44	0.92
IGHV3-30	P01768	change	0.05 (0.07) [12]	0.02 (0.03) [8]	0.68 (0.43-0.93)	0.02	0.62	0.75
IGHV3-30	P01768	start	17.59 (0.58) [12]	17.27 (0.52) [10]	0.58 (0.32-0.84)	18.04	1.00	0.25
IGHV3-30	P01768	end	17 (0.48) [12]	16.75 (0.43) [9]	0.69 (0.45-0.94)	16.75	0.67	0.83
IGHV3-38	A0A0C4DH36	change	0.01 (0.08) [12]	0.01 (0.03) [8]	0.52 (0.26-0.79)	0.02	0.88	0.42
IGHV3-38	A0A0C4DH36	start	15.51 (0.59) [12]	15.63 (0.56) [10]	0.56 (0.29-0.82)	15.23	0.90	0.50
IGHV3-38	A0A0C4DH36	end	15.46 (0.69) [12]	15.23 (0.64) [9]	0.59 (0.32-0.86)	14.97	0.56	0.75
IGHV3-49	A0A0A0MS15	end	16.88 (0.53) [12]	16.58 (0.9) [9]	0.63 (0.37-0.89)	16.58	0.56	0.75

Protein Name	Uniprot ID	Time	SAH Weaned<br><br>Mean (SD) [N]	SAH Shunted<br><br>Mean (SD) [N]	AUC (95%CI)	Cut-off	Sens.	Spec.
IGHV3-49	A0A0A0MS15	start	17.97 (0.7) [12]	17.86 (0.96) [10]	0.57 (0.31-0.82)	18.35	0.90	0.33
IGHV3-49	A0A0A0MS15	change	0.08 (0.06) [12]	0.06 (0.04) [8]	0.64 (0.38-0.89)	0.08	0.88	0.58
IGHV3-64D	A0A0J9YX35	change	0.01 (0.06) [10]	0 (0.05) [6]	0.53 (0.21-0.85)	0.06	1.00	0.30
IGHV3-64D	A0A0J9YX35	start	15.98 (0.67) [12]	15.89 (0.68) [10]	0.58 (0.32-0.83)	15.79	0.60	0.67
IGHV3-64D	A0A0J9YX35	end	15.9 (0.54) [10]	15.86 (0.75) [7]	0.56 (0.23-0.89)	15.27	0.43	0.90
IGHV3-7	P01780	change	0.05 (0.08) [12]	0.01 (0.02) [8]	0.82 (0.64-1)	0.03	1.00	0.58
IGHV3-7	P01780	start	17.87 (0.69) [12]	17.74 (0.59) [10]	0.52 (0.27-0.78)	18.22	0.90	0.33
IGHV3-7	P01780	end	17.31 (0.56) [12]	17.48 (0.46) [9]	0.65 (0.39-0.91)	17.34	0.78	0.67
IGHV3-72	A0A0B4J1Y9	change	0.06 (0.1) [12]	0.03 (0.03) [8]	0.62 (0.36-0.89)	0.04	0.75	0.67
IGHV3-72	A0A0B4J1Y9	start	17.16 (0.81) [12]	17.28 (0.78) [10]	0.56 (0.3-0.82)	17.14	0.80	0.50
IGHV3-72	A0A0B4J1Y9	end	16.46 (0.58) [12]	16.76 (0.51) [9]	0.66 (0.41-0.9)	16.14	1.00	0.33
IGHV3OR16-12	A0A075B7B8	start	14.16 (0.75) [10]	13.95 (0.76) [6]	0.6 (0.29-0.91)	14.28	0.83	0.50
IGHV3OR16-12	A0A075B7B8	end	13.24 (0.53) [8]	13.08 (0.6) [8]	0.58 (0.27-0.89)	13.13	0.75	0.62
IGHV3OR16-9	A0A0B4J2B5	end	19.82 (0.53) [12]	20.15 (0.75) [10]	0.63 (0.39-0.88)	19.78	0.70	0.67
IGHV3OR16-9	A0A0B4J2B5	start	20.53 (0.46) [12]	20.33 (0.79) [11]	0.58 (0.33-0.84)	20.66	0.82	0.50
IGHV3OR16-9	A0A0B4J2B5	change	0.06 (0.08) [12]	0.01 (0.06) [10]	0.69 (0.46-0.92)	0.04	0.80	0.58

**Protein Name**	**Uniprot ID**	**Time**	**SAH Weaned** Mean (SD) [N]	**SAH Shunted** Mean (SD) [N]	**AUC (95%CI)**	**Cut-off**	**Sens.**	**Spec.**
IGHV4-34	P06331	start	15.98 (0.93) [12]	15.79 (0.99) [10]	0.57 (0.31-0.82)	16.32	0.80	0.50
IGHV4-34	P06331	change	0.08 (0.17) [12]	0.02 (0.05) [8]	0.6 (0.34-0.87)	0.04	0.75	0.50
IGHV4-34	P06331	end	15.33 (0.77) [12]	15.32 (0.57) [9]	0.52 (0.26-0.78)	14.62	1.00	0.25
IGHV5-51	A0A0C4DH38	start	16.47 (0.65) [12]	16.42 (0.95) [10]	0.52 (0.25-0.78)	16.81	0.50	0.67
IGHV5-51	A0A0C4DH38	change	0.04 (0.06) [12]	-0.03 (0.18) [9]	0.7 (0.47-0.94)	0.05	0.89	0.58
IGHV5-51	A0A0C4DH38	end	15.95 (0.57) [12]	16.55 (2.55) [10]	0.5 (0.23-0.77)	16.43	0.30	0.92
IGKC	P01834	start	20.92 (0.8) [12]	21.19 (1.75) [11]	0.51 (0.25-0.76)	21.30	0.82	0.42
IGKC	P01834	change	0.08 (0.1) [12]	0.01 (0.18) [10]	0.68 (0.43-0.92)	0.08	0.90	0.50
IGKC	P01834	end	20.03 (0.51) [12]	20.87 (1.94) [10]	0.68 (0.45-0.92)	20.05	0.80	0.58
IGKV1-12	A0A0C4DH73	end	17.09 (0.61) [12]	17.56 (0.93) [10]	0.62 (0.37-0.88)	17.49	0.50	0.92
IGKV1-12	A0A0C4DH73	change	0.07 (0.1) [12]	-0.01 (0.08) [9]	0.79 (0.58-1)	0.04	0.89	0.75
IGKV1-12	A0A0C4DH73	start	17.98 (1.12) [12]	17.68 (1) [10]	0.59 (0.33-0.85)	17.98	0.80	0.58
IGKV1-16	P04430	end	14.31 (0.42) [7]	14.42 (0.9) [8]	0.61 (0.28-0.93)	14.84	0.50	1.00
IGKV1-16	P04430	change	0.02 (0.05) [6]	0.03 (0.06) [6]	0.58 (0.21-0.96)	0.05	0.83	0.50
IGKV1-16	P04430	start	14.67 (0.78) [9]	14.84 (0.84) [8]	0.53 (0.22-0.83)	15.64	0.25	1.00
IGKV1-17	P01599	end	15.87 (0.84) [12]	15.44 (0.58) [9]	0.63 (0.38-0.88)	16.28	1.00	0.33

**Protein Name**	**Uniprot ID**	**Time**	**SAH Weaned** Mean (SD) [N]	**SAH Shunted** Mean (SD) [N]	**AUC (95%CI)**	**Cut-off**	**Sens.**	**Spec.**
IGKV1-17	P01599	start	16.61 (0.7) [12]	16.37 (0.89) [10]	0.57 (0.31-0.82)	15.55	0.30	0.92
IGKV1-17	P01599	change	0.06 (0.06) [12]	0.04 (0.04) [8]	0.54 (0.27-0.81)	0.09	1.00	0.33
IGKV1-27	A0A075B6S5	start	15.98 (0.88) [9]	16.16 (0.83) [9]	0.58 (0.29-0.87)	15.91	0.78	0.56
IGKV1-27	A0A075B6S5	end	15.63 (0.63) [9]	15.9 (0.32) [8]	0.68 (0.4-0.96)	15.38	1.00	0.44
IGKV1-27	A0A075B6S5	change	0.04 (0.08) [6]	0.03 (0.04) [6]	0.58 (0.21-0.96)	0.05	0.83	0.50
IGKV1-5	P01602	end	16.76 (0.72) [12]	16.82 (0.6) [9]	0.52 (0.25-0.78)	17.16	0.89	0.33
IGKV1-5	P01602	start	16.73 (0.68) [12]	16.96 (0.64) [10]	0.63 (0.38-0.88)	16.87	0.70	0.67
IGKV1-5	P01602	change	0 (0.05) [12]	0 (0.03) [8]	0.53 (0.26-0.8)	-0.02	0.88	0.50
IGKV1-8	A0A0C4DH67	start	17.29 (1) [12]	17.39 (1.24) [10]	0.51 (0.25-0.77)	17.57	0.50	0.67
IGKV1-8	A0A0C4DH67	change	0.07 (0.09) [12]	0.01 (0.1) [9]	0.69 (0.46-0.93)	0.06	0.89	0.50
IGKV1-8	A0A0C4DH67	end	16.46 (0.64) [12]	17.06 (1.07) [10]	0.69 (0.45-0.93)	16.57	0.70	0.75
IGKV1D-33	P01593	change	0.08 (0.09) [12]	0.01 (0.06) [9]	0.71 (0.49-0.94)	0.07	0.89	0.50
IGKV1D-33	P01593	start	18.36 (0.97) [12]	18.19 (1.13) [10]	0.53 (0.27-0.79)	16.77	0.20	1.00
IGKV1D-33	P01593	end	17.41 (0.93) [12]	17.78 (0.47) [10]	0.62 (0.37-0.86)	17.05	1.00	0.42
IGKV1D-37	A0A075B6S9	end	16.19 (0.9) [6]	16.15 (1.57) [6]	0.53 (0.13-0.92)	15.71	0.50	0.83
IGKV1D-37	A0A075B6S9	start	17.86 (1.08) [11]	17.9 (1.27) [7]	0.55 (0.22-0.87)	17.79	0.71	0.64

**Protein Name**	**Uniprot ID**	**Time**	**SAH Weaned**<br><br>Mean (SD) [N]	**SAH Shunted**<br><br>Mean (SD) [N]	**AUC (95%CI)**	**Cut-off**	**Sens.**	**Spec.**
IGKV1D-37	A0A075B6S9	change	0.15 (0.16) [6]	0.12 (0.1) [5]	0.5 (0.11-0.89)	0.10	0.80	0.50
IGKV2-28	A0A075B6P5	end	16.22 (0.5) [11]	16.53 (0.69) [9]	0.64 (0.37-0.9)	16.83	0.33	1.00
IGKV2-28	A0A075B6P5	change	0.05 (0.07) [10]	0.02 (0.04) [8]	0.66 (0.4-0.93)	0.04	0.62	0.70
IGKV2-28	A0A075B6P5	start	16.74 (0.88) [11]	16.93 (0.9) [10]	0.57 (0.31-0.84)	17.01	0.70	0.64
IGKV2-29	A2NJV5	change	0.07 (0.09) [12]	0.05 (0.02) [8]	0.56 (0.29-0.83)	0.08	1.00	0.42
IGKV2-29	A2NJV5	end	16.45 (0.62) [12]	16.14 (0.69) [9]	0.6 (0.34-0.86)	16.20	0.56	0.67
IGKV2-29	A2NJV5	start	17.24 (0.88) [12]	17.12 (1.1) [10]	0.51 (0.24-0.78)	17.30	0.60	0.75
IGKV2-40	A0A087WW87	start	14.2 (0.84) [6]	14.2 (1.17) [5]	0.53 (0.13-0.93)	13.51	0.40	0.83
IGKV2-40	A0A087WW87	end	13.46 (0.55) [7]	14.27 (1.86) [7]	0.65 (0.34-0.97)	13.17	1.00	0.43
IGKV2D-24	A0A075B6R9	start	17.36 (0.95) [12]	17.59 (1.21) [10]	0.58 (0.32-0.85)	17.70	0.70	0.67
IGKV2D-24	A0A075B6R9	end	16.76 (0.68) [12]	16.35 (0.96) [9]	0.66 (0.39-0.92)	16.66	0.78	0.67
IGKV2D-24	A0A075B6R9	change	0.05 (0.09) [12]	0.04 (0.04) [8]	0.55 (0.28-0.83)	0.03	0.75	0.50
IGKV2D-29	A0A075B6S2	start	14.35 (0.76) [7]	14.75 (0.7) [5]	0.6 (0.21-0.99)	15.22	0.40	1.00
IGKV3-15	P01624	start	17.75 (0.51) [12]	17.63 (0.67) [10]	0.56 (0.3-0.82)	17.08	0.30	0.92
IGKV3-15	P01624	change	0.02 (0.07) [12]	-0.03 (0.03) [8]	0.76 (0.54-0.98)	0.00	0.88	0.67
IGKV3-15	P01624	end	17.46 (0.86) [12]	18.19 (0.67) [9]	0.78 (0.57-0.98)	17.47	0.89	0.58

**Protein Name**	**Uniprot ID**	**Time**	**SAH Weaned** Mean (SD) [N]	**SAH Shunted** Mean (SD) [N]	**AUC (95%CI)**	**Cut-off**	**Sens.**	**Spec.**
IGKV3-20	P01619	start	19.16 (0.54) [12]	19.33 (1.18) [11]	0.53 (0.27-0.79)	19.92	0.36	1.00
IGKV3-20	P01619	change	0.05 (0.06) [12]	0.01 (0.11) [10]	0.68 (0.43-0.92)	0.02	0.60	0.75
IGKV3-20	P01619	end	18.55 (0.58) [12]	19.1 (1.32) [10]	0.62 (0.35-0.89)	18.85	0.60	0.75
IGKV3-7	A0A075B6H7	start	18.33 (1.22) [10]	18.84 (1.72) [10]	0.6 (0.34-0.86)	18.20	0.70	0.60
IGKV3-7	A0A075B6H7	change	0.02 (0.15) [9]	-0.02 (0.08) [8]	0.54 (0.25-0.84)	0.17	1.00	0.22
IGKV3-7	A0A075B6H7	end	18.82 (1.56) [11]	19.32 (1.12) [9]	0.62 (0.36-0.88)	19.16	0.67	0.64
IGKV3D-11	A0A0A0MRZ8	end	17.62 (0.58) [12]	17.58 (0.45) [9]	0.52 (0.26-0.78)	18.06	0.89	0.33
IGKV3D-11	A0A0A0MRZ8	change	0.05 (0.09) [12]	0.02 (0.01) [8]	0.6 (0.33-0.88)	0.04	1.00	0.50
IGKV3D-11	A0A0A0MRZ8	start	18.09 (0.59) [12]	18.02 (0.45) [10]	0.58 (0.3-0.85)	18.18	0.70	0.75
IGKV3D-15	A0A087WSY6	change	0.07 (0.06) [11]	0.05 (0.05) [8]	0.62 (0.35-0.9)	0.05	0.50	0.82
IGKV3D-15	A0A087WSY6	start	18.2 (0.83) [12]	18.24 (1.01) [10]	0.58 (0.3-0.85)	18.37	0.70	0.67
IGKV3D-15	A0A087WSY6	end	17.17 (1.09) [11]	16.78 (0.78) [9]	0.6 (0.33-0.86)	17.49	0.89	0.45
IGKV3D-20	A0A0C4DH25	start	19.42 (1.12) [12]	19.41 (1.25) [10]	0.52 (0.26-0.77)	18.94	0.40	0.75
IGKV3D-20	A0A0C4DH25	change	0.1 (0.1) [12]	0.04 (0.06) [8]	0.72 (0.48-0.96)	0.05	0.62	0.83
IGKV3D-20	A0A0C4DH25	end	18.24 (1.06) [12]	18.17 (0.73) [9]	0.52 (0.25-0.78)	18.82	0.89	0.33
IGKV4-1	P06312	change	0.07 (0.06) [12]	0 (0.04) [8]	0.81 (0.62-1)	0.04	1.00	0.58

**Protein Name**	**Uniprot ID**	**Time**	**SAH Weaned**<br>Mean (SD) [N]	**SAH Shunted**<br>Mean (SD) [N]	**AUC (95%CI)**	**Cut-off**	**Sens.**	**Spec.**
IGKV4-1	P06312	start	18 (0.6) [12]	17.75 (0.9) [10]	0.52 (0.25-0.78)	17.38	0.30	0.92
IGKV4-1	P06312	end	17.15 (0.53) [12]	17.63 (0.47) [9]	0.78 (0.57-0.99)	17.42	0.89	0.58
IGKV6D-21	A0A0A0MT36	start	14.71 (1.35) [7]	15.13 (0.88) [6]	0.62 (0.28-0.96)	15.23	0.50	0.86
IGLC3	P0DOY3	change	0.07 (0.09) [12]	0 (0.05) [8]	0.75 (0.53-0.97)	0.05	1.00	0.50
IGLC3	P0DOY3	end	20.13 (0.6) [12]	20.54 (0.29) [9]	0.72 (0.48-0.96)	20.13	1.00	0.58
IGLC3	P0DOY3	start	20.95 (0.36) [12]	20.67 (1.03) [10]	0.54 (0.27-0.81)	20.20	0.30	1.00
IGLL1	P15814	start	16.58 (1.07) [10]	16.95 (0.66) [7]	0.63 (0.33-0.92)	16.37	0.86	0.60
IGLL5	A0A0B4J231	end	18.01 (0.65) [12]	18.2 (0.65) [10]	0.58 (0.33-0.84)	18.26	0.50	0.75
IGLL5	A0A0B4J231	start	18.92 (0.56) [12]	18.75 (1.06) [11]	0.51 (0.25-0.77)	18.32	0.36	0.92
IGLL5	A0A0B4J231	change	0.09 (0.13) [12]	0.02 (0.05) [10]	0.67 (0.43-0.9)	0.04	0.60	0.75
IGLV1-47	P01700	change	0.03 (0.06) [12]	-0.03 (0.15) [9]	0.59 (0.32-0.87)	-0.01	0.33	1.00
IGLV1-47	P01700	end	16.88 (0.6) [12]	17.26 (2.35) [10]	0.58 (0.3-0.85)	16.22	0.50	0.92
IGLV1-47	P01700	start	17.19 (0.81) [12]	16.95 (0.73) [10]	0.57 (0.31-0.82)	16.70	0.50	0.75
IGLV1-51	P01701	change	0.09 (0.09) [10]	0.04 (0.06) [7]	0.71 (0.45-0.98)	0.05	0.71	0.80
IGLV1-51	P01701	end	15.57 (0.54) [10]	15.75 (0.88) [8]	0.56 (0.24-0.89)	15.99	0.50	0.90
IGLV1-51	P01701	start	16.63 (0.79) [12]	16.82 (0.89) [9]	0.56 (0.3-0.83)	17.63	0.33	0.92

**Protein Name**	**Uniprot ID**	**Time**	**SAH Weaned** Mean (SD) [N]	**SAH Shunted** Mean (SD) [N]	**AUC (95%CI)**	**Cut-off**	**Sens.**	**Spec.**
IGLV3-10	A0A075B6K4	change	0.08 (0.08) [8]	0.04 (0.06) [7]	0.64 (0.33-0.95)	0.11	1.00	0.50
IGLV3-10	A0A075B6K4	end	15.3 (0.87) [10]	15.34 (0.51) [9]	0.5 (0.21-0.79)	15.49	0.56	0.70
IGLV3-10	A0A075B6K4	start	16.3 (0.73) [9]	16.28 (1.17) [7]	0.63 (0.32-0.95)	16.04	0.86	0.67
IGLV3-19	P01714	change	0.04 (0.05) [11]	0.02 (0.03) [7]	0.62 (0.34-0.91)	0.03	0.86	0.64
IGLV3-19	P01714	end	14.66 (0.61) [11]	14.53 (0.57) [9]	0.58 (0.3-0.85)	15.28	1.00	0.27
IGLV3-19	P01714	start	15.09 (0.43) [12]	15.05 (0.54) [9]	0.5 (0.2-0.8)	15.37	0.44	0.83
IGLV3-21	P80748	end	15.34 (0.95) [10]	16.46 (0.84) [7]	0.79 (0.54-1)	16.40	0.57	1.00
IGLV3-21	P80748	change	0.08 (0.17) [9]	0.01 (0.04) [6]	0.67 (0.37-0.96)	0.06	1.00	0.33
IGLV3-21	P80748	start	15.75 (1.06) [11]	16.51 (1.09) [9]	0.72 (0.48-0.96)	16.26	0.78	0.73
IGLV3-25	P01717	change	0.13 (0.16) [11]	0.03 (0.05) [8]	0.86 (0.7-1)	0.10	1.00	0.64
IGLV3-25	P01717	end	14.5 (0.56) [12]	15.07 (0.78) [9]	0.72 (0.49-0.95)	15.00	0.67	0.83
IGLV3-25	P01717	start	15.9 (0.7) [11]	15.66 (1.2) [10]	0.53 (0.25-0.8)	15.80	0.70	0.64
IGLV3-9	A0A075B6K5	change	0.11 (0.12) [12]	0.05 (0.08) [8]	0.64 (0.38-0.89)	0.15	1.00	0.33
IGLV3-9	A0A075B6K5	start	16.26 (1.18) [12]	16.71 (1.49) [10]	0.62 (0.36-0.87)	16.49	0.70	0.67
IGLV3-9	A0A075B6K5	end	14.92 (0.71) [12]	15.64 (0.56) [9]	0.79 (0.57-1)	15.36	0.78	0.83
IGLV6-57	P01721	change	0.06 (0.08) [10]	0.02 (0.05) [8]	0.64 (0.36-0.91)	0.09	1.00	0.40

Protein Name	Uniprot ID	Time	SAH Weaned<br>Mean (SD) [N]	SAH Shunted<br>Mean (SD) [N]	AUC (95%CI)	Cut-off	Sens.	Spec.
IGLV6-57	P01721	end	14.59 (0.82) [10]	14.95 (0.83) [9]	0.66 (0.38-0.93)	15.05	0.67	0.80
IGLV6-57	P01721	start	15.37 (0.72) [12]	15.54 (0.95) [10]	0.56 (0.29-0.82)	15.66	0.50	0.75
IGLV7-46	A0A075B6I9	change	0.08 (0.13) [11]	0.04 (0.07) [8]	0.53 (0.23-0.83)	0.02	0.38	0.91
IGLV7-46	A0A075B6I9	end	15.42 (0.67) [11]	15.24 (1.09) [9]	0.53 (0.24-0.81)	15.03	0.44	0.82
IGLV7-46	A0A075B6I9	start	16.15 (0.46) [12]	16.2 (0.97) [10]	0.54 (0.26-0.82)	16.40	0.50	0.75
IGLV8-61	A0A075B6I0	end	15.71 (0.88) [7]	15.44 (0.59) [8]	0.62 (0.3-0.95)	15.67	0.88	0.57
IGLV8-61	A0A075B6I0	start	16.01 (0.78) [8]	17.06 (0.75) [8]	0.84 (0.63-1)	16.28	1.00	0.62
IGSF8	Q969P0	end	15.34 (0.6) [11]	14.78 (0.42) [9]	0.79 (0.58-1)	14.97	0.78	0.82
IGSF8	Q969P0	start	14.45 (1.03) [9]	14.55 (1.33) [5]	0.51 (0.16-0.87)	16.19	0.20	1.00
IL31RA	Q8NI17	end	23.59 (0.59) [8]	23.34 (0.53) [8]	0.67 (0.39-0.96)	23.57	0.75	0.62
IL6ST	P40189	end	13.74 (0.52) [8]	13.27 (0.45) [6]	0.75 (0.46-1)	13.08	0.50	1.00
IMPAD1	Q9NX62	end	13.71 (0.69) [8]	12.56 (0.74) [7]	0.86 (0.65-1)	13.10	0.86	0.88
ISLR	O14498	change	-0.06 (0.09) [9]	-0.1 (0.08) [5]	0.51 (0.17-0.85)	-0.11	0.60	0.67
ISLR	O14498	end	15.09 (0.67) [12]	15.66 (0.41) [9]	0.83 (0.61-1)	15.73	0.78	1.00
ISLR	O14498	start	14.1 (1.42) [9]	13.72 (1.19) [7]	0.54 (0.22-0.85)	15.20	0.86	0.44
ITIH1	P19827	change	0.11 (0.11) [12]	0.1 (0.13) [10]	0.57 (0.31-0.82)	0.06	0.40	0.83
ITIH1	P19827	start	16.96 (0.72) [12]	17.79 (2.12) [11]	0.77 (0.54-1)	17.56	0.73	0.92

Protein Name	Uniprot ID	Time	SAH Weaned<br><br>Mean (SD) [N]	SAH Shunted<br><br>Mean (SD) [N]	AUC (95%CI)	Cut-off	Sens.	Spec.
ITIH1	P19827	end	15.57 (0.72) [12]	15.8 (0.5) [10]	0.63 (0.38-0.89)	15.57	0.80	0.58
ITIH2	P19823	start	17.03 (0.86) [12]	17.26 (1.04) [11]	0.63 (0.38-0.88)	17.48	0.64	0.75
ITIH2	P19823	change	0.12 (0.12) [12]	0.05 (0.08) [10]	0.71 (0.48-0.94)	0.09	0.80	0.67
ITIH2	P19823	end	15.56 (0.73) [12]	16.05 (0.62) [10]	0.73 (0.51-0.96)	16.03	0.60	0.83
ITIH3	Q06033	end	14.49 (0.71) [11]	14.65 (0.51) [9]	0.62 (0.35-0.89)	14.41	0.78	0.64
ITIH3	Q06033	change	0.1 (0.13) [11]	0.05 (0.05) [8]	0.57 (0.29-0.84)	0.07	0.75	0.55
ITIH3	Q06033	start	15.63 (0.84) [12]	15.6 (0.81) [10]	0.53 (0.28-0.79)	16.26	1.00	0.33
ITIH4	Q14624	end	16.33 (0.68) [12]	16.73 (0.82) [10]	0.7 (0.46-0.94)	16.51	0.80	0.75
ITIH4	Q14624	change	0.09 (0.1) [12]	0.03 (0.06) [10]	0.67 (0.41-0.92)	0.08	1.00	0.58
ITIH4	Q14624	start	17.47 (0.75) [12]	17.51 (0.76) [11]	0.51 (0.26-0.76)	18.17	1.00	0.17
ITIH5	C9J2H1	end	13.22 (1.04) [6]	13.24 (0.71) [5]	0.6 (0.22-0.98)	13.89	1.00	0.33
ITM2B	Q9Y287	end	13.81 (1.16) [8]	12.2 (1.63) [8]	0.8 (0.56-1)	12.72	0.75	0.88
ITPR2	Q14571	change	0.09 (0.22) [12]	0.01 (0.07) [8]	0.65 (0.39-0.9)	0.05	0.88	0.58
ITPR2	Q14571	start	19.07 (0.78) [12]	18.57 (1) [10]	0.62 (0.38-0.87)	19.01	0.70	0.58
ITPR2	Q14571	end	18.23 (1.13) [12]	18.43 (0.81) [9]	0.55 (0.29-0.81)	17.74	0.89	0.42
JCHAIN	D6RD17	change	0.11 (0.16) [11]	0.04 (0.1) [8]	0.56 (0.27-0.85)	0.13	1.00	0.45

Protein Name	Uniprot ID	Time	SAH Weaned<br><br>Mean (SD) [N]	SAH Shunted<br><br>Mean (SD) [N]	AUC (95%CI)	Cut-off	Sens.	Spec.
JCHAIN	D6RD17	start	18.23 (1.12) [12]	18.32 (1.89) [10]	0.61 (0.35-0.86)	18.32	0.80	0.58
JCHAIN	D6RD17	end	16.79 (1.43) [11]	17.17 (1.01) [9]	0.52 (0.24-0.79)	17.83	0.33	0.91
KHSRP	A0A087WTP3	end	13.99 (0.67) [6]	14 (1.46) [5]	0.57 (0.17-0.97)	14.18	0.60	0.67
KIAA1549L	H0YDE5	end	13.58 (0.87) [10]	13.32 (0.94) [7]	0.59 (0.28-0.89)	13.27	0.57	0.70
KLK6	Q92876	end	15.97 (1.21) [12]	15.61 (0.55) [9]	0.69 (0.46-0.93)	16.18	0.89	0.58
KLK6	Q92876	start	15.19 (1.01) [11]	14.71 (1.39) [10]	0.7 (0.46-0.94)	15.15	0.90	0.55
KLK6	Q92876	change	-0.07 (0.12) [11]	-0.04 (0.08) [8]	0.57 (0.29-0.84)	-0.15	1.00	0.27
KLKB1	H0YAC1	change	0.11 (0.11) [11]	0.08 (0.07) [8]	0.58 (0.3-0.86)	0.10	0.62	0.64
KLKB1	H0YAC1	end	14.11 (0.87) [11]	14.08 (0.49) [9]	0.59 (0.31-0.86)	14.32	0.44	0.82
KLKB1	H0YAC1	start	15.57 (0.8) [12]	15.85 (1.15) [10]	0.7 (0.44-0.96)	16.24	0.60	0.92
KNG1	P01042	end	16.94 (0.61) [12]	17.33 (0.28) [10]	0.79 (0.57-1)	16.90	1.00	0.75
KNG1	P01042	change	0.05 (0.06) [12]	0.02 (0.04) [9]	0.65 (0.4-0.9)	0.05	0.89	0.58
KNG1	P01042	start	17.61 (0.69) [12]	17.97 (0.81) [10]	0.69 (0.44-0.94)	18.21	0.70	0.75
KRT1	P04264	end	15.48 (2.24) [11]	15.15 (0.85) [5]	0.56 (0.27-0.85)	13.92	1.00	0.36
KRT1	P04264	start	15.27 (1.01) [8]	15.57 (4.95) [8]	0.69 (0.39-0.98)	13.62	0.50	1.00
KRT10	P13645	end	15.31 (2.25) [6]	13.38 (1.47) [6]	0.72 (0.4-1)	15.21	1.00	0.50

**Protein Name**	**Uniprot ID**	**Time**	**SAH Weaned**<br>Mean (SD) [N]	**SAH Shunted**<br>Mean (SD) [N]	**AUC (95%CI)**	**Cut-off**	**Sens.**	**Spec.**
KRT2	P35908	end	14.81 (2.57) [10]	14.11 (1.38) [5]	0.54 (0.21-0.87)	14.26	0.60	0.70
LAMA2	A0A087WX80	end	9.95 (0.98) [7]	10.47 (1.46) [8]	0.57 (0.25-0.89)	9.49	0.88	0.43
LAMP2	P13473	end	15.27 (0.88) [12]	14.58 (0.54) [9]	0.75 (0.53-0.97)	15.24	0.89	0.58
LAMP2	P13473	change	-0.11 (0.1) [11]	-0.03 (0.05) [7]	0.77 (0.54-1)	-0.07	1.00	0.64
LAMP2	P13473	start	13.91 (1.16) [11]	13.81 (1.24) [9]	0.64 (0.36-0.91)	14.02	0.89	0.55
LBP	P18428	end	14 (0.83) [8]	13.96 (0.64) [8]	0.53 (0.22-0.84)	14.67	1.00	0.25
LBP	P18428	change	0.03 (0.07) [6]	0.04 (0.03) [7]	0.55 (0.16-0.94)	0.00	0.86	0.50
LBP	P18428	start	14.46 (0.63) [8]	14.63 (0.75) [9]	0.54 (0.24-0.84)	14.86	0.44	0.75
LCAT	P04180	change	0.03 (0.05) [9]	0 (0.02) [8]	0.61 (0.31-0.91)	0.01	0.75	0.67
LCAT	P04180	end	14.65 (0.49) [10]	14.77 (0.45) [9]	0.56 (0.28-0.84)	14.87	0.56	0.70
LCAT	P04180	start	14.89 (0.71) [11]	14.74 (0.47) [10]	0.51 (0.24-0.77)	14.68	0.60	0.64
LCN2	P80188	end	15.22 (1.35) [10]	15.01 (1.13) [9]	0.62 (0.34-0.91)	15.13	0.78	0.70
LCP1	P13796	end	15.68 (1.16) [12]	14.88 (0.92) [9]	0.72 (0.5-0.95)	15.73	0.89	0.58
LCP1	P13796	start	14.16 (0.58) [10]	14.14 (0.98) [5]	0.62 (0.25-0.99)	13.92	0.60	0.80
LDHA	P00338	start	14.45 (0.93) [9]	14.17 (1.53) [6]	0.61 (0.21-1)	13.23	0.50	1.00
LDHA	P00338	end	15.38 (1.16) [12]	14.95 (0.51) [9]	0.67 (0.42-0.91)	15.75	1.00	0.42
LDHB	P07195	end	15.37 (0.63) [12]	15.03 (0.51) [9]	0.69 (0.43-0.94)	15.11	0.67	0.83

Protein Name	Uniprot ID	Time	SAH Weaned<br><br>Mean (SD) [N]	SAH Shunted<br><br>Mean (SD) [N]	AUC (95%CI)	Cut-off	Sens.	Spec.
LDHB	P07195	start	14.78 (1.11) [12]	15.7 (4.02) [9]	0.53 (0.26-0.8)	15.10	0.78	0.42
LDHB	P07195	change	-0.06 (0.11) [12]	0.05 (0.24) [7]	0.58 (0.3-0.86)	-0.04	0.71	0.58
LGALS1	P09382	end	15.19 (1.09) [12]	15.04 (0.54) [9]	0.51 (0.25-0.77)	15.07	0.56	0.67
LGALS3BP	Q08380	end	16.58 (0.75) [12]	15.66 (0.47) [9]	0.86 (0.69-1)	15.98	0.89	0.83
LGALS3BP	Q08380	start	15.52 (0.74) [12]	15.47 (0.77) [10]	0.52 (0.25-0.8)	14.97	0.40	0.92
LGALS3BP	Q08380	change	-0.09 (0.09) [12]	-0.01 (0.04) [8]	0.82 (0.62-1)	-0.07	1.00	0.67
LIAS	A0A1W2PNQ5	end	14.58 (1.24) [9]	14.86 (0.87) [8]	0.62 (0.34-0.91)	14.44	0.75	0.56
LIAS	A0A1W2PNQ5	start	15.58 (1.24) [8]	15.85 (1.17) [10]	0.59 (0.3-0.87)	16.05	0.50	0.75
LIAS	A0A1W2PNQ5	change	0.12 (0.06) [6]	0.05 (0.05) [7]	0.79 (0.51-1)	0.10	0.86	0.67
LMAN2	D6RBV2	start	13.53 (0.91) [7]	14.6 (1.35) [5]	0.74 (0.43-1)	13.19	1.00	0.57
LMAN2	D6RBV2	end	14.86 (0.28) [11]	14.74 (0.35) [9]	0.62 (0.34-0.89)	14.88	0.78	0.55
LRG1	P02750	start	16.83 (0.35) [12]	17.07 (0.41) [10]	0.72 (0.48-0.96)	16.88	0.80	0.75
LRG1	P02750	change	-0.01 (0.04) [12]	0 (0.03) [8]	0.51 (0.23-0.79)	-0.06	1.00	0.17
LRG1	P02750	end	16.84 (0.43) [12]	17.16 (0.55) [9]	0.69 (0.45-0.94)	16.85	0.78	0.67
LRP1	Q07954	end	13.33 (1.02) [8]	12.5 (0.54) [8]	0.78 (0.53-1)	13.28	1.00	0.62
LRRC4B	Q9NT99	end	13.62 (0.93) [9]	13.06 (0.73) [7]	0.67 (0.37-0.96)	13.39	0.86	0.67
LSAMP	H3BLU2	end	14.63 (0.92) [12]	14.12 (0.54) [9]	0.77 (0.55-0.98)	14.67	0.89	0.67

**Protein Name**	**Uniprot ID**	**Time**	**SAH Weaned**<br><br>Mean (SD) [N]	**SAH Shunted**<br><br>Mean (SD) [N]	**AUC (95%CI)**	**Cut-off**	**Sens.**	**Spec.**
LTBP2	G3V3X5	end	12.44 (1.2) [9]	13.15 (0.6) [8]	0.69 (0.42-0.97)	12.84	0.88	0.56
LTF	E7EQB2	end	15.01 (1.59) [12]	14 (0.96) [9]	0.69 (0.45-0.92)	14.90	0.89	0.58
LUM	P51884	end	15.7 (0.37) [12]	15.51 (0.29) [10]	0.68 (0.45-0.92)	15.56	0.70	0.75
LUM	P51884	change	0.03 (0.03) [12]	0.01 (0.03) [9]	0.62 (0.35-0.89)	-0.01	0.33	1.00
LUM	P51884	start	16.1 (0.41) [12]	15.9 (0.59) [10]	0.64 (0.37-0.91)	15.89	0.60	0.92
LY6H	O94772	end	11.39 (0.66) [9]	10.97 (0.59) [8]	0.67 (0.37-0.96)	11.36	0.88	0.67
LYVE1	Q9Y5Y7	change	-0.1 (0.13) [11]	-0.09 (0.08) [7]	0.57 (0.29-0.85)	-0.18	1.00	0.36
LYVE1	Q9Y5Y7	start	14.26 (1.57) [11]	13.84 (1.47) [9]	0.61 (0.34-0.88)	13.11	0.44	0.91
LYVE1	Q9Y5Y7	end	15.93 (1.29) [12]	15.57 (0.82) [9]	0.66 (0.41-0.9)	16.32	0.89	0.50
LYZ	A0A0B4J259	change	-0.1 (0.09) [12]	-0.1 (0.09) [8]	0.53 (0.26-0.8)	-0.20	1.00	0.17
LYZ	A0A0B4J259	end	17.44 (0.95) [12]	17.55 (0.81) [9]	0.56 (0.29-0.82)	17.03	0.89	0.42
LYZ	A0A0B4J259	start	15.88 (1.37) [12]	15.54 (1.34) [10]	0.59 (0.33-0.85)	15.26	0.60	0.75
MAG	P20916	end	13.02 (0.8) [7]	12.36 (0.54) [5]	0.71 (0.39-1)	13.21	1.00	0.43
MAN1A1	P33908	start	14.02 (0.71) [6]	13.49 (1.3) [6]	0.61 (0.25-0.97)	14.04	0.67	0.67
MAN1A1	P33908	end	14.04 (0.6) [11]	13.89 (0.27) [8]	0.56 (0.28-0.83)	14.32	1.00	0.27
MAN1C1	Q9NR34	end	13.37 (0.33) [7]	12.59 (1.14) [6]	0.79 (0.46-1)	12.83	0.67	1.00
MAN2A2	P49641	end	13.86 (0.91) [9]	13.34 (0.45) [7]	0.73 (0.46-1)	13.14	0.57	0.89

**Protein Name**	**Uniprot ID**	**Time**	**SAH Weaned** Mean (SD) [N]	**SAH Shunted** Mean (SD) [N]	**AUC (95%CI)**	**Cut-off**	**Sens.**	**Spec.**
MARCKS	P29966	end	12.4 (0.74) [10]	12.27 (0.67) [9]	0.56 (0.28-0.83)	12.95	0.89	0.30
MARCKS	P29966	start	12.11 (0.86) [9]	12.2 (0.99) [5]	0.6 (0.22-0.98)	13.24	0.40	1.00
MASP1	P48740	start	13.25 (0.54) [7]	13.99 (0.44) [7]	0.86 (0.65-1)	13.42	1.00	0.71
MBL2	P11226	start	14.16 (1.41) [7]	14.12 (0.46) [7]	0.51 (0.16-0.86)	15.13	1.00	0.29
MBL2	P11226	end	11.4 (1.55) [6]	10.57 (0.98) [5]	0.63 (0.27-1)	12.27	1.00	0.33
MCAM	P43121	end	14.35 (0.72) [11]	14.22 (0.8) [9]	0.57 (0.28-0.85)	14.27	0.67	0.64
MDH1	P40925	end	15.76 (0.69) [12]	15.26 (0.83) [9]	0.69 (0.44-0.95)	15.73	0.78	0.75
MEGF8	Q7Z7M0	end	13.71 (0.85) [7]	12.91 (0.63) [8]	0.77 (0.47-1)	12.98	0.75	0.86
MFAP4	K7ES70	end	14.25 (1.16) [5]	13.36 (0.68) [5]	0.76 (0.41-1)	13.27	0.60	1.00
MGAT1	P26572	end	14.25 (0.35) [8]	14.09 (0.18) [8]	0.62 (0.32-0.93)	14.42	1.00	0.38
MGP	P08493	end	15.67 (0.79) [11]	16.34 (0.43) [8]	0.75 (0.52-0.98)	16.18	0.75	0.73
MIF	P14174	end	16.41 (0.93) [12]	15.84 (0.69) [8]	0.71 (0.46-0.96)	16.08	0.75	0.75
MMP2	P08253	end	15.22 (0.6) [11]	15.16 (0.52) [9]	0.57 (0.3-0.83)	15.44	0.67	0.64
MMP2	P08253	start	14.22 (1.85) [6]	13.99 (1.04) [7]	0.55 (0.17-0.93)	14.12	0.71	0.67
MMP2	P08253	change	-0.06 (0.12) [6]	-0.06 (0.08) [5]	0.6 (0.2-1)	-0.11	0.60	0.83
MOG	A0A0G2JHA9	end	13.68 (0.99) [10]	12.96 (0.74) [9]	0.72 (0.48-0.96)	14.09	1.00	0.40
MRC1	P22897	end	14.98 (0.81) [11]	14.72 (0.52) [8]	0.58 (0.3-0.86)	15.54	1.00	0.36
MSN	P26038	end	13.83 (1.53) [11]	13.29 (0.8) [9]	0.62 (0.35-0.88)	13.17	0.56	0.82

**Protein Name**	**Uniprot ID**	**Time**	**SAH Weaned** Mean (SD) [N]	**SAH Shunted** Mean (SD) [N]	**AUC (95%CI)**	**Cut-off**	**Sens.**	**Spec.**
MST1	G3XAK1	start	14.04 (0.92) [6]	14.1 (1.16) [5]	0.53 (0.11-0.96)	14.77	0.60	0.83
MST1	G3XAK1	end	13.13 (0.61) [8]	13.26 (0.47) [7]	0.55 (0.23-0.88)	13.14	0.71	0.62
MYH11	P35749	end	15.62 (0.75) [7]	16.1 (0.72) [9]	0.67 (0.38-0.96)	16.10	0.56	0.86
MYH9	P35579	end	13.27 (0.59) [6]	13.52 (2.22) [8]	0.6 (0.28-0.93)	12.46	0.38	1.00
NBL1	A0A087WTY6	end	18.16 (0.59) [11]	17.96 (0.36) [9]	0.6 (0.33-0.86)	18.31	0.89	0.45
NBL1	A0A087WTY6	start	17.44 (1.09) [6]	17.11 (1.59) [5]	0.53 (0.12-0.95)	17.55	0.60	0.67
NCAM1	P13591	change	-0.08 (0.09) [11]	-0.08 (0.08) [8]	0.51 (0.23-0.79)	-0.17	0.25	0.91
NCAM1	P13591	start	14.33 (0.93) [11]	13.77 (1.5) [9]	0.64 (0.37-0.9)	14.55	0.89	0.45
NCAM1	P13591	end	15.37 (0.97) [12]	15.33 (0.74) [10]	0.68 (0.44-0.93)	15.47	0.80	0.67
NCAM2	H9KV31	end	14.74 (0.57) [11]	14.11 (0.31) [9]	0.85 (0.68-1)	14.79	1.00	0.64
NCAN	O14594	end	14.48 (0.64) [9]	13.9 (0.89) [9]	0.75 (0.5-1)	13.97	0.78	0.78
NEFM	E7EMV2	end	13.86 (3.22) [7]	14.06 (2.58) [5]	0.51 (0.11-0.92)	14.38	0.60	0.71
NEGR1	Q7Z3B1	end	14.38 (1.32) [12]	13.48 (1.19) [8]	0.7 (0.45-0.95)	14.40	0.88	0.58
NEGR1	Q7Z3B1	start	13.5 (1.36) [9]	13.74 (1.59) [5]	0.51 (0.16-0.87)	13.75	0.80	0.44
NELL2	F8VVB6	end	14.74 (0.63) [12]	13.84 (0.91) [9]	0.83 (0.63-1)	13.92	0.78	0.92
NEO1	Q92859	start	13.87 (1.09) [7]	13.74 (1.14) [5]	0.51 (0.14-0.89)	14.08	0.80	0.43

Protein Name	Uniprot ID	Time	SAH Weaned<br><br>Mean (SD) [N]	SAH Shunted<br><br>Mean (SD) [N]	AUC (95%CI)	Cut-off	Sens.	Spec.
NEO1	Q92859	end	14.36 (0.47) [11]	13.67 (0.59) [8]	0.8 (0.58-1)	13.62	0.50	1.00
NFASC	O94856	end	14.03 (0.53) [8]	13.45 (0.7) [7]	0.71 (0.42-1)	13.67	0.71	0.75
NID1	P14543	end	13.66 (0.59) [10]	13.6 (0.49) [8]	0.58 (0.29-0.86)	14.02	0.88	0.40
NID2	Q14112	end	14.33 (0.63) [8]	13.84 (0.81) [8]	0.64 (0.35-0.93)	14.46	1.00	0.38
NPC2	E7EMS2	start	15.56 (0.85) [9]	14.95 (1.29) [7]	0.68 (0.38-0.98)	15.83	0.86	0.56
NPC2	E7EMS2	end	16.48 (0.92) [11]	15.99 (0.86) [10]	0.7 (0.46-0.94)	16.49	0.90	0.55
NPC2	E7EMS2	change	-0.09 (0.11) [8]	-0.05 (0.09) [6]	0.56 (0.23-0.9)	-0.07	0.67	0.62
NPDC1	Q5SPY9	end	13.18 (0.95) [11]	12.57 (0.71) [9]	0.7 (0.44-0.95)	12.52	0.67	0.82
NPPC	P23582	end	13.65 (0.74) [7]	13.31 (0.25) [7]	0.63 (0.28-0.98)	13.97	1.00	0.43
NPTX1	Q15818	end	14.61 (1.19) [11]	14.03 (0.95) [9]	0.7 (0.45-0.95)	14.45	0.89	0.64
NPTX1	Q15818	start	13.91 (1.7) [8]	13.39 (1.74) [8]	0.59 (0.29-0.9)	13.37	0.75	0.62
NPTX1	Q15818	change	0.01 (0.22) [7]	-0.04 (0.06) [6]	0.62 (0.28-0.96)	-0.09	1.00	0.43
NPTXR	A0A1X7SBT7	end	14.5 (1.15) [12]	14.21 (1.02) [7]	0.57 (0.29-0.85)	14.65	0.86	0.50
NRCAM	C9JYY6	end	15 (0.81) [12]	14.62 (0.83) [9]	0.67 (0.42-0.92)	14.43	0.44	0.92
NRCAM	C9JYY6	start	14.02 (0.91) [9]	14.09 (1.25) [7]	0.54 (0.22-0.86)	13.80	0.71	0.67
NRCAM	C9JYY6	change	-0.09 (0.07) [9]	-0.03 (0.04) [5]	0.78 (0.48-1)	-0.02	0.60	1.00
NRN1	A0A087WWT2	end	14.78 (0.55) [7]	14.54 (0.4) [8]	0.66 (0.35-0.97)	15.09	1.00	0.43

**Protein Name**	**Uniprot ID**	**Time**	**SAH Weaned**<br><br>Mean (SD) [N]	**SAH Shunted**<br><br>Mean (SD) [N]	**AUC (95%CI)**	**Cut-off**	**Sens.**	**Spec.**
NRP1	E7EX60	end	13.39 (0.47) [10]	13.42 (0.5) [9]	0.56 (0.28-0.83)	13.18	0.78	0.50
NRXN2	G5E9G7	end	14.47 (0.88) [10]	14.09 (0.89) [7]	0.59 (0.29-0.88)	14.95	0.86	0.40
NRXN3	A0A0U1RQC5	start	13.07 (0.82) [5]	12.96 (1.18) [6]	0.6 (0.21-0.99)	12.61	0.67	0.80
NRXN3	A0A0U1RQC5	change	-0.04 (0.05) [5]	-0.04 (0.05) [5]	0.56 (0.13-0.99)	-0.04	0.60	0.80
NRXN3	A0A0U1RQC5	end	13.36 (1.12) [10]	13.46 (1.04) [8]	0.52 (0.23-0.82)	13.08	0.75	0.50
NSG1	A0A0A6YYJ2	end	10.79 (1.06) [7]	10.62 (0.94) [7]	0.55 (0.22-0.88)	10.11	0.43	0.86
NTM	Q9P121-4	end	14.6 (0.55) [11]	13.95 (0.97) [9]	0.75 (0.5-1)	14.12	0.67	0.91
NTM	Q9P121-4	start	13.77 (0.7) [6]	13.57 (1.34) [5]	0.6 (0.21-0.99)	13.74	0.80	0.50
NUCB1	Q02818	start	13.47 (0.69) [10]	12.89 (1.59) [6]	0.58 (0.25-0.91)	13.95	0.83	0.40
NUCB1	Q02818	end	14.14 (0.84) [11]	13.39 (0.21) [9]	0.74 (0.49-0.98)	13.73	1.00	0.64
OAF	Q86UD1	end	14.55 (0.54) [8]	14.58 (0.18) [6]	0.5 (0.17-0.83)	14.25	1.00	0.25
OGN	P20774	end	15.58 (0.86) [12]	15.85 (0.4) [9]	0.6 (0.34-0.86)	15.71	0.67	0.67
OGN	P20774	change	-0.08 (0.13) [11]	-0.11 (0.09) [6]	0.55 (0.25-0.84)	-0.11	0.67	0.64
OGN	P20774	start	14.4 (1.88) [11]	14.05 (1.59) [8]	0.56 (0.28-0.83)	15.21	0.75	0.55
OMD	Q99983	end	13.49 (1.08) [6]	13.06 (0.59) [6]	0.64 (0.24-1)	14.11	1.00	0.50
OMG	P23515	end	15.02 (0.82) [10]	13.87 (1.25) [7]	0.79 (0.55-1)	14.38	0.71	0.80

Protein Name	Uniprot ID	Time	SAH Weaned<br><br>Mean (SD) [N]	SAH Shunted<br><br>Mean (SD) [N]	AUC (95%CI)	Cut-off	Sens.	Spec.
ORM1	P02763	end	19.5 (0.5) [12]	19.76 (0.88) [10]	0.72 (0.47-0.96)	19.67	0.80	0.75
ORM1	P02763	change	-0.02 (0.04) [12]	0 (0.04) [10]	0.63 (0.39-0.88)	0.00	0.50	0.83
ORM1	P02763	start	19.23 (0.35) [12]	19.7 (0.62) [11]	0.77 (0.56-0.97)	19.55	0.64	0.92
ORM2	P19652	end	17.92 (0.66) [12]	18.38 (0.6) [10]	0.74 (0.51-0.97)	18.41	0.70	0.83
ORM2	P19652	start	17.77 (0.53) [12]	17.88 (0.4) [11]	0.58 (0.34-0.83)	17.88	0.64	0.67
ORM2	P19652	change	0 (0.06) [12]	-0.02 (0.02) [10]	0.58 (0.33-0.84)	0.02	1.00	0.33
P4HB	P07237	end	13.71 (1.16) [9]	12.79 (0.6) [9]	0.72 (0.47-0.97)	13.96	1.00	0.44
PAM	P19021	end	14.62 (0.61) [10]	14.18 (0.59) [9]	0.72 (0.47-0.97)	14.65	0.89	0.60
PAM	P19021	start	13.74 (0.78) [7]	13.96 (1.07) [6]	0.5 (0.13-0.87)	14.93	0.33	1.00
PAM	P19021	change	-0.05 (0.05) [6]	-0.03 (0.04) [5]	0.6 (0.19-1)	0.00	0.40	1.00
PAPLN	O95428	end	13.29 (0.61) [7]	13.02 (0.49) [6]	0.64 (0.31-0.97)	12.60	0.33	1.00
PARK7	Q99497	end	14.09 (1.02) [8]	13.63 (0.68) [7]	0.62 (0.32-0.93)	14.84	1.00	0.38
PCDHAC2	Q9Y5I4	end	14.46 (1.2) [5]	14.21 (2.17) [6]	0.6 (0.21-0.99)	13.18	0.50	1.00
PCOLCE	Q15113	end	15.84 (0.96) [12]	16.26 (0.28) [9]	0.67 (0.41-0.92)	16.20	0.78	0.67
PCOLCE	Q15113	change	-0.03 (0.08) [9]	-0.08 (0.09) [5]	0.71 (0.34-1)	-0.12	0.60	1.00
PCOLCE	Q15113	start	15.42 (1.07) [9]	14.65 (1.4) [7]	0.68 (0.36-1)	14.79	0.71	0.78
PCSK1N	Q9UHG2	end	15.28 (0.74) [12]	14.97 (0.39) [9]	0.69 (0.44-0.93)	15.36	0.89	0.58

**Protein Name**	**Uniprot ID**	**Time**	**SAH Weaned**<br><br>Mean (SD) [N]	**SAH Shunted**<br><br>Mean (SD) [N]	**AUC (95%CI)**	**Cut-off**	**Sens.**	**Spec.**
PCSK1N	Q9UHG2	change	-0.11 (0.12) [11]	-0.06 (0.05) [8]	0.58 (0.31-0.85)	-0.11	0.88	0.45
PCSK1N	Q9UHG2	start	14.08 (1.22) [11]	13.87 (1.29) [10]	0.59 (0.32-0.86)	13.83	0.60	0.73
PCSK9	Q8NBP7	start	12.09 (0.81) [5]	12.28 (0.4) [5]	0.56 (0.13-0.99)	11.55	1.00	0.40
PDIA3	P30101	end	14.4 (1.1) [11]	13.5 (0.53) [7]	0.78 (0.55-1)	13.64	0.71	0.73
PDIA6	Q15084	end	14.04 (1.49) [8]	13.2 (0.86) [6]	0.75 (0.47-1)	13.57	0.83	0.75
PEA15	Q15121	end	14.5 (0.82) [9]	14.05 (0.55) [7]	0.7 (0.42-0.98)	14.14	0.71	0.78
PEBP1	P30086	change	-0.01 (0.08) [12]	-0.03 (0.02) [5]	0.63 (0.36-0.91)	0.01	1.00	0.50
PEBP1	P30086	start	16.04 (0.97) [12]	15.04 (0.86) [7]	0.76 (0.53-0.99)	14.84	0.57	0.92
PEBP1	P30086	end	16.05 (0.66) [12]	15.6 (0.67) [9]	0.71 (0.47-0.96)	15.61	0.67	0.83
PEBP4	Q96S96	end	15.46 (0.54) [11]	15.27 (0.5) [9]	0.62 (0.35-0.88)	15.64	0.89	0.36
PEBP4	Q96S96	start	14.36 (0.81) [7]	15.16 (1.3) [5]	0.66 (0.32-1)	14.89	0.60	0.71
PENK	P01210	end	14.43 (1.02) [12]	13.55 (1.12) [9]	0.73 (0.51-0.95)	14.89	1.00	0.42
PENK	P01210	start	13.91 (0.84) [6]	14.47 (0.87) [5]	0.7 (0.31-1)	14.59	0.60	1.00
PEPD	P12955	change	-0.04 (0.04) [6]	-0.04 (0.02) [7]	0.6 (0.24-0.95)	-0.03	0.86	0.50
PEPD	P12955	end	13.33 (0.74) [10]	13.35 (0.55) [9]	0.56 (0.27-0.84)	12.95	0.89	0.40
PEPD	P12955	start	12.57 (0.56) [8]	12.66 (0.6) [9]	0.5 (0.18-0.82)	12.27	0.89	0.38
PF4	P02776	start	15.29 (1.09) [5]	15.28 (0.72) [6]	0.53 (0.14-0.93)	15.28	0.50	0.80

**Protein Name**	**Uniprot ID**	**Time**	**SAH Weaned** Mean (SD) [N]	**SAH Shunted** Mean (SD) [N]	**AUC (95%CI)**	**Cut-off**	**Sens.**	**Spec.**
PFN1	P07737	end	16.29 (0.9) [12]	15.74 (0.56) [9]	0.69 (0.45-0.92)	16.23	0.89	0.50
PFN1	P07737	change	-0.04 (0.06) [12]	0.05 (0.13) [6]	0.69 (0.4-0.98)	0.01	0.67	0.75
PFN1	P07737	start	15.81 (0.48) [12]	16.42 (2.23) [8]	0.55 (0.25-0.85)	16.18	0.50	0.83
PGAM1	P18669	start	14.91 (0.79) [10]	15.12 (1.58) [5]	0.52 (0.12-0.92)	15.57	0.40	0.90
PGAM1	P18669	end	15.15 (0.85) [12]	14.87 (0.45) [9]	0.6 (0.34-0.86)	15.37	0.89	0.50
PGK1	P00558	start	15.5 (0.74) [10]	16.47 (4.6) [7]	0.59 (0.24-0.93)	14.05	0.43	1.00
PGK1	P00558	change	-0.04 (0.08) [10]	0.09 (0.3) [5]	0.58 (0.2-0.96)	-0.05	0.80	0.50
PGK1	P00558	end	15.7 (1.21) [12]	15.26 (0.67) [9]	0.67 (0.42-0.91)	15.76	0.89	0.50
PGLYRP2	Q96PD5	end	14.91 (1.38) [11]	14.38 (0.9) [10]	0.59 (0.33-0.85)	14.12	0.50	0.82
PGLYRP2	Q96PD5	start	15.43 (0.67) [12]	15.71 (0.87) [11]	0.64 (0.39-0.88)	16.06	0.45	0.92
PGLYRP2	Q96PD5	change	0.06 (0.15) [11]	0.06 (0.07) [10]	0.51 (0.24-0.77)	0.00	0.90	0.36
PI16	Q6UXB8	end	13.23 (0.86) [10]	13.25 (1.49) [10]	0.56 (0.29-0.83)	13.85	0.90	0.30
PI16	Q6UXB8	change	-0.01 (0.08) [8]	-0.02 (0.1) [8]	0.53 (0.22-0.84)	-0.02	0.62	0.62
PI16	Q6UXB8	start	13.16 (0.65) [9]	13.06 (0.59) [9]	0.53 (0.24-0.82)	13.96	1.00	0.22
PIK3IP1	Q96FE7-4	end	15.03 (0.58) [10]	14.35 (0.86) [7]	0.77 (0.54-1)	15.25	1.00	0.50
PKM	P14618	end	15.21 (0.74) [12]	14.75 (0.54) [9]	0.7 (0.46-0.94)	14.97	0.78	0.67

Protein Name	Uniprot ID	Time	SAH Weaned<br><br>Mean (SD) [N]	SAH Shunted<br><br>Mean (SD) [N]	AUC (95%CI)	Cut-off	Sens.	Spec.
PLG	P00747	end	16.06 (0.69) [12]	16.33 (0.97) [10]	0.61 (0.36-0.86)	16.21	0.50	0.75
PLG	P00747	start	17.05 (0.7) [12]	17.52 (1.13) [11]	0.64 (0.39-0.88)	17.48	0.64	0.75
PLG	P00747	change	0.09 (0.11) [12]	0.05 (0.1) [10]	0.58 (0.33-0.84)	0.06	0.60	0.67
PLTP	P55058	end	16.11 (1.16) [12]	16.28 (0.6) [10]	0.5 (0.24-0.76)	16.53	0.60	0.67
PLTP	P55058	start	15.04 (1.05) [11]	14.94 (1.34) [11]	0.57 (0.32-0.82)	14.18	0.45	0.82
PLTP	P55058	change	-0.1 (0.14) [11]	-0.06 (0.08) [10]	0.59 (0.33-0.85)	-0.15	0.90	0.36
PLXDC2	Q6UX71	end	14.7 (1.1) [12]	14.32 (0.68) [9]	0.69 (0.45-0.94)	14.36	0.67	0.83
PLXDC2	Q6UX71	start	13.49 (1.1) [10]	13.43 (1.33) [8]	0.56 (0.26-0.87)	13.30	0.62	0.80
PLXDC2	Q6UX71	change	-0.13 (0.15) [10]	-0.06 (0.08) [6]	0.67 (0.38-0.95)	-0.14	1.00	0.40
PMFBP1	G3V1Q7	start	15.52 (0.82) [5]	16.02 (0.77) [6]	0.7 (0.36-1)	15.45	0.83	0.60
PMFBP1	G3V1Q7	end	16.22 (0.42) [8]	16.26 (0.35) [9]	0.53 (0.21-0.84)	16.22	0.67	0.62
PNP	P00491	end	14.08 (0.32) [7]	13.72 (1.39) [6]	0.55 (0.14-0.96)	13.27	0.50	1.00
PODXL2	Q9NZ53	end	12.05 (0.55) [6]	11.95 (1.17) [5]	0.53 (0.13-0.93)	12.55	0.40	0.83
POMGNT1	Q8WZA1	end	13.76 (0.52) [9]	13.76 (1.08) [8]	0.53 (0.21-0.84)	13.29	0.38	0.89
PON1	P27169	end	15.53 (1.06) [12]	15.83 (0.85) [9]	0.67 (0.41-0.92)	15.52	0.78	0.67
PON1	P27169	start	17.36 (1.04) [12]	17.55 (1.35) [10]	0.66 (0.4-0.91)	17.81	0.80	0.67

Protein Name	Uniprot ID	Time	SAH Weaned<br><br>Mean (SD) [N]	SAH Shunted<br><br>Mean (SD) [N]	AUC (95%CI)	Cut-off	Sens.	Spec.
PON1	P27169	change	0.15 (0.16) [12]	0.08 (0.09) [8]	0.68 (0.42-0.94)	0.12	0.88	0.67
PON3	C9JZ99	start	13.1 (1.02) [6]	14.41 (0.48) [6]	0.86 (0.58-1)	13.65	1.00	0.83
PPBP	P02775	start	18.48 (1.04) [11]	18.3 (1.79) [9]	0.53 (0.25-0.8)	18.84	0.56	0.64
PPIA	P62937	change	-0.03 (0.1) [12]	-0.05 (0.06) [9]	0.67 (0.42-0.92)	-0.01	0.89	0.50
PPIA	P62937	end	16.58 (1.12) [12]	16.09 (0.91) [10]	0.69 (0.45-0.94)	16.27	0.80	0.75
PPIA	P62937	start	16.4 (0.74) [12]	14.96 (1.3) [10]	0.83 (0.65-1)	15.39	0.70	0.92
PPIB	P23284	change	-0.05 (0.09) [8]	0.01 (0.05) [6]	0.69 (0.39-0.99)	-0.02	0.83	0.62
PPIB	P23284	start	14.3 (0.57) [8]	14.42 (1.12) [8]	0.53 (0.21-0.85)	15.23	0.38	1.00
PPIC	P45877	end	14.55 (0.53) [8]	14.27 (0.34) [6]	0.71 (0.4-1)	14.38	0.83	0.75
PRCP	P42785	end	14.96 (0.59) [10]	14.6 (0.46) [5]	0.72 (0.44-1)	14.91	0.80	0.70
PRDX1	Q06830	start	16.84 (0.97) [12]	16.16 (3.35) [10]	0.72 (0.45-0.98)	16.17	0.70	0.83
PRDX1	Q06830	change	-0.03 (0.16) [12]	-0.05 (0.2) [9]	0.69 (0.42-0.95)	-0.07	0.78	0.75
PRDX1	Q06830	end	17 (1.7) [12]	17.37 (2.12) [10]	0.54 (0.28-0.8)	16.52	0.50	0.75
PRDX2	P32119	change	-0.1 (0.21) [12]	-0.05 (0.21) [10]	0.51 (0.25-0.77)	-0.02	0.90	0.42
PRDX2	P32119	end	17.81 (2.16) [12]	18.03 (1.57) [10]	0.52 (0.26-0.77)	16.36	0.90	0.33
PRDX2	P32119	start	16.7 (1.48) [12]	16.61 (4.06) [11]	0.63 (0.36-0.9)	15.47	0.64	0.83
PRDX6	P30041	end	15.43 (1.37) [11]	15.1 (1.11) [9]	0.64 (0.36-0.91)	15.50	0.78	0.73

**Protein Name**	**Uniprot ID**	**Time**	**SAH Weaned**<br><br>Mean (SD) [N]	**SAH Shunted**<br><br>Mean (SD) [N]	**AUC (95%CI)**	**Cut-off**	**Sens.**	**Spec.**
PRDX6	P30041	start	16.13 (0.71) [11]	15.61 (2.09) [9]	0.7 (0.41-0.98)	15.39	0.67	0.91
PRDX6	P30041	change	0.05 (0.09) [10]	0.01 (0.14) [7]	0.61 (0.31-0.92)	0.03	0.86	0.60
PRELP	P51888	end	13.42 (1.12) [8]	13.16 (0.81) [8]	0.58 (0.27-0.88)	13.84	0.88	0.38
PRG4	A0A0U1RR20	end	13.38 (0.71) [9]	14.1 (1.18) [9]	0.74 (0.49-0.99)	14.24	0.56	1.00
PRG4	A0A0U1RR20	start	13.85 (0.93) [10]	13.31 (0.59) [10]	0.67 (0.42-0.92)	14.02	1.00	0.40
PRG4	A0A0U1RR20	change	0.06 (0.08) [8]	-0.03 (0.06) [8]	0.81 (0.59-1)	0.05	1.00	0.62
PRNP	A2A2V1	end	13.36 (0.37) [10]	13.26 (0.85) [9]	0.68 (0.38-0.97)	12.92	0.56	1.00
PROC	E7END6	start	13.95 (0.47) [10]	13.94 (0.8) [10]	0.59 (0.32-0.86)	13.93	0.80	0.50
PROC	E7END6	change	0.02 (0.05) [9]	0.01 (0.04) [8]	0.54 (0.24-0.84)	0.03	0.75	0.56
PROC	E7END6	end	13.58 (0.61) [11]	13.57 (0.36) [9]	0.55 (0.28-0.81)	13.91	0.89	0.36
PROCR	Q9UNN8	change	-0.03 (0.08) [10]	-0.02 (0.03) [7]	0.54 (0.25-0.84)	-0.07	1.00	0.40
PROCR	Q9UNN8	start	14 (0.89) [10]	14 (0.48) [9]	0.52 (0.24-0.8)	12.98	1.00	0.20
PROCR	Q9UNN8	end	14.47 (0.63) [12]	14.17 (0.48) [9]	0.67 (0.42-0.91)	14.78	1.00	0.42
PROS1	P07225	end	15.35 (0.52) [12]	15.07 (0.71) [10]	0.71 (0.46-0.96)	15.17	0.90	0.67
PROS1	P07225	change	0.04 (0.07) [12]	0.03 (0.05) [9]	0.61 (0.35-0.88)	0.04	0.67	0.67
PROS1	P07225	start	15.79 (0.37) [12]	15.93 (0.59) [10]	0.58 (0.3-0.85)	16.17	0.50	0.92

**Protein Name**	**Uniprot ID**	**Time**	**SAH Weaned** Mean (SD) [N]	**SAH Shunted** Mean (SD) [N]	**AUC (95%CI)**	**Cut-off**	**Sens.**	**Spec.**
PROZ	P22891	start	14.87 (1.02) [9]	15.33 (0.79) [8]	0.67 (0.38-0.96)	15.24	0.75	0.78
PRSS3	B1AN99	start	18.69 (2.01) [8]	18.6 (1.49) [6]	0.54 (0.21-0.87)	18.76	0.67	0.62
PRSS3	B1AN99	end	19.42 (2.15) [11]	19.76 (1.13) [6]	0.5 (0.2-0.8)	18.56	1.00	0.27
PSMA1	P25786	end	12.93 (0.68) [6]	12.68 (0.5) [5]	0.57 (0.18-0.95)	13.23	1.00	0.33
PSMA6	G3V5Z7	end	13.73 (1.1) [8]	12.38 (0.8) [8]	0.86 (0.64-1)	13.18	0.88	0.88
PTGDS	P41222	end	19.85 (1.07) [12]	19.96 (0.36) [10]	0.52 (0.26-0.77)	20.51	1.00	0.25
PTGDS	P41222	change	-0.12 (0.15) [12]	-0.09 (0.08) [10]	0.52 (0.25-0.78)	-0.03	0.90	0.42
PTGDS	P41222	start	18.14 (2.21) [12]	18.09 (1.52) [11]	0.58 (0.32-0.83)	19.11	0.82	0.50
PTPRD	P23468	end	14.07 (0.48) [9]	13.23 (0.71) [9]	0.85 (0.66-1)	13.59	0.78	0.89
PTPRG	P23470	end	14.13 (0.95) [9]	13.84 (0.59) [8]	0.61 (0.32-0.9)	14.37	0.88	0.44
PTPRG	P23470	start	13.84 (0.6) [7]	14.2 (1.24) [6]	0.6 (0.2-0.99)	13.87	0.67	0.71
PTPRN2	Q92932	end	14.95 (0.69) [8]	15.49 (0.53) [5]	0.7 (0.37-1)	15.47	0.60	0.88
PTPRS	Q13332	end	14.15 (0.55) [11]	13.42 (0.56) [6]	0.82 (0.6-1)	13.93	0.83	0.73
PTPRZ1	P23471	start	13.75 (0.83) [9]	13.81 (1.38) [5]	0.58 (0.21-0.95)	13.67	0.80	0.56
PTPRZ1	P23471	end	14.55 (0.78) [11]	13.87 (0.55) [9]	0.77 (0.55-0.99)	14.72	1.00	0.55
PVALB	B8ZZ19	end	13.78 (0.64) [9]	13.78 (0.54) [7]	0.56 (0.25-0.86)	14.14	0.86	0.44
PZP	P20742	start	14.69 (0.78) [9]	14.76 (0.81) [8]	0.51 (0.21-0.82)	15.01	0.62	0.67

Protein Name	Uniprot ID	Time	SAH Weaned<br><br>Mean (SD) [N]	SAH Shunted<br><br>Mean (SD) [N]	AUC (95%CI)	Cut-off	Sens.	Spec.
PZP	P20742	end	13.62 (0.28) [5]	13.46 (1.86) [8]	0.62 (0.27-0.98)	14.17	0.62	1.00
QDPR	P09417	end	14.18 (0.44) [8]	13.44 (0.93) [6]	0.79 (0.49-1)	13.88	0.83	0.88
QPCT	Q16769	end	13.28 (0.52) [8]	12.8 (0.68) [8]	0.66 (0.36-0.95)	12.75	0.50	0.88
QSOX1	O00391	start	13.97 (0.52) [10]	14 (0.35) [9]	0.53 (0.25-0.81)	13.71	0.89	0.40
QSOX1	O00391	change	-0.03 (0.04) [9]	-0.02 (0.03) [7]	0.56 (0.24-0.87)	-0.05	1.00	0.44
QSOX1	O00391	end	14.35 (0.44) [11]	14.38 (0.38) [9]	0.52 (0.25-0.78)	14.49	0.44	0.73
RARRES2	Q99969	start	14.32 (0.81) [8]	14.38 (1.27) [6]	0.5 (0.14-0.86)	14.72	0.50	0.75
RARRES2	Q99969	end	14.91 (0.85) [11]	14.48 (0.65) [9]	0.68 (0.43-0.93)	14.78	0.78	0.64
RBP4	P02753	end	16.15 (0.59) [12]	16.52 (1.01) [10]	0.6 (0.35-0.85)	17.04	0.30	1.00
RBP4	P02753	change	0 (0.05) [12]	0.02 (0.07) [10]	0.64 (0.39-0.89)	-0.02	0.90	0.50
RBP4	P02753	start	16.04 (0.46) [12]	16.78 (1.03) [11]	0.76 (0.53-0.98)	16.41	0.73	0.83
RGMB	J3KNF6	end	14.02 (0.91) [8]	13.11 (1.43) [5]	0.75 (0.37-1)	13.19	0.80	0.88
RNASE1	P07998	end	12.03 (1.08) [11]	12.32 (0.95) [9]	0.63 (0.36-0.89)	11.93	0.78	0.64
RNASE4	P34096	end	13.36 (0.76) [6]	12.9 (0.64) [5]	0.57 (0.18-0.95)	13.61	1.00	0.33
RNASE6	Q93091	end	14.07 (1.11) [9]	13.55 (0.59) [8]	0.68 (0.41-0.95)	14.35	1.00	0.44
RNASET2	A0A087WZM2	end	14.75 (0.82) [11]	13.66 (0.61) [9]	0.86 (0.69-1)	14.56	1.00	0.64
RNH1	P13489	end	14.73 (1.11) [9]	14.22 (0.93) [6]	0.67 (0.36-0.97)	14.43	0.83	0.67

Protein Name	Uniprot ID	Time	SAH Weaned<br><br>Mean (SD) [N]	SAH Shunted<br><br>Mean (SD) [N]	AUC (95%CI)	Cut-off	Sens.	Spec.
ROBO1	Q9Y6N7	end	13.4 (0.94) [8]	13.16 (0.33) [5]	0.65 (0.32-0.98)	13.65	1.00	0.50
RP1	P56715	end	14.29 (0.42) [6]	14.86 (0.56) [5]	0.83 (0.57-1)	14.99	0.60	1.00
S100A4	P26447	end	14.35 (2.26) [8]	11.27 (2.99) [5]	0.8 (0.53-1)	14.13	0.80	0.75
S100A8	P05109	change	-0.15 (0.21) [11]	-0.02 (0.06) [6]	0.71 (0.46-0.97)	-0.07	0.83	0.64
S100A8	P05109	start	16.29 (1.33) [12]	15.29 (2.69) [8]	0.66 (0.35-0.96)	16.19	0.75	0.67
S100A9	P06702	end	17.42 (1.47) [11]	15.35 (1.82) [9]	0.82 (0.63-1)	17.47	0.89	0.64
S100A9	P06702	change	-0.13 (0.21) [10]	0.07 (0.2) [5]	0.76 (0.5-1)	-0.07	1.00	0.60
S100A9	P06702	start	15.85 (0.74) [11]	17.33 (3.13) [6]	0.61 (0.22-0.99)	17.38	0.50	1.00
S100B	P04271	end	13.78 (1.38) [8]	13.7 (1.03) [6]	0.54 (0.21-0.87)	15.27	1.00	0.25
S100B	P04271	change	0.26 (0.13) [7]	0.09 (0.05) [5]	0.89 (0.65-1)	0.16	1.00	0.86
SCG2	P13521	start	12.83 (0.51) [9]	12.75 (0.93) [9]	0.56 (0.27-0.84)	13.18	0.78	0.44
SCG2	P13521	end	13.43 (0.76) [12]	13.11 (0.84) [9]	0.64 (0.38-0.9)	13.47	0.78	0.58
SCG2	P13521	change	-0.06 (0.05) [9]	-0.03 (0.02) [7]	0.71 (0.43-1)	-0.05	0.86	0.67
SCG3	Q8WXD2	start	13.97 (1.06) [9]	14.07 (1.55) [7]	0.52 (0.19-0.85)	14.05	0.57	0.67
SCG3	Q8WXD2	change	-0.11 (0.07) [9]	-0.07 (0.07) [5]	0.67 (0.33-1)	-0.02	0.40	1.00
SCG3	Q8WXD2	end	15.23 (1.02) [12]	15.06 (0.69) [9]	0.59 (0.33-0.85)	15.56	0.89	0.42
SCG5	P05408-2	change	-0.1 (0.17) [9]	-0.07 (0.09) [6]	0.5 (0.18-0.82)	-0.01	0.83	0.33
SCG5	P05408-2	start	14.84 (0.84) [9]	14.35 (1.33) [7]	0.62 (0.31-0.93)	15.23	0.86	0.44

Protein Name	Uniprot ID	Time	SAH Weaned<br><br>Mean (SD) [N]	SAH Shunted<br><br>Mean (SD) [N]	AUC (95%CI)	Cut-off	Sens.	Spec.
SCG5	P05408-2	end	15.7 (0.89) [12]	15.51 (1.07) [10]	0.68 (0.44-0.93)	15.70	0.80	0.67
SCRG1	O75711	end	17.16 (0.68) [11]	16.87 (0.4) [9]	0.66 (0.4-0.92)	17.26	0.89	0.55
SCRG1	O75711	start	16.09 (1.66) [6]	15.74 (1.45) [6]	0.56 (0.19-0.92)	17.90	1.00	0.17
SDF4	Q9BRK5	end	13.25 (1.04) [9]	13.08 (0.46) [7]	0.62 (0.32-0.91)	13.33	0.86	0.56
SELENBP1	Q13228	end	15.28 (0.89) [12]	14.76 (0.88) [9]	0.66 (0.4-0.91)	15.29	0.78	0.58
SELENOP	A0A182DWH7	start	15.66 (0.42) [8]	15.97 (0.34) [9]	0.67 (0.38-0.95)	15.72	0.78	0.62
SELL	P14151	start	15.87 (0.58) [10]	16.05 (0.6) [9]	0.57 (0.29-0.84)	15.38	0.89	0.30
SELL	P14151	change	0.01 (0.04) [9]	0.04 (0.02) [7]	0.68 (0.4-0.97)	0.00	1.00	0.56
SELL	P14151	end	15.6 (0.5) [11]	15.09 (0.44) [9]	0.8 (0.59-1)	15.61	1.00	0.55
SEMA4B	J3KNP4	end	13.31 (0.32) [6]	13.11 (0.82) [8]	0.52 (0.19-0.85)	13.22	0.62	0.67
SEMA7A	O75326	end	13.37 (1.1) [8]	12.7 (1.02) [7]	0.71 (0.43-0.99)	12.97	0.71	0.75
SERPINA1	P01009	change	0.01 (0.05) [12]	0.01 (0.06) [10]	0.51 (0.25-0.77)	0.00	0.80	0.50
SERPINA1	P01009	start	18.59 (0.6) [12]	18.9 (0.65) [11]	0.6 (0.36-0.84)	18.88	0.45	0.75
SERPINA1	P01009	end	18.45 (0.32) [12]	18.79 (0.89) [10]	0.68 (0.41-0.94)	18.95	0.40	1.00
SERPINA10	G3V2W1	start	13.41 (0.95) [10]	14.25 (0.86) [8]	0.72 (0.47-0.98)	14.11	0.62	0.80
SERPINA10	G3V2W1	change	0.04 (0.09) [7]	0.11 (0.06) [6]	0.76 (0.46-1)	0.03	1.00	0.57
SERPINA10	G3V2W1	end	12.61 (0.96) [9]	12.11 (1.08) [8]	0.65 (0.37-0.93)	11.19	0.38	1.00

**Protein Name**	**Uniprot ID**	**Time**	**SAH Weaned** Mean (SD) [N]	**SAH Shunted** Mean (SD) [N]	**AUC (95%CI)**	**Cut-off**	**Sens.**	**Spec.**
SERPINA3	P01011	start	17.43 (0.6) [12]	17.64 (0.85) [11]	0.54 (0.29-0.79)	16.85	1.00	0.17
SERPINA3	P01011	end	17.73 (0.58) [12]	18.14 (0.96) [10]	0.62 (0.37-0.88)	17.86	0.80	0.50
SERPINA3	P01011	change	-0.02 (0.06) [12]	-0.02 (0.06) [10]	0.54 (0.28-0.8)	-0.05	0.80	0.50
SERPINA4	P29622	end	15 (0.46) [12]	14.77 (0.37) [9]	0.69 (0.45-0.92)	14.82	0.67	0.75
SERPINA4	P29622	change	0.05 (0.06) [12]	0.04 (0.02) [8]	0.53 (0.26-0.8)	0.02	0.88	0.42
SERPINA4	P29622	start	15.62 (0.5) [12]	15.75 (0.4) [10]	0.55 (0.3-0.8)	15.60	0.80	0.42
SERPINA5	P05154	change	0.01 (0.06) [11]	0.02 (0.03) [8]	0.62 (0.36-0.89)	0.00	0.88	0.55
SERPINA5	P05154	end	14.43 (0.82) [11]	14.11 (0.42) [9]	0.69 (0.44-0.94)	14.11	0.67	0.73
SERPINA5	P05154	start	14.5 (0.41) [11]	14.67 (0.63) [10]	0.65 (0.4-0.91)	14.76	0.60	0.82
SERPINA6	P08185	change	0.03 (0.04) [12]	0.04 (0.07) [10]	0.56 (0.3-0.82)	0.03	0.60	0.67
SERPINA6	P08185	start	15.81 (0.55) [12]	16.18 (0.81) [11]	0.66 (0.42-0.9)	16.23	0.55	0.83
SERPINA6	P08185	end	15.41 (0.48) [12]	15.41 (0.83) [10]	0.58 (0.33-0.84)	15.10	0.50	0.75
SERPINA7	P05543	change	0.02 (0.07) [12]	0 (0.04) [9]	0.51 (0.24-0.78)	0.04	1.00	0.25
SERPINA7	P05543	end	14.84 (0.45) [12]	14.88 (0.73) [10]	0.51 (0.24-0.77)	14.42	0.30	0.92
SERPINA7	P05543	start	14.91 (0.61) [12]	14.89 (0.37) [10]	0.58 (0.32-0.83)	15.23	0.90	0.42
SERPINB1	P30740	end	14.39 (1.14) [10]	13.54 (0.34) [8]	0.75 (0.51-0.99)	14.23	1.00	0.50

**Protein Name**	**Uniprot ID**	**Time**	**SAH Weaned** Mean (SD) [N]	**SAH Shunted** Mean (SD) [N]	**AUC (95%CI)**	**Cut-off**	**Sens.**	**Spec.**
SERPINC1	P01008	end	16.86 (0.4) [12]	17.23 (0.82) [10]	0.62 (0.38-0.87)	17.31	0.40	0.92
SERPINC1	P01008	change	0 (0.04) [12]	0 (0.07) [10]	0.55 (0.29-0.81)	-0.02	0.70	0.58
SERPINC1	P01008	start	16.81 (0.39) [12]	17.17 (0.79) [11]	0.64 (0.4-0.88)	16.84	0.64	0.67
SERPIND1	P05546	change	0.07 (0.09) [12]	0.04 (0.05) [10]	0.51 (0.25-0.77)	0.08	0.90	0.33
SERPIND1	P05546	start	16.07 (0.54) [12]	16.26 (0.66) [11]	0.63 (0.38-0.88)	16.37	0.64	0.67
SERPIND1	P05546	end	15.3 (0.44) [12]	15.28 (0.63) [10]	0.59 (0.33-0.85)	15.33	0.80	0.58
SERPINF1	P36955	change	-0.07 (0.08) [12]	-0.01 (0.07) [10]	0.72 (0.5-0.94)	-0.11	1.00	0.42
SERPINF1	P36955	start	16.66 (1.06) [12]	16.5 (1.01) [11]	0.57 (0.32-0.82)	16.88	0.73	0.58
SERPINF1	P36955	end	17.6 (0.94) [12]	16.86 (0.4) [10]	0.79 (0.59-0.99)	17.65	1.00	0.50
SERPINF2	P08697	change	0.04 (0.05) [12]	0.02 (0.05) [10]	0.63 (0.39-0.88)	0.05	0.90	0.42
SERPINF2	P08697	start	16.67 (0.34) [12]	16.89 (0.76) [11]	0.63 (0.38-0.88)	16.97	0.55	0.92
SERPINF2	P08697	end	16.15 (0.3) [12]	16.39 (0.59) [10]	0.63 (0.38-0.88)	16.08	0.70	0.67
SERPING1	P05155	end	16.73 (0.3) [12]	16.77 (0.27) [10]	0.56 (0.3-0.81)	16.57	0.90	0.33
SERPING1	P05155	start	16.91 (0.3) [12]	17.13 (0.32) [11]	0.72 (0.49-0.95)	17.11	0.73	0.75
SERPING1	P05155	change	0.02 (0.03) [12]	0.02 (0.02) [10]	0.53 (0.27-0.8)	0.00	0.90	0.42
SERPINI1	Q99574	start	13.23 (0.61) [6]	13.36 (1.37) [5]	0.53 (0.11-0.96)	14.02	0.40	1.00

Protein Name	Uniprot ID	Time	SAH Weaned<br><br>Mean (SD) [N]	SAH Shunted<br><br>Mean (SD) [N]	AUC (95%CI)	Cut-off	Sens.	Spec.
SEZ6	Q53EL9	end	13.53 (0.66) [8]	13.15 (0.43) [8]	0.75 (0.48-1)	13.63	1.00	0.50
SEZ6L	B0QYH4	end	13.51 (0.8) [9]	13.31 (0.89) [9]	0.6 (0.32-0.89)	13.42	0.67	0.67
SEZ6L2	A0A087WYL5	end	14.53 (0.62) [11]	14.73 (0.6) [8]	0.62 (0.35-0.9)	14.92	0.50	0.82
SH3BGRL	O75368	end	14.3 (1.17) [9]	13.1 (0.42) [9]	0.85 (0.63-1)	13.80	1.00	0.67
SH3BGRL3	Q5T123	end	15.06 (1.28) [10]	14.05 (0.58) [7]	0.8 (0.57-1)	14.72	1.00	0.70
SHBG	I3L145	end	13.45 (0.45) [8]	13.88 (0.53) [9]	0.82 (0.59-1)	13.35	1.00	0.62
SHBG	I3L145	start	14.62 (1.29) [10]	14.95 (1.2) [9]	0.57 (0.29-0.85)	15.41	0.56	0.70
SHBG	I3L145	change	0.11 (0.06) [6]	0.04 (0.07) [7]	0.74 (0.45-1)	0.02	0.43	1.00
SHISA6	Q6ZSJ9	end	13.8 (0.47) [6]	13.49 (0.46) [7]	0.67 (0.32-1)	13.89	0.86	0.67
SIRPA	P78324	start	13.68 (0.64) [6]	13.97 (0.76) [5]	0.63 (0.25-1)	13.90	0.60	0.83
SIRPA	P78324	end	14.37 (0.76) [10]	13.63 (0.76) [8]	0.8 (0.59-1)	14.35	0.88	0.70
SKP1	E5RJR5	end	14.36 (0.74) [9]	13.93 (0.87) [9]	0.65 (0.39-0.92)	14.48	0.78	0.56
SKP1	E5RJR5	change	-0.02 (0.05) [7]	0 (0.05) [5]	0.57 (0.2-0.94)	-0.01	0.80	0.57
SKP1	E5RJR5	start	14.33 (0.59) [9]	13.81 (0.67) [7]	0.73 (0.46-1)	13.85	0.57	0.89
SLC3A2	F5GZS6	end	12.66 (0.37) [9]	13.06 (0.36) [7]	0.76 (0.51-1)	13.02	0.57	0.89
SLC4A1	P02730	end	13.96 (1.02) [7]	13.87 (1.02) [7]	0.55 (0.21-0.89)	14.51	0.86	0.43
SNCA	E7EPV7	end	13.02 (1.64) [8]	13.08 (1.12) [5]	0.52 (0.18-0.87)	11.54	1.00	0.25
SOD1	P00441	change	-0.09 (0.08) [11]	0.04 (0.17) [5]	0.75 (0.49-1)	-0.08	1.00	0.45

**Protein Name**	**Uniprot ID**	**Time**	**SAH Weaned** Mean (SD) [N]	**SAH Shunted** Mean (SD) [N]	**AUC (95%CI)**	**Cut-off**	**Sens.**	**Spec.**
SOD1	P00441	end	16.09 (0.5) [12]	15.8 (0.34) [9]	0.66 (0.41-0.9)	16.22	1.00	0.33
SOD1	P00441	start	15.09 (0.75) [11]	16.11 (2.71) [7]	0.58 (0.26-0.91)	16.03	0.43	1.00
SOD2	P04179	end	14.04 (0.94) [12]	13.67 (0.4) [9]	0.59 (0.32-0.86)	14.14	1.00	0.50
SOD3	P08294	end	15.7 (1.11) [12]	15.03 (0.37) [9]	0.73 (0.5-0.97)	15.23	0.78	0.75
SOD3	P08294	change	-0.08 (0.11) [12]	-0.03 (0.05) [8]	0.65 (0.39-0.9)	-0.09	0.88	0.50
SOD3	P08294	start	14.64 (1.32) [12]	14.57 (0.87) [10]	0.57 (0.31-0.82)	14.95	0.80	0.50
SORCS3	Q9UPU3	end	12.79 (0.76) [7]	12.26 (0.77) [8]	0.71 (0.43-1)	12.66	0.75	0.71
SPARC	P09486	change	-0.05 (0.09) [12]	-0.05 (0.08) [9]	0.5 (0.24-0.76)	-0.09	0.78	0.42
SPARC	P09486	start	14.67 (0.6) [12]	14.67 (1) [10]	0.52 (0.25-0.79)	14.27	0.40	0.83
SPARC	P09486	end	15.54 (1.2) [12]	15.84 (0.7) [10]	0.52 (0.25-0.78)	16.06	0.80	0.50
SPARCL1	Q14515	start	13.61 (0.64) [11]	13.37 (1.08) [10]	0.55 (0.29-0.82)	13.04	0.40	0.82
SPARCL1	Q14515	change	-0.07 (0.08) [11]	-0.05 (0.06) [8]	0.64 (0.37-0.91)	-0.05	0.75	0.64
SPARCL1	Q14515	end	14.43 (0.76) [12]	14.4 (0.47) [9]	0.56 (0.3-0.83)	14.71	0.89	0.42
SPOCK1	Q08629	end	13.18 (0.71) [8]	12.63 (0.94) [7]	0.73 (0.41-1)	13.02	0.86	0.75
SPOCK3	Q9BQ16	end	14.38 (0.95) [9]	13.52 (0.57) [7]	0.78 (0.53-1)	13.76	0.71	0.89
SPON1	Q9HCB6	end	13.99 (0.63) [8]	13.05 (0.65) [6]	0.88 (0.68-1)	13.05	0.67	1.00
SPP1	P10451	end	16.09 (1.37) [12]	16.37 (0.67) [9]	0.54 (0.27-0.8)	16.53	0.56	0.67

**Protein Name**	**Uniprot ID**	**Time**	**SAH Weaned** Mean (SD) [N]	**SAH Shunted** Mean (SD) [N]	**AUC (95%CI)**	**Cut-off**	**Sens.**	**Spec.**
SPP1	P10451	start	14.18 (1.94) [12]	14.94 (2.76) [11]	0.52 (0.26-0.77)	16.10	0.27	0.92
SPP1	P10451	change	-0.14 (0.15) [12]	-0.05 (0.15) [9]	0.68 (0.43-0.92)	-0.14	0.78	0.67
STAB1	Q9NY15	end	12.74 (0.69) [6]	12.42 (0.45) [6]	0.67 (0.32-1)	12.73	0.83	0.50
SULF2	Q8IWU5	end	14.4 (0.62) [11]	14.12 (0.39) [9]	0.66 (0.4-0.91)	14.73	1.00	0.36
SUSD5	O60279	end	12.44 (0.54) [8]	12.64 (0.6) [9]	0.56 (0.26-0.85)	12.98	0.33	0.88
SYNE3	G3V533	start	15.48 (0.74) [7]	15.95 (0.95) [8]	0.68 (0.38-0.98)	15.70	0.75	0.71
SYNE3	G3V533	end	14.85 (0.89) [7]	14.98 (0.29) [7]	0.53 (0.15-0.91)	15.37	1.00	0.43
SYT2	Q8N9I0	start	18.21 (1.06) [7]	18.65 (0.77) [5]	0.6 (0.24-0.96)	17.94	0.80	0.57
SYT2	Q8N9I0	end	17.5 (0.98) [8]	18.66 (0.63) [7]	0.84 (0.63-1)	18.02	0.86	0.75
SAA1	P0DJI8	start	16.09 (1.07) [10]	16.53 (1.1) [9]	0.66 (0.4-0.91)	15.63	0.89	0.50
SAA1	P0DJI8	end	15.42 (1.17) [9]	15.54 (1.71) [6]	0.56 (0.2-0.91)	16.92	0.33	1.00
SAA4	P35542	end	15.45 (0.68) [12]	15.81 (0.42) [9]	0.75 (0.52-0.98)	15.80	0.67	0.83
SAA4	P35542	change	0.1 (0.13) [12]	0.05 (0.07) [8]	0.57 (0.3-0.85)	0.10	1.00	0.42
SAA4	P35542	start	16.58 (0.9) [12]	16.82 (1.3) [10]	0.66 (0.4-0.92)	16.81	0.70	0.75
TAGLN	Q01995	end	14.39 (0.53) [11]	14.96 (0.62) [9]	0.82 (0.6-1)	14.82	0.78	0.91
TAGLN2	P37802	start	14.54 (1.13) [9]	14.85 (0.95) [7]	0.56 (0.23-0.88)	15.80	0.29	1.00
TALDO1	P37837	end	15.37 (1.09) [11]	14.27 (0.91) [9]	0.76 (0.53-0.99)	15.16	1.00	0.64

**Protein Name**	**Uniprot ID**	**Time**	**SAH Weaned** Mean (SD) [N]	**SAH Shunted** Mean (SD) [N]	**AUC (95%CI)**	**Cut-off**	**Sens.**	**Spec.**
TCN2	B5MBX2	end	15.19 (0.58) [7]	15.07 (2) [7]	0.63 (0.3-0.97)	14.09	0.43	1.00
TF	P02787	end	17.71 (0.46) [12]	17.89 (0.99) [10]	0.51 (0.25-0.77)	17.94	0.90	0.33
TF	P02787	start	17.88 (0.39) [12]	18.04 (0.56) [11]	0.61 (0.36-0.86)	17.77	0.82	0.58
TF	P02787	change	0.02 (0.06) [12]	0 (0.07) [10]	0.54 (0.28-0.8)	0.00	0.90	0.42
TFRC	G3V0E5	start	12 (0.67) [8]	12.8 (0.43) [5]	0.85 (0.62-1)	12.26	1.00	0.75
TFRC	G3V0E5	end	11.21 (0.61) [5]	10.78 (0.83) [6]	0.67 (0.31-1)	10.33	0.50	1.00
TGFBI	Q15582	end	15.39 (0.95) [12]	15.75 (1.37) [10]	0.51 (0.25-0.77)	15.34	0.80	0.50
TGFBI	Q15582	start	14.52 (0.93) [12]	15 (2.15) [11]	0.54 (0.28-0.8)	13.93	0.82	0.50
TGFBI	Q15582	change	-0.07 (0.09) [12]	-0.03 (0.13) [10]	0.61 (0.36-0.86)	-0.07	0.70	0.67
TGOLN2	F8W8W7	end	11.29 (1.08) [10]	12.16 (1.61) [9]	0.61 (0.33-0.89)	12.00	0.56	0.80
THBS1	P07996	end	14.36 (0.58) [8]	14.52 (0.69) [7]	0.61 (0.28-0.94)	14.90	0.57	0.88
THBS1	P07996	change	0.04 (0.05) [7]	0.04 (0.04) [5]	0.54 (0.19-0.9)	0.08	1.00	0.29
THBS1	P07996	start	15.18 (0.5) [10]	15.55 (0.6) [9]	0.7 (0.44-0.96)	15.76	0.56	0.90
THBS2	P35442	start	15.16 (1.12) [8]	14.98 (0.86) [5]	0.52 (0.17-0.88)	16.47	1.00	0.25
THBS2	P35442	end	13.69 (0.44) [7]	13.88 (0.15) [7]	0.53 (0.17-0.89)	13.58	1.00	0.43
THY1	E9PIM6	end	16.34 (0.92) [12]	15.76 (0.58) [9]	0.79 (0.58-1)	15.98	0.78	0.75
THY1	E9PIM6	change	-0.1 (0.08) [10]	-0.04 (0.07) [7]	0.7 (0.44-0.96)	-0.11	1.00	0.40

Protein Name	Uniprot ID	Time	SAH Weaned<br><br>Mean (SD) [N]	SAH Shunted<br><br>Mean (SD) [N]	AUC (95%CI)	Cut-off	Sens.	Spec.
THY1	E9PIM6	start	15.08 (0.87) [10]	14.76 (1.47) [9]	0.67 (0.39-0.95)	14.25	0.56	0.90
TIMP1	P01033	change	-0.08 (0.23) [9]	-0.09 (0.15) [8]	0.51 (0.21-0.82)	-0.22	0.88	0.44
TIMP1	P01033	start	18.1 (2.16) [9]	16.01 (2.33) [9]	0.74 (0.48-1)	17.82	0.89	0.67
TIMP1	P01033	end	18.54 (2.07) [12]	18.51 (1.39) [10]	0.55 (0.29-0.81)	19.92	1.00	0.42
TIMP2	P16035	end	15.72 (0.69) [10]	15.45 (0.75) [7]	0.6 (0.31-0.89)	16.15	1.00	0.30
TKT	P29401	end	15.16 (1.15) [11]	14.24 (1.03) [9]	0.72 (0.48-0.96)	14.10	0.56	0.91
TNC	P24821	end	13.32 (0.86) [6]	12.61 (0.37) [5]	0.8 (0.51-1)	13.15	1.00	0.67
TNXB	A0A140TA41	end	13.32 (0.51) [11]	13.54 (0.52) [9]	0.61 (0.34-0.87)	13.70	0.44	0.82
TNXB	A0A140TA41	start	13.16 (0.21) [5]	13.33 (1) [6]	0.67 (0.25-1)	13.55	0.67	1.00
TPI1	P60174	end	14.9 (1.12) [12]	14.03 (1.01) [9]	0.73 (0.49-0.97)	14.47	0.78	0.75
TPI1	P60174	change	-0.05 (0.14) [12]	0.02 (0.13) [7]	0.56 (0.27-0.85)	0.07	0.29	0.92
TPI1	P60174	start	14.5 (0.86) [12]	14.61 (1.85) [9]	0.55 (0.24-0.85)	13.72	0.44	0.92
TPM4	P67936	start	15.23 (0.7) [9]	14.86 (0.69) [6]	0.63 (0.32-0.93)	15.26	0.83	0.56
TPP1	O14773	start	14.45 (0.45) [8]	14.42 (0.52) [5]	0.52 (0.15-0.9)	14.85	0.40	0.88
TPP2	P29144	end	14.44 (1.25) [10]	15.06 (0.77) [9]	0.71 (0.46-0.97)	14.59	0.78	0.70
TPP2	P29144	start	14.97 (1.16) [10]	15.2 (1.1) [10]	0.5 (0.23-0.77)	16.72	0.20	1.00
TPP2	P29144	change	0.04 (0.07) [8]	0 (0.06) [8]	0.67 (0.38-0.96)	0.09	1.00	0.38

Protein Name	Uniprot ID	Time	**SAH Weaned**<br><br>Mean (SD) [N]	**SAH Shunted**<br><br>Mean (SD) [N]	AUC (95%CI)	Cut-off	Sens.	Spec.
TRBV7-2	A0A1B0GXF2	end	14.73 (0.53) [7]	14.88 (0.49) [9]	0.56 (0.25-0.86)	14.66	0.67	0.57
TREM2	Q9NZC2	end	14.07 (0.71) [9]	14.43 (0.51) [9]	0.63 (0.35-0.91)	13.75	1.00	0.33
TREM2	Q9NZC2	start	13.57 (0.89) [5]	13.13 (0.81) [7]	0.66 (0.29-1)	13.75	0.86	0.60
TREM2	Q9NZC2	change	-0.04 (0.08) [5]	-0.05 (0.05) [5]	0.56 (0.15-0.97)	-0.01	0.80	0.40
TTR	P02766	end	19.32 (0.92) [12]	19.04 (0.74) [10]	0.57 (0.31-0.83)	18.96	0.60	0.75
TTR	P02766	change	-0.05 (0.07) [12]	-0.02 (0.06) [10]	0.6 (0.35-0.85)	-0.11	1.00	0.33
TTR	P02766	start	18.62 (0.68) [12]	18.44 (0.75) [11]	0.62 (0.36-0.88)	18.06	0.55	0.92
TUBA1B	P68363	change	0.17 (0.19) [11]	0.06 (0.11) [7]	0.71 (0.46-0.97)	0.17	1.00	0.64
TUBA1B	P68363	end	15.23 (1.49) [11]	14.45 (1.65) [9]	0.64 (0.37-0.91)	13.59	0.44	0.91
TUBB4B	P68371	end	15.18 (1.36) [9]	15 (1.13) [6]	0.57 (0.25-0.89)	15.37	0.83	0.56
TUBB4B	P68371	change	0.14 (0.2) [9]	0.01 (0.1) [5]	0.73 (0.44-1)	0.07	1.00	0.67
TUBB4B	P68371	start	16.76 (1.33) [12]	15.35 (0.96) [10]	0.84 (0.66-1)	16.40	1.00	0.75
TXN	P10599	end	17.11 (0.86) [12]	17.08 (0.48) [9]	0.57 (0.32-0.83)	17.93	1.00	0.25
TXN	P10599	start	16.16 (0.91) [9]	16.45 (1.32) [5]	0.56 (0.18-0.93)	17.03	0.40	0.89
UBC	F5H265	end	16.33 (0.76) [12]	15.8 (0.33) [9]	0.71 (0.47-0.96)	16.21	0.89	0.67
UBC	F5H265	start	15.21 (0.72) [10]	14.53 (1.36) [9]	0.69 (0.41-0.96)	14.21	0.56	0.90
UBC	F5H265	change	-0.1 (0.1) [10]	-0.06 (0.07) [7]	0.6 (0.3-0.9)	-0.15	1.00	0.30

Protein Name	Uniprot ID	Time	SAH Weaned<br><br>Mean (SD) [N]	SAH Shunted<br><br>Mean (SD) [N]	AUC (95%CI)	Cut-off	Sens.	Spec.
VASN	Q6EMK4	end	14.43 (0.24) [11]	14.32 (0.3) [9]	0.6 (0.33-0.86)	14.22	0.33	0.91
VASN	Q6EMK4	change	-0.02 (0.02) [8]	0 (0.02) [6]	0.77 (0.5-1)	-0.02	1.00	0.62
VASN	Q6EMK4	start	14.12 (0.33) [9]	14.44 (0.55) [7]	0.76 (0.47-1)	14.22	0.86	0.78
VCAM1	P19320	end	14.01 (0.76) [10]	13.91 (0.27) [8]	0.55 (0.26-0.84)	14.27	1.00	0.30
VCAM1	P19320	start	13.46 (0.63) [6]	13.27 (1.03) [6]	0.56 (0.18-0.93)	12.41	0.33	1.00
VCAN	P13611	end	14.53 (0.65) [11]	14.24 (0.74) [9]	0.71 (0.45-0.97)	14.34	0.78	0.73
VCAN	P13611	start	14.35 (0.66) [7]	14.31 (1.36) [5]	0.54 (0.09-0.99)	14.62	0.60	0.86
VGF	O15240	start	13.15 (0.83) [8]	13.5 (0.96) [7]	0.57 (0.26-0.89)	12.46	1.00	0.25
VGF	O15240	end	14.08 (1.02) [12]	13.69 (1.1) [9]	0.59 (0.33-0.86)	14.17	0.78	0.58
VGF	O15240	change	-0.08 (0.09) [8]	-0.04 (0.04) [5]	0.62 (0.28-0.97)	-0.04	0.60	0.75
VIM	P08670	end	16.84 (1.79) [12]	16.09 (1.53) [9]	0.7 (0.45-0.95)	16.30	0.78	0.75
VIM	P08670	change	-0.13 (0.21) [11]	-0.12 (0.15) [6]	0.5 (0.2-0.8)	-0.12	0.83	0.45
VIM	P08670	start	15.44 (0.82) [11]	14.31 (1.85) [8]	0.69 (0.39-1)	14.33	0.62	1.00
VIP	P01282	change	0 (0.04) [11]	0.01 (0.03) [8]	0.56 (0.28-0.83)	-0.01	0.88	0.45
VIP	P01282	start	18.2 (0.47) [11]	18.52 (0.71) [10]	0.73 (0.48-0.97)	18.31	0.80	0.73
VIP	P01282	end	18.17 (0.42) [12]	18.41 (0.71) [9]	0.69 (0.42-0.97)	18.40	0.78	0.75
VSIG4	Q9Y279	end	15.98 (1.27) [10]	15.6 (0.58) [8]	0.7 (0.43-0.97)	16.49	1.00	0.50

Protein Name	Uniprot ID	Time	SAH Weaned<br>Mean (SD) [N]	SAH Shunted<br>Mean (SD) [N]	AUC (95%CI)	Cut-off	Sens.	Spec.
VSTM2A	B5MCX6	end	13.95 (0.86) [9]	13.51 (1.73) [7]	0.54 (0.2-0.88)	13.44	0.57	0.78
VSTM2B	A6NLU5	end	12.65 (0.67) [7]	13.02 (0.85) [7]	0.69 (0.38-1)	12.88	0.71	0.71
VTN	P04004	end	17.19 (0.63) [12]	17.28 (0.4) [9]	0.57 (0.32-0.83)	16.76	1.00	0.25
VTN	P04004	change	0.06 (0.08) [12]	0.05 (0.03) [8]	0.54 (0.27-0.81)	0.04	0.88	0.50
VTN	P04004	start	17.89 (0.8) [12]	18.46 (0.77) [10]	0.72 (0.48-0.95)	18.68	0.60	0.92
VWF	P04275	start	14.11 (0.96) [8]	14.72 (1.01) [9]	0.61 (0.32-0.9)	15.36	0.33	1.00
WDR1	O75083	end	14.72 (1.35) [9]	13.46 (1.19) [5]	0.78 (0.47-1)	13.83	0.80	0.78
WFIKKN2	C9J6G4	start	14.82 (0.92) [9]	14.68 (1.15) [6]	0.59 (0.25-0.93)	14.29	0.67	0.67
YWHAB	P31946	end	13.5 (1.38) [10]	12.75 (0.68) [7]	0.66 (0.38-0.94)	13.15	0.86	0.60
YWHAE	P62258	end	14.74 (1.35) [12]	14.51 (0.58) [8]	0.64 (0.37-0.9)	14.88	0.88	0.58
YWHAE	P62258	start	15.03 (0.9) [9]	16.18 (3.49) [5]	0.6 (0.23-0.97)	15.04	0.80	0.56
YWHAG	P61981	end	13.12 (1.23) [10]	13.58 (1.03) [8]	0.6 (0.32-0.88)	14.01	0.50	0.80
YWHAQ	P27348	start	13.94 (0.98) [9]	13.36 (0.6) [5]	0.73 (0.45-1)	13.48	0.80	0.78
YWHAQ	P27348	end	14.31 (1.37) [11]	13.6 (0.83) [8]	0.64 (0.37-0.9)	14.48	0.88	0.55
YWHAZ	P63104	change	-0.05 (0.12) [12]	-0.07 (0.06) [6]	0.62 (0.35-0.9)	0.02	1.00	0.33
YWHAZ	P63104	end	15.37 (1.18) [12]	15.04 (0.48) [9]	0.61 (0.35-0.87)	15.78	1.00	0.50
YWHAZ	P63104	start	14.8 (0.78) [12]	13.68 (1.37) [8]	0.76 (0.5-1)	13.45	0.62	1.00

**Protein Name**	**Uniprot ID**	**Time**	**SAH Weaned**<br><br>Mean (SD) [N]	**SAH Shunted**<br><br>Mean (SD) [N]	**AUC (95%CI)**	**Cut-off**	**Sens.**	**Spec.**
ZNF511-PRAP1	H7BY64	end	14.39 (0.79) [10]	13.6 (0.28) [7]	0.81 (0.58-1)	14.11	1.00	0.70

SAH: subarachnoid hemorrhage; SD: standard deviation; N: number of patients; AUC: area under the curve; CI: confidence interval; sens: sensitivity; spec: specificity.

Supplementary Table 6: Proteins not identified as possible predictors of functional outcome in SAH patients.

Protein Name	Uniprot ID	Time	mRS 0-2 Mean (SD) [N]	mRS 3-6 Mean (SD) [N]	AUC (95%CI)	Cut-off	Sens.	Spec.
A1BG	P04217-2	start	17.64 (0.81) [12]	17.73 (0.52) [10]	0.51 (0.25-0.76)	17.02	0.90	0.33
A1BG	P04217-2	end	17.1 (0.55) [11]	16.98 (0.27) [10]	0.56 (0.3-0.83)	17.44	1.00	0.27
A1BG	P04217-2	change	0.04 (0.09) [11]	0.04 (0.03) [10]	0.6 (0.35-0.85)	-0.01	1.00	0.27
A2M	P01023	end	17.32 (0.72) [11]	17.18 (0.42) [10]	0.5 (0.23-0.77)	17.26	0.60	0.64
A2M	P01023	start	17.88 (0.92) [12]	18.52 (0.69) [10]	0.72 (0.49-0.94)	18.01	0.90	0.58
A2M	P01023	change	0.04 (0.12) [11]	0.07 (0.05) [10]	0.72 (0.48-0.96)	0.05	0.90	0.64
ABHD14B	Q96IU4	end	13.46 (0.96) [10]	13.62 (0.76) [8]	0.64 (0.34-0.93)	12.97	1.00	0.50
ABHD14B	Q96IU4	start	13.06 (1.16) [6]	13.02 (1.05) [7]	0.52 (0.17-0.88)	12.54	0.43	0.83
ACAT2	Q9BWD1	end	12.1 (1.2) [5]	11.99 (0.63) [7]	0.51 (0.09-0.94)	11.29	1.00	0.40
ACSBG2	Q5FVE4	change	-0.08 (0.16) [8]	-0.05 (0.04) [7]	0.52 (0.2-0.84)	0.03	1.00	0.25
ACSBG2	Q5FVE4	end	20.92 (0.87) [10]	21.01 (0.5) [10]	0.51 (0.22-0.8)	20.21	1.00	0.30
ACSBG2	Q5FVE4	start	20.27 (0.79) [10]	20.28 (0.57) [7]	0.51 (0.22-0.81)	20.95	1.00	0.30
ACTA2	P62736	end	17.28 (0.83) [10]	16.99 (1.35) [10]	0.51 (0.24-0.78)	16.91	0.70	0.50
ACTA2	P62736	change	-0.07 (0.1) [8]	-0.06 (0.1) [8]	0.55 (0.24-0.86)	-0.03	0.75	0.50
ACTA2	P62736	start	16.55 (0.62) [10]	16.11 (1.06) [8]	0.64 (0.35-0.92)	15.91	0.50	0.90

Protein Name	Uniprot ID	Time	mRS 0-2 Mean (SD) [N]	mRS 3-6 Mean (SD) [N]	AUC (95%CI)	Cut-off	Sens.	Spec.
ACTB	P60709	change	-0.06 (0.09) [11]	-0.04 (0.13) [10]	0.56 (0.3-0.83)	-0.06	0.70	0.55
ACTB	P60709	end	17.59 (0.94) [11]	17.23 (1.39) [10]	0.55 (0.28-0.81)	16.36	0.20	1.00
ACTB	P60709	start	16.61 (0.99) [12]	16.61 (1.87) [10]	0.58 (0.33-0.84)	17.46	0.90	0.33
ACTBL2	Q562R1	end	19.42 (0.8) [10]	19.25 (1.51) [10]	0.51 (0.24-0.78)	18.29	0.20	1.00
ACTBL2	Q562R1	change	-0.08 (0.11) [10]	-0.07 (0.12) [9]	0.52 (0.23-0.81)	-0.08	0.56	0.70
ACTBL2	Q562R1	start	18.42 (1.12) [12]	17.99 (1.32) [9]	0.62 (0.36-0.88)	17.90	0.56	0.75
ADA2	B4E3Q4	end	13.58 (0.58) [7]	13.43 (0.62) [8]	0.59 (0.27-0.91)	13.19	0.50	0.86
ADAM29	Q9UKF5	end	14.83 (1.1) [6]	15.45 (0.6) [9]	0.74 (0.42-1)	15.40	0.78	0.83
ADAMTS1	Q9UHI8	end	14.67 (0.31) [6]	14.56 (0.46) [6]	0.56 (0.15-0.96)	14.57	0.67	0.83
ADGRB2	A2A3C1	end	14.49 (0.82) [9]	14.37 (0.82) [6]	0.56 (0.23-0.88)	15.48	1.00	0.22
ADGRL1	O94910	end	14.14 (0.47) [7]	13.27 (0.54) [5]	0.86 (0.57-1)	13.38	0.80	1.00
ADIPOQ	Q15848	end	11.99 (0.55) [8]	11.82 (0.78) [8]	0.58 (0.27-0.88)	11.92	0.62	0.62
ADIPOQ	Q15848	start	14.07 (1.18) [5]	13.83 (1.31) [8]	0.5 (0.12-0.88)	12.93	0.88	0.40
AEBP1	Q8IUX7	end	13.24 (0.6) [7]	13.61 (0.62) [9]	0.65 (0.36-0.94)	14.03	0.44	1.00
AFM	P43652	end	15.58 (1.29) [11]	15.3 (0.37) [10]	0.55 (0.29-0.82)	15.44	0.80	0.55
AFM	P43652	change	0.02 (0.09) [11]	0.02 (0.04) [9]	0.59 (0.31-0.86)	0.01	0.56	0.73
AFM	P43652	start	15.82 (0.42) [12]	15.68 (0.56) [9]	0.59 (0.32-0.86)	15.78	0.67	0.67

**Protein Name**	**Uniprot ID**	**Time**	**mRS 0-2** Mean (SD) [N]	**mRS 3-6** Mean (SD) [N]	**AUC (95%CI)**	**Cut-off**	**Sens.**	**Spec.**
AGA	P20933	end	14.04 (0.61) [6]	12.66 (1.6) [7]	0.88 (0.69-1)	13.09	0.71	1.00
AGRN	O00468-6	end	13.85 (0.71) [8]	13.65 (0.95) [10]	0.66 (0.38-0.95)	13.79	0.70	0.88
AGT	P01019	change	-0.02 (0.03) [11]	0 (0.06) [10]	0.54 (0.27-0.81)	0.02	0.30	1.00
AGT	P01019	start	16.25 (0.46) [12]	16.47 (0.83) [10]	0.54 (0.28-0.81)	16.33	0.70	0.58
AGT	P01019	end	16.41 (0.33) [11]	16.52 (0.35) [10]	0.67 (0.42-0.93)	16.37	0.90	0.55
AHSG	P02765	change	0.03 (0.16) [11]	0.1 (0.14) [10]	0.66 (0.42-0.91)	0.07	0.60	0.82
AHSG	P02765	start	17.6 (0.73) [12]	18.76 (2.43) [10]	0.71 (0.47-0.94)	18.00	0.70	0.75
AHSG	P02765	end	17.39 (1.96) [11]	17.08 (0.43) [10]	0.59 (0.32-0.86)	16.76	0.90	0.55
ALB	P02768	start	19.83 (0.54) [12]	19.99 (0.75) [10]	0.51 (0.25-0.77)	19.60	0.80	0.42
ALB	P02768	end	19.42 (0.71) [11]	19.3 (0.33) [10]	0.55 (0.28-0.81)	19.33	0.70	0.55
ALB	P02768	change	0.04 (0.1) [11]	0.04 (0.04) [10]	0.57 (0.31-0.84)	0.04	0.60	0.73
ALCAM	Q13740	end	14.45 (0.41) [9]	14.55 (0.62) [10]	0.53 (0.26-0.81)	14.98	0.20	1.00
ALDOA	P04075	change	-0.09 (0.1) [11]	-0.03 (0.17) [9]	0.55 (0.25-0.84)	-0.02	0.44	0.91
ALDOA	P04075	start	14.5 (1.02) [12]	15.42 (2.91) [9]	0.55 (0.28-0.82)	13.49	1.00	0.25
ALDOA	P04075	end	15.79 (1.05) [11]	15.71 (0.81) [10]	0.59 (0.32-0.86)	15.67	0.70	0.64
ALDOC	P09972	start	14.17 (1.04) [7]	13.79 (1.2) [7]	0.59 (0.26-0.92)	15.09	1.00	0.29

Protein Name	Uniprot ID	Time	mRS 0-2 Mean (SD) [N]	mRS 3-6 Mean (SD) [N]	AUC (95%CI)	Cut-off	Sens.	Spec.
ALDOC	P09972	end	14.68 (0.66) [10]	14.53 (0.42) [10]	0.58 (0.3-0.86)	15.09	1.00	0.40
ALDOC	P09972	change	-0.01 (0.08) [5]	-0.05 (0.07) [7]	0.6 (0.21-0.99)	0.04	1.00	0.40
AMBP	P02760	end	16.14 (0.55) [11]	15.83 (0.53) [10]	0.64 (0.38-0.89)	15.78	0.60	0.73
AMBP	P02760	change	0.07 (0.09) [11]	0.06 (0.05) [10]	0.54 (0.27-0.8)	0.10	0.30	0.91
AMBP	P02760	start	17.08 (0.7) [12]	16.85 (0.97) [10]	0.6 (0.34-0.86)	17.21	0.70	0.67
ANXA2	P07355	end	12.7 (2.03) [8]	13.28 (2.47) [8]	0.61 (0.31-0.91)	14.99	0.38	1.00
ANXA5	P08758	end	14.49 (0.73) [10]	14.97 (1.76) [8]	0.54 (0.24-0.84)	14.16	0.75	0.50
APCS	P02743	start	17.31 (1.76) [12]	18 (1.52) [9]	0.6 (0.34-0.86)	16.90	0.89	0.42
APCS	P02743	end	15.16 (1.39) [10]	15.09 (1.26) [10]	0.51 (0.24-0.78)	14.30	0.40	0.80
APCS	P02743	change	0.17 (0.22) [10]	0.17 (0.13) [9]	0.61 (0.34-0.88)	0.11	0.89	0.40
APLP1	B7Z4G8	end	15.59 (0.69) [10]	15.58 (0.81) [10]	0.53 (0.25-0.81)	15.25	0.50	0.80
APLP1	B7Z4G8	start	14.57 (0.99) [12]	14.42 (1.6) [7]	0.55 (0.24-0.85)	14.71	0.71	0.50
APLP1	B7Z4G8	change	-0.06 (0.06) [10]	-0.07 (0.08) [7]	0.51 (0.19-0.84)	-0.06	0.57	0.70
APLP2	Q06481	start	14.31 (0.44) [7]	13.92 (1.59) [6]	0.52 (0.1-0.95)	13.56	0.50	1.00
APLP2	Q06481	end	14.19 (0.69) [10]	14.29 (1.03) [9]	0.56 (0.27-0.84)	14.08	0.67	0.60
APLP2	Q06481	change	-0.01 (0.03) [6]	-0.05 (0.11) [6]	0.56 (0.17-0.95)	-0.03	0.67	0.67

Protein Name	Uniprot ID	Time	mRS 0-2 Mean (SD) [N]	mRS 3-6 Mean (SD) [N]	AUC (95%CI)	Cut-off	Sens.	Spec.
APOA1	P02647	start	19.81 (1.21) [12]	20.22 (0.94) [10]	0.54 (0.28-0.8)	19.29	0.90	0.42
APOA1	P02647	change	0.07 (0.17) [11]	0.11 (0.08) [10]	0.71 (0.47-0.95)	0.13	0.60	0.82
APOA1	P02647	end	18.83 (1.34) [11]	18.34 (0.53) [10]	0.59 (0.33-0.86)	18.62	0.90	0.45
APOA2	P02652	start	18.89 (0.95) [12]	19.04 (0.92) [10]	0.53 (0.28-0.79)	18.17	0.90	0.33
APOA2	P02652	end	17.61 (1.27) [11]	17.52 (0.93) [10]	0.53 (0.25-0.8)	17.62	0.80	0.55
APOA2	P02652	change	0.1 (0.16) [11]	0.09 (0.09) [10]	0.55 (0.28-0.81)	0.06	0.80	0.45
APOA4	P06727	start	17.26 (0.84) [12]	17.57 (1.01) [9]	0.61 (0.35-0.88)	17.31	0.78	0.50
APOA4	P06727	change	0.06 (0.14) [11]	0.09 (0.05) [9]	0.72 (0.48-0.96)	0.07	0.78	0.73
APOA4	P06727	end	16.5 (1.25) [11]	16.01 (0.5) [10]	0.55 (0.29-0.82)	16.84	1.00	0.27
APOB	P04114	end	15.98 (1.43) [10]	15.97 (1.34) [10]	0.56 (0.29-0.83)	15.29	0.80	0.40
APOB	P04114	start	18.18 (1.85) [12]	19.45 (2.36) [10]	0.67 (0.42-0.91)	18.50	0.90	0.50
APOB	P04114	change	0.19 (0.26) [10]	0.2 (0.17) [10]	0.67 (0.41-0.93)	0.19	0.60	0.80
APOC1	K7ERI9	end	15.91 (1.24) [10]	15.86 (0.6) [10]	0.6 (0.32-0.88)	15.43	0.90	0.50
APOC1	K7ERI9	start	17.85 (1.74) [12]	18.4 (1.72) [9]	0.55 (0.28-0.81)	17.03	0.89	0.42
APOC1	K7ERI9	change	0.15 (0.23) [10]	0.14 (0.1) [9]	0.56 (0.27-0.84)	0.14	0.78	0.60
APOC3	B0YIW2	end	16.98 (1.77) [10]	17.51 (1.34) [10]	0.71 (0.45-0.97)	16.46	0.90	0.70

Protein Name	Uniprot ID	Time	**mRS 0-2** Mean (SD) [N]	**mRS 3-6** Mean (SD) [N]	AUC (95%CI)	Cut-off	Sens.	Spec.
APOC3	B0YIW2	start	19.23 (2.35) [12]	20.33 (2.07) [9]	0.59 (0.33-0.85)	18.62	0.89	0.42
APOC3	B0YIW2	change	0.17 (0.3) [10]	0.17 (0.16) [9]	0.52 (0.24-0.8)	0.08	0.89	0.40
APOC4-APOC2	A0A024R0T9	start	17.28 (2.19) [12]	18.08 (2.02) [9]	0.51 (0.24-0.78)	16.58	0.89	0.42
APOC4-APOC2	A0A024R0T9	change	0.16 (0.25) [10]	0.15 (0.14) [9]	0.52 (0.24-0.8)	0.07	0.89	0.40
APOC4-APOC2	A0A024R0T9	end	15.18 (1.6) [10]	15.46 (0.95) [10]	0.66 (0.4-0.92)	14.78	0.80	0.60
APOD	C9JF17	start	16.83 (0.82) [12]	16.14 (0.69) [9]	0.74 (0.52-0.96)	17.03	1.00	0.50
APOD	C9JF17	change	-0.05 (0.1) [11]	-0.07 (0.07) [9]	0.63 (0.36-0.89)	-0.02	0.89	0.55
APOD	C9JF17	end	17.7 (1.68) [11]	17.25 (0.65) [10]	0.51 (0.24-0.78)	17.71	0.40	0.82
APOE	P02649	start	16.74 (0.61) [12]	16.37 (0.9) [10]	0.7 (0.45-0.95)	16.42	0.80	0.75
APOE	P02649	change	-0.06 (0.05) [11]	-0.07 (0.07) [10]	0.55 (0.29-0.82)	-0.12	0.40	0.91
APOE	P02649	end	17.76 (0.71) [11]	17.54 (0.37) [10]	0.62 (0.36-0.87)	18.09	1.00	0.36
APOF	Q13790	start	16.28 (0.67) [6]	15.81 (0.91) [8]	0.71 (0.4-1)	16.23	0.88	0.67
APOH	P02749	change	0.06 (0.06) [10]	0.02 (0.02) [9]	0.68 (0.42-0.94)	0.06	1.00	0.50
APOH	P02749	end	15.47 (0.69) [10]	15.9 (0.53) [10]	0.66 (0.41-0.91)	15.14	1.00	0.30
APOH	P02749	start	16.52 (0.71) [12]	16.4 (0.77) [9]	0.63 (0.37-0.89)	16.78	0.89	0.50
APOL1	O14791	change	0.12 (0.2) [10]	0.13 (0.13) [9]	0.62 (0.35-0.9)	0.14	0.56	0.80

Protein Name	Uniprot ID	Time	mRS 0-2 Mean (SD) [N]	mRS 3-6 Mean (SD) [N]	AUC (95%CI)	Cut-off	Sens.	Spec.
APOL1	O14791	start	15.59 (1.66) [12]	16.55 (1.56) [9]	0.66 (0.4-0.91)	15.71	0.89	0.50
APOL1	O14791	end	14.31 (1.42) [10]	14.3 (1.34) [10]	0.54 (0.27-0.81)	14.84	0.40	0.80
APOM	O95445	change	0.13 (0.18) [9]	0.13 (0.13) [8]	0.6 (0.3-0.9)	0.16	0.50	0.89
APOM	O95445	start	17.48 (1.48) [12]	17.92 (1.25) [9]	0.55 (0.28-0.81)	17.15	0.89	0.42
APOM	O95445	end	16.06 (1.09) [9]	15.42 (1.1) [9]	0.63 (0.35-0.91)	15.36	0.67	0.78
APP	P05067	change	-0.02 (0.05) [9]	-0.05 (0.07) [8]	0.62 (0.34-0.91)	0.01	1.00	0.33
APP	P05067	end	13.78 (0.86) [10]	13.98 (0.83) [10]	0.54 (0.27-0.81)	13.98	0.50	0.70
APP	P05067	start	13.53 (0.81) [11]	13.23 (1.22) [8]	0.62 (0.33-0.92)	12.94	0.62	0.82
ARPC4-TTLL3	A0A0A6YYG9	start	16.94 (0.51) [12]	16.59 (0.57) [9]	0.68 (0.44-0.92)	17.03	0.89	0.42
ARPC4-TTLL3	A0A0A6YYG9	change	0.03 (0.04) [10]	0.01 (0.03) [9]	0.63 (0.37-0.9)	0.02	0.67	0.70
ARPC4-TTLL3	A0A0A6YYG9	end	16.38 (0.47) [10]	16.32 (0.23) [10]	0.56 (0.27-0.85)	16.80	1.00	0.30
ART3	E7ESB3	end	14 (0.45) [7]	13.81 (0.63) [6]	0.55 (0.18-0.92)	13.39	0.33	1.00
ATP6AP1	Q15904	end	14.28 (0.91) [10]	14.1 (0.73) [10]	0.61 (0.34-0.88)	13.77	0.40	0.90
ATP6AP1	Q15904	change	-0.06 (0.09) [8]	-0.05 (0.04) [6]	0.56 (0.23-0.9)	-0.04	0.67	0.62
ATP6AP1	Q15904	start	13.95 (0.83) [10]	13.41 (1.14) [6]	0.68 (0.35-1)	12.96	0.50	0.90
ATRN	O75882	end	13.91 (0.61) [8]	14.02 (0.67) [6]	0.52 (0.17-0.87)	14.02	0.67	0.62
ATRN	O75882	change	0.03 (0.04) [7]	0.04 (0.06) [6]	0.5 (0.13-0.87)	0.09	0.33	1.00

**Protein Name**	**Uniprot ID**	**Time**	**mRS 0-2** Mean (SD) [N]	**mRS 3-6** Mean (SD) [N]	**AUC (95%CI)**	**Cut-off**	**Sens.**	**Spec.**
ATRN	O75882	start	14.53 (0.6) [10]	14.79 (0.4) [9]	0.6 (0.33-0.87)	14.11	1.00	0.30
AXL	P30530	end	14.57 (0.75) [5]	14.59 (0.45) [5]	0.56 (0.13-0.99)	14.16	1.00	0.40
AZGP1	P25311	end	16.88 (0.61) [11]	17.21 (0.79) [10]	0.65 (0.39-0.9)	16.86	0.80	0.55
AZGP1	P25311	start	17.03 (0.51) [12]	16.95 (0.64) [9]	0.54 (0.25-0.82)	16.51	0.44	0.92
AZGP1	P25311	change	0.01 (0.05) [11]	-0.02 (0.04) [9]	0.69 (0.44-0.94)	0.01	0.89	0.55
B2M	P61769	change	-0.06 (0.12) [11]	-0.14 (0.16) [10]	0.6 (0.33-0.87)	-0.20	0.40	1.00
B2M	P61769	end	16.92 (1.24) [11]	16.94 (0.57) [10]	0.58 (0.32-0.84)	17.24	0.80	0.55
B2M	P61769	start	15.88 (1.48) [12]	14.66 (2.48) [10]	0.63 (0.38-0.88)	16.21	0.80	0.58
B4GALT1	P15291	end	14.74 (0.44) [7]	14.2 (0.63) [6]	0.76 (0.49-1)	14.93	1.00	0.43
B4GAT1	O43505	end	16.26 (2.21) [11]	15.83 (0.83) [10]	0.51 (0.25-0.77)	14.94	0.90	0.27
B4GAT1	O43505	change	-0.09 (0.14) [10]	-0.07 (0.09) [6]	0.52 (0.2-0.84)	-0.04	0.83	0.40
B4GAT1	O43505	start	14.82 (1.24) [11]	14.81 (1.57) [6]	0.55 (0.22-0.87)	14.44	0.67	0.64
BASP1	P80723	end	12.06 (0.89) [9]	12.23 (1.4) [9]	0.52 (0.22-0.82)	13.38	0.33	1.00
BCAN	Q96GW7	change	0 (0.1) [9]	-0.02 (0.07) [5]	0.51 (0.16-0.87)	-0.12	0.20	1.00
BCAN	Q96GW7	end	14.81 (1.09) [10]	14.36 (0.83) [10]	0.69 (0.43-0.95)	14.35	0.60	0.90
BCAN	Q96GW7	start	14.72 (1.44) [11]	14.56 (0.97) [5]	0.51 (0.21-0.81)	14.86	0.80	0.45

Protein Name	Uniprot ID	Time	mRS 0-2 Mean (SD) [N]	mRS 3-6 Mean (SD) [N]	AUC (95%CI)	Cut-off	Sens.	Spec.
BCHE	P06276	start	14.62 (1.09) [5]	14.25 (1.88) [5]	0.52 (0.11-0.93)	15.38	0.80	0.40
BGN	P21810	end	13.39 (0.95) [8]	13.96 (0.89) [7]	0.62 (0.31-0.94)	12.72	1.00	0.38
BLVRB	P30043	end	15.98 (2.06) [11]	16 (1.09) [9]	0.52 (0.23-0.8)	17.63	1.00	0.36
BPGM	P07738	end	15.42 (1.39) [6]	15.28 (0.65) [8]	0.56 (0.17-0.95)	15.89	0.88	0.50
BTD	P43251	change	0.01 (0.02) [10]	0.01 (0.04) [9]	0.56 (0.25-0.86)	0.01	0.67	0.70
BTD	P43251	start	15.52 (0.36) [12]	15.56 (0.48) [9]	0.56 (0.29-0.82)	15.67	0.78	0.42
BTD	P43251	end	15.34 (0.38) [10]	15.38 (0.49) [10]	0.52 (0.25-0.79)	15.31	0.60	0.60
C16orf89	A0A0A0MT71	end	13.93 (0.45) [9]	13.75 (0.47) [9]	0.65 (0.37-0.94)	14.16	0.89	0.56
C1QA	P02745	start	15.46 (0.42) [12]	15.23 (0.59) [9]	0.68 (0.41-0.94)	15.21	0.67	0.75
C1QA	P02745	change	-0.02 (0.04) [11]	0 (0.06) [9]	0.54 (0.24-0.83)	-0.02	0.67	0.73
C1QA	P02745	end	15.73 (0.7) [11]	15.38 (0.62) [10]	0.64 (0.38-0.89)	15.79	0.80	0.64
C1QB	D6R934	end	16.5 (1.24) [11]	16.07 (0.82) [10]	0.65 (0.39-0.9)	16.65	0.90	0.55
C1QB	D6R934	start	15.78 (0.77) [12]	15.68 (0.46) [9]	0.56 (0.28-0.83)	16.14	0.89	0.50
C1QB	D6R934	change	-0.05 (0.08) [11]	-0.02 (0.06) [9]	0.63 (0.36-0.89)	-0.02	0.56	0.82
C1QC	P02747	end	17.83 (0.73) [10]	17.64 (0.73) [10]	0.61 (0.34-0.88)	17.75	0.60	0.80
C1QC	P02747	change	-0.04 (0.11) [10]	-0.01 (0.07) [9]	0.5 (0.2-0.8)	0.00	0.44	0.80

**Protein Name**	**Uniprot ID**	**Time**	**mRS 0-2** Mean (SD) [N]	**mRS 3-6** Mean (SD) [N]	**AUC (95%CI)**	**Cut-off**	**Sens.**	**Spec.**
C1QC	P02747	start	17.65 (0.47) [12]	17.48 (0.64) [9]	0.65 (0.37-0.92)	17.34	0.56	0.83
C1R	B4DPQ0	end	16.06 (0.47) [11]	16.01 (0.48) [10]	0.5 (0.23-0.77)	15.77	0.70	0.45
C1R	B4DPQ0	change	-0.01 (0.06) [11]	0 (0.04) [9]	0.52 (0.23-0.8)	0.01	0.44	0.82
C1R	B4DPQ0	start	16.11 (0.34) [12]	15.86 (0.41) [9]	0.66 (0.4-0.91)	15.75	0.44	0.92
C1RL	Q9NZP8	change	0.05 (0.02) [7]	0.06 (0.06) [7]	0.53 (0.18-0.88)	0.08	0.43	0.86
C1RL	Q9NZP8	start	14.99 (0.72) [10]	14.84 (1.03) [9]	0.51 (0.2-0.82)	15.55	0.44	0.90
C1RL	Q9NZP8	end	14.13 (0.66) [9]	13.96 (0.47) [8]	0.71 (0.43-0.99)	14.26	0.88	0.67
C1S	P09871	start	16.49 (0.29) [12]	16.16 (0.45) [9]	0.73 (0.5-0.96)	16.25	0.56	0.92
C1S	P09871	end	16.57 (0.47) [11]	16.31 (0.42) [10]	0.65 (0.4-0.91)	16.71	1.00	0.45
C1S	P09871	change	-0.02 (0.06) [11]	-0.01 (0.03) [9]	0.52 (0.24-0.79)	-0.02	0.89	0.36
C2	P06681	change	0 (0.03) [10]	0 (0.05) [9]	0.54 (0.27-0.82)	0.00	0.56	0.60
C2	P06681	start	15.5 (0.44) [12]	15.52 (0.58) [9]	0.55 (0.27-0.83)	15.67	0.56	0.75
C2	P06681	end	15.49 (0.43) [10]	15.46 (0.56) [10]	0.52 (0.24-0.8)	15.75	0.80	0.40
C2orf40	B8ZZE5	start	14.77 (1.25) [9]	14.53 (1.45) [6]	0.54 (0.21-0.87)	15.88	1.00	0.22
C2orf40	B8ZZE5	end	15.25 (0.9) [9]	15.58 (1.2) [10]	0.52 (0.24-0.8)	16.32	0.30	1.00
C2orf40	B8ZZE5	change	-0.03 (0.05) [7]	-0.08 (0.13) [6]	0.6 (0.2-0.99)	-0.05	0.67	0.71

Protein Name	Uniprot ID	Time	mRS 0-2<br>Mean (SD) [N]	mRS 3-6<br>Mean (SD) [N]	AUC (95%CI)	Cut-off	Sens.	Spec.
C3	P01024	change	0.01 (0.07) [11]	0.04 (0.05) [10]	0.59 (0.33-0.85)	-0.01	1.00	0.27
C3	P01024	start	17.71 (0.48) [12]	18.18 (0.88) [10]	0.68 (0.44-0.91)	17.81	0.80	0.58
C3	P01024	end	17.66 (0.74) [11]	17.57 (0.32) [10]	0.55 (0.28-0.81)	17.55	0.70	0.64
C4A	A0A0G2JPR0	start	15.81 (0.86) [12]	15.59 (0.81) [9]	0.62 (0.35-0.89)	15.62	0.56	0.83
C4A	A0A0G2JPR0	end	15.92 (0.72) [11]	15.78 (0.77) [10]	0.53 (0.27-0.79)	16.59	1.00	0.18
C4A	A0A0G2JPR0	change	-0.01 (0.04) [11]	-0.01 (0.08) [9]	0.61 (0.33-0.88)	-0.05	0.33	1.00
C4B	P0C0L5	start	16.89 (0.24) [12]	16.96 (0.36) [10]	0.58 (0.3-0.85)	17.08	0.50	0.83
C4B	P0C0L5	end	17.08 (0.38) [11]	16.98 (0.41) [10]	0.54 (0.27-0.8)	17.51	1.00	0.18
C4B	P0C0L5	change	-0.01 (0.02) [11]	0 (0.03) [10]	0.64 (0.38-0.89)	-0.01	0.70	0.73
C4BPA	P04003	start	16.12 (1.71) [12]	15.91 (2.42) [9]	0.52 (0.25-0.79)	15.67	0.56	0.67
C4BPA	P04003	change	0.18 (0.22) [9]	0.12 (0.16) [8]	0.5 (0.19-0.81)	0.14	0.62	0.67
C4BPA	P04003	end	14.04 (1.28) [9]	13.54 (1.07) [9]	0.64 (0.36-0.93)	14.07	0.78	0.67
C5	P01031	start	15.66 (0.68) [12]	15.88 (0.8) [9]	0.64 (0.38-0.9)	15.55	0.89	0.42
C5	P01031	change	0.07 (0.09) [10]	0.06 (0.06) [9]	0.61 (0.33-0.89)	0.05	0.67	0.70
C5	P01031	end	14.84 (0.49) [10]	14.87 (0.38) [10]	0.55 (0.27-0.83)	14.83	0.70	0.60
C6	P13671	change	0.05 (0.06) [10]	0.08 (0.07) [9]	0.67 (0.4-0.93)	0.05	0.89	0.60

Protein Name	Uniprot ID	Time	mRS 0-2 Mean (SD) [N]	mRS 3-6 Mean (SD) [N]	AUC (95%CI)	Cut-off	Sens.	Spec.
C6	P13671	end	15.48 (0.52) [10]	15 (0.51) [10]	0.71 (0.47-0.95)	15.57	1.00	0.40
C6	P13671	start	16.28 (0.84) [12]	16.45 (0.69) [9]	0.51 (0.24-0.77)	15.97	0.89	0.42
C7	P10643	end	16.71 (0.8) [11]	16.22 (0.48) [10]	0.76 (0.53-1)	16.78	1.00	0.64
C7	P10643	start	16.2 (0.6) [12]	15.44 (0.39) [9]	0.83 (0.65-1)	15.99	1.00	0.67
C7	P10643	change	-0.05 (0.11) [11]	-0.04 (0.03) [9]	0.63 (0.35-0.9)	-0.02	0.89	0.64
C8A	P07357	start	15.84 (0.63) [12]	16.23 (0.84) [10]	0.7 (0.46-0.94)	15.97	0.80	0.58
C8A	P07357	end	15.4 (0.72) [11]	15.22 (0.4) [10]	0.55 (0.28-0.82)	15.59	0.90	0.36
C8A	P07357	change	0.04 (0.11) [11]	0.06 (0.05) [10]	0.7 (0.46-0.94)	0.05	0.60	0.82
C8B	F5GY80	start	14.67 (0.53) [12]	14.81 (0.69) [9]	0.57 (0.3-0.85)	14.91	0.56	0.67
C8B	F5GY80	change	0.05 (0.08) [10]	0.03 (0.04) [8]	0.52 (0.22-0.83)	0.02	0.38	0.90
C8B	F5GY80	end	14.01 (0.44) [10]	14.07 (0.25) [9]	0.58 (0.29-0.87)	13.77	0.89	0.50
C8G	P07360	start	14.83 (0.29) [8]	14.48 (0.64) [7]	0.66 (0.32-1)	14.38	0.57	1.00
C8G	P07360	change	0.04 (0.03) [5]	0.05 (0.05) [6]	0.5 (0.11-0.89)	0.08	0.33	1.00
C8G	P07360	end	13.93 (0.48) [7]	13.96 (0.55) [8]	0.52 (0.2-0.84)	13.86	0.50	0.71
C9	P02748	start	16.09 (0.53) [12]	16.04 (0.49) [9]	0.56 (0.29-0.82)	16.43	0.89	0.42
C9	P02748	end	15.85 (0.96) [11]	15.73 (0.51) [10]	0.54 (0.27-0.8)	15.84	0.60	0.64
C9	P02748	change	0.02 (0.06) [11]	0.02 (0.04) [9]	0.52 (0.24-0.79)	0.03	0.56	0.73

Protein Name	Uniprot ID	Time	mRS 0-2 Mean (SD) [N]	mRS 3-6 Mean (SD) [N]	AUC (95%CI)	Cut-off	Sens.	Spec.
CA1	P00915	start	16.68 (2.23) [11]	17.63 (3.3) [7]	0.53 (0.23-0.83)	14.59	1.00	0.27
CA1	P00915	change	-0.11 (0.16) [10]	-0.06 (0.21) [7]	0.53 (0.19-0.86)	-0.18	0.43	0.90
CA1	P00915	end	18.19 (2.3) [11]	18.63 (0.79) [9]	0.61 (0.34-0.88)	18.16	0.89	0.55
CA2	P00918	end	15.8 (3.09) [9]	14.93 (1.33) [9]	0.53 (0.23-0.83)	16.95	1.00	0.33
CA2	P00918	start	14.76 (0.94) [10]	16.51 (4.32) [8]	0.56 (0.26-0.86)	15.19	0.50	0.80
CA2	P00918	change	-0.09 (0.17) [8]	0.1 (0.28) [8]	0.7 (0.43-0.98)	-0.16	1.00	0.38
CACNA2D1	P54289	end	13.65 (0.71) [9]	13.35 (1.08) [9]	0.62 (0.34-0.9)	13.44	0.56	0.78
CADM1	A0A087X0T8	end	15.13 (0.65) [8]	14.79 (0.77) [10]	0.64 (0.36-0.91)	15.28	0.80	0.50
CADM2	Q8N3J6	end	13.79 (0.58) [6]	13.73 (0.86) [6]	0.58 (0.21-0.96)	13.46	0.50	0.83
CADM3	Q8N126	end	15.19 (0.38) [9]	15.18 (1.31) [10]	0.67 (0.38-0.95)	14.99	0.70	0.89
CADM4	Q8NFZ8	end	15.46 (0.37) [9]	15.43 (0.59) [10]	0.56 (0.27-0.84)	15.00	0.30	1.00
CALR	P27797	end	13.58 (0.88) [10]	13.72 (0.66) [10]	0.58 (0.31-0.85)	13.55	0.50	0.80
CALR	P27797	start	13.23 (1.01) [5]	13.31 (0.8) [5]	0.52 (0.11-0.93)	12.75	0.40	0.80
CAP1	Q01518	end	14.59 (1.36) [5]	15.75 (1.32) [5]	0.76 (0.41-1)	14.34	1.00	0.60
CAPG	P40121	end	14.5 (0.67) [6]	14.19 (0.92) [6]	0.64 (0.28-1)	14.56	0.83	0.50
CARTPT	Q16568	start	13.16 (0.56) [7]	12.81 (1.16) [5]	0.54 (0.13-0.96)	11.85	0.40	1.00
CARTPT	Q16568	end	13.32 (0.61) [7]	12.98 (0.59) [8]	0.66 (0.36-0.97)	13.65	1.00	0.43

Protein Name	Uniprot ID	Time	mRS 0-2<br>Mean (SD) [N]	mRS 3-6<br>Mean (SD) [N]	AUC (95%CI)	Cut-off	Sens.	Spec.
CASP14	P31944	start	15.41 (1.21) [9]	14.49 (0.81) [5]	0.76 (0.48-1)	15.50	1.00	0.67
CASP14	P31944	end	14.3 (1.93) [9]	14.25 (0.93) [7]	0.63 (0.34-0.93)	15.22	1.00	0.44
CAT	P04040	end	15.17 (1.89) [11]	15.51 (0.48) [10]	0.55 (0.24-0.85)	14.94	1.00	0.55
CAT	P04040	change	-0.04 (0.07) [6]	-0.06 (0.19) [7]	0.69 (0.36-1)	-0.11	0.57	1.00
CAT	P04040	start	15.16 (1.69) [6]	14.53 (3.18) [7]	0.71 (0.4-1)	13.47	0.57	1.00
CBR1	P16152	change	-0.01 (0.09) [5]	0.06 (0.1) [7]	0.74 (0.44-1)	0.06	0.57	1.00
CBR1	P16152	end	14.46 (1.06) [9]	14.14 (0.64) [9]	0.63 (0.34-0.92)	14.23	0.78	0.67
CBR1	P16152	start	14.84 (0.78) [8]	15.11 (1.22) [8]	0.55 (0.24-0.85)	14.83	0.62	0.62
CCP110	O43303	end	20.89 (0.75) [8]	20.38 (0.37) [5]	0.78 (0.5-1)	20.82	1.00	0.62
CD14	P08571	end	17.04 (1.26) [10]	16.99 (0.81) [10]	0.56 (0.29-0.83)	17.99	1.00	0.20
CD14	P08571	change	-0.13 (0.24) [10]	-0.13 (0.09) [9]	0.62 (0.35-0.89)	-0.06	0.89	0.40
CD14	P08571	start	15.6 (1.22) [12]	14.63 (1.35) [9]	0.74 (0.51-0.97)	14.59	0.67	0.83
CD163	C9JHR8	end	15.55 (1.43) [9]	15.17 (1.72) [10]	0.58 (0.3-0.85)	16.27	0.80	0.44
CD44	H0YD13	change	-0.05 (0.11) [9]	-0.08 (0.09) [7]	0.7 (0.41-0.99)	-0.09	0.71	0.78
CD44	H0YD13	start	15.54 (0.98) [12]	15.09 (1.17) [8]	0.69 (0.42-0.96)	15.10	0.88	0.67
CD44	H0YD13	end	16.04 (1.13) [9]	16.41 (0.52) [9]	0.58 (0.29-0.87)	15.88	0.89	0.44
CD59	E9PNW4	end	14.54 (0.99) [8]	15.28 (0.45) [9]	0.69 (0.39-1)	14.43	1.00	0.62
CD99	P14209	end	16.21 (0.36) [7]	15.57 (0.69) [10]	0.84 (0.64-1)	16.04	0.80	0.86

**Protein Name**	**Uniprot ID**	**Time**	**mRS 0-2** Mean (SD) [N]	**mRS 3-6** Mean (SD) [N]	**AUC (95%CI)**	**Cut-off**	**Sens.**	**Spec.**
CD99L2	Q8TCZ2	end	16.28 (0.45) [9]	15.85 (0.52) [10]	0.71 (0.47-0.95)	15.52	0.40	1.00
CDH13	P55290	start	13.74 (0.63) [11]	14.76 (0.82) [7]	0.81 (0.57-1)	14.86	0.57	1.00
CDH13	P55290	change	-0.08 (0.11) [10]	0 (0.05) [7]	0.71 (0.45-0.98)	-0.05	1.00	0.40
CDH13	P55290	end	14.79 (1.02) [11]	14.51 (0.73) [10]	0.57 (0.31-0.84)	13.91	0.30	1.00
CDH2	P19022	end	15.64 (0.39) [9]	15.52 (0.51) [10]	0.56 (0.26-0.85)	15.74	0.70	0.67
CDH4	P55283	end	12.59 (0.53) [7]	12.6 (0.61) [6]	0.55 (0.18-0.91)	12.43	0.83	0.57
CDH6	D6RF86	end	13.13 (0.73) [9]	12.87 (0.5) [7]	0.57 (0.26-0.88)	13.19	0.86	0.44
CFD	K7ERG9	end	16.46 (0.84) [10]	16.72 (0.61) [9]	0.59 (0.31-0.86)	16.22	0.89	0.40
CFD	K7ERG9	start	15.43 (0.59) [10]	15.33 (0.98) [9]	0.52 (0.23-0.81)	14.74	0.33	0.90
CFD	K7ERG9	change	-0.07 (0.02) [8]	-0.07 (0.09) [8]	0.56 (0.22-0.9)	-0.08	0.62	0.75
CFH	P08603	start	16.69 (0.52) [12]	17.09 (1.27) [10]	0.57 (0.31-0.82)	16.65	0.80	0.42
CFH	P08603	change	0.02 (0.1) [11]	0.06 (0.07) [10]	0.63 (0.38-0.88)	0.05	0.40	0.91
CFH	P08603	end	16.4 (1.06) [11]	16.02 (0.33) [10]	0.58 (0.32-0.84)	15.86	0.40	0.82
CFHR1	B1AKG0	end	14.78 (0.79) [10]	15.2 (0.74) [10]	0.63 (0.37-0.89)	14.59	0.80	0.50
CFHR1	B1AKG0	start	15.79 (0.9) [12]	16.39 (1.02) [9]	0.69 (0.44-0.93)	16.58	0.56	0.83
CFHR1	B1AKG0	change	0.07 (0.07) [10]	0.06 (0.07) [9]	0.52 (0.24-0.81)	0.01	1.00	0.30

Protein Name	Uniprot ID	Time	mRS 0-2 Mean (SD) [N]	mRS 3-6 Mean (SD) [N]	AUC (95%CI)	Cut-off	Sens.	Spec.
CFI	E7ETH0	start	15.43 (0.42) [12]	15.35 (0.58) [9]	0.55 (0.28-0.82)	15.40	0.67	0.58
CFI	E7ETH0	change	0.03 (0.03) [10]	0.03 (0.04) [9]	0.54 (0.26-0.83)	0.03	0.78	0.50
CFI	E7ETH0	end	14.98 (0.42) [10]	14.86 (0.31) [10]	0.67 (0.41-0.93)	15.26	1.00	0.40
CFL1	E9PK25	end	14.93 (1.15) [10]	14.88 (1.19) [10]	0.53 (0.26-0.8)	16.64	1.00	0.10
CFL1	E9PK25	change	0 (0.1) [9]	0.01 (0.13) [9]	0.56 (0.26-0.85)	0.04	0.56	0.78
CFL1	E9PK25	start	15.05 (0.73) [11]	15.27 (1.32) [9]	0.6 (0.33-0.87)	16.25	0.33	0.91
CHGA	P10645	change	-0.09 (0.12) [9]	-0.08 (0.06) [6]	0.52 (0.2-0.84)	-0.21	1.00	0.22
CHGA	P10645	start	14.44 (0.98) [11]	14.54 (1.14) [6]	0.53 (0.2-0.86)	13.88	0.50	0.73
CHGA	P10645	end	15.61 (1.01) [10]	15.64 (0.76) [10]	0.54 (0.26-0.82)	15.47	0.50	0.80
CHGB	P05060	change	-0.09 (0.08) [10]	-0.1 (0.1) [8]	0.5 (0.2-0.8)	-0.10	0.62	0.60
CHGB	P05060	start	14.21 (1.19) [12]	13.76 (1.38) [8]	0.66 (0.38-0.93)	13.47	0.62	0.83
CHGB	P05060	end	15.58 (0.78) [10]	15.24 (0.81) [10]	0.66 (0.4-0.92)	15.74	0.80	0.60
CHI3L1	P36222	change	-0.09 (0.13) [10]	-0.05 (0.15) [8]	0.64 (0.34-0.93)	-0.08	0.75	0.70
CHI3L1	P36222	start	15.14 (1.63) [12]	15.97 (2.42) [8]	0.65 (0.36-0.94)	16.28	0.62	0.83
CHI3L1	P36222	end	16.66 (1.17) [10]	16.58 (0.8) [10]	0.51 (0.24-0.78)	17.55	1.00	0.20
CHI3L2	Q15782	start	13.9 (1.5) [8]	15.17 (1.42) [5]	0.72 (0.42-1)	14.80	0.60	0.88
CHI3L2	Q15782	change	-0.02 (0.22) [6]	0.07 (0.09) [5]	0.6 (0.23-0.97)	-0.06	1.00	0.33

**Protein Name**	**Uniprot ID**	**Time**	**mRS 0-2** Mean (SD) [N]	**mRS 3-6** Mean (SD) [N]	**AUC (95%CI)**	**Cut-off**	**Sens.**	**Spec.**
CHI3L2	Q15782	end	13.74 (1.5) [9]	13.57 (1.12) [9]	0.51 (0.22-0.8)	14.01	0.44	0.78
CHL1	O00533	end	14.47 (0.85) [10]	14.44 (0.78) [10]	0.57 (0.29-0.85)	14.19	0.60	0.80
CHL1	O00533	start	13.84 (0.83) [11]	13.23 (1.41) [7]	0.74 (0.45-1)	13.19	0.71	0.82
CHL1	O00533	change	-0.04 (0.07) [9]	-0.09 (0.09) [7]	0.63 (0.34-0.93)	-0.03	0.86	0.44
CKB	P12277	end	14.62 (1.21) [8]	15.24 (2.33) [7]	0.55 (0.24-0.87)	14.56	0.57	0.62
CKB	P12277	start	16.57 (1.57) [10]	16.28 (1.38) [8]	0.54 (0.25-0.82)	18.28	1.00	0.20
CKB	P12277	change	0.18 (0.22) [8]	0.07 (0.21) [6]	0.52 (0.15-0.89)	0.15	0.67	0.62
CLCNKB	A0A087X136	end	15.71 (0.78) [9]	15.34 (0.93) [10]	0.62 (0.35-0.89)	15.61	0.70	0.67
CLEC11A	Q9Y240	end	13.21 (0.51) [8]	12.89 (0.65) [7]	0.61 (0.29-0.92)	13.05	0.71	0.62
CLEC3B	E9PHK0	change	-0.03 (0.05) [10]	-0.02 (0.04) [9]	0.51 (0.23-0.79)	0.01	0.22	0.90
CLEC3B	E9PHK0	end	16.15 (0.59) [10]	16.1 (0.35) [10]	0.6 (0.33-0.87)	16.36	0.90	0.50
CLEC3B	E9PHK0	start	15.82 (0.49) [12]	15.78 (0.59) [9]	0.61 (0.34-0.88)	15.81	0.78	0.67
CLIC1	O00299	end	14.32 (0.87) [5]	13.94 (0.98) [6]	0.6 (0.22-0.98)	13.38	0.33	1.00
CLSTN1	O94985	change	-0.06 (0.04) [7]	-0.07 (0.08) [5]	0.54 (0.16-0.93)	-0.03	0.80	0.43
CLSTN1	O94985	start	14.92 (0.73) [9]	14.91 (1.22) [5]	0.56 (0.18-0.93)	15.15	0.80	0.44
CLSTN1	O94985	end	15.64 (0.85) [10]	15.56 (0.76) [10]	0.53 (0.25-0.81)	15.62	0.60	0.70
CLSTN3	Q9BQT9	end	12.63 (1.71) [8]	10.79 (1.83) [9]	0.82 (0.6-1)	10.61	0.67	1.00

Protein Name	Uniprot ID	Time	mRS 0-2 Mean (SD) [N]	mRS 3-6 Mean (SD) [N]	AUC (95%CI)	Cut-off	Sens.	Spec.
CLU	P10909	change	-0.02 (0.09) [11]	-0.04 (0.05) [10]	0.61 (0.35-0.87)	-0.06	0.50	0.91
CLU	P10909	start	16.52 (0.52) [12]	16.2 (0.68) [10]	0.68 (0.43-0.93)	15.92	0.50	0.92
CLU	P10909	end	17.19 (1.29) [11]	16.86 (0.44) [10]	0.56 (0.3-0.82)	16.79	0.50	0.73
CNDP1	Q96KN2	change	-0.05 (0.05) [11]	-0.05 (0.07) [9]	0.57 (0.29-0.84)	-0.03	0.78	0.55
CNDP1	Q96KN2	end	16.05 (0.65) [11]	15.84 (0.56) [10]	0.59 (0.33-0.86)	16.45	0.90	0.45
CNDP1	Q96KN2	start	15.36 (0.79) [12]	15.04 (1.3) [9]	0.68 (0.42-0.93)	15.22	0.78	0.67
CNTN1	Q12860	start	14.11 (0.82) [10]	13.97 (1.46) [6]	0.62 (0.27-0.97)	13.36	0.50	0.90
CNTN1	Q12860	change	-0.04 (0.03) [8]	-0.04 (0.1) [6]	0.54 (0.18-0.9)	-0.09	0.33	1.00
CNTN1	Q12860	end	14.62 (0.73) [10]	14.64 (0.81) [10]	0.53 (0.26-0.8)	14.93	0.80	0.40
CNTN2	A0A1W2PQ11	start	14.08 (1.12) [11]	14.45 (1.22) [5]	0.53 (0.18-0.87)	16.19	0.20	1.00
CNTN2	A0A1W2PQ11	change	-0.03 (0.05) [9]	-0.03 (0.1) [5]	0.56 (0.16-0.95)	-0.05	0.40	0.89
CNTN2	A0A1W2PQ11	end	14.46 (0.86) [10]	14.69 (0.78) [10]	0.56 (0.29-0.83)	14.29	0.80	0.40
COL15A1	A0A087X0K0	end	14 (0.53) [5]	13.55 (0.81) [5]	0.72 (0.34-1)	13.46	0.60	1.00
COL18A1	P39060	end	14.78 (1.02) [10]	14.7 (0.5) [10]	0.61 (0.34-0.88)	15.08	0.90	0.40
COL1A1	P02452	end	16.65 (1.87) [11]	15.69 (1.12) [10]	0.66 (0.41-0.91)	16.93	1.00	0.45
COL1A2	A0A087WTA8	end	15.79 (1.3) [10]	15.27 (0.7) [10]	0.64 (0.37-0.91)	16.00	1.00	0.50
COL2A1	P02458	end	15.21 (2.14) [9]	16.88 (1.3) [7]	0.76 (0.5-1)	16.19	0.86	0.78

Protein Name	Uniprot ID	Time	mRS 0-2<br>Mean (SD) [N]	mRS 3-6<br>Mean (SD) [N]	AUC (95%CI)	Cut-off	Sens.	Spec.
COL3A1	P02461	end	15.08 (0.97) [9]	14.86 (1.01) [8]	0.58 (0.28-0.89)	15.13	0.75	0.67
COL5A1	A0A087WXW9	end	13.13 (0.7) [5]	12.49 (1.14) [6]	0.7 (0.34-1)	13.18	0.83	0.60
COL6A1	A0A087X0S5	change	-0.07 (0.11) [8]	-0.05 (0.08) [5]	0.52 (0.16-0.89)	-0.10	0.80	0.50
COL6A1	A0A087X0S5	start	13.65 (1.24) [10]	13.75 (1.19) [5]	0.54 (0.21-0.87)	12.55	1.00	0.30
COL6A1	A0A087X0S5	end	14.54 (0.4) [9]	14.44 (0.41) [10]	0.68 (0.39-0.96)	14.67	0.90	0.67
COL6A3	P12111	end	14.21 (0.38) [10]	14.03 (0.35) [9]	0.64 (0.37-0.92)	14.33	0.89	0.50
COLEC12	Q5KU26	end	14.32 (0.56) [9]	14.36 (0.49) [8]	0.53 (0.22-0.83)	14.83	1.00	0.22
CORO1A	P31146	end	13.19 (1.32) [9]	13.25 (1.84) [8]	0.5 (0.19-0.81)	12.40	0.75	0.44
CORO1A	P31146	start	13.31 (0.81) [6]	12.93 (0.89) [6]	0.64 (0.27-1)	13.63	0.83	0.67
COTL1	Q14019	end	13.26 (0.75) [8]	13.56 (1.06) [9]	0.56 (0.26-0.85)	13.48	0.44	0.75
CP	P00450	change	0.01 (0.07) [11]	0.03 (0.04) [10]	0.56 (0.3-0.83)	0.02	0.50	0.82
CP	P00450	start	16.68 (0.4) [12]	17.05 (0.82) [10]	0.62 (0.38-0.87)	16.64	0.90	0.42
CP	P00450	end	16.6 (0.79) [11]	16.56 (0.24) [10]	0.63 (0.37-0.88)	16.31	0.80	0.55
CPB2	A0A087WSY5	end	15.91 (1.92) [11]	15.2 (0.54) [10]	0.72 (0.48-0.95)	15.26	0.80	0.64
CPB2	A0A087WSY5	change	0 (0.13) [11]	0.01 (0.03) [9]	0.51 (0.23-0.78)	0.01	0.67	0.55
CPB2	A0A087WSY5	start	15.83 (0.79) [12]	15.46 (0.64) [9]	0.64 (0.38-0.9)	16.09	0.89	0.58
CPE	P16870	change	0 (0.04) [9]	-0.05 (0.09) [9]	0.7 (0.43-0.97)	-0.01	0.78	0.67

Protein Name	Uniprot ID	Time	mRS 0-2 Mean (SD) [N]	mRS 3-6 Mean (SD) [N]	AUC (95%CI)	Cut-off	Sens.	Spec.
CPE	P16870	start	15.26 (0.99) [11]	14.76 (1.57) [9]	0.58 (0.3-0.85)	14.23	0.44	0.91
CPE	P16870	end	15.06 (0.95) [10]	15.49 (1.14) [10]	0.55 (0.28-0.82)	15.80	0.40	0.80
CPN1	P15169	start	15.4 (0.88) [10]	15.44 (0.45) [8]	0.52 (0.23-0.82)	15.82	0.88	0.50
CPN1	P15169	change	0.12 (0.17) [7]	0.05 (0.09) [5]	0.63 (0.28-0.98)	0.04	0.60	0.71
CPN1	P15169	end	14.29 (0.9) [8]	14.52 (1.1) [7]	0.55 (0.23-0.88)	14.69	0.57	0.75
CPN2	P22792	start	16.68 (1.09) [12]	16.7 (1.18) [9]	0.51 (0.25-0.77)	17.61	0.89	0.33
CPN2	P22792	end	15.28 (0.76) [10]	15.27 (0.98) [10]	0.52 (0.24-0.8)	14.77	0.50	0.80
CPN2	P22792	change	0.12 (0.15) [10]	0.08 (0.09) [9]	0.53 (0.24-0.83)	0.09	0.56	0.80
CPQ	Q9Y646	change	0 (0.03) [5]	-0.01 (0.08) [5]	0.52 (0.07-0.97)	-0.03	0.40	1.00
CPQ	Q9Y646	start	14.15 (0.85) [7]	14.31 (1.41) [5]	0.54 (0.15-0.94)	13.90	0.60	0.71
CPQ	Q9Y646	end	14.02 (0.8) [10]	14.15 (1.31) [10]	0.52 (0.24-0.8)	13.37	0.40	0.80
CPVL	Q9H3G5	change	0 (0.05) [6]	0 (0.07) [6]	0.53 (0.15-0.9)	0.06	0.33	1.00
CPVL	Q9H3G5	start	14.01 (0.88) [7]	14.26 (0.62) [6]	0.52 (0.17-0.88)	13.18	1.00	0.29
CPVL	Q9H3G5	end	14.03 (0.59) [9]	14.13 (0.89) [10]	0.53 (0.25-0.81)	14.97	0.30	1.00
CRP	P02741	start	14.27 (0.92) [11]	14.37 (1.64) [6]	0.52 (0.15-0.88)	14.20	0.67	0.64
CRP	P02741	end	14.83 (1.22) [8]	15.45 (1.47) [8]	0.67 (0.38-0.96)	15.64	0.62	0.75

Protein Name	Uniprot ID	Time	mRS 0-2 Mean (SD) [N]	mRS 3-6 Mean (SD) [N]	AUC (95%CI)	Cut-off	Sens.	Spec.
CRTAC1	A0A0C4DFP6	start	14.63 (0.68) [9]	13.87 (1.23) [7]	0.75 (0.46-1)	14.31	0.71	0.78
CRTAC1	A0A0C4DFP6	end	14.79 (0.43) [9]	14.62 (0.57) [10]	0.63 (0.36-0.9)	14.79	0.80	0.56
CRTAC1	A0A0C4DFP6	change	-0.01 (0.05) [8]	-0.04 (0.04) [7]	0.73 (0.46-1)	-0.05	0.57	0.88
CSF1	P09603	end	14.26 (0.56) [9]	13.91 (1.1) [9]	0.64 (0.34-0.94)	13.95	0.67	0.89
CSF1R	E9PEK4	end	15.68 (0.71) [9]	15.64 (0.49) [10]	0.53 (0.25-0.82)	16.36	1.00	0.22
CST3	P01034	end	17.56 (1.25) [11]	17.39 (0.38) [10]	0.61 (0.35-0.87)	17.46	0.80	0.55
CST3	P01034	start	16.18 (1.2) [12]	15.65 (2.05) [10]	0.58 (0.32-0.85)	16.55	0.80	0.50
CST3	P01034	change	-0.07 (0.09) [11]	-0.1 (0.13) [10]	0.59 (0.33-0.85)	-0.05	0.80	0.45
CSTB	P04080	end	15.6 (0.87) [10]	15.9 (1.13) [10]	0.6 (0.33-0.87)	16.01	0.60	0.80
CTBS	Q01459	end	14.05 (0.4) [8]	14.1 (0.71) [10]	0.54 (0.25-0.82)	13.82	0.50	0.75
CTSA	P10619	end	12.89 (0.57) [6]	12.75 (0.79) [8]	0.54 (0.21-0.87)	12.25	0.38	1.00
CTSB	P07858	end	14.95 (1.82) [9]	14.92 (1.06) [9]	0.52 (0.23-0.81)	13.63	1.00	0.22
CTSB	P07858	start	14.76 (1.05) [7]	14.7 (1.64) [5]	0.51 (0.07-0.96)	14.14	0.60	0.86
CTSC	P53634	end	13.76 (1.04) [7]	13.81 (0.5) [7]	0.61 (0.28-0.94)	13.95	0.57	0.86
CTSD	A0A1B0GV23	end	15.95 (0.77) [10]	16.24 (1.2) [10]	0.52 (0.24-0.8)	15.42	0.90	0.30
CTSD	A0A1B0GV23	start	15.53 (1.16) [12]	15.33 (1.21) [8]	0.54 (0.27-0.81)	15.61	0.62	0.58
CTSD	A0A1B0GV23	change	-0.03 (0.08) [10]	-0.07 (0.12) [8]	0.58 (0.28-0.87)	-0.20	0.25	1.00

Protein Name	Uniprot ID	Time	mRS 0-2 Mean (SD) [N]	mRS 3-6 Mean (SD) [N]	AUC (95%CI)	Cut-off	Sens.	Spec.
CTSH	A0A087X0D5	end	13.9 (0.9) [6]	14.2 (1.17) [10]	0.55 (0.25-0.85)	14.56	0.40	0.83
CTSL	P07711	change	0 (0.08) [6]	-0.02 (0.11) [5]	0.57 (0.18-0.95)	-0.05	0.40	0.83
CTSL	P07711	end	14.71 (0.84) [9]	14.56 (0.96) [10]	0.64 (0.36-0.92)	14.31	0.70	0.78
CTSL	P07711	start	15.2 (0.74) [7]	14.77 (1.21) [5]	0.57 (0.16-0.98)	13.83	0.40	1.00
CTSS	P25774	end	14.17 (0.71) [8]	14.28 (0.98) [9]	0.5 (0.2-0.8)	14.09	0.56	0.62
CTSZ	Q9UBR2	end	15.11 (1.11) [7]	15.46 (1.18) [7]	0.63 (0.31-0.95)	14.32	1.00	0.29
CUTA	O60888	end	14.75 (0.3) [6]	14.62 (1.84) [5]	0.53 (0.11-0.96)	13.91	0.40	1.00
CYCS	C9JFR7	end	13.03 (0.47) [9]	13.76 (1.33) [8]	0.75 (0.48-1)	13.41	0.62	0.89
DAG1	Q14118	end	15.32 (0.17) [9]	15.09 (0.52) [10]	0.72 (0.46-0.98)	14.95	0.50	1.00
DBH	P09172	start	13.84 (1.55) [7]	13.77 (1.09) [7]	0.55 (0.22-0.88)	14.55	0.86	0.43
DBI	A0A0A0MTI5	start	14.58 (0.21) [8]	14.64 (0.66) [8]	0.58 (0.25-0.9)	14.66	0.62	0.75
DBI	A0A0A0MTI5	change	-0.02 (0.06) [6]	-0.03 (0.06) [8]	0.6 (0.27-0.94)	0.00	0.88	0.50
DBI	A0A0A0MTI5	end	15.04 (0.6) [10]	15.14 (0.65) [9]	0.52 (0.24-0.8)	14.81	0.78	0.40
DCN	P07585	end	13.33 (0.99) [8]	12.98 (1.38) [8]	0.61 (0.3-0.92)	13.00	0.62	0.75
DKK3	F6SYF8	change	-0.07 (0.13) [10]	-0.09 (0.1) [7]	0.54 (0.24-0.84)	-0.05	0.86	0.50
DKK3	F6SYF8	end	15.52 (1.02) [10]	15.68 (0.63) [10]	0.55 (0.28-0.82)	15.51	0.50	0.80
DKK3	F6SYF8	start	14.67 (1.35) [12]	14.07 (1.6) [7]	0.63 (0.34-0.92)	14.89	0.86	0.58

Protein Name	Uniprot ID	Time	mRS 0-2 Mean (SD) [N]	mRS 3-6 Mean (SD) [N]	AUC (95%CI)	Cut-off	Sens.	Spec.
DNER	Q8NFT8	end	11.5 (0.49) [5]	11.65 (0.37) [5]	0.64 (0.25-1)	11.22	1.00	0.40
DPP7	Q9UHL4	end	12.4 (0.77) [6]	13.38 (1.15) [8]	0.75 (0.47-1)	12.84	0.75	0.83
DPYSL2	A0A1C7CYX9	start	15.63 (1.4) [6]	15.22 (1.27) [8]	0.6 (0.26-0.94)	16.65	1.00	0.33
DSC2	Q02487	end	13.87 (0.52) [9]	13.34 (0.67) [9]	0.78 (0.53-1)	13.63	0.78	0.89
ECM1	Q16610	end	14.48 (0.41) [10]	14.51 (0.4) [10]	0.53 (0.26-0.8)	14.63	0.40	0.80
ECM1	Q16610	change	0 (0.03) [8]	-0.02 (0.03) [9]	0.81 (0.58-1)	-0.01	0.78	0.88
ECM2	O94769	end	14.07 (0.53) [8]	13.91 (0.74) [9]	0.62 (0.34-0.91)	13.62	0.44	0.88
EEF1A1	P68104	end	15.12 (3.94) [9]	14.82 (1.31) [8]	0.54 (0.24-0.84)	13.05	1.00	0.33
EFCAB14	O75071	end	13.73 (0.99) [8]	13.69 (0.25) [9]	0.67 (0.34-1)	13.99	1.00	0.62
EFEMP1	A0A0U1RQV3	start	15.63 (1.05) [9]	15.6 (1.76) [7]	0.59 (0.25-0.93)	15.58	0.71	0.67
EFEMP1	A0A0U1RQV3	change	-0.03 (0.08) [7]	-0.03 (0.12) [7]	0.51 (0.18-0.84)	-0.03	0.71	0.43
EFEMP1	A0A0U1RQV3	end	16.2 (0.57) [10]	16.08 (0.66) [10]	0.59 (0.31-0.87)	16.07	0.60	0.80
EIF5A	I3L397	end	14.44 (1.1) [10]	14.56 (1.02) [9]	0.56 (0.28-0.83)	13.23	1.00	0.20
EIF5A	I3L397	start	14.13 (1.09) [8]	13.44 (1.08) [8]	0.55 (0.21-0.89)	15.04	1.00	0.38
EIF5A	I3L397	change	-0.01 (0.06) [6]	-0.09 (0.11) [7]	0.69 (0.38-1)	-0.07	0.43	1.00
ENDOD1	O94919	start	14.64 (0.43) [8]	14.47 (0.84) [5]	0.73 (0.34-1)	14.44	0.80	0.88
ENDOD1	O94919	change	-0.01 (0.03) [6]	-0.03 (0.07) [5]	0.67 (0.27-1)	-0.04	0.60	0.83
ENDOD1	O94919	end	14.88 (0.28) [9]	14.79 (0.52) [10]	0.59 (0.3-0.88)	14.82	0.70	0.67

Protein Name	Uniprot ID	Time	mRS 0-2 Mean (SD) [N]	mRS 3-6 Mean (SD) [N]	AUC (95%CI)	Cut-off	Sens.	Spec.
ENO1	P06733	end	15.85 (1.07) [10]	15.66 (1.1) [10]	0.56 (0.29-0.83)	16.62	0.90	0.30
ENO1	P06733	start	16.33 (0.74) [8]	16.63 (2.54) [9]	0.61 (0.32-0.9)	15.49	0.44	0.88
ENO1	P06733	change	0 (0.06) [7]	0.04 (0.16) [9]	0.6 (0.3-0.91)	0.06	0.44	1.00
ENO2	P09104	start	14.7 (1.09) [9]	15.67 (3.58) [8]	0.53 (0.23-0.83)	14.67	0.75	0.56
ENO2	P09104	change	0.07 (0.12) [7]	0.06 (0.21) [8]	0.66 (0.34-0.98)	0.04	0.75	0.71
ENO2	P09104	end	13.98 (1.11) [10]	14.56 (0.65) [9]	0.7 (0.44-0.96)	14.38	0.67	0.80
ENOPH1	A0A0C4DGY8	end	12.9 (1.09) [6]	13.05 (0.31) [6]	0.75 (0.41-1)	12.80	0.83	0.83
ENPP2	E7EUF1	end	16.17 (0.86) [10]	16.52 (0.9) [10]	0.6 (0.33-0.87)	15.57	1.00	0.30
ENPP2	E7EUF1	start	15.84 (0.98) [12]	15.34 (1.43) [9]	0.56 (0.3-0.83)	16.53	0.89	0.33
ENPP2	E7EUF1	change	-0.03 (0.09) [10]	-0.08 (0.11) [9]	0.63 (0.37-0.9)	-0.04	0.67	0.60
EPHA4	E9PG71	end	14.65 (0.69) [9]	14.54 (0.83) [10]	0.58 (0.3-0.85)	14.00	0.40	0.89
ERN1	O75460	start	17.75 (2.59) [11]	17.95 (1.35) [9]	0.57 (0.28-0.85)	18.96	0.89	0.55
ERN1	O75460	end	15.44 (2.13) [6]	14.52 (1.39) [6]	0.58 (0.19-0.97)	15.79	0.83	0.50
ERN1	O75460	change	0.07 (0.15) [6]	0.21 (0.14) [5]	0.8 (0.5-1)	0.27	0.60	1.00
ESD	H7BZT7	end	12.89 (1.04) [8]	12.47 (1.14) [7]	0.68 (0.38-0.98)	11.81	0.43	1.00
EXTL2	Q9UBQ6	end	14.15 (0.57) [9]	13.87 (0.6) [9]	0.6 (0.32-0.89)	14.05	0.67	0.67
F10	P00742	start	15.48 (0.78) [8]	15.5 (0.59) [8]	0.5 (0.19-0.81)	14.37	1.00	0.12

Protein Name	Uniprot ID	Time	**mRS 0-2** Mean (SD) [N]	**mRS 3-6** Mean (SD) [N]	**AUC (95%CI)**	**Cut-off**	**Sens.**	**Spec.**
F10	P00742	change	0.06 (0.04) [7]	0.08 (0.04) [6]	0.69 (0.36-1)	0.08	0.67	0.86
F10	P00742	end	14.3 (0.49) [9]	13.96 (0.47) [8]	0.71 (0.45-0.97)	14.48	0.88	0.56
F12	P00748	start	17.06 (0.99) [12]	17.13 (0.76) [9]	0.51 (0.24-0.78)	17.75	0.89	0.42
F12	P00748	change	0.04 (0.08) [10]	0.07 (0.07) [9]	0.57 (0.29-0.84)	0.02	0.89	0.40
F12	P00748	end	16.46 (0.65) [10]	15.99 (0.62) [10]	0.72 (0.48-0.96)	16.83	1.00	0.40
F13A1	P00488	end	12.87 (1.23) [7]	12.97 (1.81) [8]	0.61 (0.28-0.94)	12.79	0.75	0.71
F13B	P05160	start	14.25 (0.79) [8]	13.61 (2.68) [8]	0.52 (0.2-0.83)	14.87	0.88	0.38
F2	P00734	change	0.01 (0.05) [10]	0.04 (0.01) [9]	0.78 (0.55-1)	0.03	0.89	0.70
F2	P00734	start	16.51 (0.74) [12]	16.76 (0.68) [9]	0.56 (0.3-0.83)	16.83	0.44	0.75
F2	P00734	end	16.16 (0.44) [10]	16.04 (0.45) [10]	0.59 (0.32-0.86)	15.94	0.60	0.80
F5	A0A0A0MRJ7	start	15.04 (0.4) [12]	14.72 (0.47) [9]	0.74 (0.49-0.99)	15.01	0.89	0.75
F5	A0A0A0MRJ7	change	0.02 (0.04) [10]	-0.01 (0.07) [9]	0.63 (0.33-0.93)	-0.03	0.56	1.00
F5	A0A0A0MRJ7	end	14.55 (0.59) [10]	14.65 (1.19) [10]	0.52 (0.24-0.8)	13.80	0.40	0.90
F9	P00740	end	13.25 (0.42) [10]	13.17 (0.6) [9]	0.52 (0.23-0.81)	12.91	0.44	0.80
F9	P00740	change	0.05 (0.03) [9]	0.05 (0.04) [8]	0.54 (0.23-0.85)	0.03	0.38	0.89
F9	P00740	start	14.23 (0.49) [11]	14.12 (0.74) [9]	0.52 (0.22-0.81)	14.38	0.56	0.73
FAM3C	Q92520	start	14.04 (0.78) [10]	13.93 (1.14) [5]	0.58 (0.22-0.94)	13.73	0.80	0.60

Protein Name	Uniprot ID	Time	mRS 0-2 Mean (SD) [N]	mRS 3-6 Mean (SD) [N]	AUC (95%CI)	Cut-off	Sens.	Spec.
FAM3C	Q92520	end	14.75 (0.66) [10]	14.65 (0.61) [10]	0.6 (0.32-0.88)	14.77	0.70	0.70
FAM3C	Q92520	change	-0.05 (0.03) [8]	-0.06 (0.08) [5]	0.58 (0.19-0.96)	-0.07	0.40	0.88
FAM49B	Q9NUQ9	end	13.13 (0.99) [10]	13.23 (0.64) [8]	0.56 (0.28-0.84)	12.44	1.00	0.30
FBLN1	P23142	start	15.64 (1.05) [12]	15.01 (0.91) [9]	0.67 (0.42-0.91)	15.79	0.89	0.50
FBLN1	P23142	change	-0.05 (0.09) [11]	-0.07 (0.06) [9]	0.57 (0.3-0.83)	-0.03	0.89	0.45
FBLN1	P23142	end	16.39 (1.25) [11]	16.26 (0.49) [10]	0.54 (0.27-0.8)	16.39	0.70	0.55
FBLN5	G3V4U0	end	14.75 (0.66) [9]	13.97 (0.84) [9]	0.78 (0.54-1)	14.31	0.78	0.78
FBN1	P35555	end	15.41 (1.7) [6]	13.5 (1.17) [6]	0.83 (0.58-1)	14.53	0.83	0.83
FCGBP	Q9Y6R7	start	15.59 (1.65) [7]	13.73 (0.78) [5]	0.83 (0.55-1)	15.25	1.00	0.71
FCGBP	Q9Y6R7	end	16.16 (1.21) [8]	15.61 (1.29) [10]	0.58 (0.29-0.86)	15.50	0.50	0.75
FCGR2A	P12318	end	14.76 (0.49) [5]	14.39 (0.3) [5]	0.68 (0.27-1)	14.78	1.00	0.60
FCGR3A	A0A1W2PQB1	change	-0.06 (0.09) [7]	-0.1 (0.08) [7]	0.67 (0.34-1)	-0.12	0.71	0.86
FCGR3A	A0A1W2PQB1	start	14.49 (1.22) [9]	13.81 (1.14) [7]	0.67 (0.38-0.95)	14.38	0.71	0.67
FCGR3A	A0A1W2PQB1	end	15.33 (1.15) [10]	15.43 (0.81) [10]	0.52 (0.25-0.79)	13.91	1.00	0.20
FCN3	O75636	start	15.25 (1.25) [9]	15.67 (1.99) [8]	0.71 (0.43-0.98)	15.87	0.88	0.56
FETUB	Q9UGM5	end	14.59 (0.87) [10]	14.12 (0.48) [9]	0.62 (0.35-0.89)	14.77	1.00	0.40
FETUB	Q9UGM5	start	15.71 (1.13) [11]	15.33 (1.3) [8]	0.55 (0.24-0.85)	15.60	0.50	0.82
FETUB	Q9UGM5	change	0.11 (0.19) [9]	0.05 (0.07) [7]	0.51 (0.2-0.82)	0.09	0.43	0.78

Protein Name	Uniprot ID	Time	mRS 0-2 Mean (SD) [N]	mRS 3-6 Mean (SD) [N]	AUC (95%CI)	Cut-off	Sens.	Spec.
FGA	P02671	end	16.12 (0.89) [11]	16.05 (0.9) [10]	0.54 (0.27-0.81)	15.82	0.80	0.55
FGA	P02671	change	0.06 (0.09) [11]	0.03 (0.11) [10]	0.59 (0.33-0.85)	0.05	0.80	0.55
FGA	P02671	start	17.02 (0.92) [12]	16.4 (1.34) [10]	0.63 (0.38-0.89)	16.70	0.60	0.75
FGB	P02675	end	17.75 (1.39) [11]	17.8 (1.02) [10]	0.54 (0.27-0.81)	17.40	0.80	0.55
FGB	P02675	change	0.03 (0.13) [11]	0.01 (0.12) [10]	0.55 (0.29-0.82)	0.03	0.70	0.55
FGB	P02675	start	18.34 (1.32) [12]	17.81 (1.72) [10]	0.6 (0.34-0.86)	18.01	0.60	0.75
FGFR2	A0A0A0MR25	end	14.76 (0.52) [6]	14.4 (0.64) [6]	0.72 (0.4-1)	14.16	0.50	1.00
FGG	P02679	start	17.47 (1.29) [12]	17.09 (1.69) [10]	0.6 (0.34-0.86)	16.99	0.60	0.75
FGG	P02679	end	16.86 (1.38) [11]	16.95 (0.9) [10]	0.55 (0.28-0.83)	16.18	0.90	0.45
FGG	P02679	change	0.04 (0.13) [11]	0.01 (0.12) [10]	0.54 (0.27-0.8)	0.03	0.70	0.55
FKBP1A	P62942	end	15.38 (0.81) [9]	15.34 (1.12) [9]	0.57 (0.26-0.87)	15.02	0.78	0.67
FLNA	P21333	end	13.2 (0.94) [9]	13.35 (0.66) [8]	0.51 (0.21-0.81)	12.31	1.00	0.22
FLNA	P21333	start	13.79 (1.19) [9]	14.16 (0.9) [5]	0.67 (0.29-1)	13.71	0.80	0.78
FMOD	Q06828	end	13.47 (0.42) [8]	13.42 (1.05) [9]	0.54 (0.24-0.84)	13.57	0.78	0.50
FN1	P02751	end	16.69 (0.81) [11]	16.51 (0.54) [10]	0.5 (0.23-0.77)	16.55	0.60	0.64
FN1	P02751	start	16.65 (0.37) [12]	16.75 (0.9) [10]	0.58 (0.33-0.84)	16.65	0.70	0.58
FN1	P02751	change	0 (0.06) [11]	0.02 (0.05) [10]	0.51 (0.24-0.77)	0.04	0.90	0.36

Protein Name	Uniprot ID	Time	mRS 0-2<br>Mean (SD) [N]	mRS 3-6<br>Mean (SD) [N]	AUC (95%CI)	Cut-off	Sens.	Spec.
FRZB	Q92765	end	13.45 (0.89) [7]	13.05 (1.15) [8]	0.64 (0.33-0.95)	13.43	0.75	0.71
FSTL1	Q12841	start	13.68 (1.58) [10]	12.53 (0.98) [6]	0.72 (0.45-0.98)	14.05	1.00	0.50
FSTL1	Q12841	change	-0.05 (0.2) [8]	-0.09 (0.13) [6]	0.58 (0.26-0.91)	-0.02	0.83	0.50
FSTL1	Q12841	end	14.03 (1.43) [10]	13.98 (1.12) [10]	0.58 (0.31-0.85)	14.05	0.50	0.80
FTH1	P02794	end	13.25 (1.5) [9]	12.68 (2.43) [9]	0.63 (0.35-0.91)	12.46	0.56	0.78
FTL	P02792	end	16.3 (1.95) [10]	16.08 (2.33) [10]	0.56 (0.29-0.83)	17.52	0.90	0.30
FUCA1	P04066	end	13.75 (0.91) [10]	14 (1.01) [9]	0.54 (0.26-0.83)	13.04	1.00	0.40
FUCA2	Q9BTY2	change	-0.01 (0.04) [6]	-0.03 (0.15) [6]	0.58 (0.18-0.98)	0.03	0.50	1.00
FUCA2	Q9BTY2	end	13.73 (0.87) [9]	13.52 (1.54) [10]	0.6 (0.33-0.87)	13.08	0.50	0.78
FUCA2	Q9BTY2	start	13.82 (1.19) [8]	13.84 (1.25) [6]	0.52 (0.18-0.86)	13.20	0.50	0.75
FXYD6	Q9H0Q3	end	14.76 (0.73) [7]	14.66 (0.45) [8]	0.62 (0.31-0.94)	14.57	0.62	0.71
GALNT2	Q10471	end	13.78 (0.47) [7]	13.37 (0.47) [9]	0.73 (0.46-1)	13.46	0.67	0.86
GANAB	Q14697	end	12.65 (0.74) [8]	12.5 (0.61) [9]	0.61 (0.31-0.91)	12.50	0.67	0.75
GAP43	P17677	end	11.5 (1.2) [5]	12.34 (1.31) [8]	0.72 (0.42-1)	11.38	0.88	0.60
GAPDH	P04406	change	0 (0.09) [8]	-0.04 (0.12) [10]	0.59 (0.31-0.87)	-0.04	0.50	0.75
GAPDH	P04406	start	14.98 (0.85) [10]	15.19 (1.56) [10]	0.52 (0.23-0.81)	14.49	0.50	0.80
GAPDH	P04406	end	15.27 (0.98) [10]	15.67 (1.16) [10]	0.63 (0.37-0.89)	15.00	0.80	0.50

Protein Name	Uniprot ID	Time	mRS 0-2 Mean (SD) [N]	mRS 3-6 Mean (SD) [N]	AUC (95%CI)	Cut-off	Sens.	Spec.
GC	P02774	change	0.02 (0.08) [11]	0.02 (0.05) [10]	0.57 (0.31-0.84)	0.00	0.90	0.45
GC	P02774	start	17.15 (0.51) [12]	17.33 (0.85) [10]	0.52 (0.26-0.79)	16.95	0.50	0.75
GC	P02774	end	17.01 (0.91) [11]	16.91 (0.38) [10]	0.58 (0.32-0.85)	16.69	0.80	0.55
GDI2	P50395	change	0.01 (0.05) [5]	-0.01 (0.11) [8]	0.55 (0.21-0.89)	-0.06	0.38	1.00
GDI2	P50395	end	14.17 (0.75) [10]	14.39 (1.22) [10]	0.63 (0.36-0.9)	14.24	0.70	0.70
GDI2	P50395	start	14.07 (1.09) [6]	14.42 (1.07) [8]	0.6 (0.25-0.96)	13.57	0.88	0.50
GFAP	A0A1W2PR46	end	15.57 (1.57) [5]	14.59 (1.4) [5]	0.72 (0.34-1)	15.22	0.80	0.80
GFAP	A0A1W2PR46	start	17.11 (1.83) [8]	17.94 (3) [8]	0.62 (0.31-0.94)	17.17	0.75	0.62
GGH	Q92820	end	13.89 (0.75) [8]	13.79 (0.74) [7]	0.57 (0.24-0.9)	13.98	0.71	0.62
GGH	Q92820	start	14.38 (0.34) [9]	14.2 (0.58) [7]	0.6 (0.27-0.93)	13.88	0.43	1.00
GGH	Q92820	change	0.03 (0.04) [6]	0 (0.05) [5]	0.77 (0.44-1)	0.02	0.80	0.83
GLOD4	F6TLX2	end	15.1 (1.1) [7]	14.6 (0.82) [7]	0.67 (0.34-1)	14.83	0.86	0.71
GM2A	P17900	start	14.87 (1.24) [11]	14.4 (1.32) [7]	0.62 (0.32-0.92)	14.98	0.86	0.64
GM2A	P17900	change	-0.05 (0.1) [9]	-0.08 (0.09) [7]	0.6 (0.3-0.91)	-0.03	0.86	0.56
GM2A	P17900	end	15.57 (0.79) [10]	15.73 (0.61) [10]	0.51 (0.23-0.79)	15.58	0.50	0.80
GNPTG	Q9UJJ9	end	15.51 (0.36) [9]	15.22 (0.64) [9]	0.65 (0.37-0.94)	15.09	0.56	0.89
GOLM1	Q8NBJ4	end	13.4 (0.53) [8]	13.21 (0.32) [8]	0.59 (0.28-0.91)	13.70	1.00	0.38

Protein Name	Uniprot ID	Time	mRS 0-2 Mean (SD) [N]	mRS 3-6 Mean (SD) [N]	AUC (95%CI)	Cut-off	Sens.	Spec.
GOT1	P17174	end	14.82 (0.65) [10]	15.25 (0.76) [10]	0.68 (0.43-0.93)	15.33	0.60	0.80
GPI	A0A0A0MTS2	end	13.38 (0.85) [10]	13.95 (0.71) [9]	0.76 (0.53-0.99)	13.49	0.78	0.70
GPLD1	P80108	start	15.69 (1.11) [10]	15.9 (1.05) [9]	0.53 (0.24-0.83)	16.56	1.00	0.40
GPLD1	P80108	change	0.12 (0.13) [7]	0.07 (0.11) [6]	0.62 (0.28-0.96)	-0.02	0.33	1.00
GPLD1	P80108	end	14.33 (1) [8]	14.56 (1.07) [7]	0.55 (0.24-0.87)	14.15	0.71	0.50
GPR37	O15354	end	15.19 (0.36) [9]	14.87 (0.37) [9]	0.74 (0.46-1)	15.11	0.89	0.78
GPR37L1	O60883	change	-0.04 (0.18) [7]	-0.07 (0.1) [7]	0.55 (0.2-0.9)	0.09	1.00	0.43
GPR37L1	O60883	end	14.22 (1.03) [10]	13.99 (0.91) [10]	0.62 (0.35-0.89)	14.61	0.90	0.40
GPR37L1	O60883	start	14.03 (1.34) [9]	12.94 (0.84) [7]	0.71 (0.44-0.99)	13.44	0.86	0.67
GPX3	A0A087X1J7	change	0 (0.05) [10]	-0.01 (0.03) [9]	0.63 (0.35-0.91)	0.00	0.78	0.70
GPX3	A0A087X1J7	end	15.98 (0.37) [10]	15.78 (0.71) [10]	0.71 (0.44-0.98)	15.59	0.70	0.90
GPX3	A0A087X1J7	start	16.2 (0.54) [12]	15.7 (0.6) [9]	0.76 (0.54-0.98)	15.89	0.67	0.83
GRB2	P62993	end	12.84 (1.13) [5]	12.82 (1.36) [6]	0.53 (0.14-0.93)	12.68	0.67	0.60
GSN	P06396	change	-0.01 (0.05) [11]	-0.02 (0.04) [10]	0.59 (0.32-0.86)	-0.01	0.70	0.73
GSN	P06396	start	16.19 (0.38) [12]	16.04 (0.54) [10]	0.63 (0.37-0.89)	16.17	0.70	0.67
GSN	P06396	end	16.45 (0.69) [11]	16.38 (0.37) [10]	0.53 (0.26-0.79)	16.34	0.60	0.64
GSS	P48637	end	14.47 (1.56) [6]	15 (1.84) [9]	0.63 (0.3-0.96)	15.37	0.56	0.83

**Protein Name**	**Uniprot ID**	**Time**	**mRS 0-2** Mean (SD) [N]	**mRS 3-6** Mean (SD) [N]	**AUC (95%CI)**	**Cut-off**	**Sens.**	**Spec.**
GSTO1	P78417	start	14.72 (0.59) [5]	14.12 (1) [5]	0.68 (0.27-1)	13.91	0.60	1.00
GSTO1	P78417	end	15.31 (0.62) [10]	15.31 (1.06) [10]	0.62 (0.34-0.9)	15.26	0.80	0.60
GSTP1	P09211	change	-0.03 (0.07) [7]	-0.03 (0.12) [8]	0.57 (0.25-0.89)	0.00	0.50	0.86
GSTP1	P09211	start	14.87 (0.83) [9]	15.52 (1.28) [8]	0.62 (0.34-0.91)	15.04	0.62	0.67
GSTP1	P09211	end	15.63 (0.81) [10]	15.67 (1.01) [10]	0.51 (0.24-0.78)	15.82	0.70	0.50
HBA1	P69905	start	19.97 (2.16) [12]	19.81 (3.23) [10]	0.54 (0.28-0.8)	17.51	0.30	0.92
HBA1	P69905	end	21.23 (2.23) [11]	20.8 (2.7) [10]	0.53 (0.26-0.79)	19.21	0.30	0.91
HBA1	P69905	change	-0.08 (0.19) [11]	-0.06 (0.23) [10]	0.59 (0.32-0.86)	-0.05	0.60	0.73
HBB	P68871	start	20.07 (2.4) [12]	19.92 (3.84) [10]	0.58 (0.31-0.84)	20.81	0.80	0.50
HBB	P68871	end	21.64 (2.88) [11]	20.78 (2.82) [10]	0.54 (0.27-0.8)	23.41	1.00	0.27
HBB	P68871	change	-0.08 (0.17) [11]	-0.05 (0.26) [10]	0.64 (0.37-0.9)	-0.08	0.70	0.73
HBD	P02042	change	-0.07 (0.15) [10]	-0.02 (0.21) [8]	0.6 (0.31-0.89)	-0.08	0.75	0.60
HBD	P02042	end	18.58 (2.34) [11]	18.71 (1.32) [9]	0.57 (0.3-0.83)	17.04	0.89	0.36
HBD	P02042	start	17.67 (1.75) [11]	17.98 (3.12) [9]	0.53 (0.25-0.8)	17.96	0.78	0.45
HBE1	P02100	end	18.89 (3.15) [5]	17.26 (0.73) [5]	0.64 (0.2-1)	18.34	1.00	0.60
HBG2	P69892	start	15.86 (2.05) [10]	17.02 (3.24) [6]	0.62 (0.32-0.92)	15.05	1.00	0.50
HBG2	P69892	end	16.08 (3.24) [10]	16.44 (0.95) [8]	0.58 (0.28-0.87)	14.65	1.00	0.40

Protein Name	Uniprot ID	Time	mRS 0-2<br>Mean (SD) [N]	mRS 3-6<br>Mean (SD)<br>[N]	AUC (95%CI)	Cut-off	Sens.	Spec.
HBG2	P69892	change	-0.03 (0.17) [8]	0.04 (0.23) [6]	0.52 (0.17-0.87)	-0.17	1.00	0.25
HEXA	H3BP20	end	13.94 (0.57) [9]	14.26 (0.74) [8]	0.6 (0.27-0.92)	14.47	0.50	1.00
HEXB	P07686	end	13.63 (0.72) [9]	14.15 (1.34) [9]	0.56 (0.27-0.85)	14.48	0.33	1.00
HGFAC	D6RAR4	end	14.17 (0.42) [9]	13.92 (0.6) [7]	0.62 (0.3-0.94)	14.04	0.71	0.78
HGFAC	D6RAR4	start	15.16 (0.66) [10]	15.08 (0.53) [9]	0.57 (0.29-0.85)	15.57	1.00	0.30
HGFAC	D6RAR4	change	0.09 (0.12) [7]	0.06 (0.07) [6]	0.64 (0.31-0.97)	0.05	0.67	0.71
HIST1H1E	P10412	end	14.74 (1.53) [5]	13.98 (1.58) [7]	0.66 (0.26-1)	13.99	0.71	0.80
HIST1H2BK	O60814	end	15.68 (1.25) [6]	15.06 (1.55) [10]	0.63 (0.34-0.92)	14.22	0.40	1.00
HIST1H4A	P62805	change	-0.1 (0.2) [6]	-0.02 (0.26) [6]	0.53 (0.15-0.9)	-0.26	1.00	0.33
HIST1H4A	P62805	start	15.97 (1.57) [6]	15.66 (3.68) [6]	0.64 (0.26-1)	14.89	0.67	0.83
HIST1H4A	P62805	end	16.27 (2.48) [11]	16.23 (2.1) [10]	0.53 (0.26-0.79)	16.52	0.60	0.64
HLA-C	A0A140T921	end	13.4 (0.8) [8]	14.65 (1.09) [8]	0.86 (0.66-1)	13.57	0.88	0.75
HP	P00738	start	18.31 (2.44) [12]	19.68 (1.43) [10]	0.67 (0.43-0.9)	17.27	1.00	0.42
HP	P00738	end	16.24 (2.89) [11]	15.59 (1.54) [10]	0.51 (0.24-0.78)	18.32	1.00	0.27
HP	P00738	change	0.17 (0.44) [11]	0.24 (0.11) [10]	0.67 (0.42-0.93)	0.12	0.90	0.55
HPR	P00739	end	15.66 (2.51) [10]	14.83 (1.34) [10]	0.56 (0.29-0.83)	17.49	1.00	0.20
HPR	P00739	start	16.74 (2.08) [12]	16.81 (1.13) [9]	0.52 (0.26-0.78)	18.51	1.00	0.33

Protein Name	Uniprot ID	Time	mRS 0-2<br>Mean (SD) [N]	mRS 3-6<br>Mean (SD) [N]	AUC (95%CI)	Cut-off	Sens.	Spec.
HPR	P00739	change	0.1 (0.34) [10]	0.12 (0.12) [9]	0.56 (0.27-0.84)	0.06	0.89	0.50
HPRT1	P00492	end	14.12 (0.9) [6]	14.12 (0.61) [9]	0.57 (0.21-0.94)	14.33	0.78	0.67
HPX	P02790	change	0.05 (0.09) [11]	0.05 (0.03) [10]	0.59 (0.32-0.86)	0.01	0.90	0.45
HPX	P02790	start	18.37 (0.58) [12]	18.52 (0.5) [10]	0.56 (0.3-0.82)	18.68	0.60	0.67
HPX	P02790	end	17.75 (0.65) [11]	17.56 (0.66) [10]	0.61 (0.35-0.87)	17.74	0.70	0.64
HRG	P04196	change	0.05 (0.07) [11]	0.06 (0.06) [10]	0.62 (0.36-0.87)	0.01	0.90	0.36
HRG	P04196	start	16.86 (0.86) [12]	16.87 (0.61) [10]	0.52 (0.26-0.77)	17.40	0.90	0.33
HRG	P04196	end	16.1 (0.51) [11]	15.89 (0.48) [10]	0.61 (0.34-0.87)	16.09	0.80	0.55
HSP90B1	P14625	end	14.45 (0.71) [9]	14.3 (0.71) [10]	0.52 (0.24-0.8)	13.63	0.20	1.00
HSP90AA1	P07900	change	0.05 (0.07) [8]	-0.03 (0.13) [6]	0.73 (0.39-1)	0.03	0.83	0.75
HSP90AA1	P07900	end	14.5 (0.94) [9]	14.72 (1.07) [8]	0.53 (0.23-0.82)	13.82	0.88	0.33
HSP90AA1	P07900	start	15.34 (0.86) [11]	14.35 (1.11) [6]	0.79 (0.49-1)	14.77	0.83	0.82
HSPA1B	A0A0G2JIW1	start	15.14 (1.1) [6]	14.03 (1.15) [8]	0.73 (0.43-1)	14.94	0.88	0.67
HSPA1B	A0A0G2JIW1	end	15.32 (0.83) [10]	15.37 (1.31) [7]	0.51 (0.2-0.83)	14.42	0.29	0.90
HSPA5	P11021	end	14.92 (0.72) [9]	14.76 (0.77) [10]	0.58 (0.31-0.85)	14.36	0.30	0.89
HSPA8	P11142	start	14.98 (0.94) [10]	15.83 (3.68) [9]	0.51 (0.22-0.8)	15.18	0.78	0.50
HSPA8	P11142	end	15.12 (1.02) [10]	14.91 (1.58) [10]	0.54 (0.26-0.82)	14.13	0.40	0.90

**Protein Name**	**Uniprot ID**	**Time**	**mRS 0-2** Mean (SD) [N]	**mRS 3-6** Mean (SD) [N]	**AUC (95%CI)**	**Cut-off**	**Sens.**	**Spec.**
HSPA8	P11142	change	-0.03 (0.11) [8]	0.04 (0.23) [9]	0.57 (0.28-0.86)	-0.03	0.67	0.62
HSPG2	P98160	change	-0.02 (0.08) [8]	-0.03 (0.08) [7]	0.55 (0.23-0.88)	-0.06	0.57	0.75
HSPG2	P98160	start	13.51 (1.62) [10]	13.91 (1.36) [7]	0.56 (0.25-0.87)	14.26	0.43	0.90
HSPG2	P98160	end	14.34 (0.52) [10]	14.24 (1.29) [10]	0.7 (0.44-0.96)	14.10	0.70	0.80
HTRA1	Q92743	start	13.45 (0.87) [8]	13.22 (1.08) [6]	0.56 (0.2-0.92)	12.63	0.50	0.88
HTRA1	Q92743	end	14.43 (0.41) [9]	14.28 (0.66) [9]	0.58 (0.3-0.86)	14.55	0.78	0.44
HTRA1	Q92743	change	-0.06 (0.03) [6]	-0.06 (0.11) [5]	0.57 (0.1-1)	-0.08	0.60	0.83
HYAL1	Q12794	start	17.88 (1.42) [6]	17.92 (1.39) [6]	0.53 (0.15-0.9)	17.07	0.50	0.83
HYOU1	A0A087X054	end	14.04 (0.44) [8]	13.79 (0.31) [5]	0.72 (0.42-1)	13.77	0.60	0.88
ICOSLG	K4DIA0	change	-0.1 (0.14) [8]	-0.09 (0.1) [7]	0.57 (0.25-0.89)	-0.07	0.71	0.62
ICOSLG	K4DIA0	start	14.06 (1.02) [10]	13.74 (1.57) [7]	0.64 (0.34-0.95)	13.45	0.57	0.80
ICOSLG	K4DIA0	end	15.08 (1.01) [10]	15.03 (0.51) [10]	0.63 (0.36-0.9)	15.00	0.60	0.80
IGF2	P01344	end	14.43 (0.25) [6]	14.73 (0.47) [6]	0.69 (0.37-1)	14.37	0.83	0.50
IGFALS	P35858	change	0.08 (0.09) [10]	0.07 (0.04) [8]	0.52 (0.24-0.81)	0.11	1.00	0.30
IGFALS	P35858	start	16.05 (0.95) [12]	16.01 (0.74) [9]	0.53 (0.26-0.8)	16.79	1.00	0.33
IGFALS	P35858	end	14.86 (0.95) [10]	14.71 (0.38) [9]	0.52 (0.23-0.81)	14.57	0.78	0.50
IGFBP2	P18065	end	14.09 (0.5) [9]	14.27 (0.82) [10]	0.54 (0.27-0.82)	14.78	0.30	1.00

Protein Name	Uniprot ID	Time	**mRS 0-2** Mean (SD) [N]	**mRS 3-6** Mean (SD) [N]	**AUC (95%CI)**	**Cut-off**	**Sens.**	**Spec.**
IGFBP5	P24593	end	12.34 (0.7) [7]	11.67 (0.93) [7]	0.8 (0.52-1)	11.55	0.71	1.00
IGFBP6	P24592	start	16.48 (1.3) [12]	15.42 (1.93) [7]	0.69 (0.38-1)	15.47	0.71	0.75
IGFBP6	P24592	change	-0.08 (0.13) [10]	-0.13 (0.16) [7]	0.66 (0.35-0.96)	-0.13	0.71	0.70
IGFBP6	P24592	end	17.45 (1.11) [10]	17.61 (0.91) [10]	0.56 (0.29-0.83)	18.26	0.40	0.90
IGFBP7	Q16270	change	-0.04 (0.07) [11]	-0.04 (0.16) [10]	0.55 (0.27-0.82)	-0.06	0.70	0.55
IGFBP7	Q16270	end	16.34 (1.05) [11]	16.46 (1.16) [10]	0.53 (0.26-0.79)	15.77	0.40	0.82
IGFBP7	Q16270	start	15.65 (0.91) [12]	15.94 (2.16) [10]	0.58 (0.32-0.84)	15.69	0.80	0.58
IGHA1	A0A286YEY1	change	0.06 (0.14) [11]	0.07 (0.08) [10]	0.59 (0.33-0.85)	-0.02	1.00	0.27
IGHA1	A0A286YEY1	start	19.25 (1.25) [12]	19.24 (1.01) [10]	0.52 (0.26-0.77)	19.31	0.60	0.58
IGHA1	A0A286YEY1	end	18.61 (0.91) [11]	18.1 (0.76) [10]	0.66 (0.42-0.91)	18.37	0.70	0.73
IGHA2	A0A286YEY5	start	19.06 (1.08) [12]	19.68 (1.24) [10]	0.68 (0.43-0.92)	19.45	0.70	0.75
IGHA2	A0A286YEY5	change	0.05 (0.17) [11]	0.1 (0.09) [10]	0.69 (0.45-0.93)	0.03	0.80	0.64
IGHA2	A0A286YEY5	end	18.57 (0.99) [11]	18.04 (0.6) [10]	0.71 (0.47-0.95)	18.05	0.70	0.82
IGHD	A0A0A0MS09	start	16.36 (1) [10]	15.94 (1.76) [9]	0.64 (0.38-0.91)	16.79	0.89	0.50
IGHD	A0A0A0MS09	end	15.39 (1.19) [8]	15.52 (1.46) [5]	0.5 (0.14-0.86)	17.91	0.20	1.00
IGHG1	P01857	change	0.05 (0.07) [11]	0.05 (0.06) [10]	0.56 (0.29-0.84)	0.07	0.50	0.91
IGHG1	P01857	start	20.46 (0.53) [12]	20.38 (0.82) [10]	0.52 (0.25-0.79)	20.49	0.60	0.67

Protein Name	Uniprot ID	Time	mRS 0-2<br>Mean (SD) [N]	mRS 3-6<br>Mean (SD) [N]	AUC (95%CI)	Cut-off	Sens.	Spec.
IGHG1	P01857	end	19.75 (0.32) [11]	19.52 (0.54) [10]	0.64 (0.37-0.9)	19.50	0.60	0.82
IGHG2	P01859	start	20.3 (0.61) [12]	20.21 (0.59) [10]	0.52 (0.25-0.8)	20.39	0.60	0.75
IGHG2	P01859	end	19.93 (0.48) [11]	19.35 (0.72) [10]	0.73 (0.5-0.95)	19.48	0.50	0.91
IGHG2	P01859	change	0.04 (0.09) [11]	0.05 (0.05) [10]	0.66 (0.4-0.92)	0.05	0.60	0.91
IGHG3	P01860	end	16.58 (0.91) [10]	16.27 (1.09) [10]	0.6 (0.32-0.88)	15.68	0.50	0.90
IGHG3	P01860	start	17.77 (0.83) [12]	17.85 (1.27) [9]	0.51 (0.23-0.79)	19.29	0.22	1.00
IGHG3	P01860	change	0.09 (0.11) [10]	0.1 (0.11) [9]	0.52 (0.24-0.8)	0.14	0.33	0.90
IGHG4	A0A286YFJ8	start	16.55 (1.09) [12]	16.81 (0.82) [10]	0.58 (0.32-0.83)	16.33	0.80	0.50
IGHG4	A0A286YFJ8	end	15.49 (1.09) [11]	16.21 (1.01) [10]	0.7 (0.46-0.94)	16.80	0.50	0.91
IGHG4	A0A286YFJ8	change	0.09 (0.14) [11]	0.04 (0.07) [10]	0.63 (0.37-0.88)	0.07	0.90	0.45
IGHM	A0A1B0GUU9	end	17.85 (1.32) [10]	16.96 (1.09) [10]	0.67 (0.42-0.92)	17.56	0.80	0.50
IGHM	A0A1B0GUU9	start	19.31 (1.62) [12]	19.1 (2.27) [9]	0.56 (0.3-0.83)	19.04	0.78	0.58
IGHM	A0A1B0GUU9	change	0.11 (0.19) [10]	0.14 (0.18) [9]	0.61 (0.34-0.89)	0.19	0.44	0.90
IGHV1-18	A0A0C4DH31	end	14.72 (0.84) [8]	14.36 (0.78) [8]	0.59 (0.28-0.91)	14.45	0.62	0.75
IGHV1-18	A0A0C4DH31	start	14.96 (1.18) [9]	14.66 (0.85) [5]	0.64 (0.33-0.96)	15.07	0.80	0.67
IGHV1-2	P23083	start	16.4 (0.56) [11]	16.09 (0.49) [7]	0.69 (0.43-0.95)	16.10	0.57	0.82

Protein Name	Uniprot ID	Time	mRS 0-2<br>Mean (SD) [N]	mRS 3-6<br>Mean (SD) [N]	AUC (95%CI)	Cut-off	Sens.	Spec.
IGHV1-2	P23083	end	15.42 (0.76) [10]	14.98 (0.79) [8]	0.65 (0.37-0.93)	15.53	0.75	0.70
IGHV1-2	P23083	change	0.08 (0.06) [9]	0.06 (0.05) [6]	0.61 (0.24-0.98)	0.02	0.50	1.00
IGHV1-69	P01742	start	15.49 (0.72) [7]	15.06 (2.2) [5]	0.54 (0.16-0.93)	15.17	0.80	0.43
IGHV1OR15-1	A0A075B7D0	start	17.11 (1.8) [11]	17.74 (1.16) [9]	0.61 (0.34-0.87)	15.81	1.00	0.36
IGHV1OR15-1	A0A075B7D0	change	0.04 (0.09) [9]	0.03 (0.07) [9]	0.58 (0.29-0.87)	0.03	0.56	0.78
IGHV1OR15-1	A0A075B7D0	end	16.93 (1.58) [10]	17.15 (1.33) [10]	0.52 (0.24-0.8)	16.21	0.90	0.40
IGHV2-26	A0A0B4J1V2	start	14.11 (1.13) [10]	14.08 (1.16) [8]	0.58 (0.28-0.87)	14.29	0.75	0.60
IGHV2-26	A0A0B4J1V2	end	13.89 (0.6) [10]	13.59 (0.89) [9]	0.59 (0.3-0.87)	14.03	0.67	0.60
IGHV2-26	A0A0B4J1V2	change	0.01 (0.07) [8]	0.03 (0.08) [7]	0.55 (0.23-0.88)	0.09	0.29	1.00
IGHV2-5	P01817	start	13.27 (1.28) [7]	12.98 (1.19) [5]	0.57 (0.19-0.96)	12.83	0.60	0.71
IGHV2-5	P01817	end	12.58 (1.8) [7]	12.91 (0.39) [7]	0.57 (0.22-0.92)	12.22	1.00	0.43
IGHV3-15	A0A0B4J1V0	end	16.23 (0.62) [10]	15.97 (0.61) [10]	0.64 (0.38-0.9)	16.68	1.00	0.40
IGHV3-15	A0A0B4J1V0	change	0.02 (0.1) [10]	0 (0.03) [9]	0.56 (0.28-0.83)	-0.02	0.89	0.40
IGHV3-15	A0A0B4J1V0	start	16.2 (0.64) [12]	15.98 (0.48) [9]	0.57 (0.31-0.83)	16.68	1.00	0.33
IGHV3-30	P01768	change	0.04 (0.07) [10]	0.03 (0.04) [9]	0.62 (0.32-0.92)	0.03	0.67	0.80
IGHV3-30	P01768	end	17.04 (0.35) [10]	16.69 (0.51) [10]	0.72 (0.46-0.98)	16.75	0.70	0.90

Protein Name	Uniprot ID	Time	mRS 0-2 Mean (SD) [N]	mRS 3-6 Mean (SD) [N]	AUC (95%CI)	Cut-off	Sens.	Spec.
IGHV3-30	P01768	start	17.47 (0.48) [12]	17.29 (0.59) [9]	0.59 (0.32-0.86)	17.04	0.44	0.92
IGHV3-38	A0A0C4DH36	change	-0.01 (0.07) [10]	0.04 (0.04) [9]	0.81 (0.58-1)	0.01	0.89	0.80
IGHV3-38	A0A0C4DH36	start	15.47 (0.54) [12]	15.64 (0.63) [9]	0.61 (0.35-0.88)	15.39	0.78	0.58
IGHV3-38	A0A0C4DH36	end	15.67 (0.69) [10]	14.97 (0.4) [10]	0.83 (0.63-1)	15.31	0.90	0.80
IGHV3-49	A0A0A0MS15	change	0.06 (0.06) [10]	0.08 (0.05) [9]	0.59 (0.31-0.87)	0.07	0.67	0.70
IGHV3-49	A0A0A0MS15	start	17.95 (0.81) [12]	17.78 (0.84) [9]	0.56 (0.27-0.84)	17.63	0.56	0.75
IGHV3-49	A0A0A0MS15	end	17.11 (0.6) [10]	16.33 (0.6) [10]	0.84 (0.66-1)	17.13	1.00	0.60
IGHV3-64D	A0A0J9YX35	change	0.02 (0.06) [10]	-0.01 (0.05) [5]	0.64 (0.33-0.95)	0.04	1.00	0.40
IGHV3-64D	A0A0J9YX35	end	15.89 (0.65) [10]	15.75 (0.57) [6]	0.57 (0.25-0.88)	16.57	1.00	0.20
IGHV3-64D	A0A0J9YX35	start	16.01 (0.64) [12]	15.71 (0.56) [9]	0.59 (0.33-0.85)	15.48	0.44	0.83
IGHV3-7	P01780	change	0.05 (0.1) [10]	0.02 (0.03) [9]	0.52 (0.24-0.81)	0.00	0.33	0.90
IGHV3-7	P01780	end	17.45 (0.35) [10]	17.26 (0.63) [10]	0.59 (0.32-0.86)	17.23	0.50	0.80
IGHV3-7	P01780	start	17.86 (0.61) [12]	17.69 (0.71) [9]	0.55 (0.27-0.82)	16.98	0.22	1.00
IGHV3-72	A0A0B4J1Y9	start	17.12 (0.94) [12]	17.28 (0.57) [9]	0.58 (0.32-0.85)	16.85	0.89	0.42
IGHV3-72	A0A0B4J1Y9	change	0.05 (0.1) [10]	0.05 (0.05) [9]	0.59 (0.32-0.86)	0.03	0.67	0.60
IGHV3-72	A0A0B4J1Y9	end	16.63 (0.52) [10]	16.49 (0.62) [10]	0.6 (0.33-0.87)	16.32	0.50	0.80
IGHV3OR16-12	A0A075B7B8	start	14.15 (0.73) [9]	14.03 (0.85) [6]	0.56 (0.21-0.9)	13.87	0.50	0.78

Protein Name	Uniprot ID	Time	mRS 0-2 Mean (SD) [N]	mRS 3-6 Mean (SD) [N]	AUC (95%CI)	Cut-off	Sens.	Spec.
IGHV3OR16-12	A0A075B7B8	end	13.33 (0.69) [9]	12.94 (0.17) [7]	0.67 (0.34-0.99)	13.27	1.00	0.67
IGHV3OR16-9	A0A0B4J2B5	start	20.42 (0.72) [12]	20.41 (0.57) [10]	0.56 (0.3-0.82)	20.13	0.80	0.50
IGHV3OR16-9	A0A0B4J2B5	change	0.04 (0.1) [11]	0.04 (0.04) [10]	0.52 (0.25-0.79)	0.03	0.60	0.73
IGHV3OR16-9	A0A0B4J2B5	end	20.11 (0.79) [11]	19.84 (0.49) [10]	0.59 (0.33-0.85)	19.60	0.40	0.91
IGHV4-34	P06331	change	0.07 (0.19) [10]	0.04 (0.06) [9]	0.64 (0.35-0.94)	0.04	0.67	0.80
IGHV4-34	P06331	end	15.23 (0.7) [10]	15.31 (0.6) [10]	0.57 (0.3-0.84)	14.81	0.90	0.40
IGHV4-34	P06331	start	15.74 (0.89) [12]	16.05 (1.07) [9]	0.62 (0.36-0.88)	15.58	0.78	0.58
IGHV5-51	A0A0C4DH38	end	16.58 (2.34) [11]	15.84 (0.84) [10]	0.59 (0.33-0.85)	15.12	0.30	1.00
IGHV5-51	A0A0C4DH38	start	16.4 (0.78) [12]	16.44 (0.86) [9]	0.51 (0.24-0.78)	16.27	0.67	0.50
IGHV5-51	A0A0C4DH38	change	-0.01 (0.17) [11]	0.03 (0.05) [9]	0.52 (0.24-0.79)	-0.07	1.00	0.18
IGKC	P01834	start	20.6 (0.64) [12]	21.52 (1.79) [10]	0.71 (0.47-0.95)	21.09	0.60	0.83
IGKC	P01834	change	0.01 (0.16) [11]	0.09 (0.11) [10]	0.72 (0.47-0.97)	0.04	0.70	0.91
IGKC	P01834	end	20.78 (1.85) [11]	20.01 (0.58) [10]	0.67 (0.42-0.92)	20.00	0.60	0.82
IGKV1-12	A0A0C4DH73	start	17.74 (1.1) [12]	17.97 (1.09) [9]	0.56 (0.29-0.82)	17.94	0.56	0.67
IGKV1-12	A0A0C4DH73	change	0.02 (0.12) [11]	0.06 (0.06) [9]	0.61 (0.34-0.87)	0.07	0.44	0.91
IGKV1-12	A0A0C4DH73	end	17.55 (0.87) [11]	17.08 (0.67) [10]	0.62 (0.37-0.87)	17.61	0.90	0.36

**Protein Name**	**Uniprot ID**	**Time**	**mRS 0-2** Mean (SD) [N]	**mRS 3-6** Mean (SD) [N]	**AUC (95%CI)**	**Cut-off**	**Sens.**	**Spec.**
IGKV1-16	P04430	change	0.02 (0.07) [6]	0.02 (0.04) [6]	0.58 (0.21-0.96)	0.01	0.83	0.50
IGKV1-16	P04430	end	14.32 (0.93) [7]	14.42 (0.46) [8]	0.55 (0.21-0.9)	13.79	1.00	0.43
IGKV1-16	P04430	start	14.71 (0.72) [8]	14.75 (0.94) [8]	0.55 (0.23-0.86)	14.59	0.75	0.50
IGKV1-17	P01599	end	15.76 (0.5) [10]	15.46 (0.81) [10]	0.66 (0.4-0.92)	15.13	0.50	0.90
IGKV1-17	P01599	start	16.43 (0.67) [12]	16.47 (0.91) [9]	0.53 (0.24-0.81)	16.75	0.56	0.67
IGKV1-17	P01599	change	0.05 (0.05) [10]	0.06 (0.06) [9]	0.53 (0.23-0.84)	0.07	0.56	0.80
IGKV1-27	A0A075B6S5	start	15.85 (0.58) [9]	16.16 (1.01) [8]	0.6 (0.29-0.91)	15.91	0.75	0.56
IGKV1-27	A0A075B6S5	change	0 (0.04) [6]	0.06 (0.05) [5]	0.83 (0.5-1)	0.04	0.80	1.00
IGKV1-27	A0A075B6S5	end	15.88 (0.58) [9]	15.68 (0.39) [7]	0.54 (0.23-0.85)	16.21	1.00	0.33
IGKV1-5	P01602	end	16.84 (0.43) [10]	16.62 (0.76) [10]	0.62 (0.36-0.88)	16.53	0.50	0.80
IGKV1-5	P01602	change	-0.01 (0.05) [10]	0.01 (0.04) [9]	0.6 (0.33-0.87)	-0.01	0.67	0.60
IGKV1-5	P01602	start	16.83 (0.51) [12]	16.7 (0.75) [9]	0.55 (0.26-0.83)	16.23	0.44	0.92
IGKV1-8	A0A0C4DH67	end	16.95 (1.08) [11]	16.52 (0.67) [10]	0.6 (0.35-0.85)	17.00	0.90	0.36
IGKV1-8	A0A0C4DH67	change	0.02 (0.12) [11]	0.06 (0.07) [9]	0.61 (0.34-0.87)	0.06	0.67	0.64
IGKV1-8	A0A0C4DH67	start	17.21 (1.23) [12]	17.49 (1) [9]	0.64 (0.38-0.9)	18.03	0.44	0.92
IGKV1D-33	P01593	end	17.81 (0.6) [11]	17.32 (0.91) [10]	0.69 (0.45-0.93)	17.63	0.70	0.73

Protein Name	Uniprot ID	Time	mRS 0-2<br>Mean (SD) [N]	mRS 3-6<br>Mean (SD) [N]	AUC (95%CI)	Cut-off	Sens.	Spec.
IGKV1D-33	P01593	start	18.27 (1.14) [12]	18.24 (0.97) [9]	0.5 (0.24-0.76)	17.37	0.89	0.33
IGKV1D-33	P01593	change	0.04 (0.1) [11]	0.06 (0.07) [9]	0.58 (0.31-0.85)	0.07	0.44	0.82
IGKV1D-37	A0A075B6S9	start	17.9 (1.32) [10]	17.83 (0.96) [7]	0.51 (0.21-0.82)	18.64	0.86	0.40
IGKV1D-37	A0A075B6S9	end	15.69 (0.92) [7]	16.84 (1.37) [5]	0.77 (0.47-1)	16.17	0.80	0.71
IGKV2-28	A0A075B6P5	start	16.68 (0.88) [11]	17.01 (0.93) [9]	0.63 (0.37-0.89)	17.49	0.44	0.82
IGKV2-28	A0A075B6P5	end	16.28 (0.52) [10]	16.46 (0.73) [9]	0.62 (0.35-0.9)	16.59	0.56	0.80
IGKV2-28	A0A075B6P5	change	0.04 (0.07) [9]	0.03 (0.04) [8]	0.56 (0.26-0.85)	0.09	1.00	0.22
IGKV2-29	A2NJV5	start	17.27 (0.82) [12]	16.98 (1.17) [9]	0.59 (0.32-0.87)	17.23	0.78	0.58
IGKV2-29	A2NJV5	end	16.41 (0.59) [10]	16.16 (0.71) [10]	0.6 (0.34-0.86)	16.78	0.90	0.40
IGKV2-29	A2NJV5	change	0.07 (0.09) [10]	0.05 (0.04) [9]	0.52 (0.24-0.81)	0.07	0.44	0.80
IGKV2-40	A0A087WW87	start	14 (1.06) [5]	14.52 (0.92) [5]	0.64 (0.2-1)	14.21	0.80	0.80
IGKV2-40	A0A087WW87	end	13.45 (0.56) [7]	14.28 (1.85) [7]	0.65 (0.34-0.97)	13.69	0.57	0.86
IGKV2D-24	A0A075B6R9	start	17.8 (0.74) [12]	17.04 (1.33) [9]	0.67 (0.39-0.94)	16.76	0.56	0.92
IGKV2D-24	A0A075B6R9	end	16.93 (0.55) [10]	16.19 (0.92) [10]	0.76 (0.52-1)	16.37	0.70	0.90
IGKV2D-24	A0A075B6R9	change	0.05 (0.08) [10]	0.04 (0.07) [9]	0.56 (0.28-0.84)	0.05	0.67	0.60
IGKV3-15	P01624	end	17.91 (0.61) [10]	17.71 (1.08) [10]	0.53 (0.25-0.81)	17.80	0.60	0.70
IGKV3-15	P01624	start	17.5 (0.53) [12]	17.91 (0.6) [9]	0.69 (0.44-0.94)	17.85	0.67	0.75

**Protein Name**	**Uniprot ID**	**Time**	**mRS 0-2** Mean (SD) [N]	**mRS 3-6** Mean (SD) [N]	**AUC (95%CI)**	**Cut-off**	**Sens.**	**Spec.**
IGKV3-15	P01624	change	-0.02 (0.05) [10]	0.02 (0.08) [9]	0.59 (0.32-0.86)	0.00	0.56	0.70
IGKV3-20	P01619	end	19.08 (1.22) [11]	18.51 (0.7) [10]	0.67 (0.43-0.92)	18.53	0.60	0.82
IGKV3-20	P01619	start	19.14 (0.61) [12]	19.32 (1.19) [10]	0.52 (0.26-0.79)	19.26	0.50	0.75
IGKV3-20	P01619	change	0.01 (0.1) [11]	0.05 (0.07) [10]	0.6 (0.34-0.86)	0.07	0.40	0.91
IGKV3-7	A0A075B6H7	start	18.88 (1.83) [11]	18.22 (0.92) [8]	0.57 (0.29-0.84)	19.82	1.00	0.36
IGKV3-7	A0A075B6H7	end	19.23 (1.5) [10]	19.12 (1.03) [9]	0.53 (0.26-0.81)	20.79	1.00	0.20
IGKV3-7	A0A075B6H7	change	0.02 (0.15) [9]	-0.04 (0.07) [7]	0.6 (0.3-0.9)	0.10	1.00	0.33
IGKV3D-11	A0A0A0MRZ8	start	17.98 (0.59) [12]	18.13 (0.44) [9]	0.55 (0.29-0.81)	17.15	1.00	0.17
IGKV3D-11	A0A0A0MRZ8	end	17.66 (0.52) [10]	17.47 (0.48) [10]	0.63 (0.37-0.89)	18.11	1.00	0.30
IGKV3D-11	A0A0A0MRZ8	change	0.04 (0.1) [10]	0.03 (0.02) [9]	0.68 (0.4-0.95)	0.01	1.00	0.50
IGKV3D-15	A0A087WSY6	end	17.31 (1.02) [10]	16.56 (0.78) [9]	0.72 (0.48-0.97)	16.01	0.44	1.00
IGKV3D-15	A0A087WSY6	start	18.59 (0.74) [12]	17.59 (0.74) [9]	0.82 (0.64-1)	18.15	0.78	0.83
IGKV3D-15	A0A087WSY6	change	0.07 (0.06) [10]	0.05 (0.06) [8]	0.74 (0.46-1)	0.07	0.75	0.80
IGKV3D-20	A0A0C4DH25	end	18.51 (0.78) [10]	17.77 (0.83) [10]	0.77 (0.55-0.99)	18.30	0.90	0.60
IGKV3D-20	A0A0C4DH25	start	19.88 (0.96) [12]	18.64 (0.97) [9]	0.81 (0.63-1)	19.23	0.78	0.83
IGKV3D-20	A0A0C4DH25	change	0.1 (0.1) [10]	0.05 (0.08) [9]	0.63 (0.35-0.91)	0.01	0.44	1.00
IGKV4-1	P06312	end	17.43 (0.26) [10]	17.29 (0.77) [10]	0.51 (0.21-0.81)	17.57	0.60	0.80

Protein Name	Uniprot ID	Time	mRS 0-2 Mean (SD) [N]	mRS 3-6 Mean (SD) [N]	AUC (95%CI)	Cut-off	Sens.	Spec.
IGKV4-1	P06312	start	17.83 (0.68) [12]	17.8 (0.75) [9]	0.5 (0.23-0.77)	17.86	0.67	0.58
IGKV4-1	P06312	change	0.04 (0.06) [10]	0.03 (0.07) [9]	0.52 (0.23-0.81)	0.04	0.67	0.60
IGKV6D-21	A0A0A0MT36	start	14.32 (0.99) [6]	15.51 (1.12) [6]	0.83 (0.58-1)	14.35	1.00	0.67
IGLC3	P0DOY3	change	0.04 (0.1) [10]	0.04 (0.06) [9]	0.62 (0.35-0.89)	0.03	0.67	0.70
IGLC3	P0DOY3	start	20.78 (0.93) [12]	20.87 (0.47) [9]	0.54 (0.27-0.81)	20.78	0.89	0.42
IGLC3	P0DOY3	end	20.5 (0.35) [10]	20.2 (0.59) [10]	0.65 (0.4-0.9)	20.17	0.50	0.80
IGLL1	P15814	start	16.48 (0.8) [10]	17.15 (1.09) [6]	0.72 (0.42-1)	17.27	0.67	0.80
IGLL5	A0A0B4J231	start	18.97 (0.69) [12]	18.65 (1) [10]	0.58 (0.32-0.83)	19.72	1.00	0.25
IGLL5	A0A0B4J231	change	0.07 (0.13) [11]	0.05 (0.07) [10]	0.54 (0.26-0.81)	0.05	0.60	0.64
IGLL5	A0A0B4J231	end	18.23 (0.66) [11]	17.95 (0.65) [10]	0.63 (0.36-0.89)	17.60	0.50	0.91
IGLV1-47	P01700	change	-0.01 (0.15) [11]	0.02 (0.04) [9]	0.53 (0.25-0.8)	0.01	0.67	0.55
IGLV1-47	P01700	end	17.62 (2.05) [11]	16.41 (0.73) [10]	0.76 (0.54-0.98)	16.31	0.60	0.91
IGLV1-47	P01700	start	17.29 (0.77) [12]	16.79 (0.76) [9]	0.72 (0.48-0.96)	16.50	0.56	0.92
IGLV1-51	P01701	change	0.07 (0.09) [10]	0.07 (0.07) [7]	0.66 (0.35-0.96)	0.08	0.71	0.80
IGLV1-51	P01701	end	15.75 (0.77) [10]	15.54 (0.6) [8]	0.6 (0.32-0.88)	15.44	0.62	0.70
IGLV1-51	P01701	start	16.69 (0.84) [12]	16.63 (0.83) [8]	0.51 (0.23-0.79)	16.84	0.62	0.67
IGLV3-10	A0A075B6K4	change	0.06 (0.06) [7]	0.06 (0.08) [8]	0.52 (0.18-0.85)	-0.01	0.25	1.00

Protein Name	Uniprot ID	Time	mRS 0-2 Mean (SD) [N]	mRS 3-6 Mean (SD) [N]	AUC (95%CI)	Cut-off	Sens.	Spec.
IGLV3-10	A0A075B6K4	end	15.47 (0.65) [10]	15.15 (0.77) [9]	0.62 (0.34-0.9)	15.07	0.56	0.90
IGLV3-10	A0A075B6K4	start	16.44 (0.65) [7]	16.18 (1.11) [9]	0.56 (0.25-0.86)	15.92	0.44	0.86
IGLV3-19	P01714	start	15.13 (0.49) [12]	14.93 (0.44) [8]	0.62 (0.36-0.89)	15.32	0.88	0.42
IGLV3-19	P01714	end	14.65 (0.66) [10]	14.59 (0.54) [9]	0.52 (0.24-0.8)	15.16	0.89	0.30
IGLV3-19	P01714	change	0.03 (0.05) [10]	0.02 (0.03) [7]	0.57 (0.28-0.87)	0.02	0.71	0.60
IGLV3-21	P80748	change	0.08 (0.18) [8]	0.02 (0.04) [7]	0.52 (0.2-0.84)	0.08	1.00	0.25
IGLV3-21	P80748	start	16.18 (1.2) [11]	16.22 (0.86) [8]	0.51 (0.24-0.79)	15.32	0.88	0.36
IGLV3-21	P80748	end	15.74 (1.34) [9]	15.86 (0.67) [8]	0.54 (0.24-0.84)	15.44	0.88	0.44
IGLV3-25	P01717	change	0.11 (0.18) [9]	0.07 (0.08) [9]	0.57 (0.27-0.86)	0.09	0.56	0.78
IGLV3-25	P01717	end	14.85 (0.64) [10]	14.68 (0.81) [10]	0.65 (0.38-0.92)	15.00	0.80	0.60
IGLV3-25	P01717	start	15.68 (0.88) [11]	15.88 (1.13) [9]	0.6 (0.32-0.87)	16.90	0.33	1.00
IGLV3-9	A0A075B6K5	end	15.31 (0.68) [10]	15.26 (0.75) [10]	0.53 (0.26-0.8)	14.87	0.40	0.80
IGLV3-9	A0A075B6K5	change	0.08 (0.13) [10]	0.09 (0.1) [9]	0.58 (0.3-0.86)	0.09	0.56	0.70
IGLV3-9	A0A075B6K5	start	16.32 (1.38) [12]	16.74 (1.32) [9]	0.57 (0.31-0.84)	16.30	0.78	0.50
IGLV6-57	P01721	start	15.55 (0.64) [12]	15.35 (1.07) [9]	0.56 (0.27-0.85)	14.42	0.33	1.00
IGLV6-57	P01721	end	15.02 (0.57) [10]	14.47 (1) [9]	0.69 (0.42-0.96)	14.51	0.56	0.90

Protein Name	Uniprot ID	Time	mRS 0-2<br>Mean (SD) [N]	mRS 3-6<br>Mean (SD) [N]	AUC (95%CI)	Cut-off	Sens.	Spec.
IGLV6-57	P01721	change	0.05 (0.07) [10]	0.05 (0.07) [8]	0.55 (0.24-0.86)	0.06	0.62	0.80
IGLV7-46	A0A075B6I9	change	0.08 (0.14) [10]	0.04 (0.05) [8]	0.54 (0.24-0.84)	0.03	0.88	0.50
IGLV7-46	A0A075B6I9	start	16.4 (0.7) [12]	15.91 (0.71) [9]	0.67 (0.43-0.91)	16.75	1.00	0.33
IGLV7-46	A0A075B6I9	end	15.52 (1.07) [10]	15.15 (0.63) [9]	0.61 (0.34-0.88)	16.02	1.00	0.30
IGLV8-61	A0A075B6I0	end	15.72 (0.79) [9]	15.34 (0.61) [6]	0.7 (0.41-1)	15.39	0.67	0.78
IGLV8-61	A0A075B6I0	start	16.65 (1.08) [10]	16.2 (0.5) [5]	0.66 (0.37-0.95)	16.85	1.00	0.40
IGSF8	Q969P0	end	15.08 (0.43) [9]	15.01 (0.71) [10]	0.56 (0.27-0.84)	14.63	0.40	0.89
IGSF8	Q969P0	start	14.46 (1.16) [8]	14.63 (1.21) [5]	0.52 (0.17-0.88)	13.35	1.00	0.25
IGSF8	Q969P0	change	-0.07 (0.15) [6]	-0.04 (0.11) [5]	0.53 (0.14-0.93)	-0.01	0.80	0.50
IL31RA	Q8NI17	end	23.53 (0.54) [8]	23.39 (0.6) [8]	0.61 (0.31-0.91)	23.89	0.88	0.38
IL6ST	P40189	end	13.46 (0.53) [7]	13.62 (0.57) [7]	0.59 (0.26-0.92)	12.96	1.00	0.29
IMPAD1	Q9NX62	end	13.15 (0.99) [9]	13.22 (0.86) [6]	0.5 (0.18-0.82)	13.93	0.83	0.33
ISLR	O14498	start	14.25 (1.33) [10]	13.63 (1.12) [5]	0.7 (0.41-0.99)	15.20	1.00	0.50
ISLR	O14498	change	-0.05 (0.09) [8]	-0.09 (0.08) [5]	0.62 (0.27-0.98)	-0.06	0.80	0.62
ISLR	O14498	end	15.34 (0.81) [10]	15.35 (0.47) [10]	0.57 (0.3-0.84)	15.14	0.40	0.80
ITIH1	P19827	change	0.09 (0.12) [11]	0.12 (0.13) [10]	0.68 (0.43-0.94)	0.09	0.70	0.82

Protein Name	Uniprot ID	Time	mRS 0-2 Mean (SD) [N]	mRS 3-6 Mean (SD) [N]	AUC (95%CI)	Cut-off	Sens.	Spec.
ITIH1	P19827	end	15.67 (0.76) [11]	15.73 (0.48) [10]	0.54 (0.27-0.8)	14.83	1.00	0.27
ITIH1	P19827	start	16.96 (0.87) [12]	17.83 (2.16) [10]	0.62 (0.37-0.88)	17.49	0.70	0.67
ITIH2	P19823	end	15.84 (0.87) [11]	15.78 (0.53) [10]	0.51 (0.24-0.78)	15.15	0.90	0.36
ITIH2	P19823	change	0.08 (0.13) [11]	0.1 (0.08) [10]	0.7 (0.45-0.95)	0.08	0.70	0.73
ITIH2	P19823	start	16.89 (0.94) [12]	17.43 (0.94) [10]	0.69 (0.45-0.93)	17.34	0.80	0.67
ITIH3	Q06033	end	14.46 (0.76) [10]	14.67 (0.46) [10]	0.65 (0.39-0.91)	14.17	0.90	0.40
ITIH3	Q06033	change	0.09 (0.14) [10]	0.07 (0.07) [9]	0.6 (0.32-0.88)	0.09	0.56	0.80
ITIH3	Q06033	start	15.43 (0.77) [12]	15.84 (0.89) [9]	0.69 (0.43-0.94)	15.69	0.78	0.58
ITIH4	Q14624	end	16.56 (1.01) [11]	16.47 (0.42) [10]	0.56 (0.29-0.83)	16.20	0.90	0.36
ITIH4	Q14624	change	0.06 (0.12) [11]	0.07 (0.06) [10]	0.61 (0.35-0.87)	0.04	0.90	0.45
ITIH4	Q14624	start	17.39 (0.75) [12]	17.6 (0.79) [10]	0.6 (0.34-0.86)	17.67	0.80	0.58
ITIH5	C9J2H1	end	13.41 (0.52) [5]	13.08 (1.1) [6]	0.57 (0.19-0.95)	12.49	0.33	1.00
ITM2B	Q9Y287	end	12.63 (1.5) [6]	13.02 (1.66) [9]	0.56 (0.24-0.87)	14.21	0.33	1.00
ITPR2	Q14571	end	18.17 (1.09) [10]	18.58 (0.85) [10]	0.62 (0.36-0.88)	18.88	0.50	0.80
ITPR2	Q14571	start	18.91 (1.09) [12]	18.69 (0.66) [9]	0.63 (0.38-0.88)	19.53	1.00	0.42
ITPR2	Q14571	change	0.11 (0.24) [10]	0 (0.07) [9]	0.64 (0.38-0.91)	0.01	0.56	0.80

Protein Name	Uniprot ID	Time	mRS 0-2 Mean (SD) [N]	mRS 3-6 Mean (SD) [N]	AUC (95%CI)	Cut-off	Sens.	Spec.
JCHAIN	D6RD17	change	0.08 (0.14) [10]	0.09 (0.16) [8]	0.62 (0.33-0.92)	0.09	0.62	0.80
JCHAIN	D6RD17	end	17.44 (1.06) [10]	16.47 (1.35) [9]	0.68 (0.42-0.93)	16.60	0.44	0.90
JCHAIN	D6RD17	start	18.36 (1.25) [12]	18.08 (1.87) [9]	0.51 (0.24-0.77)	17.16	0.89	0.33
KIAA1549L	H0YDE5	end	13.63 (0.95) [9]	13.29 (0.82) [8]	0.64 (0.35-0.93)	13.27	0.62	0.78
KLK6	Q92876	start	14.98 (1.08) [12]	14.96 (1.5) [8]	0.57 (0.3-0.85)	15.12	0.75	0.50
KLK6	Q92876	change	-0.05 (0.11) [10]	-0.07 (0.11) [8]	0.59 (0.3-0.88)	-0.08	0.50	0.80
KLK6	Q92876	end	15.6 (1.13) [10]	15.93 (0.81) [10]	0.56 (0.29-0.83)	16.73	0.30	1.00
KLKB1	H0YAC1	start	15.57 (0.97) [12]	15.81 (1.02) [9]	0.54 (0.26-0.81)	15.15	0.89	0.42
KLKB1	H0YAC1	change	0.1 (0.12) [10]	0.09 (0.08) [8]	0.56 (0.27-0.85)	0.08	0.88	0.40
KLKB1	H0YAC1	end	14.21 (0.94) [10]	13.97 (0.42) [9]	0.57 (0.28-0.85)	14.38	0.89	0.40
KNG1	P01042	end	17.15 (0.51) [11]	17.13 (0.56) [10]	0.51 (0.24-0.78)	17.53	0.40	0.82
KNG1	P01042	start	17.63 (0.79) [12]	17.98 (0.73) [9]	0.6 (0.34-0.87)	17.49	0.89	0.50
KNG1	P01042	change	0.03 (0.05) [11]	0.05 (0.06) [9]	0.64 (0.37-0.9)	0.02	0.78	0.55
KRT1	P04264	end	15.01 (0.83) [10]	15.36 (2.83) [5]	0.6 (0.15-1)	13.92	0.60	0.90
KRT2	P35908	end	13.94 (1.37) [9]	14.88 (2.83) [5]	0.56 (0.16-0.95)	15.63	0.40	1.00
LAMA2	A0A087WX80	end	10.68 (1.33) [8]	9.7 (0.96) [7]	0.75 (0.49-1)	10.24	0.71	0.75
LAMP2	P13473	change	-0.06 (0.08) [9]	-0.1 (0.11) [8]	0.69 (0.41-0.98)	-0.05	0.88	0.56

Protein Name	Uniprot ID	Time	mRS 0-2<br>Mean (SD) [N]	mRS 3-6<br>Mean (SD) [N]	AUC (95%CI)	Cut-off	Sens.	Spec.
LAMP2	P13473	end	14.74 (0.71) [10]	15.12 (0.9) [10]	0.61 (0.35-0.87)	14.66	0.70	0.60
LAMP2	P13473	start	14.01 (0.89) [11]	13.64 (1.58) [8]	0.68 (0.4-0.97)	13.28	0.62	0.82
LBP	P18428	change	0.07 (0.05) [6]	0.01 (0.04) [7]	0.79 (0.49-1)	0.04	0.86	0.83
LBP	P18428	start	14.85 (0.59) [7]	14.31 (0.71) [9]	0.71 (0.44-0.99)	14.81	0.78	0.71
LBP	P18428	end	13.79 (0.8) [8]	14.17 (0.6) [8]	0.62 (0.33-0.92)	13.60	0.88	0.50
LCAT	P04180	change	0.01 (0.05) [9]	0.02 (0.04) [7]	0.7 (0.42-0.98)	0.00	0.86	0.67
LCAT	P04180	start	14.51 (0.37) [11]	15.15 (0.67) [9]	0.76 (0.51-1)	14.96	0.67	0.91
LCAT	P04180	end	14.56 (0.56) [10]	14.85 (0.31) [8]	0.61 (0.34-0.89)	14.37	1.00	0.30
LCN2	P80188	end	15.41 (1.3) [10]	14.65 (1.09) [8]	0.62 (0.35-0.9)	15.74	1.00	0.40
LCP1	P13796	start	14.59 (0.77) [7]	13.71 (0.35) [7]	0.9 (0.69-1)	14.08	1.00	0.86
LCP1	P13796	end	15.22 (1.25) [10]	15.39 (1.07) [10]	0.51 (0.24-0.78)	13.14	1.00	0.10
LCP1	P13796	change	-0.05 (0.07) [6]	-0.1 (0.1) [7]	0.67 (0.34-0.99)	-0.07	0.71	0.67
LDHA	P00338	change	-0.05 (0.05) [6]	-0.11 (0.1) [6]	0.69 (0.35-1)	-0.09	0.67	0.83
LDHA	P00338	start	14.37 (1.38) [8]	14.09 (0.86) [6]	0.52 (0.18-0.86)	13.23	1.00	0.38
LDHA	P00338	end	15.2 (0.92) [10]	15.25 (1.04) [10]	0.58 (0.31-0.85)	14.62	0.90	0.40
LDHB	P07195	change	-0.08 (0.12) [9]	0.02 (0.22) [9]	0.54 (0.25-0.83)	0.03	0.22	1.00
LDHB	P07195	start	14.59 (1.08) [11]	15.74 (3.99) [9]	0.53 (0.25-0.8)	14.63	0.56	0.64

Protein Name	Uniprot ID	Time	mRS 0-2 Mean (SD) [N]	mRS 3-6 Mean (SD) [N]	AUC (95%CI)	Cut-off	Sens.	Spec.
LDHB	P07195	end	15.26 (0.67) [10]	15.19 (0.58) [10]	0.52 (0.24-0.8)	15.56	0.90	0.40
LGALS1	P09382	end	15.08 (0.75) [10]	15.24 (1.05) [10]	0.55 (0.28-0.82)	15.36	0.40	0.80
LGALS3BP	Q08380	change	-0.04 (0.1) [10]	-0.06 (0.07) [9]	0.69 (0.42-0.96)	-0.03	0.78	0.70
LGALS3BP	Q08380	start	15.63 (0.67) [12]	15.29 (0.85) [9]	0.64 (0.38-0.9)	15.73	0.89	0.50
LGALS3BP	Q08380	end	15.99 (0.75) [10]	16.22 (0.69) [10]	0.52 (0.25-0.79)	15.43	1.00	0.20
LIAS	A0A1W2PNQ5	start	15.75 (1.24) [9]	15.81 (1.21) [8]	0.51 (0.22-0.81)	16.09	0.75	0.44
LIAS	A0A1W2PNQ5	change	0.08 (0.03) [6]	0.08 (0.09) [7]	0.52 (0.16-0.89)	0.04	0.43	1.00
LIAS	A0A1W2PNQ5	end	14.72 (1.31) [8]	14.7 (0.86) [9]	0.5 (0.2-0.8)	14.84	0.56	0.62
LMAN2	D6RBV2	change	-0.05 (0.03) [5]	-0.04 (0.12) [5]	0.56 (0.1-1)	-0.07	0.60	0.80
LMAN2	D6RBV2	start	13.98 (0.71) [6]	14.24 (1.66) [5]	0.5 (0.05-0.95)	15.21	0.40	1.00
LMAN2	D6RBV2	end	14.81 (0.36) [9]	14.78 (0.29) [10]	0.52 (0.23-0.81)	14.77	0.50	0.78
LRG1	P02750	change	0 (0.04) [10]	-0.01 (0.04) [9]	0.54 (0.27-0.82)	0.03	0.89	0.30
LRG1	P02750	start	16.89 (0.38) [12]	17.03 (0.42) [9]	0.63 (0.36-0.9)	17.14	0.56	0.83
LRG1	P02750	end	16.82 (0.41) [10]	17.17 (0.56) [10]	0.7 (0.46-0.94)	16.91	0.70	0.70
LRP1	Q07954	end	13.07 (0.95) [9]	12.71 (0.86) [7]	0.65 (0.35-0.95)	12.77	0.71	0.67
LRRC4B	Q9NT99	end	13.56 (0.61) [7]	13.19 (1.11) [8]	0.62 (0.32-0.93)	12.67	0.38	1.00
LSAMP	H3BLU2	end	14.25 (0.93) [10]	14.48 (0.67) [10]	0.53 (0.26-0.8)	14.93	0.40	0.80

Protein Name	Uniprot ID	Time	mRS 0-2<br>Mean (SD) [N]	mRS 3-6<br>Mean (SD) [N]	AUC (95%CI)	Cut-off	Sens.	Spec.
LTBP2	G3V3X5	end	13.13 (1.01) [9]	12.37 (0.9) [8]	0.75 (0.49-1)	13.15	0.88	0.67
LTF	E7EQB2	start	14.88 (1.25) [7]	13.94 (1.68) [6]	0.67 (0.32-1)	15.03	0.83	0.57
LTF	E7EQB2	end	14.22 (1.57) [10]	14.69 (1.07) [10]	0.64 (0.37-0.91)	14.14	0.80	0.70
LTF	E7EQB2	change	-0.05 (0.2) [6]	-0.09 (0.11) [6]	0.67 (0.31-1)	-0.04	0.83	0.67
LUM	P51884	change	0.04 (0.03) [11]	0.01 (0.03) [9]	0.76 (0.53-0.99)	0.03	0.89	0.64
LUM	P51884	end	15.65 (0.34) [11]	15.52 (0.33) [10]	0.59 (0.33-0.85)	15.93	1.00	0.27
LUM	P51884	start	16.24 (0.39) [12]	15.7 (0.51) [9]	0.74 (0.5-0.98)	15.96	0.67	0.83
LY6H	O94772	end	11.23 (0.73) [9]	11.15 (0.59) [8]	0.6 (0.31-0.89)	10.83	0.50	0.78
LYVE1	Q9Y5Y7	end	15.68 (1.39) [10]	15.81 (0.85) [10]	0.52 (0.24-0.8)	15.28	0.80	0.40
LYVE1	Q9Y5Y7	change	-0.06 (0.12) [9]	-0.13 (0.09) [8]	0.65 (0.37-0.94)	-0.08	0.88	0.56
LYVE1	Q9Y5Y7	start	14.39 (1.81) [11]	13.71 (1.04) [8]	0.53 (0.24-0.83)	15.39	1.00	0.45
LYZ	A0A0B4J259	start	16.04 (1.44) [12]	15.35 (1.23) [9]	0.63 (0.38-0.88)	17.52	1.00	0.25
LYZ	A0A0B4J259	end	17.42 (0.93) [10]	17.44 (0.81) [10]	0.52 (0.24-0.8)	16.91	0.90	0.40
LYZ	A0A0B4J259	change	-0.08 (0.08) [10]	-0.12 (0.09) [9]	0.7 (0.44-0.96)	-0.16	0.56	0.90
MAG	P20916	end	12.83 (0.92) [6]	12.65 (0.63) [6]	0.5 (0.13-0.87)	11.82	1.00	0.17
MAN1A1	P33908	end	14.06 (0.5) [10]	13.76 (0.28) [8]	0.75 (0.51-0.99)	13.77	0.62	0.90
MAN1C1	Q9NR34	end	13.01 (1.05) [8]	13.02 (0.57) [5]	0.52 (0.19-0.86)	13.75	1.00	0.25

Protein Name	Uniprot ID	Time	mRS 0-2 Mean (SD) [N]	mRS 3-6 Mean (SD) [N]	AUC (95%CI)	Cut-off	Sens.	Spec.
MAN2A2	P49641	end	14.04 (0.8) [8]	13.23 (0.51) [8]	0.81 (0.59-1)	13.42	0.75	0.88
MARCKS	P29966	start	11.79 (0.53) [6]	12.29 (1.03) [7]	0.62 (0.25-0.99)	12.72	0.57	1.00
MARCKS	P29966	end	12.5 (0.71) [10]	12.16 (0.67) [9]	0.63 (0.36-0.9)	12.17	0.56	0.80
MARCKS	P29966	change	-0.03 (0.06) [5]	0 (0.07) [6]	0.67 (0.3-1)	-0.04	0.83	0.60
MASP1	P48740	start	13.63 (0.41) [7]	13.81 (0.65) [6]	0.64 (0.28-1)	13.86	0.67	0.86
MBL2	P11226	start	13.8 (1.04) [7]	14.61 (0.93) [6]	0.71 (0.39-1)	14.49	0.67	0.86
MBL2	P11226	end	11.57 (1.45) [6]	10.37 (0.91) [5]	0.73 (0.38-1)	10.80	0.80	0.83
MCAM	P43121	end	14.44 (0.64) [9]	14.11 (0.84) [10]	0.59 (0.31-0.87)	14.68	0.80	0.56
MDH1	P40925	change	-0.03 (0.07) [6]	-0.05 (0.07) [5]	0.6 (0.22-0.98)	-0.01	0.80	0.50
MDH1	P40925	end	15.36 (0.76) [10]	15.71 (0.83) [10]	0.63 (0.36-0.9)	15.78	0.60	0.80
MDH1	P40925	start	15.27 (0.92) [7]	15.58 (0.85) [5]	0.6 (0.25-0.95)	14.44	1.00	0.29
MEGF8	Q7Z7M0	end	13.3 (0.86) [8]	13.27 (0.85) [7]	0.52 (0.19-0.84)	12.30	1.00	0.25
MGAT1	P26572	end	14.23 (0.31) [8]	14.11 (0.25) [8]	0.56 (0.26-0.87)	14.13	0.62	0.62
MGP	P08493	end	16.02 (0.71) [9]	15.8 (0.77) [9]	0.59 (0.3-0.89)	16.37	0.78	0.56
MIF	P14174	start	16.45 (1.41) [5]	15.85 (1.13) [6]	0.63 (0.24-1)	15.63	0.67	0.80
MIF	P14174	end	15.98 (0.93) [10]	16.41 (0.85) [9]	0.67 (0.39-0.94)	16.56	0.67	0.80
MMP2	P08253	end	15.32 (0.48) [9]	15 (0.55) [10]	0.71 (0.45-0.97)	15.41	0.70	0.78
MOG	A0A0G2JHA9	end	13.5 (0.65) [8]	13.03 (0.97) [10]	0.65 (0.36-0.94)	12.95	0.70	0.88

Protein Name	Uniprot ID	Time	mRS 0-2 Mean (SD) [N]	mRS 3-6 Mean (SD) [N]	AUC (95%CI)	Cut-off	Sens.	Spec.
MRC1	P22897	end	14.85 (0.68) [9]	14.77 (0.7) [9]	0.56 (0.27-0.85)	14.92	0.78	0.56
MSN	P26038	end	13.52 (1.24) [10]	13.59 (1.4) [9]	0.56 (0.27-0.84)	13.61	0.78	0.60
MST1	G3XAK1	start	14.13 (1.01) [5]	13.93 (1.15) [5]	0.52 (0.11-0.93)	13.22	0.40	0.80
MST1	G3XAK1	end	13.21 (0.56) [9]	13.17 (0.55) [6]	0.52 (0.18-0.86)	12.85	0.50	0.78
MYH11	P35749	end	15.9 (0.97) [7]	15.88 (0.59) [9]	0.54 (0.21-0.87)	14.90	1.00	0.29
MYH9	P35579	end	14.17 (2.34) [6]	12.84 (0.67) [8]	0.69 (0.38-1)	12.98	0.62	0.83
NBL1	A0A087WTY6	end	18.1 (0.35) [9]	17.96 (0.57) [10]	0.61 (0.34-0.88)	17.53	0.30	1.00
NCAM1	P13591	end	15.31 (1.09) [11]	15.3 (0.53) [10]	0.58 (0.32-0.85)	15.42	0.70	0.64
NCAM1	P13591	start	14.02 (1.37) [12]	14.23 (1.1) [7]	0.51 (0.23-0.8)	14.55	0.86	0.42
NCAM1	P13591	change	-0.07 (0.09) [11]	-0.08 (0.09) [7]	0.58 (0.3-0.87)	-0.05	0.86	0.45
NCAM2	H9KV31	end	14.39 (0.51) [9]	14.43 (0.59) [10]	0.54 (0.26-0.83)	15.10	0.30	1.00
NCAN	O14594	end	14.23 (0.63) [9]	14.15 (1) [9]	0.56 (0.27-0.84)	13.89	0.56	0.67
NEFM	E7EMV2	end	13.39 (1.69) [5]	14.1 (3.8) [6]	0.7 (0.32-1)	15.03	0.67	1.00
NEGR1	Q7Z3B1	end	13.75 (1.36) [9]	14.2 (1.37) [10]	0.59 (0.31-0.87)	13.70	0.70	0.67
NELL2	F8VVB6	end	14.57 (0.67) [10]	14.15 (1.06) [10]	0.65 (0.38-0.92)	13.73	0.50	0.90
NEO1	Q92859	end	14.18 (0.33) [8]	13.92 (0.78) [10]	0.61 (0.33-0.9)	13.62	0.40	1.00
NEO1	Q92859	start	13.84 (0.65) [6]	13.44 (1.28) [5]	0.77 (0.38-1)	13.20	0.80	0.83

Protein Name	Uniprot ID	Time	mRS 0-2 Mean (SD) [N]	mRS 3-6 Mean (SD) [N]	AUC (95%CI)	Cut-off	Sens.	Spec.
NFASC	O94856	end	13.63 (0.72) [8]	13.92 (0.6) [7]	0.62 (0.32-0.93)	14.17	0.43	0.88
NID1	P14543	end	13.87 (0.41) [9]	13.33 (0.56) [8]	0.79 (0.56-1)	13.63	0.75	0.78
NID2	Q14112	end	14.02 (0.9) [8]	13.99 (0.42) [7]	0.61 (0.29-0.92)	14.03	0.57	0.75
NPC2	E7EMS2	change	-0.05 (0.08) [7]	-0.08 (0.12) [6]	0.52 (0.16-0.88)	-0.03	0.83	0.43
NPC2	E7EMS2	end	16.17 (0.85) [10]	16.16 (0.89) [10]	0.52 (0.25-0.79)	15.81	0.50	0.70
NPC2	E7EMS2	start	15.5 (1.08) [9]	15.05 (1.2) [6]	0.65 (0.32-0.98)	15.50	0.83	0.67
NPDC1	Q5SPY9	end	13.1 (0.91) [10]	12.72 (0.91) [9]	0.61 (0.34-0.88)	12.70	0.56	0.70
NPPC	P23582	end	13.49 (0.61) [7]	13.48 (0.55) [7]	0.55 (0.22-0.88)	13.30	0.71	0.57
NPTX1	Q15818	change	0.02 (0.21) [7]	-0.04 (0.05) [5]	0.54 (0.19-0.9)	-0.09	1.00	0.29
NPTX1	Q15818	start	13.88 (1.66) [9]	13.47 (1.93) [6]	0.59 (0.27-0.92)	14.31	0.83	0.44
NPTX1	Q15818	end	14.32 (1.16) [10]	14.37 (1.17) [9]	0.51 (0.23-0.79)	14.34	0.67	0.50
NPTXR	A0A1X7SBT7	end	14.16 (1.11) [9]	14.57 (1.13) [9]	0.58 (0.3-0.86)	14.31	0.67	0.56
NRCAM	C9JYY6	change	-0.08 (0.07) [8]	-0.05 (0.08) [5]	0.75 (0.4-1)	-0.04	0.80	0.88
NRCAM	C9JYY6	start	14.04 (0.83) [10]	14.22 (1.52) [5]	0.5 (0.1-0.9)	14.06	0.60	0.70
NRCAM	C9JYY6	end	14.8 (0.87) [10]	14.87 (0.86) [10]	0.55 (0.27-0.83)	14.58	0.50	0.80
NRN1	A0A087WWT2	end	14.78 (0.47) [8]	14.5 (0.46) [7]	0.7 (0.41-0.98)	14.66	0.71	0.75
NRP1	E7EX60	end	13.28 (0.56) [9]	13.52 (0.39) [9]	0.69 (0.42-0.96)	13.13	0.89	0.56

Protein Name	Uniprot ID	Time	mRS 0-2 Mean (SD) [N]	mRS 3-6 Mean (SD) [N]	AUC (95%CI)	Cut-off	Sens.	Spec.
NRXN2	G5E9G7	end	14.45 (0.84) [9]	14.16 (0.96) [8]	0.6 (0.3-0.89)	14.95	0.88	0.44
NRXN3	A0A0U1RQC5	end	13.31 (0.99) [8]	13.4 (1.18) [9]	0.53 (0.22-0.84)	12.89	0.44	0.88
NSF	I3L0N3	end	12.7 (0.6) [6]	12.91 (0.38) [5]	0.63 (0.23-1)	12.40	1.00	0.50
NSG1	A0A0A6YYJ2	end	11.23 (0.46) [7]	10.19 (1.09) [7]	0.86 (0.58-1)	10.68	0.86	1.00
NTM	Q9P121-4	end	14.12 (0.88) [9]	14.4 (0.78) [10]	0.58 (0.3-0.85)	14.56	0.50	0.78
NUCB1	Q02818	change	-0.05 (0.08) [7]	-0.05 (0.09) [5]	0.51 (0.13-0.9)	-0.17	0.20	1.00
NUCB1	Q02818	start	13.2 (1.37) [10]	13.3 (0.54) [5]	0.62 (0.32-0.92)	13.95	1.00	0.50
NUCB1	Q02818	end	13.73 (0.59) [9]	13.73 (0.75) [10]	0.57 (0.28-0.85)	13.55	0.70	0.67
OAF	Q86UD1	end	14.65 (0.25) [8]	14.54 (0.61) [5]	0.62 (0.23-1)	14.43	0.40	1.00
OGN	P20774	end	15.62 (0.86) [10]	15.8 (0.55) [10]	0.52 (0.25-0.79)	16.04	0.80	0.40
OGN	P20774	change	-0.05 (0.09) [10]	-0.15 (0.13) [6]	0.7 (0.39-1)	-0.20	0.50	1.00
OGN	P20774	start	14.75 (1.56) [12]	13.58 (1.88) [6]	0.71 (0.42-1)	12.67	0.50	0.92
OMD	Q99983	end	13.32 (0.84) [6]	13.14 (1.03) [5]	0.5 (0.11-0.89)	12.69	0.80	0.50
OMG	P23515	end	14.63 (1.23) [7]	14.4 (1.17) [9]	0.54 (0.23-0.85)	14.12	0.44	0.86
ORM1	P02763	end	19.67 (0.49) [11]	19.56 (0.92) [10]	0.52 (0.24-0.79)	20.37	0.30	1.00
ORM1	P02763	change	-0.01 (0.02) [11]	0 (0.06) [10]	0.53 (0.25-0.81)	-0.01	0.70	0.64
ORM1	P02763	start	19.42 (0.35) [12]	19.52 (0.75) [10]	0.5 (0.22-0.78)	19.94	0.30	1.00
ORM2	P19652	end	18.15 (0.69) [11]	18.18 (0.64) [10]	0.51 (0.25-0.77)	18.39	0.60	0.55

Protein Name	Uniprot ID	Time	mRS 0-2 Mean (SD) [N]	mRS 3-6 Mean (SD) [N]	AUC (95%CI)	Cut-off	Sens.	Spec.
ORM2	P19652	change	0 (0.06) [11]	-0.02 (0.03) [10]	0.62 (0.36-0.88)	0.02	1.00	0.36
ORM2	P19652	start	17.93 (0.46) [12]	17.77 (0.41) [10]	0.62 (0.37-0.88)	17.88	0.70	0.67
P4HB	P07237	end	13.15 (0.96) [9]	13.36 (1.11) [9]	0.54 (0.26-0.83)	12.72	0.78	0.44
PAM	P19021	start	14.02 (0.75) [7]	13.87 (1.01) [5]	0.54 (0.15-0.93)	13.90	0.80	0.57
PAM	P19021	end	14.56 (0.57) [9]	14.21 (0.68) [9]	0.68 (0.4-0.96)	14.42	0.78	0.78
PAPLN	O95428	end	13.19 (0.52) [7]	13.14 (0.64) [6]	0.55 (0.16-0.94)	12.78	0.50	0.86
PARK7	Q99497	end	13.86 (0.95) [6]	13.88 (0.89) [9]	0.52 (0.19-0.85)	14.84	0.89	0.33
PCDHAC2	Q9Y5I4	end	14.32 (2.11) [6]	14.33 (1.33) [5]	0.53 (0.14-0.93)	12.78	1.00	0.33
PCOLCE	Q15113	change	-0.03 (0.09) [8]	-0.08 (0.09) [5]	0.72 (0.38-1)	-0.08	0.80	0.75
PCOLCE	Q15113	start	15.4 (1.2) [10]	14.49 (1.37) [5]	0.66 (0.28-1)	14.01	0.60	0.90
PCOLCE	Q15113	end	15.94 (1.01) [10]	16.08 (0.52) [10]	0.54 (0.27-0.81)	15.71	0.40	0.90
PCSK1N	Q9UHG2	end	15.22 (0.56) [10]	15 (0.69) [10]	0.64 (0.37-0.91)	15.09	0.70	0.70
PCSK1N	Q9UHG2	change	-0.08 (0.1) [10]	-0.1 (0.1) [8]	0.56 (0.28-0.85)	-0.06	0.75	0.50
PCSK1N	Q9UHG2	start	14.32 (1.07) [12]	13.47 (1.41) [8]	0.71 (0.44-0.97)	14.34	0.88	0.58
PCSK9	Q8NBP7	start	12.15 (0.59) [5]	12.22 (0.7) [5]	0.6 (0.19-1)	12.33	0.60	0.80
PDGFB	A9UJN9	end	13.3 (0.5) [5]	13.72 (0.79) [6]	0.7 (0.35-1)	14.01	0.50	1.00
PDIA3	P30101	end	14.11 (1.04) [8]	13.96 (1.08) [9]	0.56 (0.26-0.85)	13.54	0.56	0.75

Protein Name	Uniprot ID	Time	mRS 0-2 Mean (SD) [N]	mRS 3-6 Mean (SD) [N]	AUC (95%CI)	Cut-off	Sens.	Spec.
PDIA6	Q15084	end	13.48 (1.56) [7]	13.88 (1.03) [7]	0.51 (0.16-0.86)	14.12	0.86	0.43
PEA15	Q15121	end	14.24 (0.9) [9]	14.42 (0.52) [6]	0.56 (0.23-0.88)	14.35	0.67	0.67
PEBP1	P30086	start	15.5 (0.99) [10]	15.76 (1.13) [8]	0.58 (0.29-0.86)	15.61	0.62	0.60
PEBP1	P30086	end	15.61 (0.57) [10]	16.07 (0.77) [10]	0.68 (0.43-0.93)	16.12	0.60	0.80
PEBP1	P30086	change	-0.01 (0.08) [8]	-0.03 (0.06) [8]	0.55 (0.24-0.86)	0.06	1.00	0.25
PEBP4	Q96S96	end	15.35 (0.58) [9]	15.28 (0.3) [10]	0.5 (0.19-0.81)	14.86	1.00	0.33
PEBP4	Q96S96	start	15.04 (0.55) [6]	14.36 (1.55) [5]	0.8 (0.41-1)	14.03	0.80	1.00
PENK	P01210	end	14.33 (0.66) [10]	13.7 (1.46) [10]	0.65 (0.37-0.93)	13.72	0.60	0.90
PEPD	P12955	start	12.6 (0.46) [9]	12.63 (0.75) [7]	0.65 (0.32-0.98)	12.30	0.57	0.89
PEPD	P12955	end	13.17 (0.7) [10]	13.53 (0.53) [9]	0.73 (0.48-0.98)	13.18	0.78	0.80
PEPD	P12955	change	-0.03 (0.01) [7]	-0.05 (0.03) [6]	0.76 (0.46-1)	-0.04	0.67	0.86
PFN1	P07737	change	-0.02 (0.06) [8]	0 (0.13) [9]	0.54 (0.23-0.86)	0.00	0.67	0.75
PFN1	P07737	start	15.98 (0.48) [10]	16.15 (2.15) [9]	0.63 (0.35-0.92)	15.83	0.78	0.70
PFN1	P07737	end	16.04 (0.85) [10]	16.07 (0.85) [10]	0.57 (0.3-0.84)	16.23	0.50	0.80
PGAM1	P18669	end	15.02 (0.54) [10]	15.09 (0.89) [10]	0.59 (0.3-0.88)	15.30	0.60	0.80
PGAM1	P18669	start	14.83 (0.43) [5]	15 (1.35) [9]	0.51 (0.19-0.84)	15.33	0.44	1.00
PGD	P52209	end	14.98 (1.71) [5]	14.38 (0.97) [6]	0.7 (0.3-1)	15.49	1.00	0.60

Protein Name	Uniprot ID	Time	mRS 0-2 Mean (SD) [N]	mRS 3-6 Mean (SD) [N]	AUC (95%CI)	Cut-off	Sens.	Spec.
PGK1	P00558	end	15.5 (0.86) [10]	15.57 (1.24) [10]	0.56 (0.29-0.83)	16.32	0.40	0.90
PGK1	P00558	change	-0.03 (0.05) [7]	0.03 (0.28) [7]	0.55 (0.17-0.94)	-0.12	0.43	1.00
PGK1	P00558	start	15.24 (0.95) [9]	16.8 (4.45) [7]	0.56 (0.23-0.89)	16.28	0.43	0.89
PGLYRP2	Q96PD5	end	14.66 (0.88) [10]	14.47 (1.38) [10]	0.64 (0.37-0.91)	14.46	0.90	0.50
PGLYRP2	Q96PD5	change	0.07 (0.14) [10]	0.07 (0.09) [10]	0.63 (0.37-0.89)	0.05	0.80	0.50
PGLYRP2	Q96PD5	start	15.44 (0.69) [12]	15.72 (0.9) [10]	0.59 (0.33-0.85)	16.19	0.30	1.00
PI16	Q6UXB8	change	-0.02 (0.1) [8]	0 (0.08) [7]	0.55 (0.24-0.87)	-0.03	0.71	0.50
PI16	Q6UXB8	start	13.33 (0.5) [9]	12.83 (0.67) [8]	0.68 (0.4-0.96)	12.77	0.50	1.00
PI16	Q6UXB8	end	13.62 (1.29) [10]	12.71 (0.92) [9]	0.74 (0.5-0.98)	12.98	0.67	0.80
PIK3IP1	Q96FE7-4	end	14.58 (0.81) [9]	14.82 (0.68) [7]	0.57 (0.23-0.91)	15.29	0.43	1.00
PKM	P14618	start	14.58 (0.87) [8]	14.48 (1.03) [7]	0.57 (0.25-0.89)	14.92	0.86	0.38
PKM	P14618	change	-0.04 (0.08) [6]	-0.06 (0.08) [7]	0.57 (0.21-0.93)	-0.01	0.86	0.50
PKM	P14618	end	14.94 (0.45) [10]	15.15 (0.88) [10]	0.6 (0.31-0.89)	15.07	0.60	0.80
PLD3	Q8IV08	end	13.78 (0.91) [6]	14.19 (1.08) [7]	0.64 (0.31-0.97)	14.85	0.43	1.00
PLG	P00747	change	0.05 (0.14) [11]	0.09 (0.07) [10]	0.75 (0.51-0.98)	0.05	0.90	0.64
PLG	P00747	start	17.01 (0.76) [12]	17.59 (1.12) [10]	0.62 (0.38-0.87)	17.73	0.30	1.00

Protein Name	Uniprot ID	Time	mRS 0-2 Mean (SD) [N]	mRS 3-6 Mean (SD) [N]	AUC (95%CI)	Cut-off	Sens.	Spec.
PLG	P00747	end	16.37 (1.07) [11]	15.98 (0.44) [10]	0.58 (0.32-0.84)	16.47	1.00	0.36
PLTP	P55058	start	15.12 (1.09) [12]	14.88 (1.38) [9]	0.58 (0.31-0.85)	15.08	0.78	0.58
PLTP	P55058	change	-0.07 (0.14) [11]	-0.09 (0.1) [9]	0.61 (0.34-0.87)	-0.07	0.67	0.64
PLTP	P55058	end	16.11 (1.16) [11]	16.3 (0.69) [10]	0.52 (0.26-0.78)	15.22	1.00	0.18
PLXDC2	Q6UX71	change	-0.12 (0.17) [8]	-0.09 (0.09) [7]	0.55 (0.23-0.88)	-0.07	0.71	0.62
PLXDC2	Q6UX71	start	13.44 (1.16) [10]	13.48 (1.38) [7]	0.5 (0.19-0.81)	13.30	0.71	0.50
PLXDC2	Q6UX71	end	14.32 (1.19) [10]	14.62 (0.59) [10]	0.55 (0.28-0.82)	14.01	0.90	0.40
PMFBP1	G3V1Q7	end	16.34 (0.32) [9]	16.13 (0.42) [8]	0.69 (0.41-0.98)	16.41	0.88	0.56
PNP	P00491	end	13.71 (0.91) [5]	14.05 (1.01) [8]	0.6 (0.26-0.94)	14.28	0.50	0.80
PODXL2	Q9NZ53	end	12.2 (0.4) [5]	11.84 (1.1) [6]	0.57 (0.19-0.95)	11.46	0.33	1.00
POMGNT1	Q8WZA1	end	13.45 (0.79) [8]	14.03 (0.75) [9]	0.69 (0.42-0.97)	13.48	0.78	0.62
PON1	P27169	change	0.13 (0.18) [10]	0.12 (0.1) [9]	0.68 (0.4-0.95)	0.11	0.78	0.70
PON1	P27169	start	17.26 (1.28) [12]	17.63 (1.1) [9]	0.57 (0.31-0.84)	17.41	0.89	0.50
PON1	P27169	end	15.78 (1.14) [10]	15.54 (0.86) [10]	0.56 (0.29-0.83)	16.95	1.00	0.20
PON3	C9JZ99	start	14.11 (0.4) [6]	13.6 (1.42) [5]	0.6 (0.12-1)	13.34	0.60	1.00
POSTN	B1ALD9	end	13.98 (0.71) [7]	13.62 (0.47) [6]	0.6 (0.25-0.94)	13.81	0.83	0.57

**Protein Name**	**Uniprot ID**	**Time**	**mRS 0-2** Mean (SD) [N]	**mRS 3-6** Mean (SD) [N]	**AUC (95%CI)**	**Cut-off**	**Sens.**	**Spec.**
PPBP	P02775	start	18.58 (1.2) [10]	18.12 (1.66) [9]	0.61 (0.34-0.89)	18.67	0.67	0.70
PPIA	P62937	change	-0.05 (0.09) [11]	-0.03 (0.08) [9]	0.63 (0.35-0.9)	-0.01	0.56	0.82
PPIA	P62937	end	16.54 (0.95) [11]	16.09 (1.15) [10]	0.58 (0.32-0.84)	15.99	0.50	0.73
PPIA	P62937	start	15.74 (1.36) [12]	15.58 (1.07) [9]	0.56 (0.3-0.83)	16.49	0.78	0.50
PPIB	P23284	change	-0.02 (0.06) [7]	-0.03 (0.11) [6]	0.52 (0.17-0.88)	0.01	0.50	0.71
PPIB	P23284	end	14.29 (0.86) [10]	14.6 (1.02) [10]	0.59 (0.32-0.86)	14.34	0.60	0.70
PPIB	P23284	start	14.26 (0.87) [9]	14.4 (0.93) [6]	0.52 (0.15-0.89)	13.76	0.50	0.89
PPIC	P45877	end	14.48 (0.36) [7]	14.28 (0.56) [6]	0.64 (0.3-0.99)	14.30	0.67	0.71
PRCP	P42785	end	14.67 (0.58) [7]	14.91 (0.53) [7]	0.57 (0.24-0.91)	14.05	1.00	0.29
PRDX1	Q06830	end	17.28 (2.19) [11]	17.08 (1.66) [10]	0.55 (0.28-0.81)	16.92	0.70	0.55
PRDX1	Q06830	change	-0.07 (0.15) [10]	-0.02 (0.2) [10]	0.52 (0.25-0.79)	0.07	0.30	0.90
PRDX1	Q06830	start	16.25 (1.73) [11]	16.65 (2.97) [10]	0.53 (0.26-0.79)	17.51	0.90	0.36
PRDX2	P32119	change	-0.11 (0.19) [11]	-0.06 (0.24) [10]	0.55 (0.27-0.82)	-0.09	0.60	0.73
PRDX2	P32119	start	16.32 (2.15) [12]	16.92 (3.87) [10]	0.54 (0.28-0.8)	16.21	0.70	0.58
PRDX2	P32119	end	18.01 (1.84) [11]	17.97 (2.03) [10]	0.52 (0.25-0.78)	17.12	0.80	0.45
PRDX6	P30041	end	15.19 (1.38) [10]	15.37 (1.15) [10]	0.52 (0.24-0.8)	14.87	0.80	0.50

**Protein Name**	**Uniprot ID**	**Time**	**mRS 0-2** Mean (SD) [N]	**mRS 3-6** Mean (SD) [N]	**AUC (95%CI)**	**Cut-off**	**Sens.**	**Spec.**
PRDX6	P30041	change	0.02 (0.08) [8]	0.05 (0.14) [9]	0.57 (0.27-0.87)	0.15	0.33	1.00
PRDX6	P30041	start	15.54 (1.26) [10]	16.17 (1.73) [9]	0.59 (0.31-0.87)	15.35	0.89	0.50
PRELP	P51888	end	13.41 (0.64) [8]	13.18 (1.23) [8]	0.62 (0.3-0.95)	13.12	0.62	0.88
PRG4	A0A0U1RR20	change	0.02 (0.08) [9]	-0.01 (0.09) [7]	0.54 (0.22-0.86)	-0.07	0.29	1.00
PRG4	A0A0U1RR20	start	13.73 (0.93) [11]	13.41 (0.65) [9]	0.61 (0.34-0.87)	13.46	0.56	0.73
PRG4	A0A0U1RR20	end	13.63 (0.77) [10]	13.87 (1.31) [8]	0.52 (0.23-0.82)	14.85	0.25	1.00
PRNP	A2A2V1	end	13.35 (0.72) [8]	13.19 (0.53) [10]	0.59 (0.3-0.88)	13.09	0.60	0.75
PROC	E7END6	start	13.91 (0.49) [10]	13.99 (0.83) [9]	0.6 (0.32-0.88)	14.22	0.56	0.80
PROC	E7END6	change	0.01 (0.02) [8]	0.03 (0.06) [8]	0.67 (0.36-0.99)	0.03	0.62	0.88
PROC	E7END6	end	13.56 (0.39) [10]	13.52 (0.6) [9]	0.52 (0.24-0.8)	13.61	0.56	0.60
PROCR	Q9UNN8	change	-0.01 (0.07) [9]	-0.04 (0.05) [7]	0.71 (0.44-0.99)	-0.05	0.57	0.89
PROCR	Q9UNN8	start	14.24 (0.73) [11]	13.69 (0.59) [7]	0.77 (0.54-0.99)	14.25	0.86	0.64
PROCR	Q9UNN8	end	14.38 (0.68) [10]	14.23 (0.44) [10]	0.59 (0.31-0.87)	14.80	1.00	0.30
PROS1	P07225	end	15.26 (0.67) [11]	15.08 (0.52) [10]	0.59 (0.33-0.85)	14.76	0.40	0.91
PROS1	P07225	change	0.05 (0.08) [11]	0.03 (0.03) [9]	0.68 (0.42-0.94)	0.02	0.56	0.91
PROS1	P07225	start	15.99 (0.4) [12]	15.68 (0.56) [9]	0.72 (0.47-0.97)	15.54	0.56	0.92

Protein Name	Uniprot ID	Time	mRS 0-2 Mean (SD) [N]	mRS 3-6 Mean (SD) [N]	AUC (95%CI)	Cut-off	Sens.	Spec.
PROZ	P22891	start	14.91 (0.99) [10]	15.37 (0.89) [6]	0.63 (0.34-0.93)	14.19	1.00	0.40
PROZ	P22891	end	13.26 (0.45) [8]	13.64 (0.72) [9]	0.67 (0.38-0.95)	13.66	0.56	0.88
PROZ	P22891	change	0.08 (0.02) [6]	0.13 (0.09) [5]	0.8 (0.41-1)	0.10	0.80	1.00
PRSS3	B1AN99	start	17.93 (1.83) [7]	19.32 (1.54) [6]	0.74 (0.4-1)	18.76	0.83	0.86
PRSS3	B1AN99	end	19.58 (2.03) [8]	19.93 (1.27) [8]	0.53 (0.21-0.85)	20.90	0.88	0.38
PSAP	C9JIZ6	end	13.54 (1.37) [6]	14.33 (1.02) [6]	0.64 (0.29-0.98)	13.59	0.83	0.50
PSMA6	G3V5Z7	end	12.7 (1.43) [8]	13.41 (0.73) [8]	0.67 (0.34-1)	12.63	1.00	0.62
PTGDS	P41222	end	19.79 (1.01) [11]	19.92 (0.52) [10]	0.57 (0.3-0.84)	20.04	0.80	0.55
PTGDS	P41222	start	18.64 (1.36) [12]	17.5 (2.34) [10]	0.68 (0.44-0.92)	18.88	0.80	0.58
PTGDS	P41222	change	-0.06 (0.09) [11]	-0.14 (0.14) [10]	0.65 (0.41-0.9)	-0.20	0.30	1.00
PTPRD	P23468	end	13.88 (0.69) [9]	13.43 (0.75) [9]	0.65 (0.38-0.93)	13.84	0.78	0.56
PTPRG	P23470	end	14.05 (0.77) [8]	13.94 (0.85) [9]	0.56 (0.26-0.85)	13.74	0.56	0.75
PTPRG	P23470	start	13.74 (0.76) [7]	14.53 (1.04) [5]	0.66 (0.29-1)	13.43	1.00	0.43
PTPRN2	Q92932	end	15.09 (0.91) [7]	15.24 (0.24) [6]	0.55 (0.19-0.91)	15.52	1.00	0.43
PTPRS	Q13332	end	13.95 (0.69) [7]	13.75 (0.59) [9]	0.67 (0.36-0.98)	14.18	0.89	0.57
PTPRZ1	P23471	change	-0.01 (0.03) [5]	-0.05 (0.11) [5]	0.8 (0.41-1)	-0.05	0.80	1.00
PTPRZ1	P23471	start	13.69 (0.94) [8]	13.94 (1.3) [5]	0.52 (0.17-0.88)	12.66	1.00	0.25
PTPRZ1	P23471	end	14.11 (0.64) [9]	14.27 (0.83) [10]	0.56 (0.28-0.83)	14.92	0.30	1.00

**Protein Name**	**Uniprot ID**	**Time**	**mRS 0-2** Mean (SD) [N]	**mRS 3-6** Mean (SD) [N]	**AUC (95%CI)**	**Cut-off**	**Sens.**	**Spec.**
PVALB	B8ZZ19	end	13.92 (0.65) [8]	13.55 (0.47) [7]	0.75 (0.48-1)	14.14	1.00	0.50
PZP	P20742	start	14.49 (0.84) [10]	15.09 (0.6) [6]	0.7 (0.43-0.97)	14.59	0.83	0.60
PZP	P20742	end	12.98 (1.67) [7]	14.15 (0.84) [6]	0.69 (0.36-1)	14.17	0.67	0.86
QDPR	P09417	end	13.83 (0.61) [6]	13.79 (0.93) [7]	0.52 (0.17-0.87)	14.57	0.29	1.00
QPCT	Q16769	end	13.12 (0.46) [7]	12.98 (0.77) [9]	0.54 (0.23-0.84)	12.75	0.44	0.86
QSOX1	O00391	change	-0.01 (0.03) [8]	-0.03 (0.04) [7]	0.75 (0.45-1)	-0.05	0.57	1.00
QSOX1	O00391	end	14.33 (0.43) [10]	14.34 (0.37) [9]	0.5 (0.22-0.78)	14.48	0.44	0.70
QSOX1	O00391	start	14.19 (0.43) [10]	13.77 (0.34) [8]	0.76 (0.53-0.99)	13.88	0.62	0.80
RARRES2	Q99969	change	-0.02 (0.04) [6]	-0.03 (0.1) [5]	0.67 (0.25-1)	-0.05	0.80	0.83
RARRES2	Q99969	end	14.84 (0.69) [9]	14.55 (0.87) [10]	0.68 (0.39-0.96)	14.58	0.70	0.89
RARRES2	Q99969	start	14.61 (0.69) [8]	14.08 (1.41) [5]	0.73 (0.34-1)	14.46	0.80	0.75
RBP4	P02753	change	0 (0.06) [11]	0.02 (0.07) [10]	0.62 (0.36-0.87)	0.01	0.60	0.73
RBP4	P02753	end	16.44 (0.93) [11]	16.15 (0.69) [10]	0.62 (0.36-0.88)	16.40	0.80	0.55
RBP4	P02753	start	16.28 (0.67) [12]	16.57 (1.08) [10]	0.54 (0.29-0.8)	16.35	0.60	0.58
RGMB	J3KNF6	end	13.98 (0.76) [6]	13.41 (1.45) [7]	0.69 (0.34-1)	13.03	0.57	1.00
RNASE1	P07998	end	12.17 (1.06) [9]	12.03 (1) [10]	0.54 (0.26-0.83)	12.59	0.90	0.44
RNASE4	P34096	end	13.19 (0.92) [6]	13.11 (0.47) [5]	0.57 (0.18-0.95)	13.20	0.80	0.50
RNASE6	Q93091	end	14.09 (0.75) [9]	13.25 (0.75) [7]	0.79 (0.56-1)	13.84	0.86	0.67

Protein Name	Uniprot ID	Time	mRS 0-2 Mean (SD) [N]	mRS 3-6 Mean (SD) [N]	AUC (95%CI)	Cut-off	Sens.	Spec.
RNASET2	A0A087WZM2	end	14.06 (0.76) [9]	14.3 (0.97) [10]	0.56 (0.28-0.83)	14.64	0.40	0.89
RNH1	P13489	end	14.38 (0.92) [9]	14.74 (1.25) [6]	0.57 (0.24-0.91)	14.29	0.67	0.56
ROBO1	Q9Y6N7	end	13.26 (0.85) [7]	13.37 (0.7) [6]	0.55 (0.2-0.9)	13.08	0.83	0.43
S100A1	P23297	start	14.07 (1.27) [6]	13.23 (1) [7]	0.69 (0.35-1)	13.60	0.71	0.83
S100A8	P05109	change	-0.08 (0.19) [10]	-0.16 (0.18) [6]	0.67 (0.35-0.99)	-0.08	0.67	0.80
S100A8	P05109	end	16.62 (2.4) [10]	17.8 (1.61) [9]	0.63 (0.35-0.91)	16.31	0.89	0.60
S100A8	P05109	start	15.78 (2.13) [12]	15.92 (1.98) [7]	0.52 (0.24-0.81)	16.19	0.71	0.58
S100A9	P06702	end	15.87 (2.32) [10]	17.12 (1.28) [9]	0.67 (0.4-0.93)	15.73	0.89	0.60
S100A9	P06702	start	15.87 (1.15) [9]	17.04 (2.8) [7]	0.59 (0.28-0.9)	18.29	0.29	1.00
S100A9	P06702	change	-0.11 (0.23) [8]	-0.01 (0.25) [6]	0.58 (0.25-0.92)	0.00	0.50	0.75
S100B	P04271	start	15.97 (1.07) [10]	16.2 (1.39) [9]	0.54 (0.26-0.82)	17.58	0.22	1.00
S100B	P04271	end	13.82 (1.41) [9]	13.61 (0.79) [5]	0.51 (0.18-0.84)	13.08	1.00	0.44
SCG2	P13521	start	12.91 (0.53) [11]	12.68 (1.06) [6]	0.64 (0.28-1)	12.48	0.67	0.82
SCG2	P13521	end	13.39 (0.63) [10]	13.12 (0.95) [10]	0.64 (0.36-0.92)	12.88	0.60	0.90
SCG2	P13521	change	-0.04 (0.03) [9]	-0.04 (0.06) [6]	0.56 (0.22-0.89)	-0.02	0.50	0.78
SCG3	Q8WXD2	start	14.22 (1.1) [9]	13.83 (1.59) [6]	0.65 (0.32-0.98)	14.16	0.83	0.67
SCG3	Q8WXD2	end	15.15 (0.99) [10]	15.17 (0.85) [10]	0.54 (0.27-0.81)	14.72	0.40	0.80

Protein Name	Uniprot ID	Time	mRS 0-2 Mean (SD) [N]	mRS 3-6 Mean (SD) [N]	AUC (95%CI)	Cut-off	Sens.	Spec.
SCG3	Q8WXD2	change	-0.1 (0.06) [7]	-0.09 (0.09) [6]	0.52 (0.17-0.88)	-0.11	0.67	0.57
SCG5	P05408-2	start	14.82 (0.97) [9]	14.3 (1.32) [6]	0.63 (0.29-0.97)	14.77	0.83	0.67
SCG5	P05408-2	end	15.71 (1.07) [11]	15.34 (0.7) [10]	0.63 (0.35-0.9)	15.52	0.70	0.73
SCG5	P05408-2	change	-0.1 (0.18) [8]	-0.08 (0.09) [6]	0.56 (0.23-0.89)	-0.04	0.67	0.62
SCRG1	O75711	end	16.9 (0.52) [9]	17.05 (0.58) [10]	0.53 (0.25-0.81)	17.50	0.20	1.00
SDF4	Q9BRK5	end	13.14 (1.06) [6]	13.06 (0.57) [9]	0.61 (0.27-0.95)	13.58	0.89	0.50
SELENBP1	Q13228	change	-0.03 (0.04) [5]	0.04 (0.18) [5]	0.6 (0.19-1)	0.03	0.40	1.00
SELENBP1	Q13228	start	15.68 (1.1) [6]	15.97 (2.62) [5]	0.63 (0.24-1)	15.01	0.60	0.83
SELENBP1	Q13228	end	15.08 (1.09) [10]	15.08 (0.78) [10]	0.51 (0.23-0.79)	14.32	0.90	0.40
SELENOP	A0A182DWH7	start	15.84 (0.33) [9]	15.77 (0.51) [7]	0.54 (0.21-0.87)	15.27	0.29	1.00
SELL	P14151	change	0.03 (0.03) [8]	0.03 (0.04) [7]	0.57 (0.25-0.9)	0.02	0.57	0.75
SELL	P14151	start	16.07 (0.54) [10]	15.83 (0.68) [8]	0.61 (0.31-0.91)	15.56	0.50	0.90
SELL	P14151	end	15.48 (0.4) [10]	15.12 (0.48) [9]	0.72 (0.47-0.97)	15.36	0.78	0.70
SEMA4B	J3KNP4	end	12.99 (0.85) [7]	13.4 (0.29) [7]	0.71 (0.4-1)	13.11	1.00	0.57
SEMA7A	O75326	end	13.18 (0.9) [7]	12.94 (1.27) [8]	0.62 (0.32-0.93)	12.56	0.50	0.86
SERPINA1	P01009	change	0.01 (0.07) [11]	0.01 (0.04) [10]	0.51 (0.25-0.77)	-0.04	1.00	0.18
SERPINA1	P01009	start	18.76 (0.64) [12]	18.75 (0.68) [10]	0.68 (0.42-0.93)	18.86	0.90	0.67

**Protein Name**	**Uniprot ID**	**Time**	**mRS 0-2** Mean (SD) [N]	**mRS 3-6** Mean (SD) [N]	**AUC (95%CI)**	**Cut-off**	**Sens.**	**Spec.**
SERPINA1	P01009	end	18.73 (0.8) [11]	18.46 (0.48) [10]	0.53 (0.26-0.8)	18.85	0.90	0.36
SERPINA10	G3V2W1	change	0.06 (0.08) [7]	0.1 (0.09) [6]	0.62 (0.28-0.96)	0.05	0.83	0.57
SERPINA10	G3V2W1	start	13.74 (1.2) [9]	13.79 (0.83) [8]	0.53 (0.23-0.82)	12.50	1.00	0.22
SERPINA10	G3V2W1	end	12.52 (1.04) [9]	12.21 (1.03) [8]	0.6 (0.31-0.89)	11.45	0.38	0.89
SERPINA3	P01011	change	-0.02 (0.06) [11]	-0.02 (0.06) [10]	0.53 (0.26-0.79)	-0.07	0.30	0.91
SERPINA3	P01011	end	17.98 (0.94) [11]	17.83 (0.66) [10]	0.51 (0.24-0.77)	18.32	0.90	0.27
SERPINA3	P01011	start	17.64 (0.5) [12]	17.45 (0.95) [10]	0.68 (0.42-0.93)	17.20	0.60	0.83
SERPINA4	P29622	change	0.06 (0.07) [10]	0.04 (0.02) [9]	0.53 (0.25-0.81)	0.07	1.00	0.30
SERPINA4	P29622	end	14.81 (0.39) [10]	14.96 (0.49) [10]	0.59 (0.32-0.86)	15.13	0.50	0.80
SERPINA4	P29622	start	15.67 (0.46) [12]	15.66 (0.48) [9]	0.52 (0.25-0.79)	15.92	0.56	0.67
SERPINA5	P05154	end	14.1 (0.5) [9]	14.35 (0.76) [10]	0.64 (0.38-0.91)	14.59	0.50	0.89
SERPINA5	P05154	change	0.02 (0.05) [9]	0.02 (0.04) [9]	0.52 (0.22-0.82)	0.01	0.78	0.56
SERPINA5	P05154	start	14.47 (0.61) [11]	14.7 (0.42) [9]	0.62 (0.35-0.88)	14.58	0.78	0.64
SERPINA6	P08185	start	15.96 (0.56) [12]	15.99 (0.89) [10]	0.58 (0.32-0.83)	15.86	0.50	0.75
SERPINA6	P08185	change	0.03 (0.06) [11]	0.04 (0.05) [10]	0.51 (0.24-0.77)	0.02	0.70	0.55
SERPINA6	P08185	end	15.51 (0.83) [11]	15.26 (0.4) [10]	0.56 (0.3-0.82)	15.54	0.90	0.36

**Protein Name**	**Uniprot ID**	**Time**	**mRS 0-2** Mean (SD) [N]	**mRS 3-6** Mean (SD) [N]	**AUC (95%CI)**	**Cut-off**	**Sens.**	**Spec.**
SERPINA7	P05543	change	0.02 (0.08) [11]	0 (0.03) [9]	0.54 (0.26-0.81)	0.04	1.00	0.27
SERPINA7	P05543	end	14.93 (0.63) [11]	14.7 (0.48) [10]	0.6 (0.34-0.86)	14.58	0.50	0.73
SERPINA7	P05543	start	15 (0.5) [12]	14.77 (0.53) [9]	0.63 (0.38-0.88)	15.08	0.78	0.58
SERPINB1	P30740	end	14.01 (0.73) [9]	13.94 (1.25) [8]	0.65 (0.36-0.94)	13.46	0.50	0.89
SERPINC1	P01008	change	0 (0.06) [11]	0 (0.06) [10]	0.52 (0.25-0.78)	0.00	0.80	0.45
SERPINC1	P01008	end	17.02 (0.86) [11]	17.01 (0.31) [10]	0.62 (0.36-0.88)	16.84	0.80	0.55
SERPINC1	P01008	start	16.97 (0.42) [12]	17.02 (0.86) [10]	0.62 (0.36-0.87)	16.84	0.70	0.67
SERPIND1	P05546	change	0.05 (0.1) [11]	0.06 (0.03) [10]	0.68 (0.43-0.93)	0.04	0.80	0.73
SERPIND1	P05546	end	15.34 (0.7) [11]	15.19 (0.25) [10]	0.55 (0.28-0.81)	15.60	1.00	0.27
SERPIND1	P05546	start	16.03 (0.58) [12]	16.28 (0.64) [10]	0.6 (0.34-0.86)	16.13	0.80	0.58
SERPINF1	P36955	start	16.7 (0.96) [12]	16.42 (1.17) [10]	0.59 (0.34-0.84)	17.44	0.90	0.33
SERPINF1	P36955	end	17.11 (0.68) [11]	17.26 (0.84) [10]	0.51 (0.24-0.78)	16.64	0.40	0.82
SERPINF1	P36955	change	-0.03 (0.06) [11]	-0.05 (0.09) [10]	0.61 (0.34-0.88)	-0.08	0.50	0.82
SERPINF2	P08697	change	0.03 (0.06) [11]	0.04 (0.05) [10]	0.58 (0.31-0.85)	-0.01	1.00	0.36
SERPINF2	P08697	start	16.72 (0.39) [12]	16.85 (0.79) [10]	0.52 (0.26-0.78)	16.29	0.90	0.25
SERPINF2	P08697	end	16.36 (0.57) [11]	16.12 (0.3) [10]	0.68 (0.44-0.93)	16.07	0.70	0.73

**Protein Name**	**Uniprot ID**	**Time**	**mRS 0-2** Mean (SD) [N]	**mRS 3-6** Mean (SD) [N]	**AUC (95%CI)**	**Cut-off**	**Sens.**	**Spec.**
SERPING1	P05155	change	0.01 (0.03) [11]	0.02 (0.02) [10]	0.55 (0.28-0.81)	-0.01	1.00	0.27
SERPING1	P05155	start	16.98 (0.3) [12]	17.08 (0.36) [10]	0.6 (0.34-0.86)	17.24	0.40	0.92
SERPING1	P05155	end	16.73 (0.27) [11]	16.72 (0.3) [10]	0.55 (0.28-0.81)	16.62	0.80	0.45
SERPINI1	Q99574	end	13.47 (0.77) [5]	13.42 (1.49) [5]	0.52 (0.07-0.97)	13.91	0.60	0.80
SEZ6	Q53EL9	end	13.49 (0.66) [8]	13.15 (0.5) [7]	0.71 (0.42-1)	13.52	1.00	0.62
SEZ6L	B0QYH4	end	13.56 (0.44) [8]	13.2 (1.07) [9]	0.62 (0.32-0.93)	12.98	0.44	1.00
SEZ6L2	A0A087WYL5	end	14.8 (0.44) [8]	14.58 (0.65) [10]	0.6 (0.32-0.88)	14.79	0.70	0.62
SH3BGRL	O75368	end	13.46 (1.06) [8]	13.79 (1.07) [9]	0.65 (0.35-0.95)	13.19	0.89	0.62
SH3BGRL3	Q5T123	end	14.48 (1.08) [9]	14.83 (1.26) [8]	0.62 (0.33-0.92)	14.51	0.75	0.67
SHBG	I3L145	start	14.63 (1.39) [10]	14.96 (1.14) [8]	0.59 (0.3-0.87)	14.15	0.88	0.50
SHBG	I3L145	end	13.68 (0.46) [8]	13.67 (0.61) [9]	0.61 (0.29-0.93)	13.56	0.78	0.75
SHBG	I3L145	change	0.07 (0.05) [6]	0.07 (0.1) [7]	0.52 (0.17-0.87)	0.14	0.29	1.00
SHISA6	Q6ZSJ9	end	13.81 (0.34) [6]	13.48 (0.54) [7]	0.71 (0.4-1)	13.61	0.71	0.83
SIAE	Q9HAT2	end	13.76 (0.76) [5]	14.78 (0.94) [5]	0.8 (0.5-1)	14.92	0.60	1.00
SIRPA	P78324	end	14.16 (0.64) [7]	13.91 (0.98) [10]	0.53 (0.22-0.83)	12.99	0.30	1.00
SKP1	E5RJR5	start	13.9 (0.56) [8]	14.17 (0.67) [7]	0.62 (0.31-0.94)	13.92	0.71	0.62
SKP1	E5RJR5	change	0 (0.06) [6]	-0.02 (0.03) [6]	0.69 (0.31-1)	0.02	1.00	0.67

Protein Name	Uniprot ID	Time	mRS 0-2 Mean (SD) [N]	mRS 3-6 Mean (SD) [N]	AUC (95%CI)	Cut-off	Sens.	Spec.
SKP1	E5RJR5	end	13.83 (0.87) [9]	14.46 (0.66) [9]	0.7 (0.43-0.97)	14.07	0.78	0.67
SLC3A2	F5GZS6	end	12.74 (0.26) [8]	12.93 (0.52) [8]	0.64 (0.32-0.96)	13.07	0.50	1.00
SLC4A1	P02730	end	14.14 (0.81) [7]	13.69 (1.14) [7]	0.63 (0.31-0.95)	14.51	0.86	0.43
SNCA	E7EPV7	end	13.78 (1.49) [5]	12.58 (1.22) [8]	0.75 (0.46-1)	12.39	0.50	1.00
SOD1	P00441	end	15.89 (0.53) [10]	15.97 (0.33) [10]	0.56 (0.29-0.83)	16.01	0.50	0.80
SOD1	P00441	start	15.25 (0.7) [9]	15.73 (2.65) [8]	0.54 (0.22-0.86)	14.71	0.50	0.89
SOD1	P00441	change	-0.07 (0.1) [7]	-0.03 (0.16) [8]	0.52 (0.2-0.84)	-0.07	0.50	0.71
SOD2	P04179	end	13.68 (0.59) [10]	14.04 (0.92) [10]	0.61 (0.34-0.88)	14.05	0.50	0.90
SOD3	P08294	end	15.09 (0.69) [10]	15.58 (1.01) [10]	0.59 (0.33-0.85)	14.78	0.80	0.40
SOD3	P08294	start	14.87 (0.9) [12]	14.28 (1.38) [9]	0.67 (0.42-0.91)	14.69	0.67	0.75
SOD3	P08294	change	-0.03 (0.09) [10]	-0.09 (0.1) [9]	0.66 (0.39-0.93)	-0.03	0.67	0.80
SORCS3	Q9UPU3	end	12.7 (0.83) [8]	12.28 (0.73) [7]	0.62 (0.32-0.93)	13.06	0.86	0.50
SPARC	P09486	change	-0.04 (0.08) [11]	-0.06 (0.09) [9]	0.59 (0.32-0.85)	-0.03	0.78	0.45
SPARC	P09486	start	14.43 (0.77) [12]	15.01 (0.76) [9]	0.71 (0.48-0.94)	14.21	1.00	0.42
SPARC	P09486	end	15.37 (1.18) [11]	15.93 (0.7) [10]	0.64 (0.39-0.89)	15.99	0.60	0.73
SPARCL1	Q14515	change	-0.06 (0.07) [10]	-0.05 (0.07) [8]	0.58 (0.28-0.87)	-0.04	0.50	0.80

Protein Name	Uniprot ID	Time	mRS 0-2 Mean (SD) [N]	mRS 3-6 Mean (SD) [N]	AUC (95%CI)	Cut-off	Sens.	Spec.
SPARCL1	Q14515	start	13.44 (0.93) [12]	13.63 (0.84) [8]	0.51 (0.22-0.8)	13.77	0.50	0.75
SPARCL1	Q14515	end	14.38 (0.73) [10]	14.4 (0.58) [10]	0.59 (0.32-0.86)	14.00	0.40	0.90
SPOCK1	Q08629	end	13.14 (0.77) [7]	12.64 (0.95) [7]	0.69 (0.39-1)	12.10	0.43	1.00
SPOCK3	Q9BQ16	end	14.05 (0.29) [7]	13.71 (0.99) [8]	0.7 (0.38-1)	13.53	0.62	1.00
SPON1	Q9HCB6	end	13.51 (0.85) [7]	13.67 (0.77) [7]	0.53 (0.17-0.89)	12.90	1.00	0.43
SPP1	P10451	change	-0.09 (0.13) [10]	-0.11 (0.19) [10]	0.53 (0.26-0.8)	-0.04	0.80	0.40
SPP1	P10451	start	14.52 (1.43) [12]	14.62 (3.3) [10]	0.52 (0.25-0.8)	14.62	0.70	0.58
SPP1	P10451	end	15.9 (1.33) [10]	16.39 (0.8) [10]	0.61 (0.35-0.87)	16.81	0.40	0.90
STAB1	Q9NY15	end	12.73 (0.69) [7]	12.36 (0.35) [5]	0.74 (0.41-1)	12.63	1.00	0.71
SULF2	Q8IWU5	end	14.4 (0.28) [9]	14.06 (0.6) [10]	0.73 (0.47-0.99)	13.94	0.50	1.00
SUSD5	O60279	end	12.66 (0.39) [8]	12.44 (0.69) [9]	0.65 (0.36-0.95)	12.38	0.67	0.75
SYNE3	G3V533	start	15.71 (0.91) [9]	15.63 (0.9) [5]	0.51 (0.16-0.87)	16.74	1.00	0.22
SYNE3	G3V533	end	14.99 (0.7) [9]	14.77 (0.55) [5]	0.64 (0.33-0.95)	15.15	1.00	0.56
SYT2	Q8N9I0	end	17.81 (1.2) [8]	18.3 (0.73) [7]	0.59 (0.27-0.9)	17.50	1.00	0.38
SAA1	P0DJI8	end	15.44 (1.39) [6]	15.49 (1.41) [9]	0.52 (0.19-0.85)	15.10	0.56	0.83
SAA1	P0DJI8	start	16.58 (1.23) [10]	15.99 (0.9) [8]	0.62 (0.35-0.9)	16.13	0.75	0.60
SAA4	P35542	change	0.08 (0.14) [10]	0.07 (0.09) [9]	0.62 (0.35-0.89)	0.04	0.89	0.40

Protein Name	Uniprot ID	Time	mRS 0-2 Mean (SD) [N]	mRS 3-6 Mean (SD) [N]	AUC (95%CI)	Cut-off	Sens.	Spec.
SAA4	P35542	end	15.61 (0.68) [10]	15.67 (0.53) [10]	0.6 (0.33-0.87)	15.42	0.80	0.50
SAA4	P35542	start	16.55 (0.98) [12]	16.87 (1.3) [9]	0.64 (0.38-0.9)	16.67	0.78	0.50
TAGLN	Q01995	end	14.88 (0.46) [10]	14.45 (0.74) [9]	0.63 (0.35-0.91)	14.29	0.44	1.00
TAGLN2	P37802	start	14.32 (1.24) [9]	15.12 (0.48) [6]	0.7 (0.42-0.99)	14.26	1.00	0.56
TALDO1	P37837	start	14.78 (0.89) [6]	16.32 (2.45) [5]	0.67 (0.25-1)	16.29	0.60	1.00
TALDO1	P37837	change	-0.03 (0.06) [5]	0.06 (0.24) [5]	0.64 (0.2-1)	0.10	0.60	1.00
TALDO1	P37837	end	14.76 (1.29) [10]	14.94 (1.06) [9]	0.54 (0.25-0.83)	15.40	0.89	0.40
TCN2	B5MBX2	end	15.66 (1.86) [6]	14.74 (0.93) [8]	0.67 (0.34-0.99)	14.89	0.62	0.83
TF	P02787	start	17.97 (0.4) [12]	17.96 (0.59) [10]	0.59 (0.34-0.85)	18.10	0.80	0.50
TF	P02787	end	17.79 (0.98) [11]	17.72 (0.36) [10]	0.63 (0.37-0.88)	17.52	0.80	0.55
TF	P02787	change	0.02 (0.08) [11]	0.01 (0.04) [10]	0.6 (0.34-0.86)	0.02	0.80	0.45
TFRC	G3V0E5	start	12.16 (0.75) [6]	12.35 (0.71) [6]	0.58 (0.22-0.95)	12.03	0.83	0.50
TGFBI	Q15582	end	15.72 (1.52) [11]	15.35 (0.65) [10]	0.55 (0.29-0.82)	16.23	1.00	0.27
TGFBI	Q15582	start	14.79 (0.89) [12]	14.81 (2.29) [10]	0.66 (0.41-0.9)	14.95	0.90	0.50
TGFBI	Q15582	change	-0.06 (0.1) [11]	-0.03 (0.13) [10]	0.54 (0.27-0.8)	-0.03	0.80	0.45
TGOLN2	F8W8W7	end	12.21 (1.48) [10]	11.15 (1.09) [9]	0.76 (0.5-1)	11.79	0.89	0.70
TGOLN2	F8W8W7	start	10.41 (0.94) [6]	10.4 (1.31) [5]	0.57 (0.14-0.99)	9.87	0.60	0.83

**Protein Name**	**Uniprot ID**	**Time**	**mRS 0-2** Mean (SD) [N]	**mRS 3-6** Mean (SD) [N]	**AUC (95%CI)**	**Cut-off**	**Sens.**	**Spec.**
THBS1	P07996	start	15.35 (0.5) [11]	15.46 (0.68) [7]	0.6 (0.27-0.92)	15.63	0.71	0.73
THBS1	P07996	end	14.33 (0.67) [9]	14.59 (0.54) [6]	0.63 (0.33-0.93)	14.37	0.83	0.56
THBS2	P35442	start	14.67 (0.55) [7]	15.58 (1.22) [6]	0.67 (0.32-1)	15.82	0.50	1.00
THBS2	P35442	end	13.75 (0.35) [9]	13.86 (0.31) [5]	0.53 (0.12-0.94)	14.07	0.40	1.00
THY1	E9PIM6	end	16.01 (0.91) [10]	16.1 (0.8) [10]	0.54 (0.27-0.81)	15.88	0.50	0.80
THY1	E9PIM6	start	14.86 (0.98) [11]	15.12 (1.54) [7]	0.52 (0.2-0.84)	15.24	0.57	0.73
THY1	E9PIM6	change	-0.08 (0.08) [9]	-0.05 (0.07) [7]	0.51 (0.19-0.82)	-0.12	1.00	0.22
TIMP1	P01033	end	18.48 (2.01) [11]	18.44 (1.56) [10]	0.53 (0.26-0.79)	20.19	1.00	0.27
TIMP1	P01033	change	-0.04 (0.21) [10]	-0.14 (0.13) [6]	0.65 (0.37-0.93)	-0.10	0.83	0.60
TIMP1	P01033	start	17.68 (2.68) [11]	16.16 (1.85) [6]	0.68 (0.41-0.95)	18.53	1.00	0.55
TIMP2	P16035	end	15.61 (0.67) [8]	15.57 (0.82) [8]	0.53 (0.21-0.85)	15.85	0.88	0.50
TKT	P29401	end	14.4 (1.18) [10]	15.07 (1.18) [9]	0.69 (0.42-0.96)	14.99	0.78	0.80
TLN1	Q9Y490	start	15.32 (1.5) [5]	14.11 (1.28) [5]	0.68 (0.3-1)	15.90	1.00	0.40
TMEM189-UBE2V1	I3L0A0	end	15.1 (1.15) [5]	14.05 (0.83) [5]	0.8 (0.5-1)	13.78	0.60	1.00
TNC	P24821	end	12.74 (0.58) [6]	13.31 (0.87) [5]	0.77 (0.46-1)	12.42	1.00	0.50
TNXB	A0A140TA41	end	13.47 (0.61) [10]	13.37 (0.46) [9]	0.59 (0.3-0.88)	13.62	0.78	0.60
TPI1	P60174	start	14.2 (1.07) [11]	14.85 (1.61) [9]	0.62 (0.36-0.88)	13.72	0.89	0.36

Protein Name	Uniprot ID	Time	mRS 0-2 Mean (SD) [N]	mRS 3-6 Mean (SD) [N]	AUC (95%CI)	Cut-off	Sens.	Spec.
TPI1	P60174	end	14.52 (1.02) [10]	14.5 (1.35) [10]	0.5 (0.23-0.77)	14.47	0.60	0.60
TPI1	P60174	change	-0.05 (0.14) [9]	0 (0.15) [9]	0.62 (0.32-0.91)	-0.01	0.67	0.78
TPM4	P67936	end	14.4 (1.19) [7]	13.13 (3.73) [5]	0.69 (0.3-1)	14.05	0.80	0.71
TPM4	P67936	start	15.21 (0.78) [9]	14.76 (0.5) [5]	0.71 (0.41-1)	14.75	0.60	0.89
TPP1	O14773	start	14.39 (0.49) [7]	14.36 (0.3) [5]	0.54 (0.17-0.91)	14.47	0.80	0.57
TPP1	O14773	change	0.05 (0.03) [5]	-0.01 (0.1) [5]	0.64 (0.2-1)	0.00	0.60	1.00
TPP1	O14773	end	13.87 (0.65) [9]	14.2 (1.18) [9]	0.57 (0.28-0.85)	14.36	0.44	0.78
TPP2	P29144	start	15.07 (1.32) [10]	15.16 (0.96) [9]	0.51 (0.23-0.79)	15.37	0.44	0.70
TPP2	P29144	end	14.8 (1.33) [10]	14.66 (0.76) [9]	0.51 (0.21-0.81)	14.14	0.89	0.40
TPP2	P29144	change	0.01 (0.06) [8]	0.04 (0.07) [8]	0.67 (0.38-0.96)	0.07	0.50	1.00
TRBV7-2	A0A1B0GXF2	end	14.86 (0.65) [7]	14.78 (0.39) [9]	0.59 (0.25-0.92)	15.23	1.00	0.43
TREM2	Q9NZC2	end	14.23 (0.63) [9]	14.27 (0.67) [9]	0.51 (0.22-0.79)	14.79	0.89	0.22
TREM2	Q9NZC2	change	-0.03 (0.06) [5]	-0.07 (0.06) [5]	0.68 (0.3-1)	-0.08	0.60	0.80
TREM2	Q9NZC2	start	13.27 (0.97) [7]	13.37 (0.7) [5]	0.51 (0.15-0.88)	12.44	1.00	0.29
TTR	P02766	end	19.14 (0.84) [11]	19.13 (0.81) [10]	0.53 (0.26-0.8)	19.51	0.80	0.45
TTR	P02766	change	-0.02 (0.05) [11]	-0.04 (0.08) [10]	0.56 (0.29-0.84)	-0.05	0.50	0.82
TTR	P02766	start	18.56 (0.72) [12]	18.5 (0.77) [10]	0.5 (0.23-0.77)	18.11	0.80	0.50

Protein Name	Uniprot ID	Time	mRS 0-2 Mean (SD) [N]	mRS 3-6 Mean (SD) [N]	AUC (95%CI)	Cut-off	Sens.	Spec.
TUBA1B	P68363	change	0.13 (0.2) [9]	0.09 (0.12) [8]	0.53 (0.23-0.83)	0.31	1.00	0.22
TUBA1B	P68363	end	15.08 (1.45) [10]	14.78 (1.82) [9]	0.6 (0.32-0.88)	13.05	0.33	1.00
TUBA1B	P68363	start	16.37 (1.74) [11]	16.39 (1.07) [9]	0.53 (0.26-0.8)	16.35	0.56	0.64
TUBB4B	P68371	end	15.01 (1.28) [9]	15.26 (1.27) [6]	0.57 (0.25-0.9)	15.08	0.67	0.56
TUBB4B	P68371	start	16.01 (1.46) [12]	16.04 (1.13) [9]	0.52 (0.25-0.79)	15.62	0.44	0.75
TUBB4B	P68371	change	0.12 (0.2) [9]	0.04 (0.14) [5]	0.58 (0.23-0.92)	0.10	0.80	0.44
TXN	P10599	end	17.07 (0.76) [10]	17.13 (0.73) [10]	0.63 (0.35-0.91)	16.82	0.90	0.60
TXN	P10599	start	16.77 (1.09) [6]	15.76 (0.85) [7]	0.74 (0.44-1)	16.52	0.86	0.67
TXN	P10599	change	-0.11 (0.18) [5]	-0.09 (0.07) [7]	0.6 (0.2-1)	0.00	1.00	0.40
UBC	F5H265	end	15.94 (0.63) [10]	16.2 (0.7) [10]	0.64 (0.38-0.9)	16.32	0.50	0.80
UBC	F5H265	change	-0.08 (0.09) [9]	-0.09 (0.1) [7]	0.54 (0.21-0.87)	-0.13	0.43	0.89
UBC	F5H265	start	14.89 (1.29) [11]	14.83 (0.89) [7]	0.52 (0.24-0.8)	15.51	0.86	0.36
VASN	Q6EMK4	end	14.39 (0.3) [10]	14.35 (0.25) [9]	0.56 (0.28-0.83)	14.22	0.33	0.90
VASN	Q6EMK4	change	-0.01 (0.02) [8]	0 (0.02) [5]	0.6 (0.25-0.95)	-0.01	0.80	0.50
VASN	Q6EMK4	start	14.25 (0.48) [9]	14.37 (0.42) [6]	0.54 (0.19-0.88)	14.47	0.50	0.78
VCAM1	P19320	end	14.16 (0.71) [9]	13.74 (0.37) [8]	0.74 (0.48-1)	13.81	0.62	0.89
VCAN	P13611	end	14.4 (0.66) [9]	14.37 (0.78) [10]	0.54 (0.26-0.83)	14.42	0.60	0.78

Protein Name	Uniprot ID	Time	mRS 0-2 Mean (SD) [N]	mRS 3-6 Mean (SD) [N]	AUC (95%CI)	Cut-off	Sens.	Spec.
VGF	O15240	change	-0.07 (0.07) [7]	-0.05 (0.09) [5]	0.63 (0.25-1)	-0.06	0.80	0.57
VGF	O15240	start	13.24 (0.86) [9]	13.45 (1.08) [5]	0.53 (0.17-0.9)	14.88	0.20	1.00
VGF	O15240	end	14.15 (0.71) [10]	13.63 (1.32) [10]	0.65 (0.36-0.94)	13.41	0.70	0.90
VIM	P08670	start	14.86 (1.58) [10]	15.07 (1.41) [8]	0.5 (0.21-0.79)	13.87	0.88	0.30
VIM	P08670	end	16.65 (1.32) [10]	16.37 (2.12) [10]	0.55 (0.28-0.82)	17.51	0.80	0.40
VIM	P08670	change	-0.16 (0.2) [8]	-0.1 (0.2) [8]	0.58 (0.27-0.88)	-0.10	0.62	0.62
VIP	P01282	end	18.31 (0.52) [10]	18.25 (0.65) [10]	0.5 (0.23-0.77)	18.23	0.70	0.50
VIP	P01282	start	18.47 (0.63) [11]	18.23 (0.62) [9]	0.62 (0.35-0.88)	18.41	0.67	0.64
VIP	P01282	change	0 (0.04) [9]	0 (0.04) [9]	0.52 (0.23-0.81)	-0.01	0.67	0.56
VSIG4	Q9Y279	end	16 (0.74) [8]	15.56 (1.24) [9]	0.62 (0.33-0.92)	15.42	0.56	0.88
VSTM2A	B5MCX6	end	13.97 (1) [8]	13.55 (1.55) [8]	0.55 (0.24-0.85)	15.26	1.00	0.12
VSTM2B	A6NLU5	end	13.39 (0.68) [6]	12.42 (0.53) [8]	0.88 (0.62-1)	13.16	1.00	0.83
VTN	P04004	change	0.04 (0.07) [10]	0.06 (0.05) [9]	0.59 (0.32-0.86)	-0.02	1.00	0.30
VTN	P04004	end	17.43 (0.58) [10]	17.12 (0.35) [10]	0.64 (0.38-0.9)	17.66	1.00	0.40
VTN	P04004	start	18.14 (0.88) [12]	18.17 (0.84) [9]	0.52 (0.25-0.79)	18.81	0.33	0.83
VWF	P04275	start	14.53 (1.32) [7]	14.38 (0.83) [9]	0.52 (0.2-0.85)	15.51	1.00	0.29
WDR1	O75083	end	14.1 (1.31) [7]	14.44 (1.57) [7]	0.51 (0.16-0.86)	14.18	0.71	0.57

Protein Name	Uniprot ID	Time	mRS 0-2 Mean (SD) [N]	mRS 3-6 Mean (SD) [N]	AUC (95%CI)	Cut-off	Sens.	Spec.
WFIKKN2	C9J6G4	start	14.67 (0.97) [8]	15.05 (1.04) [6]	0.58 (0.25-0.92)	14.55	0.67	0.62
WFIKKN2	C9J6G4	change	0.01 (0.03) [6]	0 (0.09) [6]	0.5 (0.11-0.89)	0.03	0.50	0.83
WFIKKN2	C9J6G4	end	14.53 (0.85) [9]	14.72 (1.15) [10]	0.57 (0.27-0.87)	14.77	0.60	0.89
YWHAB	P31946	end	13.01 (1.13) [9]	13.24 (1.3) [7]	0.54 (0.23-0.85)	12.86	0.57	0.67
YWHAE	P62258	end	14.58 (1.02) [9]	14.67 (1.25) [10]	0.6 (0.32-0.88)	14.36	0.80	0.56
YWHAE	P62258	start	15.15 (0.74) [6]	15.55 (3.03) [7]	0.55 (0.19-0.91)	14.67	0.57	0.83
YWHAE	P62258	change	0.04 (0.1) [5]	0.04 (0.2) [7]	0.6 (0.25-0.95)	0.05	0.71	0.60
YWHAG	P61981	end	13.03 (1.14) [10]	13.7 (1.09) [8]	0.66 (0.39-0.93)	14.50	0.38	1.00
YWHAQ	P27348	end	14.3 (1.18) [10]	13.36 (0.75) [8]	0.71 (0.45-0.97)	13.88	0.88	0.70
YWHAQ	P27348	start	13.45 (1.07) [6]	13.85 (0.74) [7]	0.6 (0.24-0.95)	12.85	1.00	0.33
YWHAZ	P63104	change	-0.06 (0.12) [9]	-0.06 (0.09) [8]	0.54 (0.23-0.85)	0.03	1.00	0.33
YWHAZ	P63104	start	14.16 (1.33) [11]	14.42 (0.89) [8]	0.55 (0.26-0.83)	13.10	1.00	0.27
YWHAZ	P63104	end	15.08 (0.75) [10]	15.31 (1.14) [10]	0.61 (0.33-0.89)	15.24	0.70	0.70
ZNF511-PRAP1	H7BY64	end	13.9 (0.45) [8]	14.02 (0.78) [8]	0.52 (0.2-0.83)	13.41	0.38	0.88

SAH: subarachnoid hemorrhage; SD: standard deviation; N: number of patients; AUC: area under the curve; CI: confidence interval; sens: sensitivity; spec: specificity.
