# Supplementary material for: Proteomic profile and predictive markers of outcome in patients with subarachnoid hemorrhage
Source: Clin Proteomics. 2024 Jul 23;21:51. doi: 10.1186/s12014-024-09493-6 (PMC11267790; doi:10.1186/s12014-024-09493-6)
Supplement: Supplementary file 3 — Supplementary Material 3: Table 7 can be found in Supplementary file 3 [file 12014_2024_9493_MOESM3_ESM.pdf]

Supplementary Table 7 - Window Sizes

Compound	m/z	z	RT Time (min)	Window (min)	Isolation Window (m/z)
window 1	376	3	10.5	21	53.5
window 2	420.5	3	10.5	21	38
window 3	454	3	10.5	21	31
window 4	482.5	3	10.5	21	28
window 5	508.5	3	10.5	21	26
window 6	533	3	10.5	21	25
window 7	557	3	10.5	21	25
window 8	580.5	3	10.5	21	24
window 9	604	3	10.5	21	25
window 10	628	3	10.5	21	25
window 11	652.5	3	10.5	21	26
window 12	678.5	3	10.5	21	28
window 13	706	3	10.5	21	29
window 14	735.5	3	10.5	21	32
window 15	768.5	3	10.5	21	36
window 16	805.5	3	10.5	21	40
window 17	849	3	10.5	21	49
window 18	903	3	10.5	21	61
window 19	975.5	3	10.5	21	86
window 20	1099	3	10.5	21	163
window 21	1297	3	10.5	21	235
window 22	1531.5	3	10.5	21	236
